# Mechanistic insights into excited-state palladium catalysis for C–S bond formations and dehydrogenative sulfonylation of amines

**DOI:** 10.1038/s41467-023-42392-2

**Published:** 2023-10-19

**Authors:** Krishnamoorthy Muralirajan, Rajesh Kancherla, Bholanath Maity, Safakath Karuthedath, Frédéric Laquai, Luigi Cavallo, Magnus Rueping

**Affiliations:** 1 KAUST Catalysis Center, King Abdullah University of Science and Technology (KAUST), Thuwal, 23955-6900 Saudi Arabia; 2 KAUST Solar Center, King Abdullah University of Science and Technology (KAUST), Thuwal, 23955-6900 Saudi Arabia

**Keywords:** Reaction mechanisms, Photocatalysis, Homogeneous catalysis

## Abstract

Photocatalytic selective C(sp^3^)–H activation/cross-coupling reactions are appealing in organic synthesis. In this manuscript, we describe the development of photoexcited-state Pd-catalyzed dehydrogenative β-sulfonylation reactions using amines and aryl sulfonyl chlorides via intermolecular hydrogen atom transfer and C−S cross-coupling processes at room temperature. The transformation can be achieved by the direct generation of two distinct Pd-radical hybrid species and their capability to promote two different reactivities from Pd(0) and aryl sulfonyl chlorides, allowing for the efficient conversion of readily available amines into stable sulfonyl-substituted enamines at room temperature. The in-depth experimental, computational, and transient optical spectroscopic study and catalytic applications of a dehydrogenative functionalization event provide evidence for both static and dynamic quenching, as well as inner-sphere and outer-sphere mechanisms.

## Abstract (web summary)

Photocatalytic selective C(sp^3^)–H activation/cross-coupling reactions are appealing in organic synthesis. Here the authors describe the development of excited-state Pd-catalyzed dehydrogenative β-sulfonylation reactions using amines and aryl sulfonyl chlorides via intermolecular hydrogen atom transfer and C−S cross-coupling processes at room temperature.

## Introduction

**Fig. 1:**
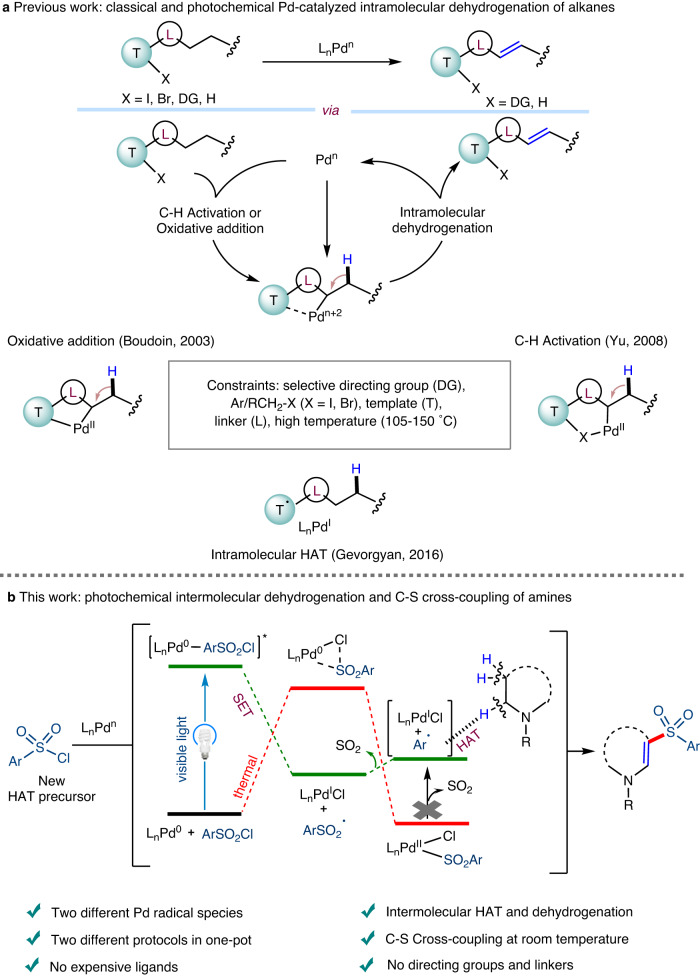
Palladium-catalyzed intra and intermolecular oxidative dehydrogenation and its challenges. **a** Previous work using classical and photochemical Pd-catalyzed intramolecular dehydrogenation of alkanes. **b** Our proposed photochemical merging of intermolecular dehydrogenation of amines and C-S cross-coupling in one-pot. L ligand, HAT hydrogen atom transfer, SET single electron transfer, Ar aryl, R alkyl.

The selective dehydrogenation of kinetically inert C(sp^3^)−H bonds adjacent to heteroatoms is a thermodynamically unfavorable process and requires harsh reaction conditions or sacrificial metal hydrogen acceptors^1,2^. Recent studies have suggested that the existence of an *N*-atom decreases the endothermicity of the reaction when compared to other alkanes^3^. However, most of the reported strategies are known to proceed via metal-nitrogen σ-coordination followed by β-hydride elimination to indirectly activate the adjacent α-C−H bond^4,5^. In contrast, the simultaneous oxidation of both α and β-C−H bonds in alkylamines remains challenging in homogeneous catalysis. Moreover, the availability of practical and sustainable methods for the transition-metal-catalyzed oxidative dehydrogenation of alkanes to alkenes has been limited due to the requirement of less favorable reaction conditions^6–9^. In particular, some strategies describing Pd-catalyzed intramolecular oxidative dehydrogenation reactions via C−H activation have been reported and they typically involve directed concerted metalation–deprotonation or intramolecular hydrogen atom transfer (HAT) mechanistic pathway (Fig. 1a)^10–16^. Among these, Gevorgyan’s pioneering work on the photo-induced generation of aryl or alkyl Pd-radical hybrid species from aryl/alkyl iodides has demonstrated the strategic importance of intramolecular oxidative dehydrogenation studies^14–16^. However, all above mentioned Pd-catalytic approaches to the reactions are typically restricted to intramolecular substrates and require higher temperatures, directing groups, selected linkers, expensive ligands, and aryl/alkyl halides. Thus, difficulties performing this transformation (selectivity and removal of linkers) and the reduced generality and practicality prompted us to address the question of whether Pd-radical hybrid species could be successfully utilized to carry out an intermolecular oxidative dehydrogenation reaction, followed by cross-coupling in one-pot (Fig. 1b).

Recently, photo-induced Pd-catalyzed cross-coupling reactions via the single electron oxidative addition of a C−X bond (X = I, Br, and COO-NR_1_R_2_) to Pd(0) to generate carbon-centered Pd(I)-radical hybrid species have attracted attention, because of their unusual reactivity in cross-coupling reactions at room temperature^17–19^. The reactivity of Pd-alkyl radical species has been extensively explored^20–28^, but the generation and reactivity of Pd-arene/vinyl radical hybrid species^14,16,29–31^ have been rarely reported. Pioneering studies have shown that arene Pd-radical hybrid species play a key role in the intramolecular 1,*n*-HAT step used to obtain alkenes from alkanes. However, the use of iodoarenes with selected linkers and ferrocene-type phosphine ligands to generate arene Pd-hybrid radical species is considered less practical. Moreover, all recent photoinduced Pd-catalytic developments were focused on a single-cycle process for either C–C cross-coupling or dehydrogenation reactions to validate the reactivity of carbon-centered Pd radical species. Based on our recent results in mechanism-based reaction development^32^ and excited-state Pd catalysis^24,33,34^, we aimed to explore dehydrogenative C–S cross-coupling reactions, previously achieved with other methods^9,35–38^ via an intermolecular HAT process with sufficient mechanistic details in order to open a more effective strategy for the discovery of unusual photoinduced Pd catalytic reactions at room temperature which avoid the requirement of elevated temperatures typically associated with the oxidative addition^39–41^. Just recently, Gevorgyan et al. reported an allylic C–H amination of alkenes with amines via intermolecular HAT using aryl bromide as the HAT reagent^42^. To accomplish an intermolecular Pd-catalysed dehydrogenation process using an arene (HAT) radical, several principles need to be considered: (i) selectivity and stability - the use of highly reactive radical intermediates capable of selective H-abstraction (Csp^2^ radicals are very short-lived intermediates, and rate of H-abstraction is on the order of 10^6^ M^–1^ s^–1^)^43^; (ii) molecular interactions - examination of the intermolecular atomic interactions (Csp^3^−H···arene radical) based on enthalpy, polar effects, distance, and ideal arrangement of the atoms^44–47^; (iii) practicability and versatility - the use of inexpensive additives, ligands, aryl, and alkyl substrates.

## Results and discussion

### Development of oxidative dehydrogenation

**Fig. 2:**
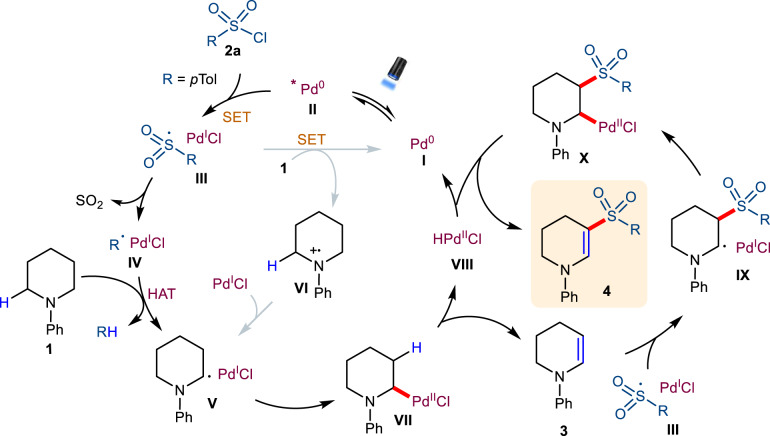
The proposed mechanism for the dehydrogenative C-S cross-coupling reaction. HAT hydrogen atom transfer, SET single electron transfer, R aryl.

With these considerations in mind, we first exposed *N*-phenyl piperidine (desaturation source) and iodobenzene (HAT source) to blue-light irradiation in the presence of a Pd(OAc)_2_ catalyst, PPh_3_ ligand, and Cs_2_CO_3_ base in benzene. Interestingly, we obtained the expected 1-phenyl-1,2,3,4-tetrahydropyridine product (**3**) in 24% yield (see supplementary information for further details), which supported our intermolecular HAT hypothesis. With this interesting result in hand, we further investigated the use of aryl sulfonyl chlorides to execute both the intermolecular HAT and cross-coupling reagent in the one-pot medium by the anticipated generation of two different radical hybrid species [ArSO_2_ Pd(I) and Ar^•^ Pd(I)] under visible-light irradiation. As expected, we found the formation of 1-phenyl-5-tosyl-1,2,3,4-tetrahydropyridine **4** in 54% yield at room temperature. From these initial results, the hypothetical mechanism for the dehydrogenative functionalization of alkylamines in the presence of tosyl chloride (**2a**) using Pd-catalysis is outlined in Fig. 2.

### Proposed mechanism

Visible-light excitation of the [Pd(0)L_n_] photocatalyst (**I**) is known to generate the excited-state *Pd(0) complex **II**^17^, which can promote the single electron transfer (SET) process with **2a** via an expected inner sphere mechanism to yield the ClPd(I)Ts^•^ hybrid species (**III**). Notably, the reduction potential of **2a** [E_1/2_^red^ = –0.94 V vs. SCE]^48^ is higher than the excited-state oxidation potential of *Pd(PPh_3_)_4_ [E(M^+^/M*) = −1.72 V vs. SCE]^23^. Therefore, we cannot completely rule out the possibility of an outer sphere mechanism based on the redox potential. Subsequently, intermediate **III** generates a phenyl radical via the extrusion of SO_2_. The resulting Pd(I) phenyl radical (**IV**) can participate in an intermolecular HAT process with *N*-phenyl amine **1a** to afford the α-amino carbon radical intermediate **V** and toluene. Concurrently, the Pd(I) species undergoes recombination with **V** to generate the Pd(II) intermediate **VII**. In another pathway, the Pd(I) species could also undergo a single electron reduction with **1a** to give the amino radical cation intermediate **VI** and Pd(0) via an outer sphere reductive quenching mechanism^22^. Subsequently, **VI** rapidly promotes a [1,2]-radical shift with the most hydridic α-C–H bond to give α-amino radical **V**, which undergoes radical recombination with the excess Pd(I) species to afford intermediate **VII**. However, this route was ruled out by comparing the redox potentials of *N*-phenyl amines (E_1/2_^ox^ = 0.74 V vs SCE for *N*-phenyl pyrrolidine in CH_3_CN)^49^ and Pd(I) species (E_1/2_^red^ = +0.262 V vs SCE for [Pd(PPh_3_)_4_]^+^ in THF)^23^. Also, the single-electron oxidation of *N*-phenyl pyrrolidine by Pd(I) species is endergonic by 35 kcal/mol (Supplementary Fig. 6). Intermediate **VII** undergoes β-hydride elimination to produce **3** and the regenerated Pd(0) catalyst **I**. Finally, intermediate **3** undergoes C−S cross-coupling in the presence of excess Ts^•^ to afford 1-phenyl-5-tosyl-1,2,3,4-tetrahydropyridine **4** (Fig. 2, and Supplementary Fig. 7). In addition to **4**, we also observed an excess amount of toluene and a small amount of 4,4’-dimethyl-1,1’-biphenyl as by-products, which strongly supports our hypothesis. The key results achieved upon studying various reaction parameters are summarized in Table 1. We stress that substituted vinyl sulfones are an important functional group in biologically-active compounds (e.g., potent inhibitors of cysteine protease and HIV-1 integrase)^50–52^ and also used in divergent synthetic organic transformations due to the usefulness of the sulfone group^53–55^.

**Table 1:** Optimization of reaction conditions^a^


Entry	Change in the standard reaction condition	Yield of 5 (%)
1	none	93 (88)
2	Change in Pd source: Pd(PPh_3_)_4_, Pd(PPh_3_)_2_Cl_2_	84, 86
	PdCl_2_, PdI_2_, PdBr_2_	49, 54, 37
3	Change in base: Cs_2_CO_3_, Na_2_CO_3_	75, 34
	K_3_PO_4_, K_2_HPO_4_	77, 21
	DABCO, Quinuclidine	41, 23
4	Change in ligand: P(1-Nap)_3_ (20 mol%), Xantphos (10 mol%)	95, 61
	*rac*-BINAP (10 mol%), DPEPhos (10 mol%)	89, 91
	PCy_3_ (20 mol%)	84
5	Change in solvent: THF, PhCl, CH_3_CN	83, 80, 45
	DMA, DME	36, 62
6	No light or Pd or ligand	0, 0, 0
7	at 100 °C (absence of light)	trace

^a^Unless otherwise mentioned all reactions were carried out using 1-(*p*-tolyl)pyrrolidine (0.2 mmol), tosyl chloride (0.6 mmol), [Pd] (5 mol%), ligand (10–20 mol%), base (3 equiv), and solvent (0.1 M, 2.0 mL) by blue LEDs irradiation (34 W) with fan cooling (to keep the reaction at room temperature) for 48 h under Ar. Yields were determined by ^1^HNMR analysis of the crude mixture relative to trimethoxy benzene as an internal standard and isolated yield in parentheses.

### Scope of substrates

**Fig. 3:**
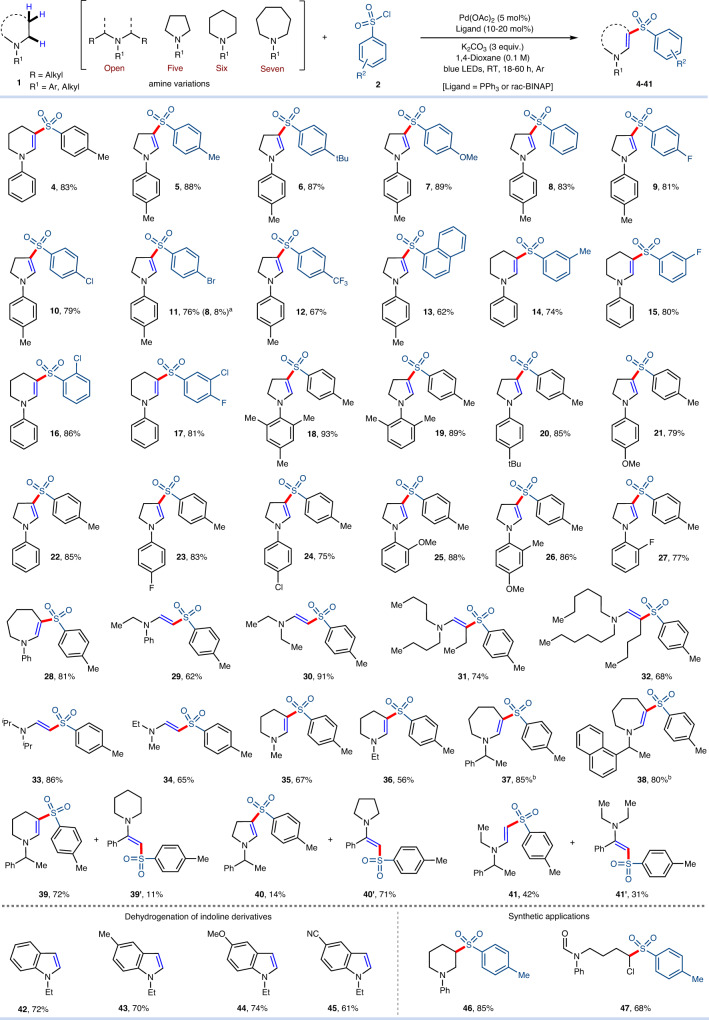
Substrate scope of photoinduced Pd-catalyzed dehydrogenative sulfonylation reactions and its applications. Amines and sulfonyls scope. Reaction conditions: amines (0.2 mmol), arylsulfonyl chlorides (0.6 mmol), K_2_CO_3_ (0.6 mmol), Pd(OAc)_2_ (0.01 mmol, 5 mol%), ligand (PPh_3_, 0.04 mmol or *rac*-BINAP, 0.02 mmol), dry 1,4-dioxane (0.1 M), room temperature, irradiation with 34 W blue LEDs with fan cooling under Ar. All yields are isolated yields. ^a^7% Debromination product was observed. ^b^<5% Exocyclic sulfonylation product was observed. BINAP, 2,2′-bis(diphenylphosphino)-1,1′-binaphthyl.

With the optimal reaction conditions in hand, we applied this photocatalytic protocol to a range of amines and aryl sulfonyl chlorides to investigate the scope of the reaction (Fig. 3). A variety of aryl sulfonyl chloride derivatives containing both electron-donating (ED) or electron-withdrawing (EW) groups react smoothly with 1-(*p*-tolyl)pyrrolidine and 1-phenylpiperidine to give their corresponding dehydrogenative sulfonylated products (**4–17**) in excellent yields and with high selectivity. Subsequently, the electronic properties of the *N*-phenyl pyrrolidines also revealed a significant role in the oxidative dehydrogenative functionalization reaction. For example, substrates bearing ED substituents gave their corresponding products in excellent yields compared to those bearing EW substituents (**18–27**), due to the low energy required to remove the α-H-atom from the electron-rich amines.

Interestingly, we also observed that seven-membered *N*-phenyl amine and open-chain (*N*,*N*-diethyl)phenyl amine could lead to vinyl-sulfonylated products (**28–29)** in 81% and 62% yields, respectively. The reactivity difference suggests that electron-rich amines exhibit high efficiency in the oxidative dehydrogenation process. Likewise, we also noticed that simple electron-rich aliphatic amines, such as triethyl, butyl, and hexyl amines, undergo efficient dehydrogenative alkene functionalization reactions, resulting in the formation of more stable vinyl sulfones (**30–31**) with excellent yields. Remarkably, this intermolecular HAT strategy can also be applied to more complex unsymmetrical tertiary amines such as diisopropyl ethylamine, diethyl methylamine, *N*-methyl piperidine, *N*-ethyl piperidine, 1-(1-phenylethyl)azepane, and 1-(1-(naphthalen-1-yl)ethyl)azepane without compromising any proximal regio-centers. These results show that β-tosyl substituted endo/exo cyclic enamines **32–37** can be prepared in good yields and with high selectivity, which enables further functional group modification. Moreover, the selectivity was altered by changing the ring size of the amine, such as piperidine, pyrrolidine, and *N*-ethyl amines, leading to highly separable β-tosyl substituted enamines **38–40** with moderate selectivity. Finally, the dehydrogenation of indolines containing various functional groups was also investigated and gave their corresponding aromatized products (**41–44)** in good yields. To show the functional group versatility of the enamines obtained in this strategy as templates for further diversification, selective hydrogenation (**46**) and incorporation of chlorine atom (**47**) were realized to afford the saturated cyclic and ring-opened amine products in good yields (Supplementary Fig. 35).

### Computational study

**Fig. 4:**
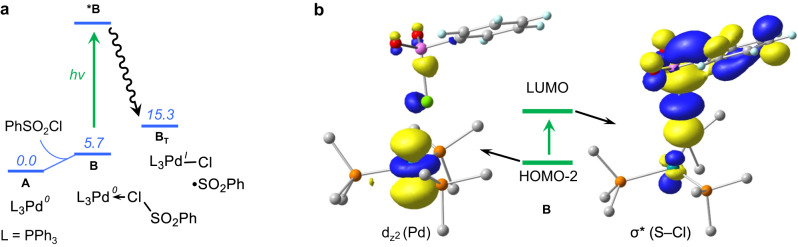
Mechanism of photo absorption of Pd(0)-complex. **a** Single electron transfer (SET) step. **b** KS-MO’s of **B** involved in the vertical excitation at λ_max_ = 431 nm. The energy values are at the M06(SMD)/SDD/Def2-TZVP//PBE0/SDD/Def2-SVP level of theory.

**Fig. 5:**
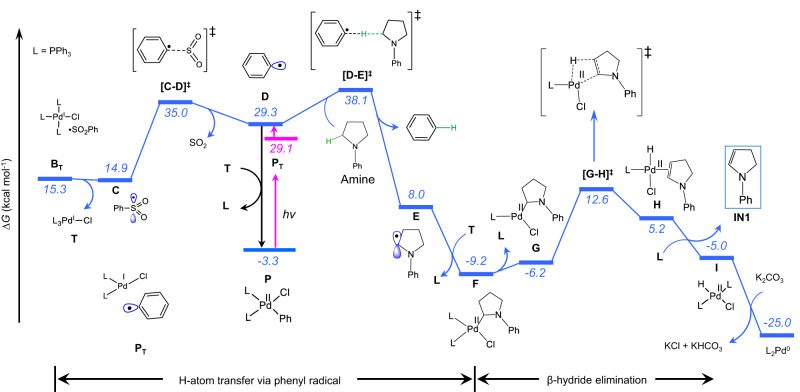
Computed energy profile for the dehydrogenation of amine. For energy conventions refer Fig. 4.

To understand the mechanism of the excited-state palladium-catalyzed dehydrogenative C‒S cross-coupling reaction, we performed density functional theory (DFT) calculations (See Computational Details in Supplementary Information). The overall pathway is divided into two major sections: dehydrogenation and C‒S coupling. Photoexcitation of L_n_Pd(0) to activate the benzene sulfonyl chloride (PhSO_2_Cl) reagent via SET is the common starting step in both sections. Among the different number of phosphine-ligated Pd(0) species, the tri-coordinated (PPh_3_)_3_Pd(0), **A**, is the most stable one and thus we used it as the initial catalytic species (Supplementary Fig. 1). The formal two-electron oxidative addition of PhSO_2_Cl to **A** is not considered due to a very high activation barrier (∆*G*^‡^ = 36.5 kcal/mol, Supplementary Fig. 2). Reaction of **A** into the reactive intermediate (PPh_3_)_3_Pd(I)‒Cl and sulfonyl radical, **B**_**T**_, can occur along two pathways. In the former^24^, upon photoexcitation, **A** (λ = 418 nm) is promoted to the triplet state **A**_**T**_, which interacts with PhSO_2_Cl and, via barrier-less SET, leads to **B**_**T**_ (Supplementary Figs. 3–5). In the alternative pathway, PhSO_2_Cl binds to **A** in an endergonic step (5.7 kcal/mol) to generate **B** before photoexcitation (Fig. 4a). TD-DFT calculations in combination with natural population analysis (NPA) reveal that **B** is excited in the visible light region (λ = 431 nm), triggering charge transfer from the Pd d_z_^2^ orbital to the PhO_2_S‒Cl σ* bond orbital resulting in the cleavage of the S‒Cl bond (Fig. 4b). From the possible excitations, the former one is less feasible because of lower oscillator strength (*f* = 0.005 vs. 0.294 for **A** and **B** respectively, Supplementary Table 2). The excited-state electronic structure derived from the DFT calculations (Fig. 4) is supported by photophysical characterizations based on quenching experiments and time-resolved spectroscopy (*vide infra*). The triplet-state intermediate **B**_**T**_ decomposes into two doublet species, namely (PPh_3_)_3_Pd(I)‒Cl (**T**) and the sulfonyl radical **C** (Fig. 5). Successively, in an outer sphere mechanism **C** is decomposed into the phenyl radical **D** and SO_2_ via transition state **[C-D]**^**‡**^ with an estimated free energy barrier of 20.1 kcal/mol (Supplementary Fig. 12)^56–58^. The competitive inner sphere mechanism is disfavored by a higher energy barrier of 28.3 kcal/mol, which indicates an outer sphere phenyl radical formation (Supplementary Fig. 11)^59^. The phenyl radical serves as a HAT reagent^57,60^ and activates the inert C(sp^3^)‒H bond of the α-amino substrate (Supplementary Fig. 8a, HAT steps by different radical species are shown in Supplementary Fig. 8b, c). An overall activation barrier of 23.2 kcal/mol from **C** is required for the HAT step, which proceeds through the transition state **[D-E]**^**‡**^, and leads to the formation of the α-amino alkyl radical **E** with the liberation of benzene. In the following step, **E** is readily captured by (PPh_3_)_3_Pd(I)‒Cl to generate the Pd(II)(Cl)-alkyl intermediate **F** with the loss of one phosphine ligand. Slightly endergonic dissociation of a phosphine ligand from **F** generates **G**, which has a vacant coordination site on Pd facilitating a β-hydride elimination step via transition state **[G-H]**^**‡**^. The overall free energy barrier from **F** is 21.8 kcal/mol. The pathway is slightly favored over the direct β-hydride elimination from **F**, which requires an activation barrier of 23.9 kcal/mol via the transition state **[F-I]**^**‡**^ (Supplementary Fig. 13). The resulting intermediate **H** includes the dehydrogenated amine, which is then separated as **IN1** by the replacement with a phosphine ligand. On the other hand, the (PPh_3_)_2_Pd(II)HCl species formed in the β-hydride elimination step is reduced to (PPh_3_)_2_Pd(0) in the presence of K_2_CO_3_ (Fig. 5). The energy profile for the dehydrogenation step reveals that the HAT occurs via an outer sphere mechanism (Fig. 5). For completeness, the competitive alternative pathways of C(sp^3^)‒H bond activation, including inner sphere mechanism, are disfavored by clearly higher activation barriers (for details refer Supplementary Fig. 11).

**Fig. 6:**
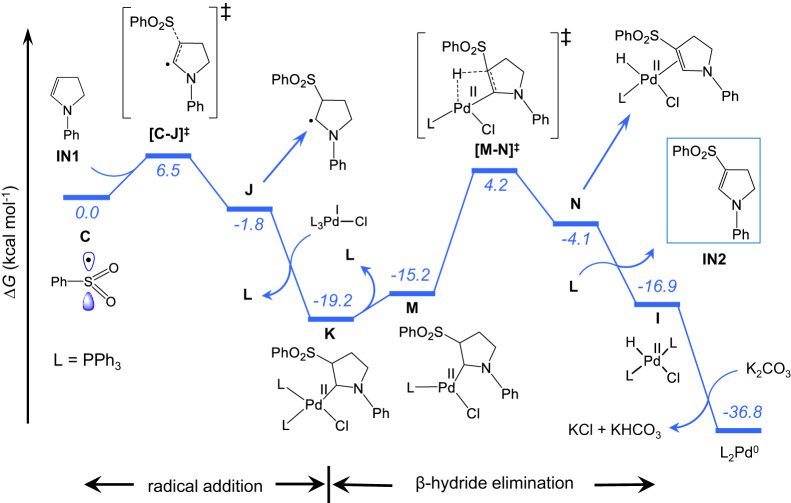
Computed energy profile for the C‒S cross-coupling step. For energy conventions refer Fig. 4.

As discussed beforehand, to initiate the second step another molecule of PhSO_2_Cl must be activated by the photoexcited Pd(0) catalyst via SET. The resulting sulphonyl radical (**C**) reacts with **IN1** leading to the carbon-centered radical **J** via the transition state **[C-J]**^**‡**^ with an activation barrier of 6.5 kcal/mol. In the next step, **J** promptly binds with (PPh_3_)_3_Pd(I)‒Cl to produce intermediate **K** with dissociation of one phosphine ligand, a step exergonic by 17.4 kcal/mol. Similar to the previous step, one more phosphine ligand dissociates from **K** with an endergonicity of 4.0 kcal/mol. The resulting intermediate **M** undergoes β-hydride elimination via the transition state **[M-N]**^**‡**^ with an activation barrier of 23.4 kcal/mol from **K** (Fig. 6 and Supplementary Fig. 14). The C−S cross-coupling product **IN2** is liberated from **N** upon replacement by a phosphine ligand. Similar to the first step, the (PPh_3_)_2_Pd(II)HCl species is transformed into (PPh_3_)_2_Pd(0) and initiates another catalytic cycle.

### Mechanistic studies

**Fig. 7:**
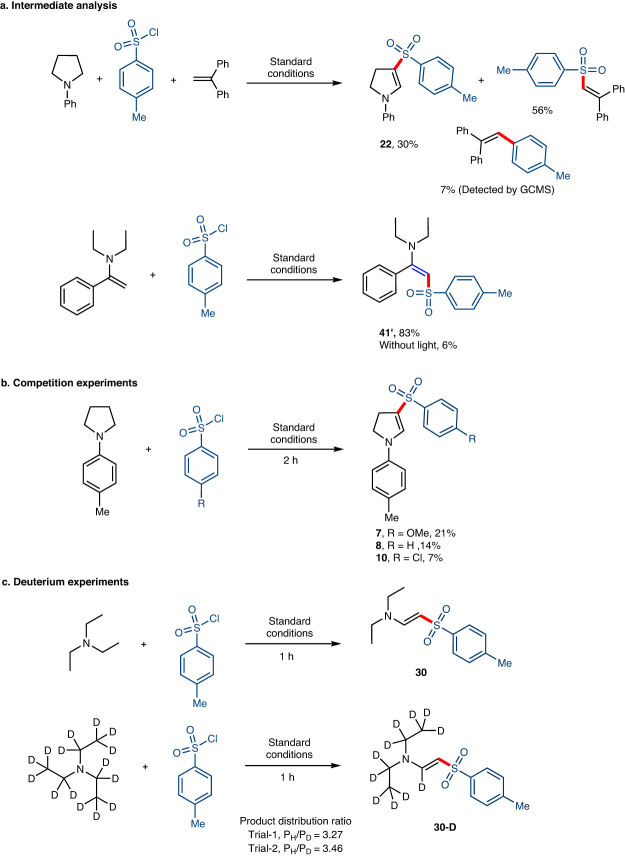
Mechanistic related experiments. **a** Radical intermediate experiment. **b** Competition experiments using different aryl sulfonyl chlorides. **c** Intermolecular competition experiment between triethyl amine and d_15_-triethyl amine.

To gain more insight into the mechanism of intermolecular HAT described above, a series of mechanistic studies were carried out (see Supplementary Information for further detailed experiments and results) and all preliminary experiments (Supplementary Figs. 16–20) support the generation of both sulfonyl and arene radical intermediates by Pd-catalysis. For example, when the reaction was performed in the presence of 1,1-diphenylethylene, the tosyl radical addition product, namely (2-tosylethene−1,1-diyl)dibenzene was observed in 56% yield along with **22** (Fig. 7a). Similarly, in order to prove that the C−H sulfonylated product was formed via intermediate **IN1**, we performed a reaction using *N*,*N*-diethyl-1-phenylethen-1-amine, and **2a** under the standard reaction conditions, which gave **41’** in 83% yield (Fig. 7a). These results indicate that the intermolecular HAT process is initiated by the sulfonyl radical. Additionally, we also performed “on-off” light switching experiments, and no reactivity was observed after stirring in the absence of visible-light irradiation (Supplementary Fig. 17). The repeated “on–off” cycles demonstrate that the dehydrogenative C−S cross-coupling process requires continuous visible-light irradiation and show that the radical chain mechanism is not operative. Notably, we did not observe the C(sp^3^) sulfonylated product in the presence of *N*,*N*-dimethylaniline, suggesting that radical-radical cross-coupling can be ruled out in this proposed reaction cycle (Supplementary Fig. 18). It is important to mention that our competition experiments suggest that the reduction potential of the aryl sulfonyl chlorides also plays a pivotal role in promoting the HAT process. For example, 4-methoxyphenyl sulfonyl chloride (E_1/2_^red^ = −0.99 V vs. SCE) gave a higher yield than phenyl sulfonyl chloride (E_1/2_^red^ = −0.95 V vs. SCE) and 4-chlorophenylsulfonyl chloride (E_1/2_^red^ = −0.79 V vs. SCE) (Fig. 7b, and Supplementary Fig. 21)^48^. The observed reaction efficiency supports that the outer sphere mechanism may influence the rate of the intermolecular aryl radical H-atom abstraction process (**[D-E]**^**‡**^). Finally, to gain insights into the nature of C−H functionalization, we conducted competition experiments using triethylamine and d_15_-triethylamine (Supplementary Fig. 22). The product distribution of **30** and **30-D** was determined to be in the ratio of 3.27 (trial-1) and 3.46 (trial-2) (Fig. 7c). The observed high P_H_/P_D_ ratio indicates that the C–H bond-breaking process is predominantly achieved through a HAT pathway. In addition, the oxidation of the amine to generate intermediate **V** (as shown in Fig. 2) via the Pd(I)Cl intermediate through SET is not expected to occur kinetically under standard reaction conditions. We have also examined the feasibility of single-electron oxidation of *N*-phenyl pyrrolidine by DFT analysis. The calculated energy values indicate that the electron transfer process is unfavored due to very high endergonicity (Supplementary Fig. 6).

### Spectroscopic studies

Furthermore, steady-state along with time-resolved absorption and emission spectroscopy were used to substantiate the proposed reaction intermediates. The steady-state UV-Vis spectra recorded for each component showed that the Pd(0)(PPh_3_)_4_ complex absorbs light in the range of 400 to 500 nm, while the other reactants (i.e. *N*-phenyl pyrrolidine and **2a**) do not exhibit any absorption in the visible-light region (Supplementary Fig. 23). Similarly, the absorption spectrum obtained for a mixture of Pd(0) and *N*-phenyl pyrrolidine revealed that no chemical modification of the Pd(0) complex occurs prior to photoexcitation. However, the absorption spectrum recorded for a mixture of Pd(0) and **2a** displays a notable redshift, clearly indicating a ground state association of the two components in the solution (Supplementary Fig. 23). These experimental results can also be accounted for by the calculated sum of the Pd(0) and PPh_3_ absorption spectra (Supplementary Figs. 3, 15). Surprisingly, we noticed that the steady-state luminescence intensity of the Pd-catalyst increased upon increasing the concentration of *N*-phenyl pyrrolidine (Supplementary Figs. 24, 25) due to the fluorescence of *N*-phenyl pyrrolidine (λ_em_ = 420 nm, Supplementary Fig. 26). In addition, steady-state Stern-Volmer analysis supported the quenching of the excited state of the Pd(0) by **2a** (Supplementary Fig. 27), and the Stern–Volmer plot exhibits a quadratic correlation between the observed excited-state luminescence of the Pd(0) complex and the concentration of **2a** (Supplementary Fig. 28).

**Fig. 8:**
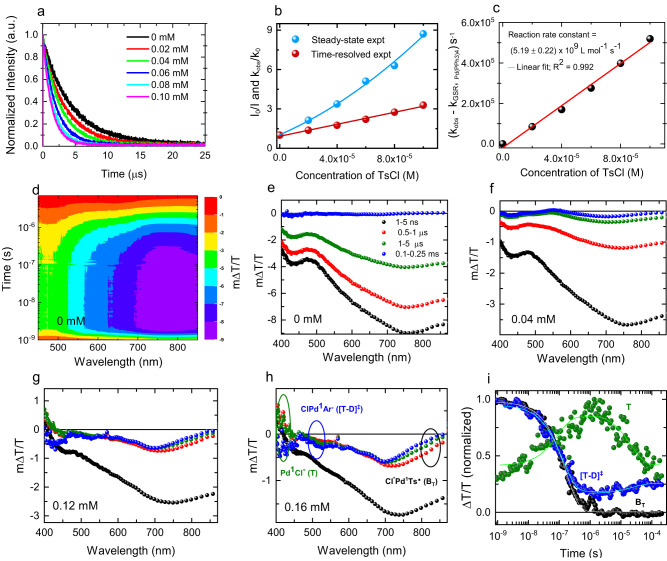
Photophysical experiments. **a** Photoluminescence dynamics of Pd(0) in the absence and presence of different concentrations of TsCl. **b** Combined steady-state and time-resolved Stern-Volmer plots of Pd(0) in the presence of different concentrations of TsCl. **c** Reaction rate as a function of TsCl concentration (filled dots) and linear fit to the data to determine the reaction rate constant; total observed rate (k_obs_); ground state recovery rate (k_GSR_). **d** The surface plot of ns-µs transient absorption of Pd(0) in the absence of TsCl. **e**–**h** Selected TA spectra of Pd(0) at the times indicated in the absence of TsCl (**e**) and in the presence of 0.04 mM (**f**), 0.12 mM (**g**), and 0.16 mM (**h**) TsCl. **i** TA dynamics extracted at spectral positions indicated in (**h**) (circles) assigned to the different species shown in Fig. 5.

In contrast, the Stern–Volmer plot obtained from time-resolved spectroscopy (Fig. 8a, and Supplementary Fig. 29) exhibits a linear correlation (Fig. 8b, Supplementary Fig. 30). Altogether, these results demonstrate that the photoexcited *Pd(0) complex is quenched by **2a** via a SET step, and both static and dynamic quenching proceed simultaneously in this system. Further analysis of the time-resolved photoluminescence measurements revealed that the excited-state lifetime of Pd(PPh_3_)_4_ is 4.4 ± 0.017 µs when monitoring its maximum emission at 630 nm in THF (Supplementary Fig. 32). An excited-state electron transfer rate constant (k_ET_) of 5.19 ± 0.22 × 10^9^ L mol^−1^ s^–1^ was determined using a linear plot of the total observed rate (k_obs_) − the ground state recovery rate (k_GSR_) versus the **2a** concentration (Fig. 8c, Supplementary Fig. 31).

Figure 8d–i show the nanosecond transient absorption (ns-TA) spectra of neat L_n_Pd^0^ and of L_n_Pd^0^ in the presence of different quencher (**2a**) concentrations. The positive signals in the spectra represent the photo bleach (PB), while the negative signals of the spectra represent photo-induced absorption (PA). Figure 8d, e shows the ns-TA spectra of neat L_n_Pd^0^ after excitation at 355 (λ_max_) nm. The feature observed at 700–850 nm was assigned to triplet-induced absorption of *(L_n_Pd^0^). *(L_n_Pd^0^) decayed with an inverse rate constant of 2.85 ± 0.008 µs (Supplementary Fig. 34). Upon addition of 4 × 10^−5^ M quencher (**2a**, Fig. 8f), the decay of *(L_n_Pd^0^) became significantly faster, and in addition, a slight blue shift and an emergence of a band centered around 690 nm were observed, which we assign to ClPd^I^Ts^•^ (**B**_**T**_), that is, the radical hybrid species formed by the decay of *(L_n_Pd^0^). This is evidence of the single electron transfer process from *(L_n_Pd^0^) to TsCl via excited-state oxidative quenching. Interestingly, further addition of quencher molecules did not only enhance the quenching rate but also led to the observation of spectral features of products generated from the decay of the **B**_**T**_ radical intermediate (Fig. 8g, h, and Supplementary Fig. 33). More precisely, the PB band that emerged at 400 nm can be assigned to Pd^I^Cl (**T**) and its decay promotes the generation of ClPd^I^Ar^•^ (**[T-D]**^**‡**^), which leads to the rise of the band at 500 nm (Fig. 8g, h). Figure 8i shows the integrated dynamics of selected spectral regions (circles in Fig. 8h) and the corresponding exponential fits. The fit yielded an inverse rate constant of 163 ±0.03 ns for the decay of **B**_**T**_ and 91 ± 0.05 ns for the generation of **T**, respectively. The generation dynamics of **[T-D]**^**‡**^ yielded 24 ± 4 µs, consistent with the decay of **T** (21 ± 4 µs). Altogether, these time-resolved photophysical and mechanistic experiments support that a SET process is operative via two distinct Pd(I) radical hybrid species.

In summary, we have developed dehydrogenative β-sulfonylation reactions using amines and aryl sulfonyl chlorides via intermolecular HAT and C−S cross-coupling process at room temperature. Furthermore, we demonstrated the direct generation of two distinct Pd-radical hybrid species and their capability to promote two different reactivities from Pd(0) and aryl sulfonyl chlorides, which permits efficient conversion of readily available amines into stable enamines at room temperature. Control experiments, as well as computational and transient optical spectroscopic studies clearly show that both static and dynamic quenching, as well as inner sphere and outer sphere mechanisms are operative. Moreover, this work also is an example of an in-depth experimental, computational, spectroscopic study and catalytic application of a dehydrogenative functionalization event. We believe that this methodology can be applied to the selective dehydrogenation of more complex molecules and new cross-coupling transformations. The mechanistic insight of the reactive species is also of relevance to the further development of photoexcited-state Pd-catalysis and will inspire the design of tailored catalysts and excited-state catalysis reactions.

## Methods

### General procedure for photoinduced palladium-catalyzed dehydrogenative sulfonylation

A clean, oven-dried screw cap reaction tube equipped with a Teflon-coated magnetic stir bar was charged with amines (0.2 mmol, 1 equiv.), aryl sulfonyl chlorides (0.6 mmol, 3 equiv.), Pd(OAc)_2_ (2.3 mg, 0.01 mmol, 5 mol%), ligand (PPh_3_, 10.5 mg, 0.04 mmol, 20 mol% or *rac*-BINAP, 12.4 mg, 0.02 mmol, 10 mol%), and K_2_CO_3_ (82.8 mg, 0.6 mmol, 3 equiv.). The reaction tube was capped with a rubber septum, evacuated, and backfilled with argon (3 times). Then, degassed 1,4-dioxane (0.1 M, 2.0 mL) was added via a syringe. The reaction mixture was stirred at room temperature for 18–60 h under irradiation with 34 W blue LEDs (3 cm away from blue LEDs) with fan cooling. Upon completion, the reaction was quenched via exposure to air. The reaction mixture was diluted with EtOAc and filtered through a small bed of Celite and concentrated in vacuo. The residue was purified by column chromatography using aluminum oxide (~150 mesh size) and *n*-hexane/ethyl acetate as the eluent. All the compounds were fully characterized (see Supplementary Information).

### Supplementary information

Supplementary Information

Peer Review File

### Source data

Source Data

## Data Availability

The authors declare that all other data supporting the findings of this study are available within the article and its Supplementary Information files. The experimental procedures and characterization of all new compounds are provided in the Supplementary Information file. For the energies and Cartesian coordinates, see Supplementary source file. All data are available from the corresponding author upon request. Source data are provided with this paper.
