# Supplementary material for: Mechanistic insights into excited-state palladium catalysis for C–S bond formations and dehydrogenative sulfonylation of amines
Source: Nat Commun. 2023 Oct 19;14:6622. doi: 10.1038/s41467-023-42392-2 (PMC10587301; doi:10.1038/s41467-023-42392-2)
Supplement: Supplementary file 1 — Supplementary Information [file 41467_2023_42392_MOESM1_ESM.pdf]

Supplementary Information

Mechanistic Insights into Excited-state Palladium Catalysis for C–S bond Formations and Dehydrogenative Sulfonylation of Amines

Krishnamoorthy Muralirajan,[1,3] Rajesh Kancherla,[1,3] Bholanath Maity,[1] Safakath Karuthedath,[2] Frédéric Laquai,[2]* Luigi Cavallo,[1]* and Magnus Rueping[1]*

[1]KAUST Catalysis Center, King Abdullah University of Science and Technology, Thuwal 23955-6900, Saudi Arabia.

[2]KAUST Solar Center, King Abdullah University of Science and Technology, Thuwal 23955-6900, Saudi Arabia.

[3]These authors contributed equally to this work.

email: frederic.laquai@kaust.edu.sa

email: luigi.cavallo@kaust.edu.sa

email: magnus.rueping@kaust.edu.sa

Table of Contents

General information
Optimization studies
General procedure for the catalytic reactions
Computational methods and details
TEMPO trapping experiment
On-off experiments
Radical-radical cross coupling experiment
Intermediate experiment
Competition experiments
Different arene radical generation experiments
Comparison of UV-Visible experiments and emission spectrum of blue LEDs
Stern-Volmer quenching experiments
Time-resolved emission experiments
Time-resolved absorption experiments
Transformations of obtained aminovinyl sulfones
Characterization data
NMR spectra
References

General Information:

Unless otherwise stated, all reactions were carried out under an argon atmosphere in screw cap reaction tubes. All the reagents and solvents were bought from Sigma Aldrich and Alfa Aesar in a sure-seal bottle and were used as received. THF, CH_3CN, DCM, and Et_2O were distilled over sodium-lead alloy (Solvona®) under argon.

Palladium catalyst and ligands were obtained from Sigma Aldrich and Strem chemicals. For column chromatography, aluminum oxide (~150 mesh) from Aldrich was used. A gradient elution using *n*-Hexane and Ethyl Acetate were performed based on Merck aluminum TLC sheets (silica gel 60 F_{254}) and were visualized under UV light (254 nm) or by staining with aqueous potassium permanganate solutions or vanillin alcoholic solution. Organic solutions were concentrated under reduced pressure on a Büchi rotary evaporator using a water bath at 40 °C.

All isolated compounds are characterized by ^1H NMR, ^{13}C NMR, ^{19}F NMR spectroscopy, gas chromatography-mass spectra (GC-MS) and high-resolution mass spectra (HRMS). Copies of the ^1H NMR, ^{13}C NMR can be found in the supporting information. ^1H NMR spectra were recorded in deuterated solvents on Bruker Avance-II spectrometers at 400 or 500 MHz, with residual protic solvent as the internal standard ($CDCl_3$ referenced at 7.26 ppm, and DMSO as referenced at 2.50 ppm, respectively). ^{13}C NMR spectra were recorded in deuterated solvents on Bruker Avance-II spectrometers at 101 or 125 MHz, with the central peak of the deuterated solvent as the internal standard ($CDCl_3$ referenced at 77.16 ppm, and DMSO as referenced at 39.51 ppm, respectively). ^{19}F NMR spectra were recorded in deuterated solvents on Bruker Avance-II at 377 MHz. Chemical shifts (δ) are given in parts per million (ppm), and coupling constants (*J*) are given in Hertz (Hz) rounded to the nearest 0.1 Hz. The ^1H NMR spectra are reported as δ/ppm downfield from tetramethylsilane (multiplicity, number of protons, assignment, coupling constant *J*/Hz). The ^{13}C NMR spectra are reported as δ/ppm and were obtained with ^1H decoupling and if coupled to fluorine, multiplicity and coupling constant (Hz). Data are reported in the following order: chemical shift (δ) in ppm; multiplicities are indicated s (singlet), bs (broad singlet), d (doublet), t (triplet), q = quartet, dd = doublet of doublets and dt = doublet of triplets, m (multiplet); coupling constants (*J*) are in Hertz (Hz). All GCMS analyses were done by Agilent 7890A GC system connected with 5975C inert XL EI/CI MSD (with triple axis detector). High-resolution mass spectra (HRMS) analysis was performed using Bruker micro Time-of-Flight (TOF)-MS equipped with an ESI source. The mass scan range was set to 100–1500 *m/z*, with a resolving power of 100,000. FT-IR spectra were recorded on a Nicolet 6700 FT-IR (ATR) spectrometer and are reported in wavenumbers (cm^{-1}). UV-Vis absorption spectra were recorded on a Agilent Technologies Cary 60 UV-Vis spectrophotometer. Luminescence intensities were recorded using a fluoromax-4 spectrophotometer from Horiba Scientific.

Transient absorption (TA) spectroscopy was carried out using a home-built pump-probe setup. The output of a titanium: sapphire amplifier (Coherent LEGEND DUO, 4.5 mJ, 3 kHz, 100 fs) was split into three beams (2 mJ, 1 mJ, and 1.5 mJ). Two of them were used to separately pump

two optical parametric amplifiers (OPA) (Light Conversion TOPAS Prime). The TOPAS 1 generates tunable pump pulses, while the TOPAS 2 generates signal (1300 nm) and idler (2000 nm) only. TOPAS 2 was used to produce a white-light supercontinuum from 350 to 1100 nm by sending the 1300 nm pulses through a calcium fluoride (CaF_2) crystal which is mounted on continuously moving stage. The excitation light (pump pulse) was provided by an actively Q-switched Nd:YVO4 laser (InnoLas picolo AOT) frequency-doubled to provide pulses at 355 nm. The pump laser was triggered by an electronic delay generator (Stanford Research Systems DG535) itself triggered by the transistor– transistor logic (TTL) sync from the Legend DUO, allowing control of the delay between pump and probe with a jitter of roughly 100 ps. Pump and probe beams were focused on the sample. The sample solution was in a nitrogen filled 1 mm cuvettes (Hellma Analytics High Precision Cell made of quartz) .The transmitted fraction of the white light was guided to a custom-made prism spectrograph (Entwicklungsbüro Stresing) where it was dispersed by a prism onto a 512 pixel complementary metal-oxide-semiconductor (CMOS) the linear image sensor (Hamamatsu G11608- 512DA). The probe pulse repetition rate was 3 kHz and the excitation pulses were directly generated at 1.5 kHz frequency, while the detector array was read out at 3 kHz. Adjacent diode readings corresponding to the transmission of the sample after excitation and in the absence of an excitation pulse were used to calculate $\Delta T/T$ *(or DAS)*. Measurements were averaged over several thousand shots to obtain a good signal-to-noise ratio. The delay at which pump and probe arrive simultaneously on the sample (i.e., zero time) was determined from the point of the maximum positive slope of the TA signal rise for each wavelength. All reaction mixtures were irradiated with 34 W Kessil KSH150B from 4 cm away. Regular fans are employed to maintain the temperature at room temperature.

1. Screening for Pd-catalyzed intermolecular dehydrogenation reaction:

A clean, oven-dried screw cap reaction tube equipped with a PTFE-coated stir bar was brought into the Ar-filled glove box. $Pd(OAc)_2$ (2.3 mg, 0.01 mmol, 5 mol%), PPh_3 (10.5 mg, 0.04 mmol, 20 mol%), Cs_2CO_3 (195.0 mg, 0.6 mmol, 3 equiv.), 1-phenylpiperidine (32.2 mg, 0.2 mmol), additive (0.6 mmol), and degassed solvent (0.1 M, 2 mL) was added into the vial and stirred well for 5 min under the Ar-filled glove box and capped with Teflon septum. Then the reaction tube was removed from the glove box and placed 3 cm away from 34 W blue LEDs while stirring at room temperature (under fan cooling to keep the reaction at room temperature) for 48 h. After 48 hours, the reaction was quenched via exposure to air. The reaction mixture was diluted with EtOAc then trimethoxy benzene was added as the internal standard. Yields were determined by GCMS analysis of the crude mixture relative to trimethoxy benzene as an internal standard.

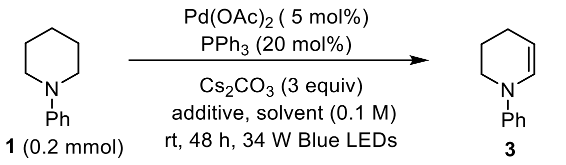

Entry	Additive	Solvent	Yield (%)
1	PhI (3 equiv)	benzene	24
2	PhI (3 equiv)	THF	trace
3	-	PhI	trace
4	PhCl (3 equiv)	benzene	trace
5	PhCl (3 equiv)	THF	7
6	-	PhCl	15
7	PhBr (3 equiv)	benzene	trace
8	PhBr (3 equiv)	THF	trace
9	-	PhBr	trace

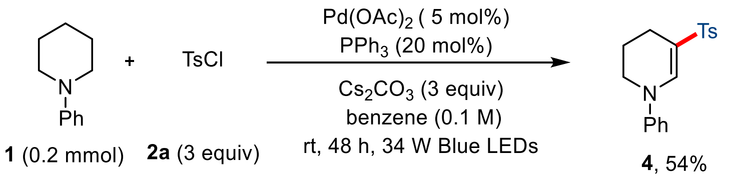

2. Optimization studies for Pd-catalyzed intermolecular dehydrogenation and sulfonylation:

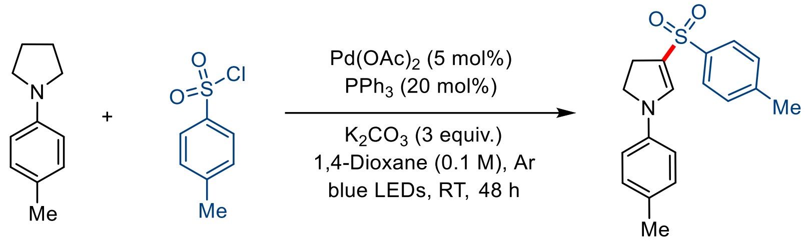

Entry	Change in the standard reaction condition	Yield (%)
1	none	93
2	PPh_3 (10 mol%)	85
3	$Pd(PPh_3)_4$ instead of $Pd(OAc)_2$	84
4	$Pd(PPh_3)_2Cl_2$ instead of $Pd(OAc)_2$	86
5	$PdCl_2$ instead of $Pd(OAc)_2$	49
6	PdI_2 instead of $Pd(OAc)_2$	54
7	$PdBr_2$ instead of $Pd(OAc)_2$	37
8	Change in base: Cs_2CO_3, Na_2CO_3	75, 34
	K_3PO_4, K_2HPO_4	77, 21
	DABCO, Quinuclidine	41, 23
9	Change in ligand: $P(1\text{-}Nap)_3$ (20 mol%)	95
	Xantphos (10 mol%)	61
	rac-Binap (10 mol%)	89
	DPEPhos (10 mol%)	91
	PCy_3 (20 mol%)	84
	PCy_3 (10 mol%)	78
10	Change in solvent: THF	83
	PhCl	80
	DCE	73
	DCM	71
	CH_3CN	45
	DMA	36
	DME	62
	DMSO	-
11	without irradiation	0
12	without palladium	0
13	without PPh_3	0
14	at 100 ℃ (absence of light)	trace

[a] **Supplementary Table 1**. Unless otherwise mentioned, all reactions were carried out using 1-(p-tolyl)pyrrolidine (0.2 mmol), tosyl chloride (0.6 mmol), [Pd] (5 mol%), ligand (10-20 mol%), base (3 equiv), and solvent (0.1 M, 2.0 mL) by blue LEDs irradiation (34 W) with fan cooling (to keep the reaction at room temperature) for 48 h under Ar. [b] Yields were determined by ^{1}H-NMR analysis of the crude mixture relative to trimethoxy benzene as an internal standard.

3. General procedure for Pd-catalyzed dehydrogenative C-H sulfonylation:

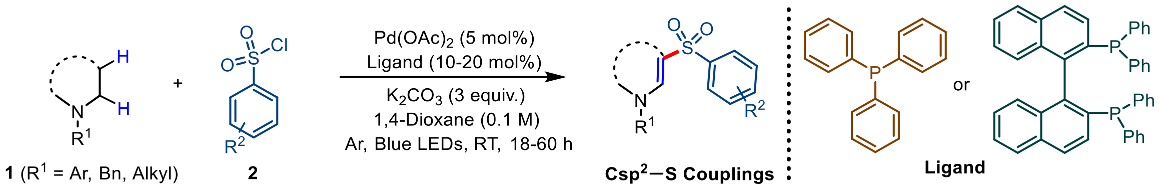

A clean, oven-dried screw cap reaction tube equipped with a Teflon-coated magnetic stir bar was charged with amines (**1**, 0.2 mmol, 1 equiv.), arylsulfonyl chlorides (**2**, 0.6 mmol, 3 equiv.), $Pd(OAc)_2$ (2.3 mg, 0.01 mmol, 5 mol%), ligand (PPh_3, 10.5 mg, 0.04 mmol, 20 mol% or *rac*-BINAP, 12.4 mg, 0.02 mmol, 10 mol%), and K_2CO_3 (82.8 mg, 0.6 mmol, 3 equiv.). The reaction tube was capped with a rubber septum, evacuated and backfilled with argon (3 times). Then, degassed 1,4-dioxane (0.1 M, 2.0 mL) was added via syringe. The reaction mixture was stirred at room temperature for 18-60 h under irradiation with 34 W blue LEDs (3 cm away from blue LEDs) with fan cooling. Upon completion, the reaction was quenched via exposure to air. The reaction mixture was diluted with EtOAc and filtered through a small bed of Celite and concentrated in vacuo. The residue was purified by column chromatography using aluminum oxide (~150 mesh size) and *n*-hexane/ethyl acetate as the eluent.

4. Computational Study:

Methods

All the geometries were optimized with the generalized gradient approximation (GGA) method with Gaussian 09 program packages,[1] using hybrid GGA DFT functional PBE0.[2] The electronic configuration of all the non-metal elements were described with the Ahlrichs split-valance polarization basis function Def2-SVP[3] while Pd is treated with the small-core, quasi-relativistic Stuttgart-Dresden effective core potential, with the associated triple-ζ valence basis set SDD.[4] The Geometries were optimized without any symmetry constraints. Harmonic force constants were computed at the optimized geometries to characterize the stationary points as minima or saddle points. All transition states were optimized using the default Berny algorithm implemented in the Gaussian09 package.[2] For further validation of energetics, single-point calculations were performed on the PBE0/SDD(Pd)/Def2-SVP(non-metals) optimized geometries using meta hybrid-GGA functional M06[5] employing a valence triple-ζ-type of basis set Def2-TZVP[6] for non-metals, and SDD(ECP) for Pd. The solvent effects (1,4-dioxane, $\varepsilon = 2.21$) were evaluated implicitly by a self-consistent reaction field (SCRF) approach for all the intermediates and transitions states, using the SMD continuum solvation model.[7] Unless otherwise specified, the ΔG was used throughout the text. The ΔG value was obtained by augmenting the ΔE_L^S energy terms at M06(SMD)/SDD(Pd)/Def2-TZVP(non-metals) with the respective free energy corrections at the PBE0/SDD(Pd)/Def2-SVP(non-metals) level in the gas phase. To compute the UV-Vis spectroscopy of Pd(0) complexes, TD-DFT calculations at M06(SMD)/SDD(Pd)/Def2-TZVP(non-metals) level of theory have been performed on the optimized geometries at

PBE0/SDD(Pd)/Def2-SVP(non-metals). As it is known that translational and rotational entropies in solution for association/dissociation processes are overestimated/underestimated, and that the deviation in the free energies is ~1.89 kcal mol^{-1} from the standard state (1 atm) to 1 M in solution we reduced by 1.89 kcal mol^{-1} the free energy change for association steps and we added 1.89 kcal mol^{-1} to the free energy change of the dissociation steps.[8] Ball and stick models are made from CYLView visualization programs.[9]

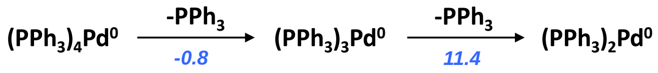

Supplementary Figure 1. Energetics of PPh_3 dissociation from initial Pd^0 catalyst. Free energies in solution at the M06(SMD)/SDD/Def2-TZVP//PBE0/SDD/Def2-SVP level are displayed.

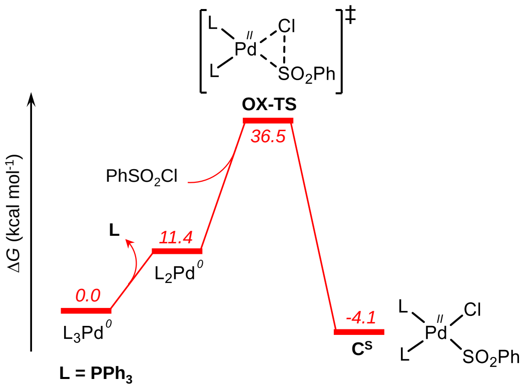

Supplementary Figure 2. Free energy barrier of formal two-electron oxidative addition of $PhSO_2Cl$ to L_nPd^0.

In line with our previous well-explored mechanism,[10] upon photoexcitation **A** is promoted to the triplet state $\mathbf{A_T}$, that interacts with the $PhSO_2Cl$ within SET mechanism. The single electron oxidation, a barrier less process, leads to $\mathbf{B_T}$, composed by $(PPh_3)_3Pd(I)–Cl$ and sulfonyl radical (Supplementary Fig. 3). In an alternative pathway, the $PhSO_2Cl$ binds to **A** with an endergonic step (5.7 kcal/mol) to generate **B** prior to photo absorption. However, the DFT results predict both mechanisms are possible, differently from alkyl bromide, where the second mechanism was not preferred.[10]

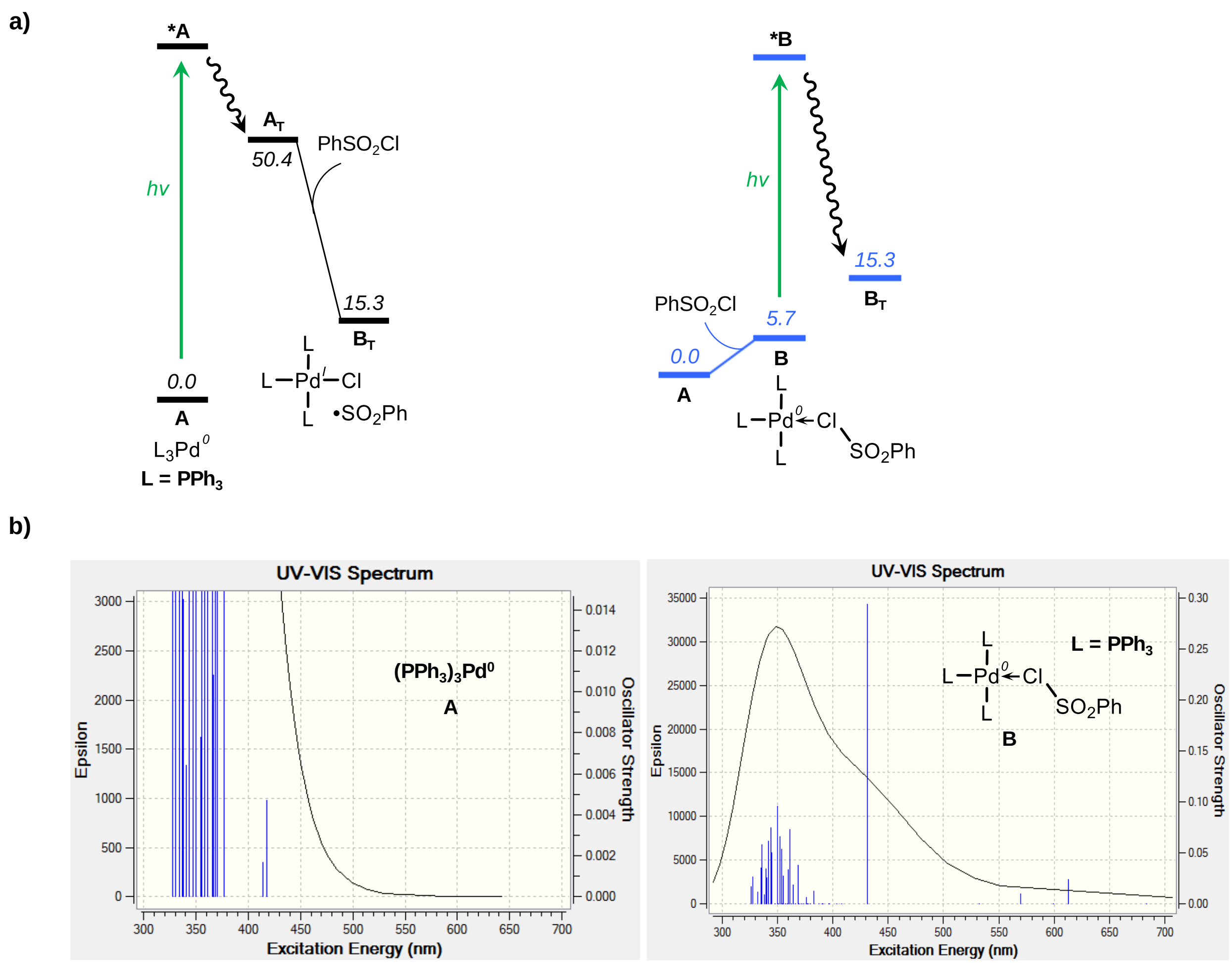

Supplementary Figure 3. a) Two alternative mechanisms of photoabsorption of $L_nPd(0)$ species and b) Computed UV-VIS spectroscopy at M06(SMD)/SDD(Pd)/Def2-TZVP(nonmetals) level are displayed.

Supplementary Table 2. Details of the vertical singlet electron excitation based on the TD-DFT of **A** and **B**.

complex	λ_{max} (nm)	f^a	E[eV]	Character
**A**	418	0.005	2.97	HOMO → LUMO (89%)
**B**	431	0.294	2.88	HOMO-2 → LUMO (90%)

[a]Oscillator Strength.

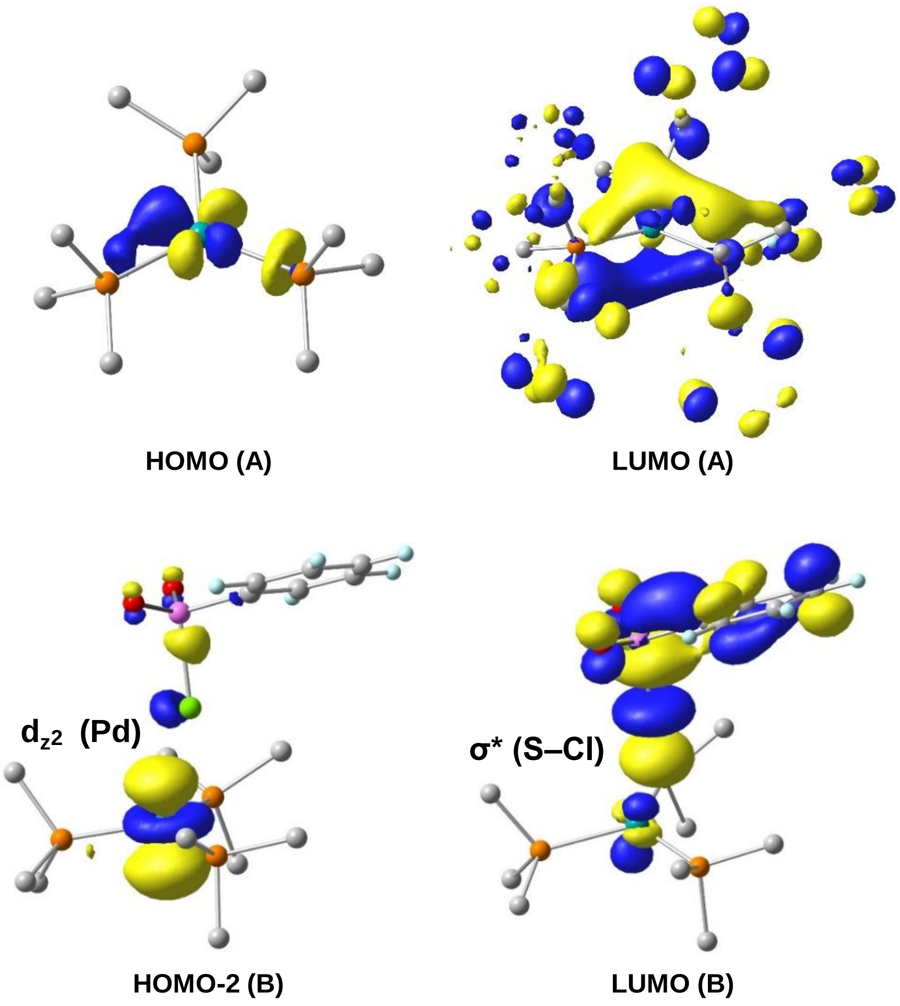

Supplementary Figure 4. Selected KS-MO's of **A** and **B** involve in the vertical excitations which are reported in Supplementary Table 2.

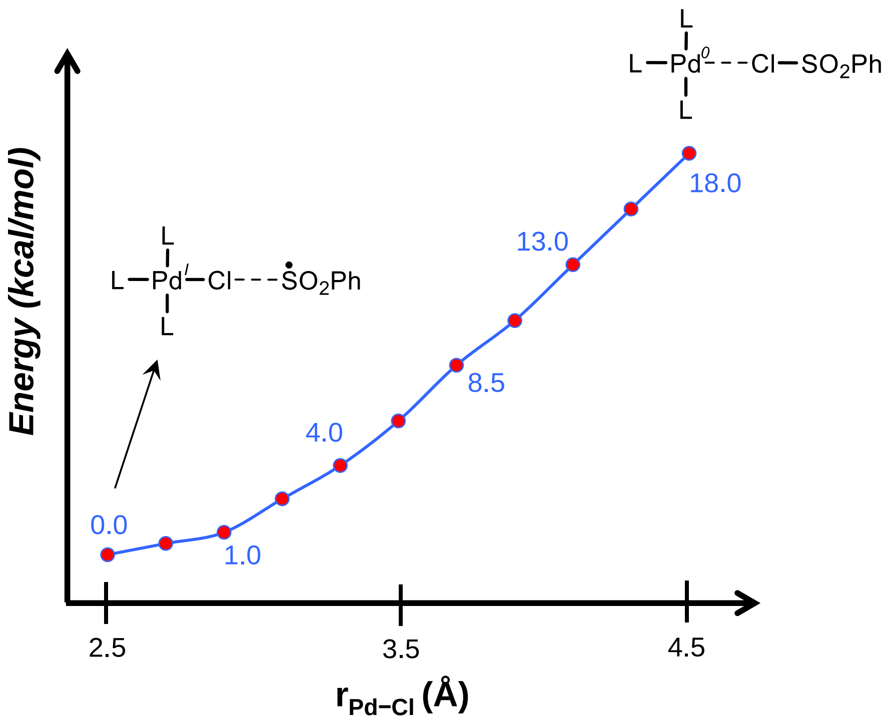

Supplementary Figure 5. Potential energy surface (PES) for the SET between $\mathbf{A_T}$ and $\mathbf{PhSO_2Cl}$ along the Pd-Cl bond coordinates ($r_{Pd\text{-}Cl}$ represents the distance between Pd and Cl atoms).

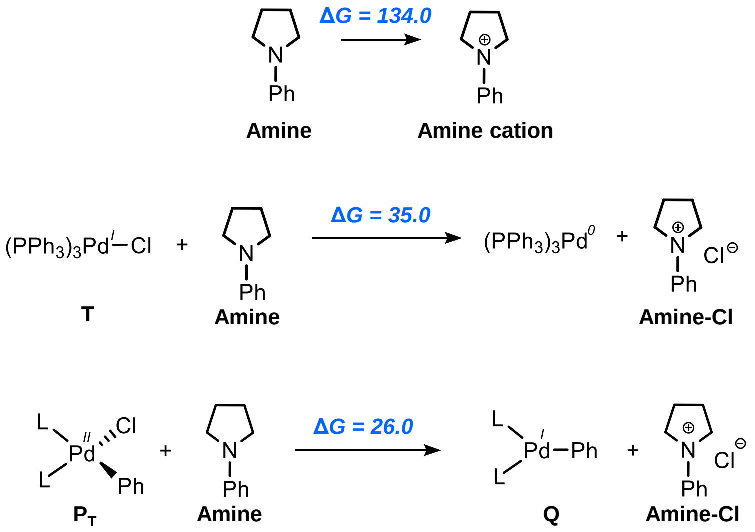

Supplementary Figure 6. Energetics (in kcal/mol) of single electron oxidation of N-phenyl pyrrolidine by Pd(I) and Pd(II) complexes. Calculated energy values indicate that both the Pd(I) and Pd(II) complexes are unable to oxidize the **Amine** because of high endothermicity.

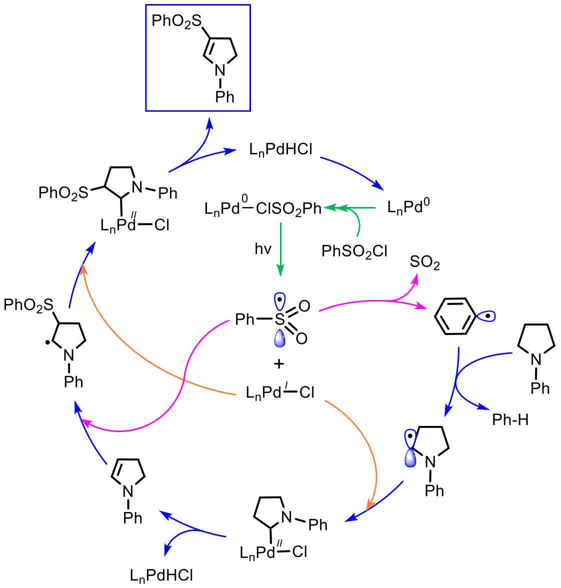

Supplementary Figure 7. Complete catalytic cycle. Catalytic cycle showing various events involved in the dehydrogenative sulfonylation of amines.

We have calculated HAT steps to activate α-amino-C_{sp3}–H bond of amine using different radical species (**D**, **C** and **J**) involved in the proposed catalytic pathway. Calculations indicate that the HAT step involving the phenyl radical, **D**, is more preferred by 10.1 and 15.0 kcal/mol barrier over the other two radicals, **C** and **J**, respectively (Supplementary Fig. 8). Therefore, radical **D** is most active for the HAT step to activate the α-amino-C_{sp3}–H bond.

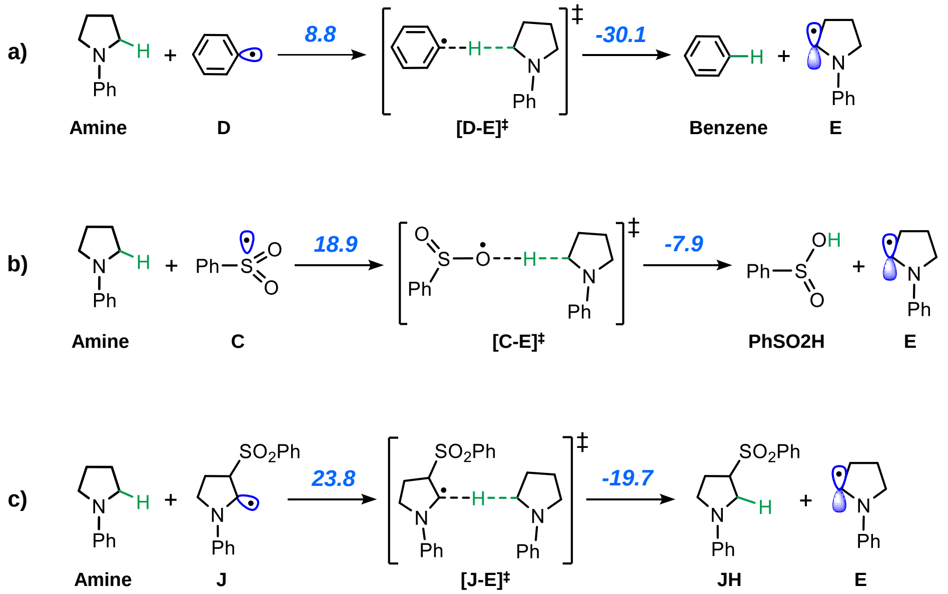

Supplementary Figure 8. Energetics (in kcal/mol) of HAT steps by different radical species to activate α-amino-C_{sp3}–H bond of amine. a) HAT step involving the phenyl radical D. b) HAT step involving the phenyl sulfonyl radical C. c) HAT step involving the α-amino radical J.

Calculations suggest that the *trans* isomer of $(Ph_3P)_2Pd(II)HCl$ is stabilized by 5.2 kcal/mol over cis isomer (**I**$_c$). The liberation of HCl from the cis isomer is thermodynamically feasible and is further accelerated in the presence of the strong base K_2CO_3.

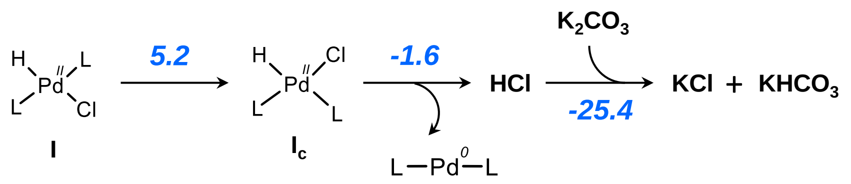

Supplementary Figure 9. Reduction of $(Ph_3P)_2Pd(II)HCl$ to $(Ph_3P)_2Pd(II)$ in presence of base K_2CO_3.

We have considered both the pathways of β-H elimination from intermediate **F** and the PPh_3 dissociated from, **G** (Supplementary Fig. 13). Calculated results indicate that the ligand dissociated pathway is the more preferred one and has a free energy barrier of 21.8 kcal/mol. However, the alternative pathway, having two PPh_3 ligands, requires a barrier of 23.9 kcal/mol and is also viable at the current reaction conditions. Therefore, in the presence of BINAP ligand, the β-H elimination step is expected to be feasible. To support this statement, we have performed further calculations incorporating the BINAP ligand. Consistently, the calculated free energy barrier is 23.5 kcal/mol, which can be achieved at this reaction condition, and thus the step is feasible.

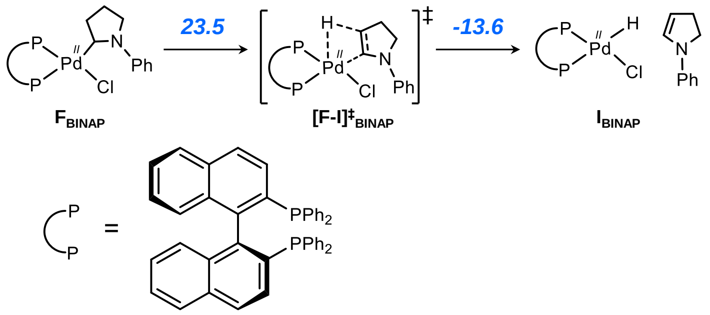

Supplementary Figure 10. Energetics (in kcal/mol) of β-H elimination step in presence of BINAP ligand.

Phenyl Radical Formation:

There are three different pathways for the phenyl radical formation from the **C**, displayed in Supplementary Fig. 11. Two of them are inner sphere (red line and light blue line) and another is outer-sphere mechanisms (blue line). In the inner-sphere mechanisms the **C** binds with **T** to generate **O**$_T$ liberating one PPh_3 via a thermoneutral step. From **O**$_T$ the SO_2 is liberated via triplet transition state **[O-P]**$_T^‡$ leading to **P**$_T$ (red line). This step is endergonic along with requires a free energy barrier of 28.3 kcal/mol. Another inner-sphere mechanism takes place via ground state Pd(II)-complex **O** (light blue line). In this pathway the SO2 liberation occurs via singlet transition state **[O-P]**‡ and free energy barrier of 35.8 kcal/mol. In outer-sphere mechanism the tosyl radical

C is decomposed into phenyl radical and SO_2. This step is endergonic by 14.4 kcal/mol. There is no energy hump is located in the potential energy surface along reaction coordinates of Ph–S bond elongation (Supplementary Fig. 12). The free energy is gradually increasing and it reaches the maximum value of 20.1 kcal/mol at Ph–S distance of ~4Å. The separated form of phenyl radical and SO_2 is 5.7 kcal/mol lower than the top highest energy of the potential surface. Therefore, the highest free energy value, 20.1 kcal/mol is approximated as the energy barrier of tosyl radical decomposition. Comparing all the three competitive pathways the outer sphere mechanism is suggested to be most preferred than the inner sphere mechanism (Supplementary Fig. 12).

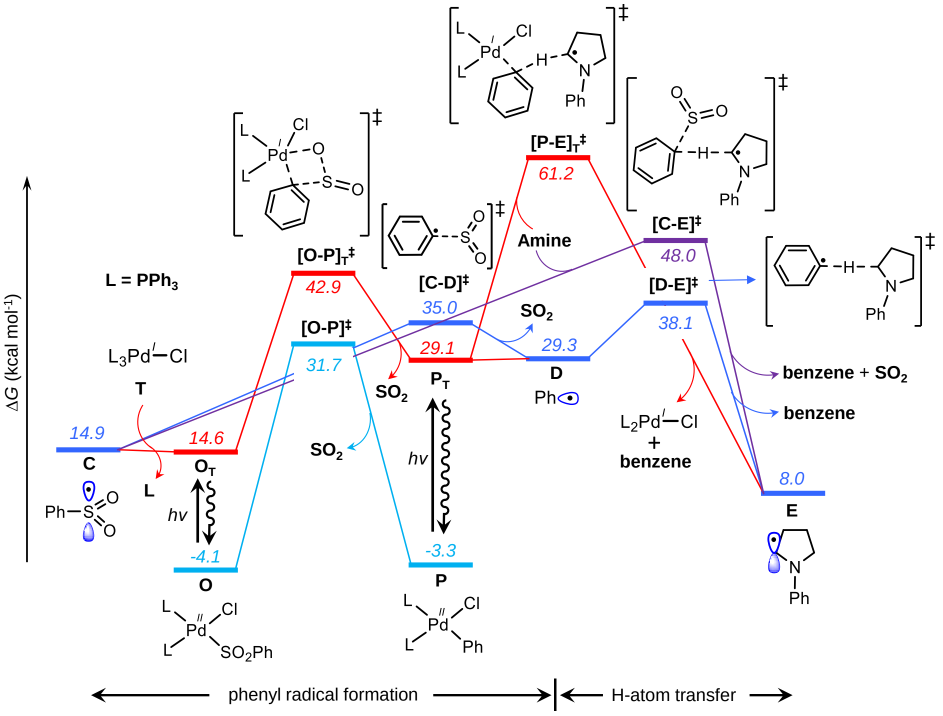

Supplementary Figure 11. Free energy profile for outer sphere (blue line) vs. inner sphere (red line) mechanisms for the HAT step.

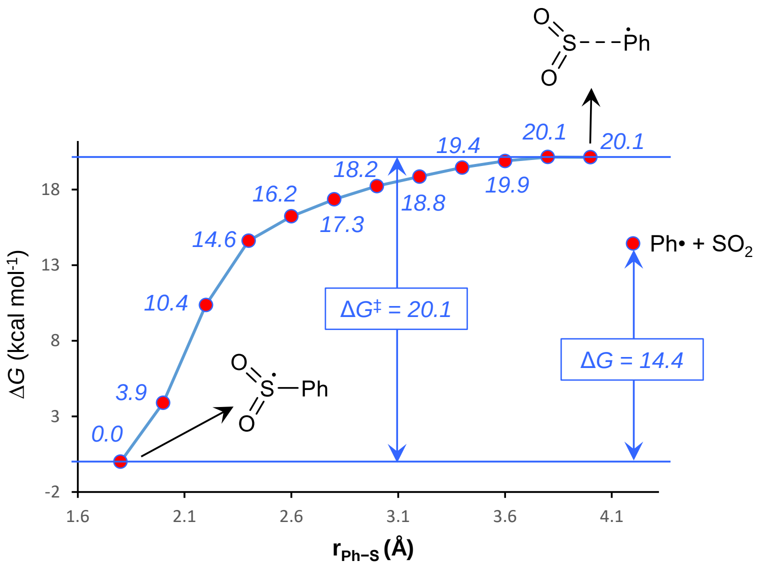

Supplementary Figure 12. Potential energy surface (PES) for the phenyl radical formation from C along the Ph-S bond coordinates (r_{Ph-S} represents the distance between Ph and S atoms).

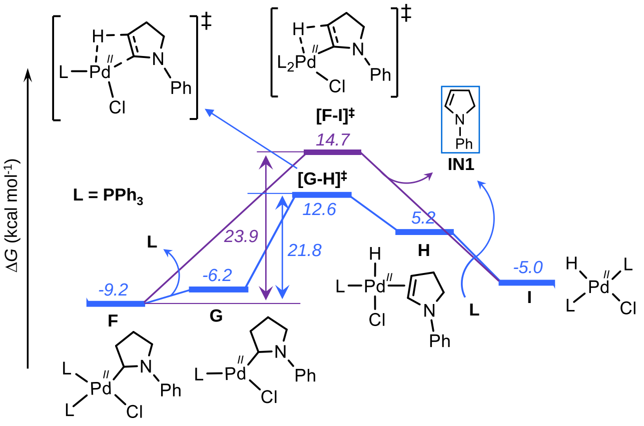

Supplementary Figure 13. Free energy profile for two alternative pathway of β-hydride elimination step from intermediate **F**.

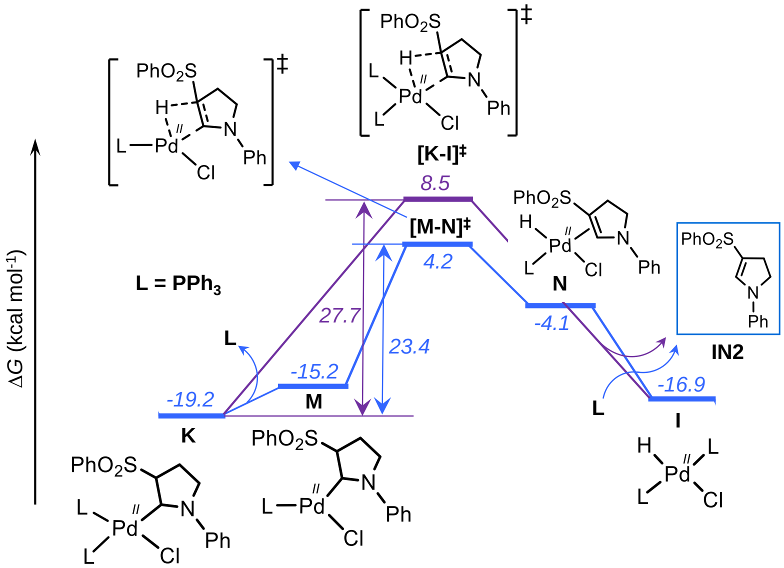

Supplementary Figure 14. Free energy profile for two alternative pathway of β-hydride elimination step from intermediate **K.**

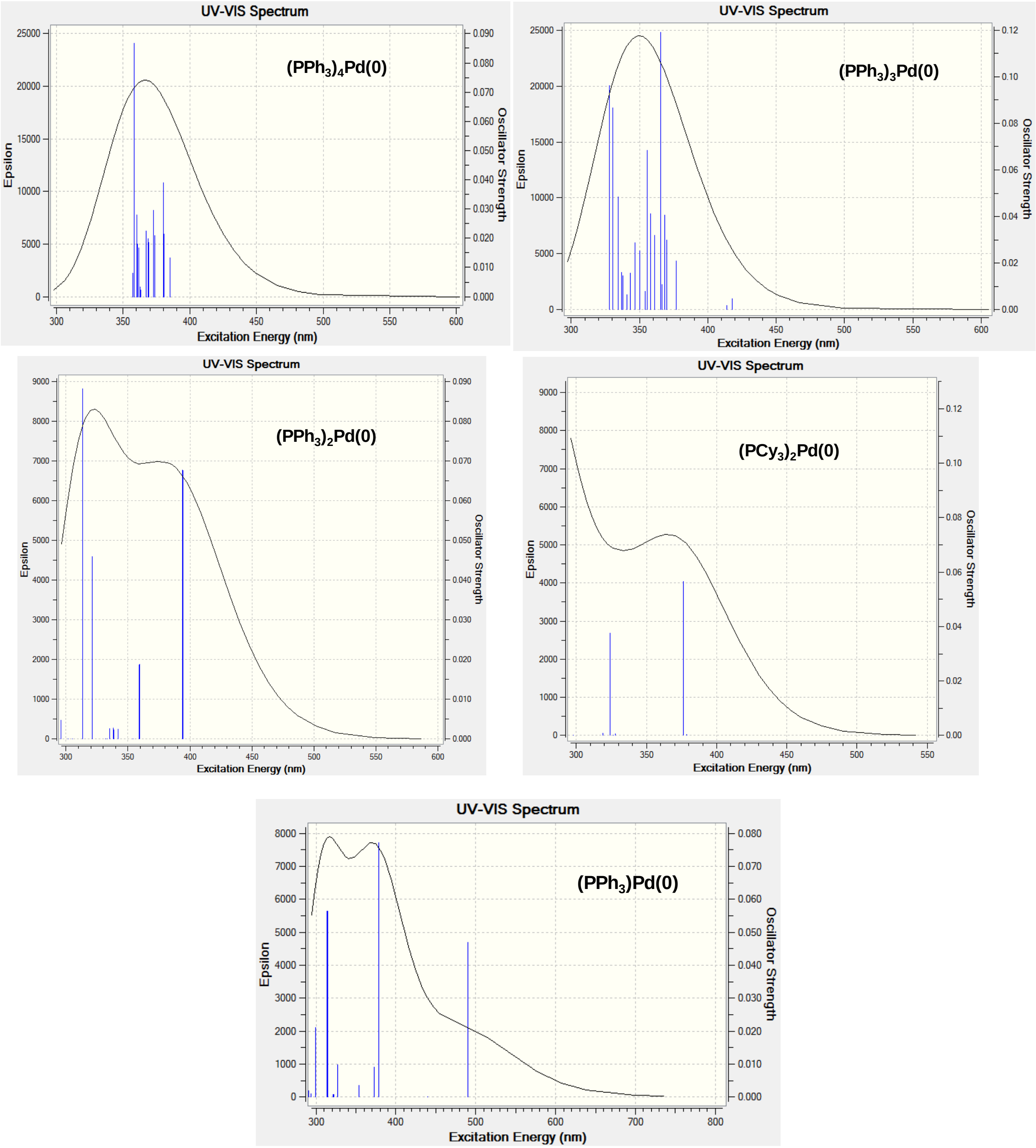

Supplementary Figure 15. Calculated UV-VIS spectra of tetra-ligated, tri-ligated, bi-ligated, mono-ligated palladium.

5. Mechanistic Related Experiments

Radical trapping experiment:

A clean, oven-dried screw cap reaction tube equipped with a Teflon-coated magnetic stir bar was charged with 1-phenylpyrrolidine (29.4 mg, 0.2 mmol, 1 equiv.), tosyl chloride (114.4 mg, 0.6 mmol, 3 equiv.), TEMPO (93.6 mg, 0.6 mmol, 3 equiv.), $Pd(OAc)_2$ (2.3 mg, 0.01 mmol, 5 mol%), PPh_3 (10.5 mg, 0.04 mmol, 20 mol%), and K_2CO_3 (82.8 mg, 0.6 mmol, 3 equiv.). The reaction tube was capped with a rubber septum, evacuated and backfilled with argon (3 times). Then, degassed 1,4-dioxane (0.1 M, 2.0 mL) was added via syringe. The reaction mixture was stirred at room temperature for 48 h under irradiation with 34 W blue LEDs (3 cm away from blue LEDs) with fan cooling. After 48 h, the reaction was quenched via exposure to air. The reaction mixture was diluted with EtOAc and filtered through a small bed of Celite. The GCMS of the crude reaction mixture did not show the formation of product **22**.

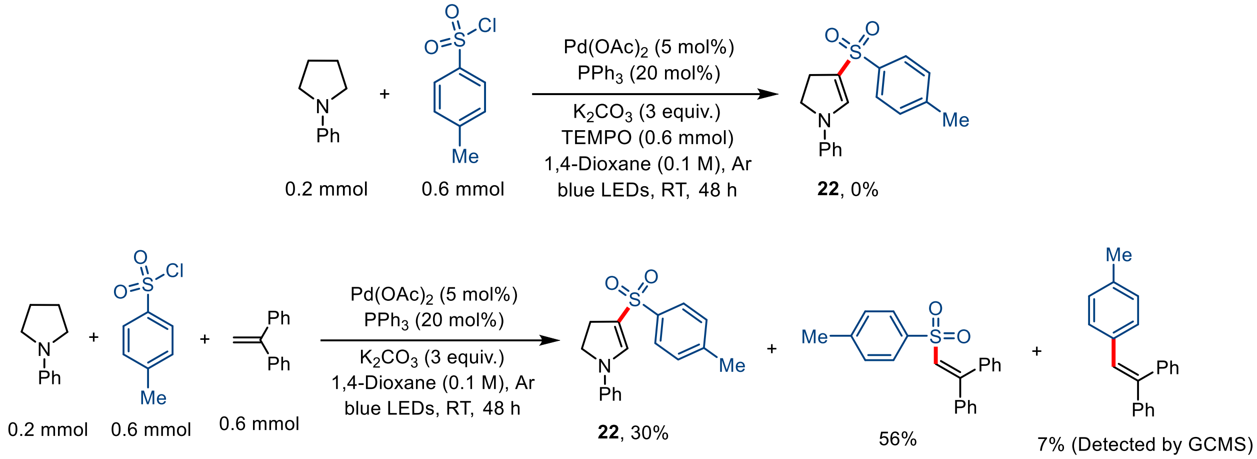

Supplementary Figure 16. Radical trapping experiment using TEMPO and diphenylethylene as the radical quenchers.

A clean, oven-dried screw cap reaction tube equipped with a Teflon-coated magnetic stir bar was charged with 1-phenylpyrrolidine (29.4 mg, 0.2 mmol, 1 equiv.), tosyl chloride (114.4 mg, 0.6 mmol, 3 equiv.), 1,1-diphenylethylene (108.0 mg, 0.6 mmol, 3 equiv.), $Pd(OAc)_2$ (2.3 mg, 0.01 mmol, 5 mol%), PPh_3 (10.5 mg, 0.04 mmol, 20 mol%), and K_2CO_3 (82.8 mg, 0.6 mmol, 3 equiv.). The reaction tube was capped with a rubber septum, evacuated and backfilled with argon (3 times). Then, degassed 1,4-dioxane (0.1 M, 2.0 mL) was added via syringe. The reaction mixture was stirred at room temperature for 48 h under irradiation with 34 W blue LEDs (3 cm away from blue LEDs) with fan cooling. After 48 h, the reaction was quenched via exposure to air. The reaction mixture was diluted with EtOAc and filtered through a small bed of Celite and concentrated in vacuo. The residue was purified by column chromatography using aluminum oxide (~150 mesh size) and *n*-hexane/ethyl acetate as the eluent.

Light on-off experiment:

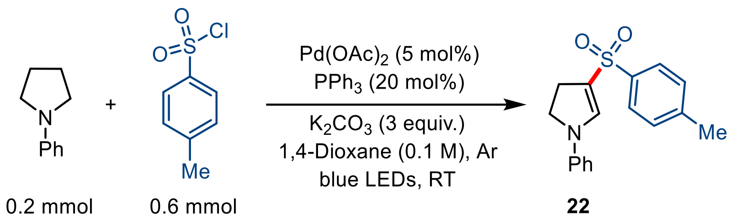

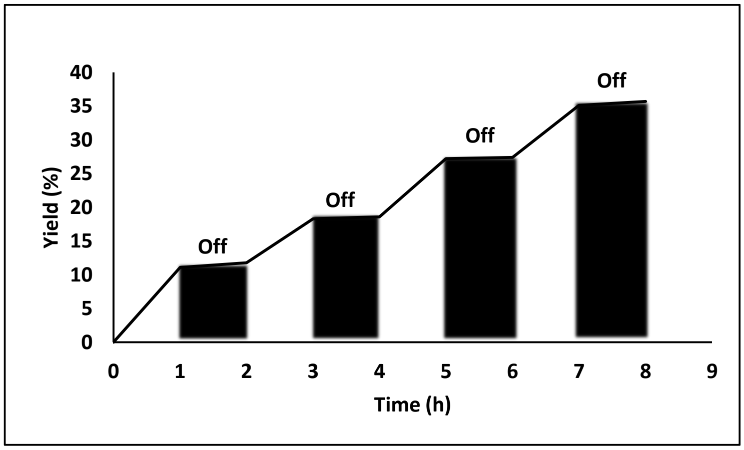

Supplementary Figure 17. Light on-off experiment showing that the radical chain propagation mechanism is not involved in the reaction.

To exclude the possibility of a radical chain process in the reaction, light on-off experiments were carried following the general procedure, with 1-phenylpyrrolidine (29.4 mg, 0.2 mmol, 1 equiv.), tosyl chloride (114.4 mg, 0.6 mmol, 3 equiv.), $Pd(OAc)_2$ (2.3 mg, 0.01 mmol, 5 mol%), PPh_3 (10.5 mg, 0.04 mmol, 20 mol%), and K_2CO_3 (82.8 mg, 0.6 mmol, 3 equiv.). The light was kept off during the off-periods and the yields of the reactions were determined by ^{1}HNMR analysis using trimethoxy benzene as an internal standard. No reaction was observed during the light off-cycles which confirms that the reaction is not proceeding by radical chain propagation mechanism.

Radical-radical cross coupling experiment:

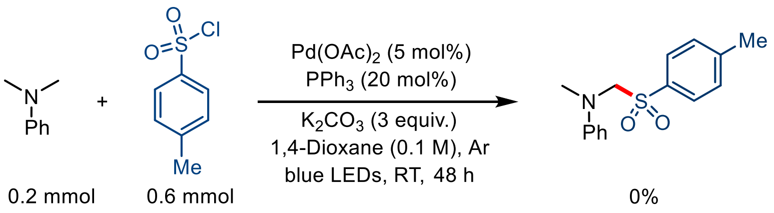

Supplementary Figure 18. Radical-radical cross coupling experiment showing that the reaction does not promote any radical-radical cross-coupling pathway

A clean, oven-dried screw cap reaction tube equipped with a Teflon-coated magnetic stir bar was charged with *N*,*N*-dimethylaniline (24.2 mg, 0.2 mmol, 1 equiv.), tosyl chloride (114.4 mg, 0.6

mmol, 3 equiv.), $Pd(OAc)_2$ (2.3 mg, 0.01 mmol, 5 mol%), PPh_3 (10.5 mg, 0.04 mmol, 20 mol%), and K_2CO_3 (82.8 mg, 0.6 mmol, 3 equiv.). The reaction tube was capped with a rubber septum, evacuated and backfilled with argon (3 times). Then, degassed 1,4-dioxane (0.1 M, 2.0 mL) was added via syringe. The reaction mixture was stirred at room temperature for 48 h under irradiation with 34 W blue LEDs (3 cm away from blue LEDs) with fan cooling. After 48 h, the reaction was quenched via exposure to air. The reaction mixture was diluted with EtOAc and filtered through a small bed of Celite. The GCMS of the crude reaction mixture did not show the formation of the product and confirms that the reaction does not promote any radical-radical cross-coupling pathway.

Experiments to prove arene radical generation:

Experiment-1:

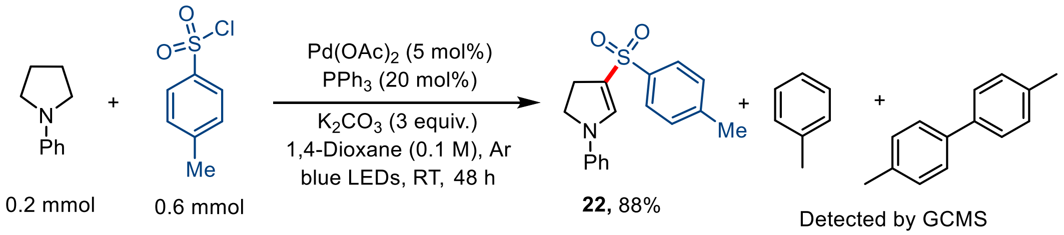

Experiment-2:

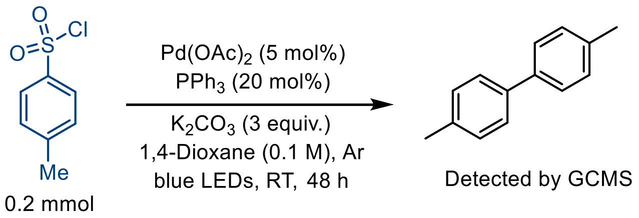

Experiment-3:

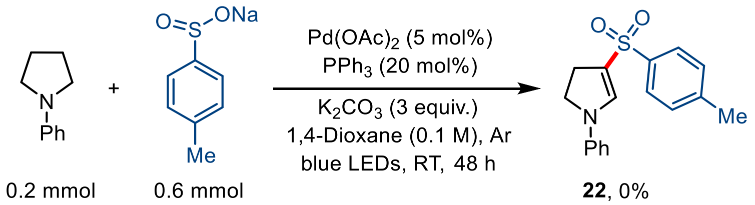

Experiment-4:

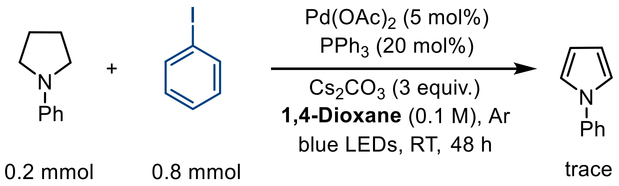

Experiment-5:

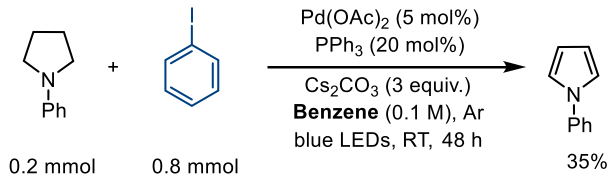

Experiment-6:

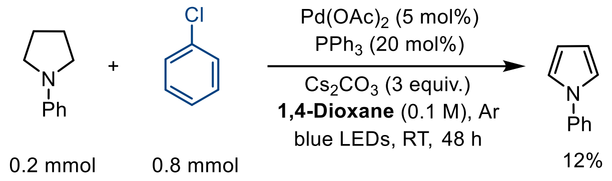

Experiment-7:

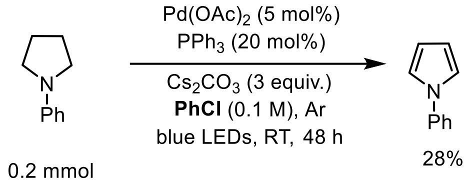

Experiment-8:

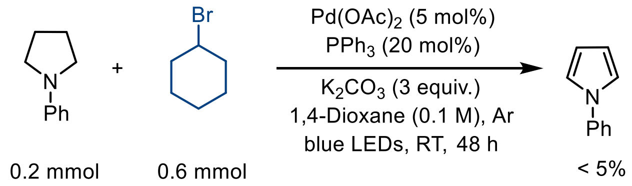

Supplementary Figure 19. Arene radical generation experiments. All above experiments results clearly demonstrates that the arene radicals were generated from aryl halides and tosyl chloride in the presence of Pd(0) complex under photo-irradiation condition. No dehydrogenation product was formed in the absence of light. All yields were calculated by NMR analysis.

Intermediate experiments:

A clean, oven-dried screw cap reaction tube equipped with a Teflon-coated magnetic stir bar was charged with *N*,*N*-diethyl-1-phenylethen-1-amine[11] (35 mg, 0.2 mmol, 1 equiv.), tosyl chloride (76 mg, 0.4 mmol, 2 equiv.), $Pd(OAc)_2$ (2.3 mg, 0.01 mmol, 5 mol%), PPh_3 (10.5 mg, 0.04 mmol, 20 mol%), and K_2CO_3 (55.2 mg, 0.4 mmol, 2 equiv.). The reaction tube was capped with a rubber septum, evacuated and backfilled with argon (3 times). Then, degassed 1,4-dioxane (0.1 M, 2.0 mL) was added via syringe. The reaction mixture was stirred at room temperature for 20 h under irradiation with 34 W blue LEDs (3 cm away from blue LEDs) with fan cooling. After 20 h, the reaction was quenched via exposure to air. The reaction mixture was diluted with EtOAc and

filtered through a small bed of Celite and concentrated in vacuo. The residue was purified by column chromatography using aluminum oxide (~150 mesh size) and *n*-hexane/ethyl acetate as the eluent.

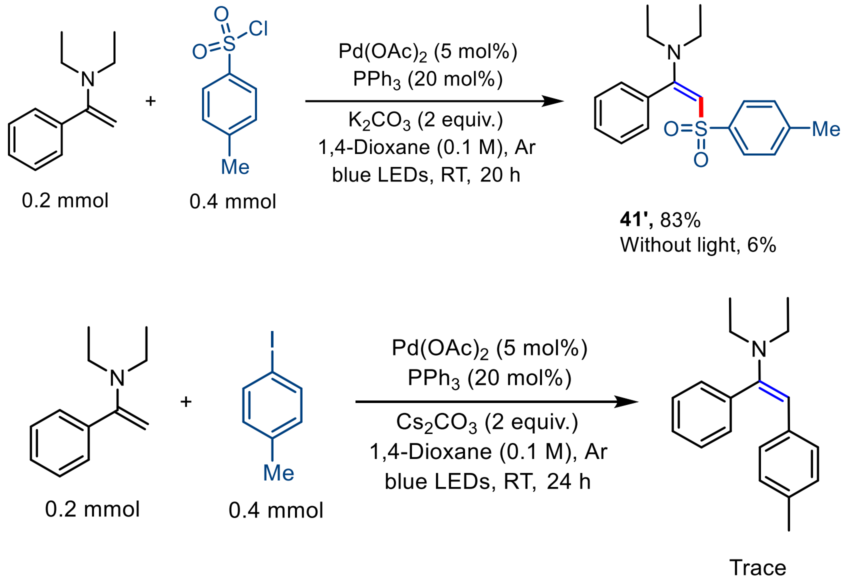

Supplementary Figure 20. Intermediate experiments showing the possible intermediates involved in the reaction.

A clean, oven-dried screw cap reaction tube equipped with a Teflon-coated magnetic stir bar was charged with *N*,*N*-diethyl-1-phenylethen-1-amine (35 mg, 0.2 mmol, 1 equiv.), 1-iodo-4-methylbenzene (87.2 mg, 0.4 mmol, 2 equiv.), $Pd(OAc)_2$ (2.3 mg, 0.01 mmol, 5 mol%), PPh_3 (10.5 mg, 0.04 mmol, 20 mol%), and Cs_2CO_3 (130 mg, 0.4 mmol, 2 equiv.). The reaction tube was capped with a rubber septum, evacuated and backfilled with argon (3 times). Then, degassed 1,4-dioxane (0.1 M, 2.0 mL) was added via syringe. The reaction mixture was stirred at room temperature for 24 h under irradiation with 34 W blue LEDs (3 cm away from blue LEDs) with fan cooling. After 24 h, the reaction was quenched via exposure to air. The product conversion was examined by GCMS analysis.

Competition experiments:

Three separate oven-dried screw cap reaction tubes equipped with a Teflon-coated magnetic stir bar were charged with 1-(*p*-tolyl)pyrrolidine (32.2 mg, 0.2 mmol, 1 equiv.), 4-methoxybenzenesulfonyl chloride (**2c**, 124.0 mg, 0.6 mmol, 3 equiv.), benzenesulfonyl chloride (**2d**, 106.0 mg, 0.6 mmol, 3 equiv.), 4-chlorobenzenesulfonyl chloride (**2f**, 126.6 mg, 0.6 mmol, 3 equiv.), $Pd(OAc)_2$ (2.3 mg, 0.01 mmol, 5 mol%), PPh_3 (10.5 mg, 0.04 mmol, 20 mol%), and K_2CO_3 (82.8 mg, 0.6 mmol, 3 equiv.), respectively. The reaction tubes were capped with a rubber septum, evacuated and backfilled with argon (3 times). Then, degassed 1,4-dioxane (0.1 M, 2.0 mL) was added via syringe. The reaction mixtures were stirred at room temperature for 2 h under irradiation with 34 W blue LEDs (3 cm away from blue LEDs) with fan cooling. After 2 h, the reactions were

quenched via exposure to air. The reaction mixtures were diluted with EtOAc and filtered through a small bed of Celite and concentrated in vacuo. Yields were determined by ^{1}HNMR analysis of the crude mixture relative to trimethoxy benzene as an internal standard.

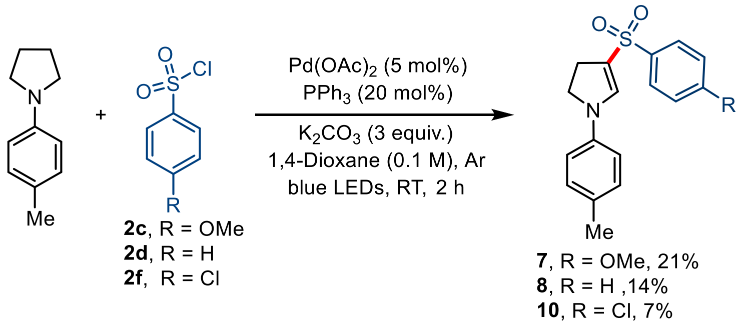

Supplementary Figure 21. Competition experiments showing the effect of various substituents on the reaction.

Intermolecular competition experiment between triethyl amine and d$_{15}$-triethyl amine:

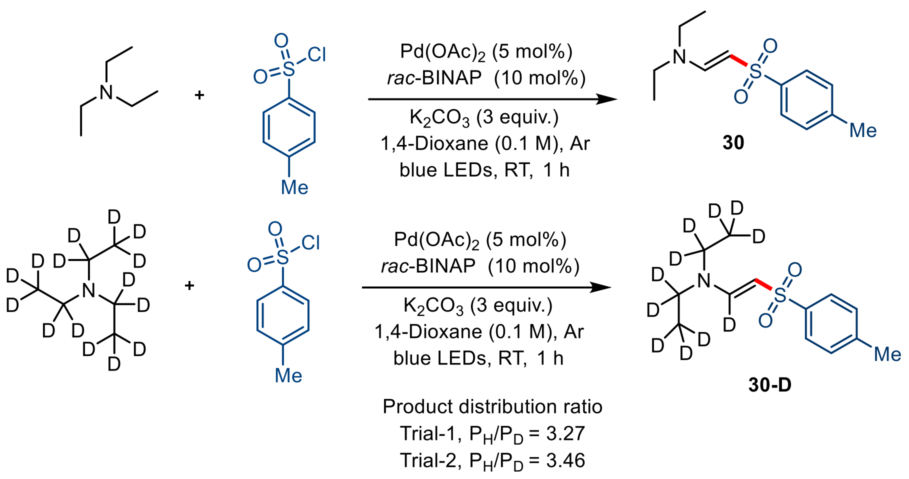

Supplementary Figure 22. Intermolecular competition experiment between triethyl amine and d$_{15}$-triethyl amine.

A separate oven-dried screw cap reaction tubes equipped with a Teflon-coated magnetic stir bar were charged with triethylamine (20.2 mg, 0.20 mmol), tosyl chloride (114.4 mg, 0.4 mmol, 3 equiv.), $Pd(OAc)_2$ (2.3 mg, 0.01 mmol, 5 mol%), *rac*-BINAP (12.4 mg, 0.02 mmol, 10 mol%), and K_2CO_3 (82.8 mg, 0.6 mmol, 3 equiv.). The reaction tubes were capped with a rubber septum, evacuated and backfilled with argon (3 times). Then, degassed 1,4-dioxane (0.1 M, 2.0 mL) was added via syringe. The reaction mixture was stirred at room temperature for 1 h under irradiation with 34 W blue LEDs (3 cm away from blue LEDs) with fan cooling. After 1 h, the reaction was quenched via exposure to air. The conversion was determined by GCMS analysis of the crude mixture relative to dodecane as an internal standard.

A similar procedure was repeated using the d_{15}-triethyl amine and the conversion of product **30-D** was determined by GCMS analysis.

	Time (60 min)	Area of IS	Area of product	Ratio of product/IS	P_H/P_D
Trial-1	TEA	664324.8	69395.15	0.10446	3.276073
	d_{15}-TEA	662992.9	21139.95	0.031886	
Trial- 2	TEA	219272.5	43509.7	0.198428	3.46078
	d_{15}-TEA	230597.2	13221.54	0.057336	

6. Comparison of UV-Vis absorption studies and emission spectrum of blue LEDs:

UV-Vis spectra of the substrates 1-phenylpyrrolidine and tosyl chloride, the catalyst $Pd(PPh_3)_4$ and the mixture of the catalyst with substrates were measured individually with the same concentration used in the reaction mixture using THF as the solvent. The samples were prepared in 2 mL quartz cuvettes, equipped with screw cap PTFE stoppers, and sealed with parafilm inside the argon filled glove-box. The UV-vis spectra of both the catalyst and mixture of catalyst with1-phenylpyrrolidine shows exactly identical, suggesting that no ground state association is happening between the catalyst and 1-phenylpyrrolidine substrate. However, UV-vis spectrum of the mixture of catalyst with tosyl chloride, a red-shift was observed and clearly reveals that ground state association between the two compounds exists in reaction solution without irradiation.

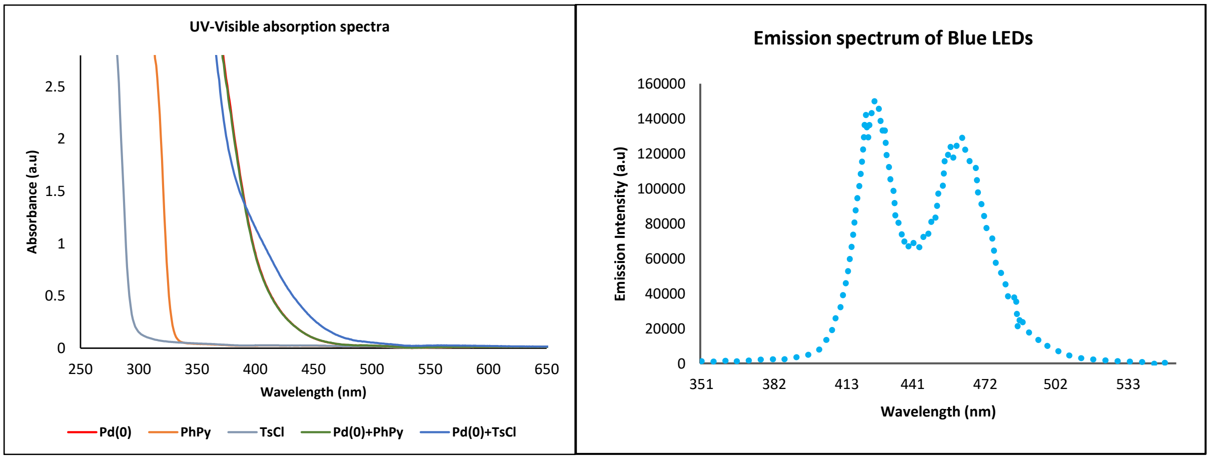

Supplementary Figure 23. a) UV-Vis spectra of 1-phenylpyrrolidine, tosyl chloride, $Pd(PPh_3)_4$ and the mixture of both $Pd(PPh_3)_4$ with 1-phenylpyrrolidine and tosyl chloride in THF (0.1 M) solution. b) Emission spectrum of blue LEDs.

7. Steady-state Stern-Volmer quenching experiments:

Steady-state Stern-Volmer quenching experiments were carried out using a 0.0004 M solution of $Pd(PPh_3)_4$ [**Pd(0)**] and variable concentrations (0.00002, 0.00004, 0.00006, 0.00008, 0.0001, 0.00012, 0.00014 M) of 1-phenylpyrrolidine (**PhPy**) and tosyl chloride (**TsCl**) in THF. The samples were prepared in 2 mL quartz cuvettes, equipped with screw cap PTFE stoppers, and sealed with parafilm inside the argon filled glove-box. The intensity of the emission peak at 630 nm (λ_{ex} = 380 nm) expressed as the ratio I_0/I, where I_0 is the emission intensity of **Pd(0)** at 630 nm in the absence of a quencher and I is the observed intensity, as a function of the quencher different concentration was measured. Fluorescence emission spectra and Stern-Volmer plots for each component are given in below.

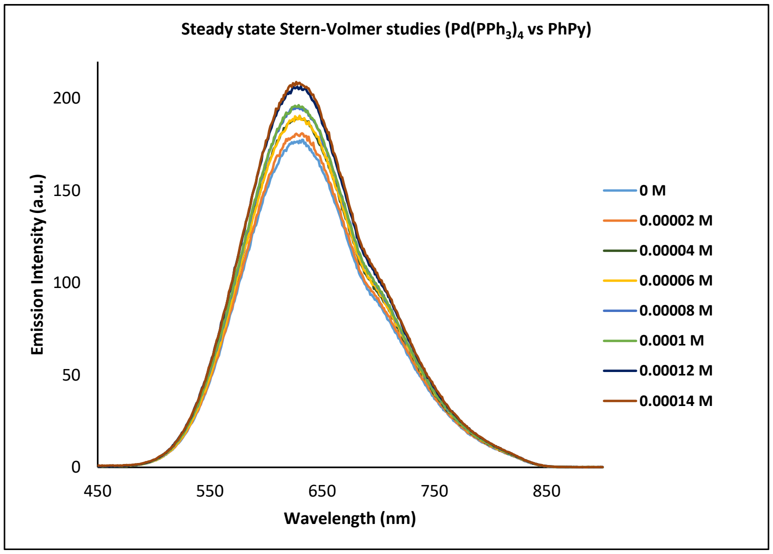

Supplementary Figure 24. Steady-state emission spectra of **Pd(0)** (0.0004 M) with different concentrations of **PhPy**.

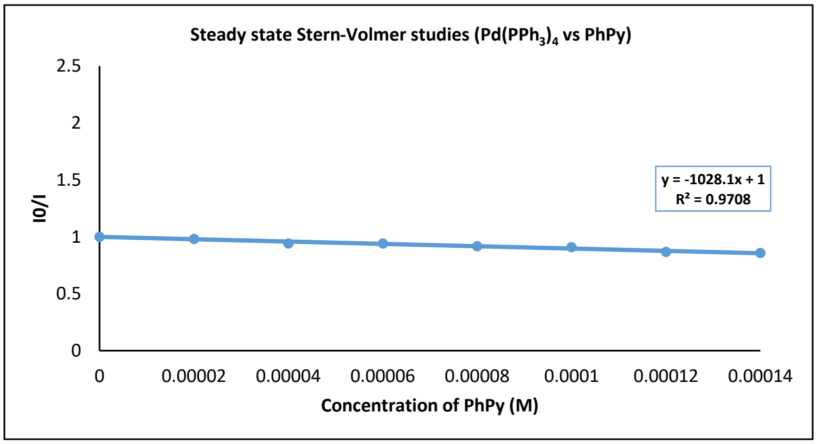

Supplementary Figure 25. Steady-state Stern-Volmer plot of **Pd(0)** (0.0004 M) with different concentrations of **PhPy.**

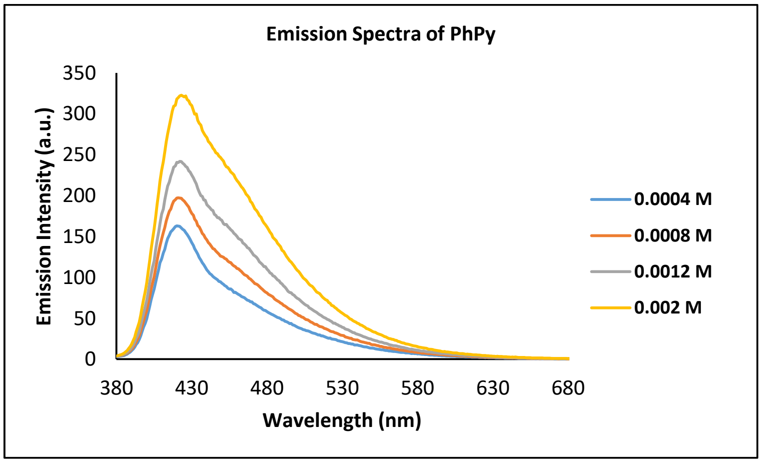

Supplementary Figure 26. Steady-state emission spectra of **PhPy** (λ_{max} = 420 nm) with different concentrations.

Quenching studies [$Pd(PPh_3)_4$ vs PhPy]	λ_{max} (nm)
0 M	633
0.00002 M	632
0.00004 M	630
0.00006 M	630
0.00008 M	628
0.0001 M	628
0.00012 M	627
0.00014 M	626

Supplementary Table 3. Decreasing λ_{max} value of **Pd(0)** with increasing concentration of **PhPy**.

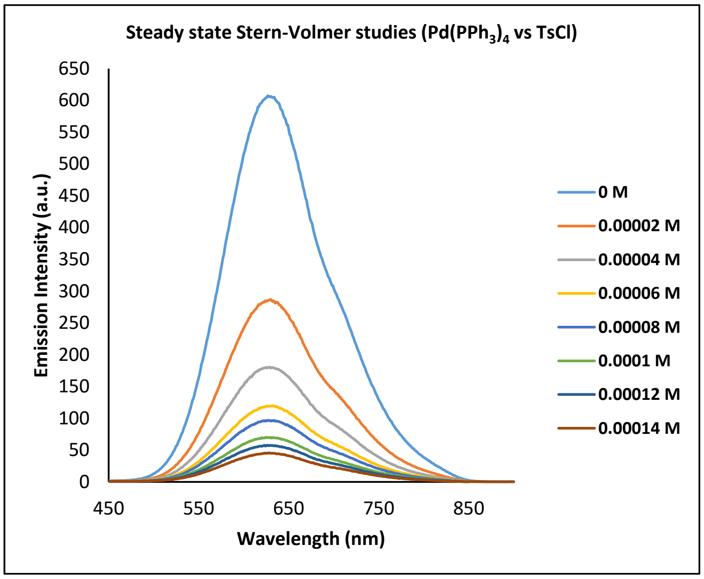

Supplementary Figure 27. Steady-state emission spectra of **Pd(0)** (0.0004 M) with different concentrations of **TsCl**.

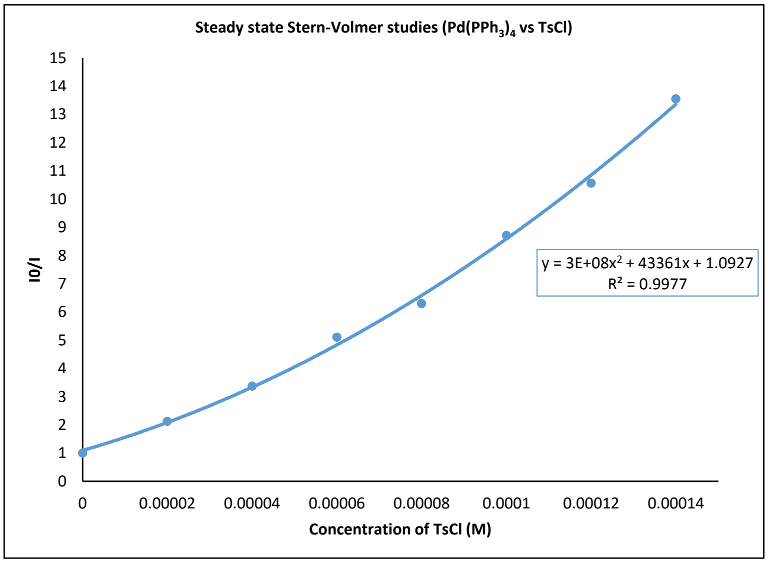

Supplementary Figure 28. Steady-state Stern-Volmer plot of **Pd(0)** (0.0004 M) with different concentrations of **TsCl**.

8. Photoluminescence lifetime (Time resolved Stern-Volmer quenching) experiments:

Time resolved Stern-Volmer quenching experiments were carried out using a 0.0004 M solution of $Pd(PPh_3)_4$ [**Pd(0)**] and variable concentrations (0.00002, 0.00004, 0.00006, 0.00008, 0.0001 M) of tosyl chloride (**TsCl**) in THF. The samples were prepared in 2 mL quartz cuvettes, equipped with screw cap PTFE stoppers, and sealed with parafilm inside the argon filled glove-box. The intensity of the emission peak at 630 nm expressed as the ratio k_{obs}/k_0 [$k = 1/\tau$ (sec^{-1})], where k_0 is the decay of **Pd(0)** at 630 nm in the absence of a quencher and k_{obs} is the observed decay, as a function of the quencher concentration was measured. For lifetime determination, Ar-saturated 0.0004 M solutions in THF were used for determination of the photoluminescence lifetimes of the $Pd(PPh_3)_4$ complex. Photoluminescence decay traces were acquired based on time-correlated single-photon-counting (TCSPC) techniques using a fluoromax-4 spectrophotometer from Horiba Scientific. A 372 nm diode laser was used as the excitation source. The photoluminescence signals were obtained using an automated motorized monochromator. Time resolved emission data were fit to a single exponential decay to extract the observed rate constant (k_{obs}). Phosphorescence emission spectra and Stern-Volmer plots for each component are given in below.

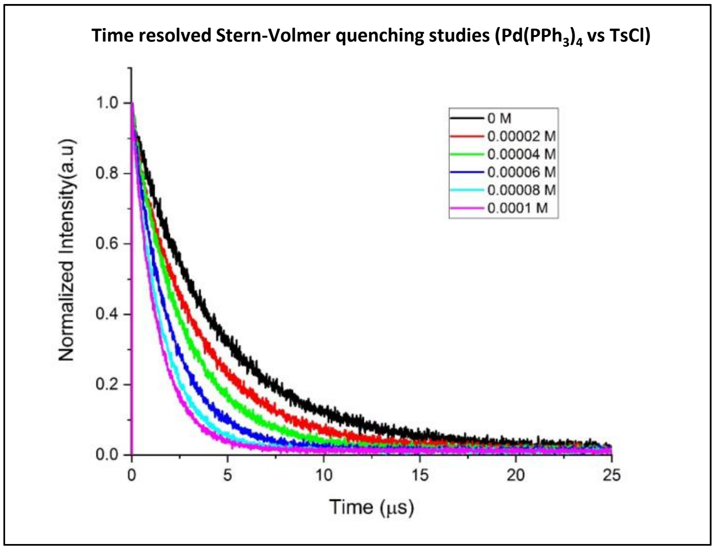

Supplementary Figure 29. Phosphorescence lifetimes of **Pd(0)** (0.0004 M) with different concentrations of **TsCl**. Spectroscopic experiments were performed one single time.

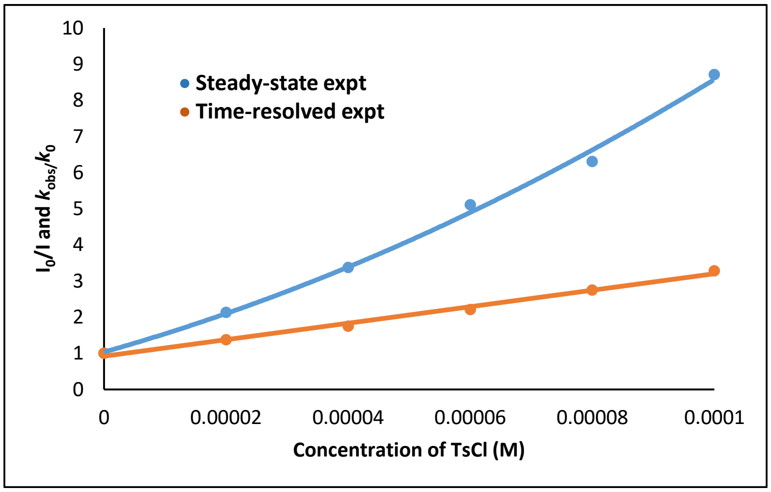

Supplementary Figure 30. Combined steady state and time resolved Stern-Volmer quenching plot of **Pd(0)** (0.0004 M) with different concentrations of **TsCl**.

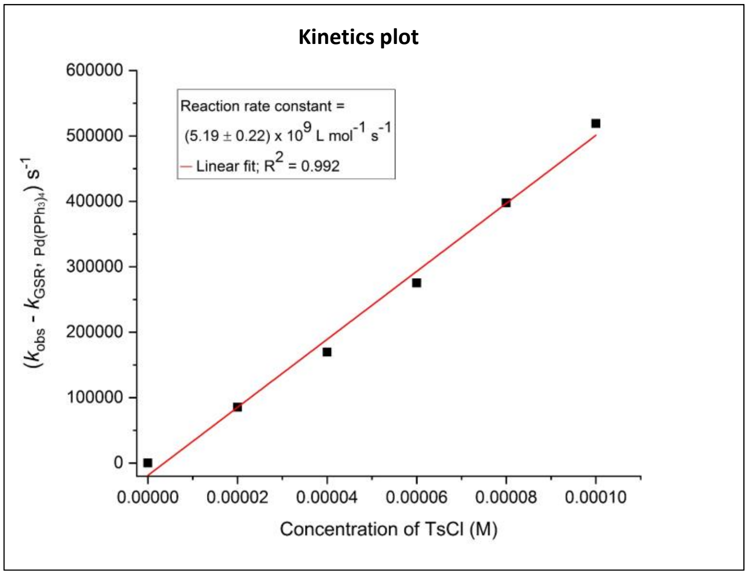

Supplementary Figure 31. Plot of the observed electron transfer rate constant of ***Pd(0)** (0.0004 M) deactivation corrected by the intrinsic GSR rate of **Pd(0)** *vs.* different concentration of **TsCl**. Data were collected by the use of phosphorescence lifetime measurements.

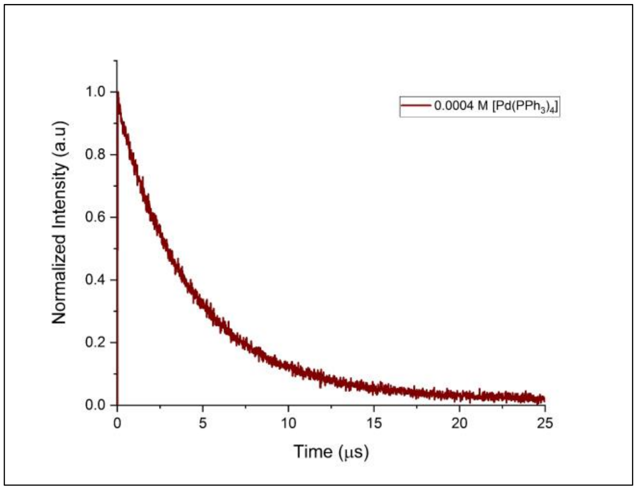

Supplementary Figure 32. Lifetime measurement of $Pd(PPh_3)_4$ at 0.0004 M in THF solvent. The observed excited-state lifetime of $*Pd(PPh_3)_4$ is 4.4 ±0.017 µs using its maximum emission at 630 nm.

9. Time-resolved absorption experiments:

Time-resolved absorption experiments were carried out using a 0.0004 M solution of $Pd(PPh_3)_4$ **[Pd(0)]** and variable concentrations (0.00004, 0.00008, 0.00012, 0.00016 M) of tosyl chloride (**TsCl**) in THF. The samples were prepared in 1 mm cuvettes, equipped with screw cap PTFE stoppers, sealed with parafilm inside the argon-filled glove-box, and excited at 355 nm. Time-resolved absorption spectra for each component are given in below.

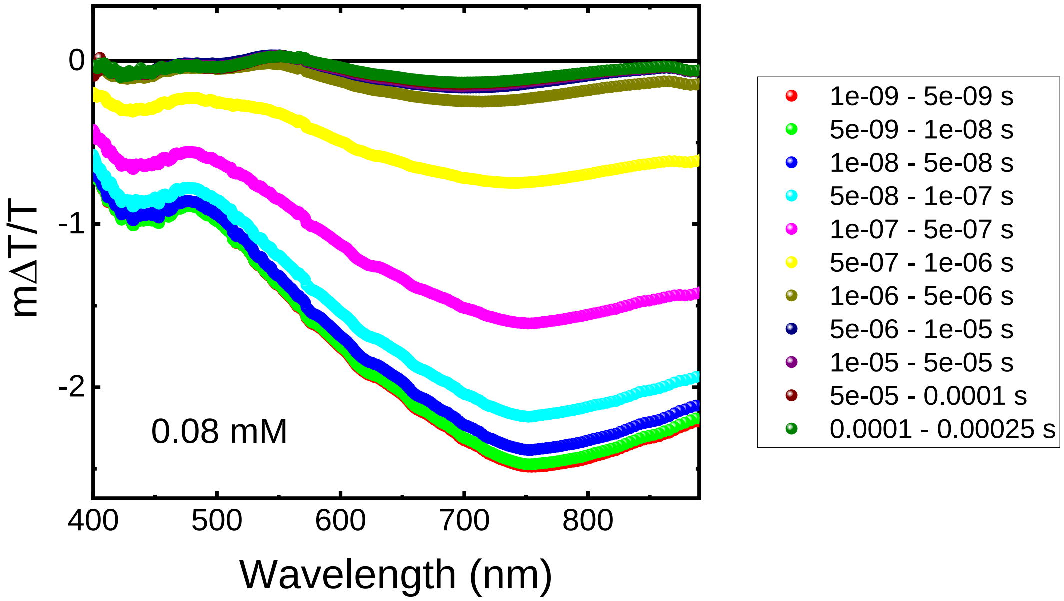

Supplementary Figure 33. Nanosecond-microsecond TA spectra of **Pd(0)** + 0.08 mM **TsCl**.

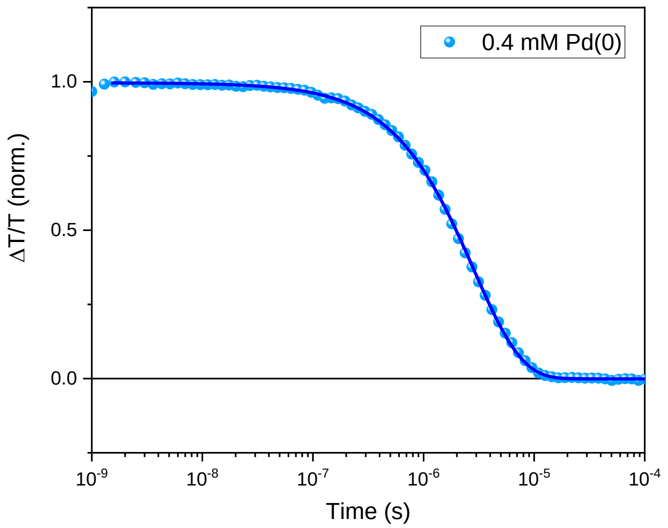

Supplementary Figure 34. ns-TA kinetics (closed symbols) of $Pd(PPh_3)_4$ (0.4 mM) in the absence of quencher **TsCl** and corresponding mono-exponential fit (blue line). The exponential fit yield an inverse rate constant of 2.85± 0.008 µs.

10. Applications

A solution of **4** (50 mg, 0.16 mmol) in trifluoroacetic acid (1 mL) was stirred at 60 °C for 10 min. A solution of triethylsilane (51 μL, 0.32 mmol) in trifluoroacetic acid (1 mL) was then added dropwise, and the reaction stirred at 60 °C for 60 h. The reaction was cooled to RT, and concentrated in vacuo, and the crude residue was basified with aqueous 2M NaOH (5 mL), diluted with water (5 mL) and extracted with DCM (3 x 10 mL). The organic layer was collected, dried ($MgSO_4$), filtered, and concentrated in vacuo. The product was purified by aluminum oxide column chromatography using EtOAc/*n*-hexane as the eluent, to afford **46** (42.8 mg, 85%).

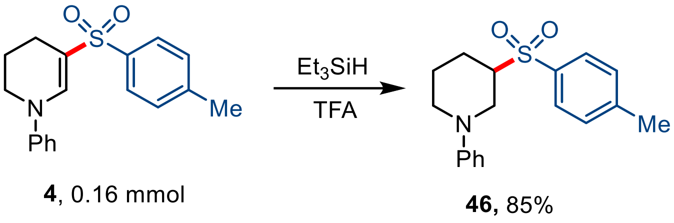

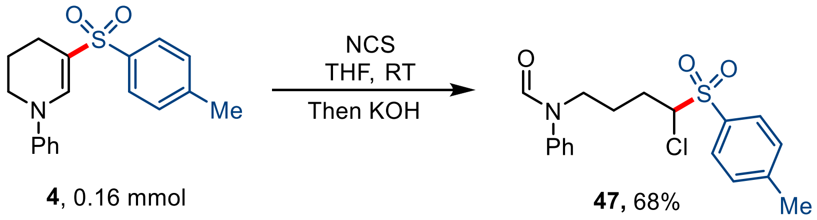

Supplementary Figure 35. Transformations of obtained aminovinyl sulfones.

A solution of **4** (50 mg, 0.16 mmol) and *N*-chlorosuccinimide (32 mg, 0.24 mmol) in THF (2 mL) was stirred at RT for 2 h. After 2 h, aqueous KOH (1 M, 0.32 mL, 0.32 mmol) was added and then the reaction stirred at RT for 24 h. After reaction completed the reaction mixture was diluted with water (5 mL) and extracted with DCM (3 x 10 mL). The organic layer was collected, dried ($MgSO_4$), filtered, and concentrated in vacuo. The product was purified by aluminum oxide column chromatography using EtOAc/*n*-hexane as the eluent, to afford **47** (39.8 mg, 68%).

11. Characterization Data:

1-Phenyl-5-tosyl-1,2,3,4-tetrahydropyridine (4)

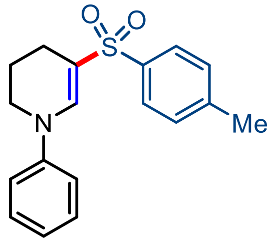

The title compound was synthesized according to the general procedure employing $Pd(OAc)_2$ (2.3 mg, 0.01 mmol), PPh_3 (10.5 mg, 0.04 mmol), K_2CO_3 (82.8 mg, 0.60 mmol), 1-phenylpiperidine (32.2 mg, 0.20 mmol), tosyl chloride (114.4 mg, 0.60 mmol), and 1,4-dioxane (0.1 M, 2 mL) for 48 h. The product was purified by column chromatography (aluminum oxide, gradient 15 to 18% EA/*n*-hexane). Yield = 52 mg (83%).

^1H NMR (400 MHz, DMSO-d_6): δ 7.75 – 7.67 (m, 3H), 7.44 – 7.34 (m, 4H), 7.20 (d, J = 7.9 Hz, 2H), 7.09 (t, J = 7.3 Hz, 1H), 3.60 – 3.49 (m, 2H), 2.38 (s, 3H), 2.13 (t, J = 6.1 Hz, 2H), 1.85 (p, J = 6.0 Hz, 2H); ^{13}C NMR (101 MHz, DMSO-d_6): δ 144.97, 142.68, 138.92, 138.55, 129.69, 129.48, 126.77, 123.07, 117.47, 107.49, 45.22, 20.98, 20.61, 19.65; GCMS (EI) *m/z* calc. for $C_{18}H_{19}NO_2S$ [M^+] 313.1, found 313.1, 296.0, 248.1, 220.1, 174.0, 156.1, 143.0, 129.0, 117.0, 104.0, 91.0; HRMS (ESI-TOF) m/z: $(M+Na)^+$ calcd. for $C_{18}H_{19}NO_2SNa$, 336.1029; found 336.1046; FT-IR ν_{max}(ATR) cm^{-1}: 2942, 1618, 1594, 1494, 1450, 1337, 1284, 1270, 1179, 1099, 1079, 1029, 763, 706, 666.

1-(*p*-Tolyl)-4-tosyl-2,3-dihydro-1*H*-pyrrole (5)

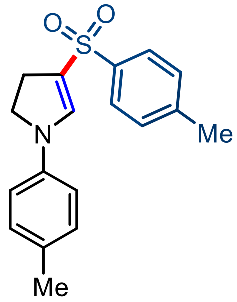

The title compound was synthesized according to the general procedure employing $Pd(OAc)_2$ (2.3 mg, 0.01 mmol), PPh_3 (10.5 mg, 0.04 mmol), K_2CO_3 (82.8 mg, 0.60 mmol), 1-(*p*-tolyl)pyrrolidine (32.2 mg, 0.20 mmol), tosyl chloride (114.4 mg, 0.60 mmol), and 1,4-dioxane (0.1 M, 2 mL) for 48 h. The product was purified by column chromatography (aluminum oxide, gradient 16 to 18% EA/*n*-hexane). Yield = 55.2 mg (88%).

^1H NMR (400 MHz, DMSO-d_6): δ 7.93 (s, 1H), 7.73 (d, J = 8.2 Hz, 2H), 7.40 (d, J = 8.1 Hz, 2H), 7.09 (d, J = 8.4 Hz, 2H), 6.99 (d, J = 8.5 Hz, 2H), 3.95 (t, J = 10.1 Hz, 2H), 2.71 (t, J = 10.1 Hz, 2H), 2.38 (s, 3H), 2.22 (s, 3H); ^{13}C NMR (101 MHz, DMSO-d_6): δ 142.85, 142.63, 140.90, 139.11, 129.77, 129.30, 126.47, 121.08, 114.25, 112.30, 49.73, 26.62, 20.98; GCMS (EI) *m/z* calc. for $C_{18}H_{19}NO_2S$ [M^+] 313.1, found 313.1, 248.2, 174.1, 157.1, 143.1, 118.1, 91.1, 65.1, 39.1; HRMS (ESI-TOF) m/z: $(M+Na)^+$ calcd. for $C_{18}H_{19}NO_2SNa$, 336.1029; found, 336.1042; FT-IR ν_{max}(ATR) cm^{-1}: 2918, 1588, 1572, 1520, 1405, 1337, 1282, 1183, 1140, 1109, 1074, 993, 871, 847, 822, 662.

4-((4-(*tert*-Butyl)phenyl)sulfonyl)-1-(*p*-tolyl)-2,3-dihydro-1*H*-pyrrole (6)

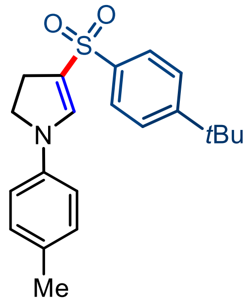

The title compound was synthesized according to the general procedure employing $Pd(OAc)_2$ (2.3 mg, 0.01 mmol), PPh_3 (10.5 mg, 0.04 mmol), K_2CO_3 (82.8 mg, 0.60 mmol), 1-(*p*-tolyl)pyrrolidine (32.2 mg, 0.20 mmol), 4-(*tert*-butyl)benzenesulfonyl chloride (139.6 mg, 0.60 mmol), and 1,4-dioxane (0.1 M, 2 mL) for 40 h. The product was purified by column chromatography (aluminum oxide, gradient 16 to 18% EA/*n*-hexane). Yield = 61.8 mg (87%).

^{1}H NMR (400 MHz, DMSO-d_6): δ 7.95 (s, 1H), 7.77 (d, J = 8.4 Hz, 2H), 7.61 (d, J = 8.4 Hz, 2H), 7.09 (d, J = 8.3 Hz, 2H), 6.99 (d, J = 8.4 Hz, 2H), 3.95 (t, J = 10.1 Hz, 2H), 2.73 (t, J = 10.0 Hz, 2H), 2.22 (s, 3H), 1.29 (s, 9H); ^{13}C NMR (101 MHz, DMSO-d_6): δ 155.39, 143.00, 139.34, 138.59, 130.08, 129.73, 126.23, 126.14, 114.28, 111.33, 49.84, 34.83, 30.80, 26.66, 20.13; GCMS (EI) *m/z* calc. for $C_{21}H_{25}NO_2S$ [M^+] 355.2, found 355.1, 276.1, 174.1, 157.1, 143.1, 118.1, 91.1, 65.1, 41.1; HRMS (ESI-TOF) m/z: $(M+Na)^+$ calcd. for $C_{21}H_{25}NO_2SNa$, 378.1498; found, 378.1515; FT-IR ν_{max}(ATR) cm^{-1}: 2918, 1570, 1503, 1461, 1403, 1331, 1285, 1237, 1175, 1143, 1102, 988, 831, 706.

4-((4-Methoxyphenyl)sulfonyl)-1-(*p*-tolyl)-2,3-dihydro-1*H*-pyrrole (7)

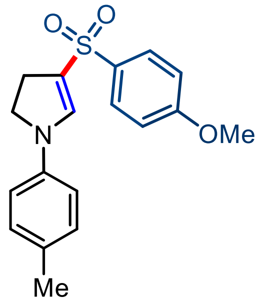

The title compound was synthesized according to the general procedure employing $Pd(OAc)_2$ (2.3 mg, 0.01 mmol), PPh_3 (10.5 mg, 0.04 mmol), K_2CO_3 (82.8 mg, 0.60 mmol), 1-(*p*-tolyl)pyrrolidine (32.2 mg, 0.20 mmol), 4-methoxybenzenesulfonyl chloride (123.9 mg, 0.60 mmol), and 1,4-dioxane (0.1 M, 2 mL) for 30 h. The product was purified by column chromatography (aluminum oxide, gradient 18 to 20% EA/*n*-hexane). Yield = 58.6 mg (89%).

^{1}H NMR (400 MHz, DMSO-d_6): δ 7.91 (s, 1H), 7.77 (d, J = 8.8 Hz, 2H), 7.10 (t, J = 8.3 Hz, 4H), 6.99 (d, J = 8.5 Hz, 2H), 3.95 (t, J = 10.1 Hz, 2H), 3.83 (s, 3H), 2.71 (t, J = 10.0 Hz, 2H), 2.23 (s, 3H); ^{13}C NMR (101 MHz, DMSO-d_6): δ 162.24, 142.39, 138.69, 133.76, 129.94, 129.73, 128.60, 114.46, 114.21, 111.97, 55.65, 49.80, 26.65, 20.12; GCMS (EI) *m/z* calc. for $C_{18}H_{19}NO_3S$ [M^+] 329.1, found 329.1, 264.1, 250.1, 174.1, 157.1, 132.6, 118.1, 91.1, 65.1; HRMS (ESI-TOF) m/z: $(M+Na)^+$ calcd. for $C_{18}H_{19}NO_3SNa$, 352.0978; found, 352.0988; FT-IR ν_{max}(ATR) cm^{-1}: 2919, 1590, 1573, 1517, 1495, 1313, 1290, 1255, 1137, 1105, 1086, 1022, 990, 800, 698.

4-(Phenylsulfonyl)-1-(*p*-tolyl)-2,3-dihydro-1*H*-pyrrole (8)

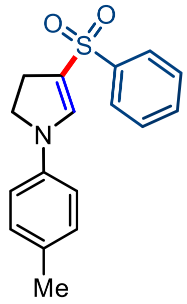

The title compound was synthesized according to the general procedure employing $Pd(OAc)_2$ (2.3 mg, 0.01 mmol), PPh_3 (10.5 mg, 0.04 mmol), K_2CO_3 (82.8 mg, 0.60 mmol), 1-(*p*-tolyl)pyrrolidine (32.2 mg, 0.20 mmol), benzenesulfonyl chloride (105.9 mg, 0.60 mmol), and 1,4-dioxane (0.1 M, 2 mL) for 48 h. The product was purified by column chromatography (aluminum oxide, gradient 15 to 17% EA/*n*-hexane). Yield = 49.7 mg (83%).

^1H NMR (400 MHz, DMSO-d_6): δ 7.98 (s, 1H), 7.86 (d, J = 7.0 Hz, 2H), 7.73 – 7.47 (m, 3H), 7.09 (d, J = 8.2 Hz, 2H), 7.01 (d, J = 8.3 Hz, 2H), 3.96 (t, J = 10.2 Hz, 2H), 2.73 (t, J = 10.1 Hz, 2H), 2.22 (s, 3H); ^{13}C NMR (101 MHz, DMSO-d_6): δ 143.39, 142.11, 138.54, 132.44, 130.19, 129.73, 129.32, 126.31, 114.35, 110.88, 49.90, 26.61, 20.13; GCMS (EI) *m/z* calc. for $C_{17}H_{17}NO_2S$ [M^+] 299.1, found 299.1, 282.1, 234.1, 220.1, 190.0, 160.1, 143.1, 116.1, 104.1, 77.0; HRMS (ESI-TOF) m/z: $(M+Na)^+$ calcd. for $C_{17}H_{17}NO_2SNa$, 322.0872; found, 322.0891; FT-IR ν_{max}(ATR) cm^{-1}: 2917, 1613, 1591, 1513, 1477, 1445, 1301, 1257, 1223, 1141, 1111, 1084, 989, 802, 773.

4-((4-Fluorophenyl)sulfonyl)-1-(*p*-tolyl)-2,3-dihydro-1*H*-pyrrole (9)

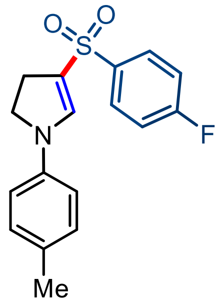

The title compound was synthesized according to the general procedure employing $Pd(OAc)_2$ (2.3 mg, 0.01 mmol), PPh_3 (10.5 mg, 0.04 mmol), K_2CO_3 (82.8 mg, 0.60 mmol), 1-(*p*-tolyl)pyrrolidine (32.2 mg, 0.20 mmol), 4-fluorobenzenesulfonyl chloride (116.8 mg, 0.60 mmol), and 1,4-dioxane (0.1 M, 2 mL) for 48 h. The product was purified by column chromatography (aluminum oxide, gradient 15 to 18% EA/*n*-hexane). Yield = 51.4 mg (81%).

^1H NMR (400 MHz, DMSO-d_6): δ 7.99 (s, 1H), 7.92 (dd, J = 8.7, 5.3 Hz, 2H), 7.43 (t, J = 8.8 Hz, 2H), 7.10 (d, J = 8.4 Hz, 2H), 7.01 (d, J = 8.5 Hz, 2H), 4.13 – 3.81 (m, 2H), 2.74 (t, J = 10.1 Hz, 2H), 2.23 (s, 3H); ^{13}C NMR (101 MHz, DMSO-d_6): δ 164.15 (d, $J_{C\text{-}F}$ = 249.0 Hz), 143.60, 138.58 (d, $J_{C\text{-}F}$ = 3.0 Hz), 138.50, 130.27, 129.73, 129.41 (d, $J_{C\text{-}F}$ = 10.0 Hz), 116.44 (d, $J_{C\text{-}F}$ = 22.0 Hz), 114.39, 110.60, 49.93, 26.53, 20.14; ^{19}F NMR (377 MHz, DMSO-d_6) δ -106.98; GCMS (EI) *m/z* calc. for $C_{17}H_{16}FNO_2S$ [M^+] 317.1, found 317.1, 252.1, 174.1, 157.1, 143.0, 118.1, 91.1, 65.1, 51.1; HRMS (ESI-TOF) m/z: $(M+Na)^+$ calcd. for $C_{17}H_{16}FNO_2SNa$, 340.0778; found, 340.0805; FT-IR ν_{max}(ATR) cm^{-1}: 2920, 1586, 1574, 1519, 1494, 1403, 1317, 1301, 1231, 1180, 1140, 1127, 1110, 1071, 1012, 840, 665.

4-((4-Chlorophenyl)sulfonyl)-1-(*p*-tolyl)-2,3-dihydro-1*H*-pyrrole (10)

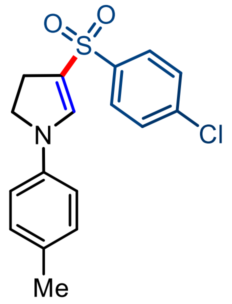

The title compound was synthesized according to the general procedure employing $Pd(OAc)_2$ (2.3 mg, 0.01 mmol), PPh_3 (10.5 mg, 0.04 mmol), K_2CO_3 (82.8 mg, 0.60 mmol), 1-(*p*-tolyl)pyrrolidine (32.2 mg, 0.20 mmol), 4-chlorobenzenesulfonyl chloride (126.6 mg, 0.60 mmol), and 1,4-dioxane (0.1 M, 2 mL) for 60 h. The product was purified by column chromatography (aluminum oxide, gradient 16 to 18% EA/*n*-hexane). Yield = 52.7 mg (79%). ^1H NMR (400 MHz, DMSO-d_6): δ 8.01 (s, 1H), 7.86 (d, J = 8.5 Hz, 2H), 7.66 (d, J = 8.5 Hz, 2H), 7.10 (d, J = 8.3 Hz, 2H), 7.02 (d, J = 8.4 Hz, 2H), 3.97 (t, J = 10.1 Hz, 2H), 2.74 (t, J = 10.1 Hz, 2H), 2.23 (s, 3H); ^{13}C NMR (101 MHz, DMSO-d_6): δ 143.95, 141.05, 138.42, 137.27, 130.37, 129.73, 129.43, 128.32, 114.45, 110.17, 49.97, 26.47, 20.14; GCMS (EI) *m/z* calc. for $C_{17}H_{16}ClNO_2S$ [M^+] 333.1, found 333.1, 268.1, 233.1, 174.1, 157.1, 143.1, 118.1, 91.1, 65.1; HRMS (ESI-TOF) m/z: $(M+Na)^+$ calcd. for $C_{17}H_{16}ClNO_2SNa$, 356.0482; found, 356.0516; FT-IR ν_{max}(ATR) cm^{-1}: 2857, 1572, 1514, 1392, 1364, 1313, 1280, 1177, 1143, 1110, 1085, 1012, 801, 775, 755, 622.

4-((4-Bromophenyl)sulfonyl)-1-(*p*-tolyl)-2,3-dihydro-1*H*-pyrrole (11)

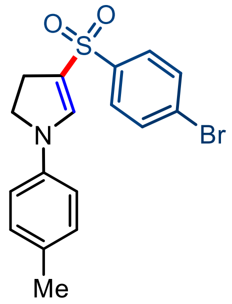

The title compound was synthesized according to the general procedure employing $Pd(OAc)_2$ (2.3 mg, 0.01 mmol), PPh_3 (10.5 mg, 0.04 mmol), K_2CO_3 (82.8 mg, 0.60 mmol), 1-(*p*-tolyl)pyrrolidine (32.2 mg, 0.20 mmol), 4-bromobenzenesulfonyl chloride (153.3 mg, 0.60 mmol), and 1,4-dioxane (0.1 M, 2 mL) for 60 h. The product was purified by column chromatography (aluminum oxide, gradient 16 to 18% EA/*n*-hexane). Yield = 57.4 mg (76%). ^1H NMR (400 MHz, DMSO-d_6): δ 8.00 (s, 1H), 7.88 – 7.66 (m, 4H), 7.10 (d, J = 8.4 Hz, 2H), 7.01 (d, J = 8.5 Hz, 2H), 3.97 (t, J = 10.1 Hz, 2H), 2.74 (t, J = 10.1 Hz, 2H), 2.22 (s, 3H); ^{13}C NMR (101 MHz, DMSO-d_6): δ 143.96, 141.47, 138.42, 132.38, 130.39, 129.74, 128.42, 126.25, 114.46, 110.14, 49.99, 26.48, 20.14; GCMS (EI) *m/z* calc. for $C_{17}H_{16}BrNO_2S$ [M^+] 377.0, found 379.0, 377.0, 314.0, 312.1, 233.1, 204.9, 174.1, 157.1, 143.1, 118.1, 65.1; HRMS (ESI-TOF) m/z: $(M+Na)^+$ calcd. for $C_{17}H_{16}BrNO_2SNa$, 399.9977; found, 400.0005; FT-IR ν_{max}(ATR) cm^{-1}: 2918, 1732, 1614, 1571, 1514, 1365, 1305, 1232, 1177, 1140, 1109, 1083, 1065, 1007, 798, 737.

1-(*p*-Tolyl)-4-((4-(trifluoromethyl)phenyl)sulfonyl)-2,3-dihydro-1*H*-pyrrole (12)

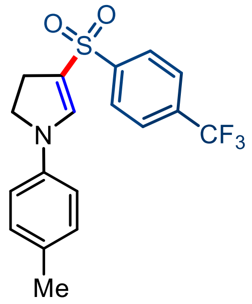

The title compound was synthesized according to the general procedure employing $Pd(OAc)_2$ (2.3 mg, 0.01 mmol), PPh_3 (10.5 mg, 0.04 mmol), K_2CO_3 (82.8 mg, 0.60 mmol), 1-(*p*-tolyl)pyrrolidine (32.2 mg, 0.20 mmol), 4-(trifluoromethyl)benzenesulfonyl chloride (146.8 mg, 0.60 mmol), and 1,4-dioxane (0.1 M, 2 mL) for 60 h. The product was purified by column chromatography (aluminum oxide, gradient 15 to 18% EA/*n*-hexane). Yield = 49.2 mg (67%).

^1H NMR (400 MHz, DMSO-d_6): δ 8.09 – 8.04 (m, 3H), 7.97 (d, J = 8.4 Hz, 2H), 7.10 (d, J = 8.5 Hz, 2H), 7.04 (d, J = 8.6 Hz, 2H), 4.06 – 3.89 (m, 2H), 2.77 (t, J = 10.1 Hz, 2H), 2.23 (s, 3H); ^{13}C NMR (101 MHz, DMSO-d_6): δ 146.12, 144.76, 138.29, 132.05 (q, $J_{C\text{-}F}$ = 32.0 Hz), 130.61, 129.74, 127.29, 126.55 (q, $J_{C\text{-}F}$ = 4.0 Hz), 123.53 (q, $J_{C\text{-}F}$ = 271.0 Hz), 114.58, 109.35, 50.07, 26.40, 20.14; GCMS (EI) *m/z* calc. for $C_{18}H_{16}F_3NO_2S$ [M^+] 367.1, found 367.1, 302.1, 284.1, 174.1, 157.1, 143.1, 130.1, 118.1, 91.1; HRMS (ESI-TOF) m/z: $(M+Na)^+$ calcd. for $C_{18}H_{16}F_3NO_2SNa$, 390.0746; found, 390.0762; FT-IR ν_{max}(ATR) cm^{-1}: 2921, 1565, 1521, 1436, 1402, 1319, 1299, 1259, 1192, 1142, 1105, 1073, 1013, 963, 840.

4-(Naphthalen-1-ylsulfonyl)-1-(*p*-tolyl)-2,3-dihydro-1*H*-pyrrole (13)

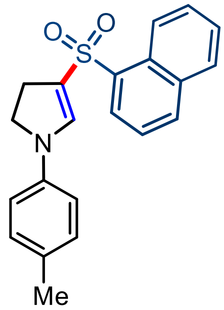

The title compound was synthesized according to the general procedure employing $Pd(OAc)_2$ (2.3 mg, 0.01 mmol), PPh_3 (10.5 mg, 0.04 mmol), K_2CO_3 (82.8 mg, 0.60 mmol), 1-(*p*-tolyl)pyrrolidine (32.2 mg, 0.20 mmol), naphthalene-1-sulfonyl chloride (135.9 mg, 0.60 mmol), and 1,4-dioxane (0.1 M, 2 mL) for 48 h. The product was purified by column chromatography (aluminum oxide, gradient 17 to 20% EA/*n*-hexane). Yield = 43.3 mg (62%).

^1H NMR (400 MHz, DMSO-d_6): δ 8.82 (d, J = 8.6 Hz, 1H), 8.29 – 8.17 (m, 3H), 8.09 (d, J = 8.1 Hz, 1H), 7.79 – 7.52 (m, 3H), 7.23 – 6.91 (m, 4H), 3.89 (t, J = 10.1 Hz, 2H), 2.65 (t, J = 10.1 Hz, 2H), 2.22 (s, 3H); ^{13}C NMR (101 MHz, DMSO-d_6): δ 143.67, 138.54, 136.16, 134.07, 133.89, 130.21, 129.72, 129.11, 128.69, 128.03, 127.49, 126.82, 124.77, 124.41, 114.40, 110.98, 49.86, 26.76, 20.12; GCMS (EI) *m/z* calc. for $C_{21}H_{19}NO_2S$ [M^+] 349.1, found 349.1, 284.1, 268.1, 174.1, 157.1, 143.0, 118.1, 105.1, 91.1, 77.1; HRMS (ESI-TOF) m/z: $(M+Na)^+$ calcd. for $C_{21}H_{19}NO_2SNa$, 372.1029; found, 372.1074; FT-IR ν_{max}(ATR) cm^{-1}: 2918, 1583, 1570, 1431, 1404, 1364, 1299, 1287, 1179, 1150, 1134, 1089, 1025, 991, 800, 767.

1-Phenyl-5-(*m*-tolylsulfonyl)-1,2,3,4-tetrahydropyridine (14)

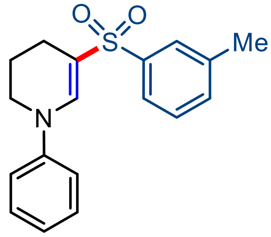

The title compound was synthesized according to the general procedure employing $Pd(OAc)_2$ (2.3 mg, 0.01 mmol), PPh_3 (10.5 mg, 0.04 mmol), K_2CO_3 (82.8 mg, 0.60 mmol), 1-phenylpiperidine (32.2 mg, 0.20 mmol), 3-methylbenzenesulfonyl chloride (114.4 mg, 0.60 mmol), and 1,4-dioxane (0.1 M, 2 mL) for 48 h. The product was purified by column

chromatography (aluminum oxide, gradient 18 to 20% EA/*n*-hexane). Yield = 46.3 mg (74%). ^{1}H NMR (400 MHz, DMSO-d_6): δ 7.73 (s, 1H), 7.70 – 7.58 (m, 2H), 7.57 – 7.31 (m, 4H), 7.20 (d, *J* = 8.0 Hz, 2H), 7.09 (t, *J* = 7.3 Hz, 1H), 3.56 (t, *J* = 5.5 Hz, 2H), 2.39 (s, 3H), 2.15 (t, *J* = 6.2 Hz, 2H), 1.85 (p, *J* = 6.0 Hz, 2H); ^{13}C NMR (101 MHz, DMSO-d_6): δ 144.95, 141.71, 138.97, 138.77, 133.03, 129.47, 129.07, 126.80, 123.90, 123.10, 117.49, 107.23, 45.22, 20.82, 20.59, 19.66; GCMS (EI) *m/z* calc. for $C_{18}H_{19}NO_2S$ [M$^+$] 313.1, found 313.1, 296.1, 249.2, 220.1, 206.1, 174.1, 156.1, 143.1, 129.1, 104.1; HRMS (ESI-TOF) m/z: (M+Na)$^+$ calcd. for $C_{18}H_{19}NO_2SNa$, 336.1029; found, 336.1045; FT-IR ν_{max}(ATR) cm^{-1} : 2916, 1712, 1628, 1591, 1493, 1465, 1282, 1268, 1252, 1130, 1093, 1075, 1037, 999, 907, 757.

5-((3-Fluorophenyl)sulfonyl)-1-phenyl-1,2,3,4-tetrahydropyridine (15)

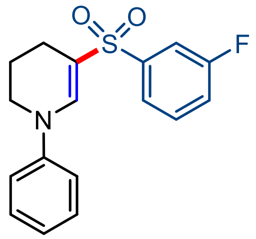

The title compound was synthesized according to the general procedure employing $Pd(OAc)_2$ (2.3 mg, 0.01 mmol), PPh_3 (10.5 mg, 0.04 mmol), K_2CO_3 (82.8 mg, 0.60 mmol), 1-phenylpiperidine (32.2 mg, 0.20 mmol), 3-fluorobenzenesulfonyl chloride (116.8 mg, 0.60 mmol), and 1,4-dioxane (0.1 M, 2 mL) for 48 h. The product was purified by column chromatography (aluminum oxide, gradient 17 to 19% EA/*n*-hexane). Yield = 50.7 mg (80%). ^{1}H NMR (400 MHz, DMSO-d_6): δ 7.77 (s, 1H), 7.72 – 7.60 (m, 3H), 7.49 (td, *J* = 8.7, 2.7 Hz, 1H), 7.39 (t, *J* = 7.8 Hz, 2H), 7.23 (d, *J* = 8.2 Hz, 2H), 7.10 (t, *J* = 7.3 Hz, 1H), 3.57 (t, *J* = 5.6 Hz, 2H), 2.16 (t, *J* = 6.1 Hz, 2H), 1.86 (p, *J* = 5.9 Hz, 2H); ^{13}C NMR (101 MHz, DMSO-d_6): δ 161.89 (d, *J*C-F = 249.5 Hz), 144.90, 144.15 (d, $J_{C\text{-}F}$ = 6.0 Hz), 139.85, 131.56 (d, $J_{C\text{-}F}$ = 8.0 Hz), 129.44, 123.33, 122.97 (d, $J_{C\text{-}F}$ = 3.0 Hz), 119.53 (d, $J_{C\text{-}F}$ = 21.2 Hz), 117.74, 113.71 (d, $J_{C\text{-}F}$ = 23.2 Hz), 106.02, 45.34, 20.56, 19.60; GCMS (EI) *m/z* calc. for $C_{17}H_{16}FNO_2S$ [M$^+$] 317.1, found 317.1, 300.1, 252.1, 224.1, 206.1, 174.1, 156.1, 143.1, 129.1, 117.1, 77.1; HRMS (ESI-TOF) m/z: (M+Na)$^+$ calcd. for $C_{17}H_{16}FNO_2Sna$, 340.0778; found, 340.0805; FT-IR ν_{max}(ATR) cm^{-1}: 2922, 1617, 1585, 1494, 1435, 1306, 1285, 1223, 1129, 1093, 1027, 1000, 966, 880, 691.

5-((2-Chlorophenyl)sulfonyl)-1-phenyl-1,2,3,4-tetrahydropyridine (16)

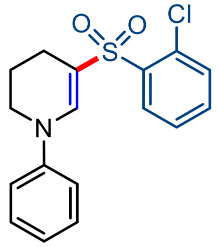

The title compound was synthesized according to the general procedure employing $Pd(OAc)_2$ (2.3 mg, 0.01 mmol), PPh_3 (10.5 mg, 0.04 mmol), K_2CO_3 (82.8 mg, 0.60 mmol), 1-phenylpiperidine (32.2 mg, 0.20 mmol), 2-chlorobenzenesulfonyl chloride (126.6 mg, 0.60 mmol), and 1,4-dioxane (0.1 M, 2 mL) for 48 h. The product was purified by column chromatography (aluminum oxide, gradient 17 to 19% EA/*n*-hexane). Yield = 57.4 mg (86%). ^{1}H NMR (400 MHz, DMSO-d_6): δ 8.07 (d, *J* = 8.2 Hz, 1H), 7.80 (s, 1H), 7.69 – 7.61 (m, 2H), 7.59-7.55 (m, 1H), 7.40 (t, *J* = 7.7 Hz, 2H), 7.19 (d, *J* = 8.1 Hz, 2H), 7.12 (t, *J* = 7.3 Hz, 1H), 3.61 (t, *J* = 5.5 Hz, 2H), 2.07 (t, *J* = 6.2 Hz, 2H), 1.83 (p, *J* = 5.9 Hz, 2H); ^{13}C NMR (101 MHz, DMSO-d_6): δ 144.91, 141.59, 137.61, 134.28, 132.15, 130.95, 130.86, 129.62, 127.79, 123.50, 117.73, 104.11, 45.52, 20.63, 19.54; GCMS (EI) *m/z* calc. for $C_{17}H_{16}ClNO_2S$ [M$^+$] 333.1,

found 333.1, 316.1, 240.1, 222.1, 206.1, 174.1, 156.1, 143.1, 129.1, 104.1, 77.1; HRMS (ESI-TOF) m/z: $(M+Na)^+$ calcd. for $C_{17}H_{16}ClNO_2SNa$, 356.0482; found, 356.0533; FT-IR ν_{max}(ATR) cm^{-1}: 2916, 1618, 1591, 1495, 1449, 1318, 1298, 1259, 1249, 1141, 1131, 1106, 1052, 1035, 986, 783.

5-((3-Chloro-4-fluorophenyl)sulfonyl)-1-phenyl-1,2,3,4-tetrahydropyridine (17)

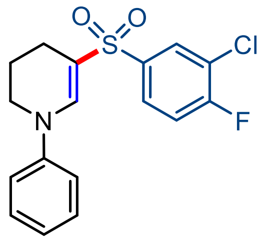

The title compound was synthesized according to the general procedure employing $Pd(OAc)_2$ (2.3 mg, 0.01 mmol), PPh_3 (10.5 mg, 0.04 mmol), K_2CO_3 (82.8 mg, 0.60 mmol), 1-phenylpiperidine (32.2 mg, 0.20 mmol), 3-chloro-4-fluorobenzenesulfonyl chloride (137.4 mg, 0.60 mmol), and 1,4-dioxane (0.1 M, 2 mL) for 60 h. The product was purified by column chromatography (aluminum oxide, gradient 18 to 20% EA/*n*-hexane). Yield = 57 mg (81%).

^{1}H NMR (400 MHz, DMSO-d_6): δ 8.09 (dd, J = 6.9, 2.3 Hz, 1H), 7.91-7.87 (m, 1H), 7.79 (s, 1H), 7.61 (t, J = 8.9 Hz, 1H), 7.38 (t, J = 7.9 Hz, 2H), 7.24 (d, J = 7.9 Hz, 2H), 7.10 (t, J = 7.3 Hz, 1H), 3.57 (t, J = 5.6 Hz, 2H), 2.16 (t, J = 6.2 Hz, 2H), 1.86 (p, J = 5.9 Hz, 2H); ^{13}C NMR (101 MHz, DMSO-d_6): δ 159.37 (d, $J_{C\text{-}F}$ = 254.5 Hz), 144.87, 140.05, 139.54 (d, $J_{C\text{-}F}$ = 4.0 Hz), 129.40, 129.29, 128.24 (d, $J_{C\text{-}F}$ = 9.1 Hz), 123.34, 120.74 (d, $J_{C\text{-}F}$ = 19.2 Hz), 117.99, 117.76, 105.85, 45.34, 20.54, 19.52; GCMS (EI) *m/z* calc. for $C_{17}H_{15}ClFNO_2S$ [M^+] 351.0, found 351.0, 334.0, 287.1, 258.0, 222.0, 174.1, 156.1, 143.1, 129.0, 117.0, 104.1; HRMS (ESI-TOF) m/z: $(M+Na)^+$ calcd. for $C_{17}H_{15}ClFNO_2Sna$, 374.0388; found, 374.0438; FT-IR ν_{max}(ATR) cm^{-1}: 2916, 1616, 1590, 1494, 1478, 1401, 1337, 1282, 1272, 1243, 1147, 1122, 1104, 1000, 925, 782.

1-Mesityl-4-tosyl-2,3-dihydro-1*H*-pyrrole (18)

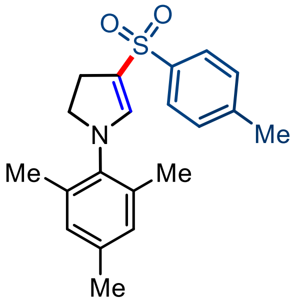

The title compound was synthesized according to the general procedure employing $Pd(OAc)_2$ (2.3 mg, 0.01 mmol), PPh_3 (10.5 mg, 0.04 mmol), K_2CO_3 (82.8 mg, 0.60 mmol), 1-mesitylpyrrolidine (37.9 mg, 0.20 mmol), tosyl chloride (114.4 mg, 0.60 mmol), and 1,4-dioxane (0.1 M, 2 mL) for 24 h. The product was purified by column chromatography (aluminum oxide, gradient 15 to 18% EA/*n*-hexane). Yield = 63.5 mg (93%).

^{1}H NMR (400 MHz, $CDCl_3$): δ 7.77 (d, J = 7.8 Hz, 2H), 7.30 (d, J = 5.7 Hz, 2H), 7.00 (s, 1H), 6.87 (s, 2H), 3.78 (t, J = 10.3 Hz, 2H), 2.88 (t, J = 9.2 Hz, 2H), 2.43 (s, 3H), 2.26 (s, 3H), 2.19 (s, 6H); ^{13}C NMR (101 MHz, $CDCl_3$): δ 150.54, 142.65, 139.79, 137.74, 136.30, 136.23, 129.62, 129.50, 126.78, 106.58, 53.77, 27.81, 21.58, 20.96, 18.04; GCMS (EI) *m/z* calc. for $C_{20}H_{23}NO_2S$ [M^+] 341.1, found 341.1, 324.1, 307.1, 276.1, 207.0, 185.1, 170.1, 146.0, 131.0, 119.1, 91.0; HRMS (ESI-TOF) m/z: $(M+Na)^+$ calcd. for $C_{20}H_{23}NO_2SNa$, 364.1342; found, 364.1354; FT-IR ν_{max}(ATR) cm^{-1}: 2864, 1572, 1487, 1446, 1403, 1307, 1297, 1286, 1221, 1137, 1119, 1100, 1033, 985, 866, 851.

1-(2,6-Dimethylphenyl)-4-tosyl-2,3-dihydro-1*H*-pyrrole (19)

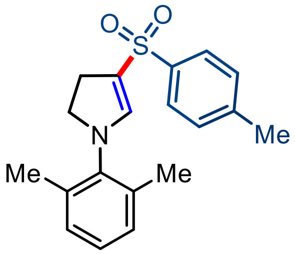

The title compound was synthesized according to the general procedure employing Pd(OAc)$_2$ (2.3 mg, 0.01 mmol), PPh$_3$ (10.5 mg, 0.04 mmol), K$_2$CO$_3$ (82.8 mg, 0.60 mmol), 1-(2,6-dimethylphenyl)pyrrolidine (35.0 mg, 0.20 mmol), tosyl chloride (114.4 mg, 0.60 mmol), and 1,4-dioxane (0.1 M, 2 mL) for 28 h. The product was purified by column chromatography (aluminum oxide, gradient 15 to 17% EA/*n*-hexane). Yield = 58.2 mg (89%).
^{1}H NMR (400 MHz, CDCl$_3$): δ 7.77 (d, *J* = 8.2 Hz, 2H), 7.31 (d, *J* = 8.0 Hz, 2H), 7.14 – 6.98 (m, 4H), 3.89 – 3.73 (m, 2H), 2.89 (t, *J* = 10.6 Hz, 2H), 2.43 (s, 3H), 2.24 (s, 6H); ^{13}C NMR (101 MHz, CDCl$_3$): δ 150.33, 142.75, 139.79, 138.90, 136.61, 129.68, 128.91, 127.99, 126.86, 107.09, 53.72, 27.88, 21.62, 18.20; GCMS (EI) *m/z* calc. for $C_{19}H_{21}NO_2S$ [M$^+$] 327.1, found 327.1, 262.1, 218.0, 188.0, 171.0, 156.1, 132.0, 105.0, 91.0, 77.0; HRMS (ESI-TOF) m/z: (M+Na)$^+$ calcd. for $C_{19}H_{21}NO_2SNa$, 350.1185; found, 350.1247; FT-IR ν_{max}(ATR) cm^{-1}: 2919, 1572, 1484, 1452, 1405, 1374, 1309, 1284, 1184, 1141, 1117, 1079, 1040, 992, 906, 818.

1-(4-(*tert*-Butyl)phenyl)-4-tosyl-2,3-dihydro-1*H*-pyrrole (20)

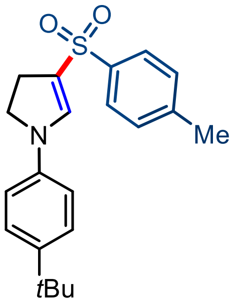

The title compound was synthesized according to the general procedure employing Pd(OAc)$_2$ (2.3 mg, 0.01 mmol), PPh$_3$ (10.5 mg, 0.04 mmol), K$_2$CO$_3$ (82.8 mg, 0.60 mmol), 1-(4-(*tert*-butyl)phenyl)pyrrolidine (40.6 mg, 0.20 mmol), tosyl chloride (114.4 mg, 0.60 mmol), and 1,4-dioxane (0.1 M, 2 mL) for 24 h. The product was purified by column chromatography (aluminum oxide, gradient 17 to 19% EA/*n*-hexane). Yield = 60.4 mg (85%). ^{1}H NMR (400 MHz, DMSO-*d*$_6$): δ 7.95 (s, 1H), 7.74 (d, *J* = 8.2 Hz, 2H), 7.40 (d, *J* = 8.1 Hz, 2H), 7.29 (d, *J* = 8.8 Hz, 2H), 7.02 (d, *J* = 8.8 Hz, 2H), 4.01 – 3.88 (m, 2H), 2.72 (t, *J* = 10.0 Hz, 2H), 2.38 (s, 3H), 1.24 (s, 9H); ^{13}C NMR (101 MHz, DMSO-*d*$_6$): δ 143.52, 142.88, 142.77, 139.22, 138.51, 129.76, 126.42, 125.95, 114.03, 111.43, 49.84, 33.81, 31.19, 26.64, 20.98; GCMS (EI) *m/z* calc. for $C_{21}H_{25}NO_2S$ [M$^+$] 355.2, found 355.1, 340.1, 216.1, 201.1, 184.1, 169.0, 156.0, 139.0, 118.0, 91.0; HRMS (ESI-TOF) m/z: (M+Na)$^+$ calcd. for $C_{21}H_{25}NO_2SNa$, 378.1498; found, 378.1525; FT-IR ν_{max}(ATR) cm^{-1}: 2954, 2922, 1595, 1512, 1348, 1323, 1299, 1286, 1250, 1176, 1141, 1086, 1062, 1050, 989, 836, 725.

1-(4-Methoxyphenyl)-4-tosyl-2,3-dihydro-1*H*-pyrrole (21)

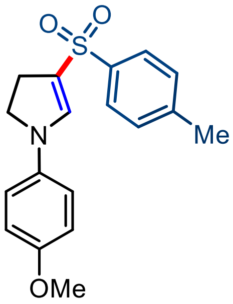

The title compound was synthesized according to the general procedure employing Pd(OAc)$_2$ (2.3 mg, 0.01 mmol), PPh$_3$ (10.5 mg, 0.04 mmol), K$_2$CO$_3$ (82.8 mg, 0.60 mmol), 1-(4-methoxyphenyl)pyrrolidine (35.4 mg, 0.20 mmol), tosyl chloride (114.4 mg, 0.60 mmol), and 1,4-dioxane (0.1 M, 2 mL) for 18 h. The product was purified by column chromatography (aluminum oxide, gradient 20 to 22% EA/*n*-hexane). Yield = 52 mg (79%).

^1H NMR (400 MHz, DMSO-d_6): δ 7.89 (s, 1H), 7.73 (d, *J* = 8.2 Hz, 2H), 7.39 (d, *J* = 7.9 Hz, 2H), 7.05 (d, *J* = 9.1 Hz, 2H), 6.87 (d, *J* = 9.0 Hz, 2H), 3.94 (t, *J* = 10.1 Hz, 2H), 3.70 (s, 3H), 2.70 (t, *J* = 10.1 Hz, 2H), 2.37 (s, 3H); ^{13}C NMR (101 MHz, DMSO-d_6): δ 154.19, 143.38, 142.64, 139.40, 134.67, 129.72, 126.36, 115.65, 114.62, 110.45, 55.26, 50.22, 26.68, 20.97; GCMS (EI) *m/z* calc. for $C_{18}H_{19}NO_3S$ [M^+] 329.1, found 329.1, 314.1, 250.1, 190.0, 173.0, 158.0, 134.0, 120.0, 107.0, 91.1; HRMS (ESI-TOF) m/z: $(M+Na)^+$ calcd. for $C_{18}H_{19}NO_3SNa$, 352.0978; found, 352.1021; FT-IR ν_{max}(ATR) cm^{-1}: 2917, 2848, 1592, 1576, 1506, 1474, 1431, 1365, 1282, 1241, 1176, 1140, 1085, 1031, 989, 812.

1-Phenyl-4-tosyl-2,3-dihydro-1*H*-pyrrole (22)

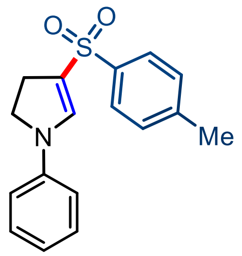

The title compound was synthesized according to the general procedure employing $Pd(OAc)_2$ (2.3 mg, 0.01 mmol), PPh_3 (10.5 mg, 0.04 mmol), K_2CO_3 (82.8 mg, 0.60 mmol), 1-phenylpyrrolidine (29.4 mg, 0.20 mmol), tosyl chloride (114.4 mg, 0.60 mmol), and 1,4-dioxane (0.1 M, 2 mL) for 48 h. The product was purified by column chromatography (aluminum oxide, gradient 16 to 18% EA/*n*-hexane). Yield = 50.8 mg (85%).

^1H NMR (400 MHz, DMSO-d_6): δ 7.99 (s, 1H), 7.74 (d, *J* = 6.8 Hz, 2H), 7.41 (d, *J* = 7.3 Hz, 2H), 7.29 (d, *J* = 7.2 Hz, 2H), 7.10 (d, *J* = 7.7 Hz, 2H), 6.93 (t, *J* = 7.3 Hz, 1H), 3.98 (t, *J* = 10.0 Hz, 2H), 2.73 (t, *J* = 10.0 Hz, 2H), 2.38 (s, 3H); ^{13}C NMR (101 MHz, DMSO-d_6): δ 142.85, 142.63, 140.90, 139.11, 129.77, 129.30, 126.47, 121.08, 114.25, 112.30, 49.73, 26.62, 20.98; GCMS (EI) *m/z* calc. for $C_{17}H_{17}NO_2S$ [M^+] 299.1, found 299.1, 282.0, 234.1, 174.1, 157.1, 143.1, 118.1, 91.1, 77.0, 65.0; HRMS (ESI-TOF) m/z: $(M+Na)^+$ calcd. for $C_{17}H_{17}NO_2SNa$, 322.0872; found, 322.0895; FT-IR ν_{max}(ATR) cm^{-1}: 2919, 1588, 1573, 1458, 1412, 1367, 1309, 1297, 1284, 1184, 1141, 1111, 1075, 1034, 997, 851, 706, 682.

1-(4-Fluorophenyl)-4-tosyl-2,3-dihydro-1*H*-pyrrole (23)

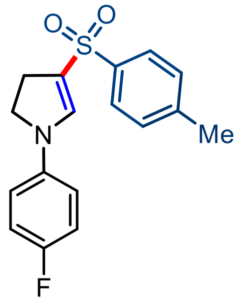

The title compound was synthesized according to the general procedure employing $Pd(OAc)_2$ (2.3 mg, 0.01 mmol), PPh_3 (10.5 mg, 0.04 mmol), K_2CO_3 (82.8 mg, 0.60 mmol), 1-(4-fluorophenyl)pyrrolidine (33.0 mg, 0.20 mmol), tosyl chloride (114.4 mg, 0.60 mmol), and 1,4-dioxane (0.1 M, 2 mL) for 60 h. The product was purified by column chromatography (aluminum oxide, gradient 17 to 19% EA/*n*-hexane). Yield = 52.6 mg (83%).

^1H NMR (400 MHz, DMSO-d_6): δ 7.97 (s, 1H), 7.74 (d, *J* = 8.2 Hz, 2H), 7.40 (d, *J* = 8.1 Hz, 2H), 7.12 (d, *J* = 6.5 Hz, 4H), 3.96 (t, *J* = 10.1 Hz, 2H), 2.72 (t, *J* = 10.0 Hz, 2H), 2.38 (s, 3H); ^{13}C NMR (101 MHz, DMSO-d_6): δ 157.13 (d, $J_{C\text{-}F}$ = 239.4 Hz), 143.03, 142.85, 139.12, 137.63, 129.76, 126.46, 115.88 (d, $J_{C\text{-}F}$ = 13.1 Hz), 115.78 (d, $J_{C\text{-}F}$ = 21.2 Hz), 112.19, 50.14, 26.72, 20.98; ^{19}F NMR (377 MHz, DMSO-d_6) δ 122.94; GCMS (EI) *m/z* calc. for $C_{17}H_{16}FNO_2S$ [M^+] 317.1, found 317.0, 300.0, 252.1, 178.0, 161.0, 122.0, 95.0, 65.0, 39.1; HRMS (ESI-TOF) m/z: $(M+Na)^+$ calcd. for $C_{17}H_{16}FNO_2SNa$, 340.0778; found, 340.0827; FT-IR

ν_{max}(ATR) cm^{-1} : 2924, 2863, 1733, 1595, 1573, 1507, 1438, 1335, 1264, 1180, 1140, 1113, 1086, 1016, 994, 847.

1-(4-Chlorophenyl)-4-tosyl-2,3-dihydro-1*H*-pyrrole (24)

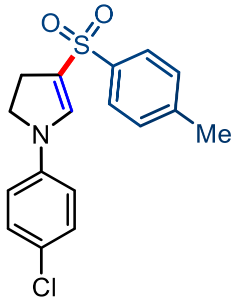

The title compound was synthesized according to the general procedure employing $Pd(OAc)_2$ (2.3 mg, 0.01 mmol), PPh_3 (10.5 mg, 0.04 mmol), K_2CO_3 (82.8 mg, 0.60 mmol), 1-(4-chlorophenyl)pyrrolidine (36.3 mg, 0.20 mmol), tosyl chloride (114.4 mg, 0.60 mmol), and 1,4-dioxane (0.1 M, 2 mL) for 60 h. The product was purified by column chromatography (aluminum oxide, gradient 16 to 18% EA/*n*-hexane). Yield = 50 mg (75%).

^{1}H NMR (400 MHz, DMSO-d_6): δ 8.00 (s, 1H), 7.74 (d, *J* = 8.2 Hz, 2H), 7.41 (d, *J* = 8.1 Hz, 2H), 7.31 (d, *J* = 8.9 Hz, 2H), 7.12 (d, *J* = 8.9 Hz, 2H), 3.96 (t, *J* = 10.0 Hz, 2H), 2.73 (t, *J* = 10.0 Hz, 2H), 2.38 (s, 3H); ^{13}C NMR (101 MHz, DMSO-d_6): δ 142.99, 142.43, 139.92, 138.92, 129.80, 129.01, 126.53, 124.72, 115.90, 113.36, 49.86, 26.71, 21.00; GCMS (EI) *m/z* calc. for $C_{17}H_{16}ClNO_2S$ [M^+] 333.1, found 333.0, 268.1, 194.0, 177.0, 155.0, 138.0, 111.0, 91.0, 77.0, 65.0; HRMS (ESI-TOF) m/z: $(M+Na)^+$ calcd. for $C_{17}H_{16}ClNO_2SNa$, 356.0482; found, 356.0511; FT-IR ν_{max}(ATR) cm^{-1}: 2922, 1594, 1496, 1415, 1369, 1289, 1178, 1143, 1085, 1033, 1009, 807, 713, 679.

1-(2-Methoxyphenyl)-4-tosyl-2,3-dihydro-1*H*-pyrrole (25)

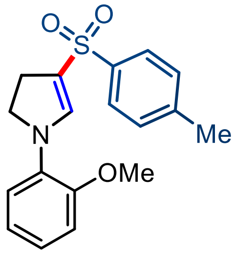

The title compound was synthesized according to the general procedure employing $Pd(OAc)_2$ (2.3 mg, 0.01 mmol), PPh_3 (10.5 mg, 0.04 mmol), K_2CO_3 (82.8 mg, 0.60 mmol), 1-(2-methoxyphenyl)pyrrolidine (35.4 mg, 0.20 mmol), tosyl chloride (114.4 mg, 0.60 mmol), and 1,4-dioxane (0.1 M, 2 mL) for 40 h. The product was purified by column chromatography (aluminum oxide, gradient 18 to 20% EA/*n*-hexane). Yield = 58 mg (88%).

^{1}H NMR (400 MHz, DMSO-d_6): δ 7.79 (s, 1H), 7.71 (d, *J* = 8.2 Hz, 2H), 7.41 (d, *J* = 8.1 Hz, 2H), 7.08-7.03 (m, 2H), 6.98-6.91 (m, 2H), 4.01 (t, *J* = 10.0 Hz, 2H), 3.84 (s, 3H), 2.65 (t, *J* = 10.0 Hz, 2H), 2.39 (s, 3H); ^{13}C NMR (101 MHz, DMSO-d_6): δ 150.32, 147.13, 142.71, 139.30, 130.47, 129.77, 126.36, 123.82, 121.04, 118.88, 112.46, 110.20, 55.85, 52.45, 26.07, 20.98; GCMS (EI) *m/z* calc. for $C_{18}H_{19}NO_3S$ [M^+] 329.1, found 329.1, 264.1, 250.1, 190.1, 173.0, 159.0, 134.0, 120.0, 107.0, 91.0; HRMS (ESI-TOF) m/z: $(M+Na)^+$ calcd. for $C_{18}H_{19}NO_3SNa$, 352.0978; found, 352.1023; FT-IR ν_{max}(ATR) cm^{-1} : 2918, 1571, 1503, 1461, 1403, 1331, 1285, 1237, 1143, 1102, 1074, 1049, 868, 832, 775, 706.

1-(4-Methoxy-2-methylphenyl)-4-tosyl-2,3-dihydro-1*H*-pyrrole (26)

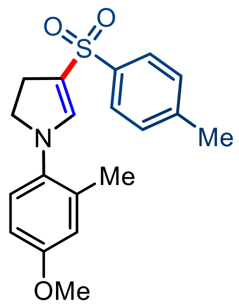

The title compound was synthesized according to the general procedure employing $Pd(OAc)_2$ (2.3 mg, 0.01 mmol), PPh_3 (10.5 mg, 0.04 mmol), K_2CO_3 (82.8 mg, 0.60 mmol), 1-(4-methoxy-2-methylphenyl)pyrrolidine (38.2 mg, 0.20 mmol), tosyl chloride (114.4 mg, 0.60 mmol), and 1,4-dioxane (0.1 M, 2 mL) for 30 h. The product was purified by column chromatography (aluminum oxide, gradient 18 to 20% EA/*n*-hexane). Yield = 59 mg (86%).

^{1}H NMR (400 MHz, DMSO-d_6): δ 7.70 (d, J = 8.2 Hz, 2H), 7.40 (d, J = 8.0 Hz, 2H), 7.28 (s, 1H), 7.04 (d, J = 8.6 Hz, 1H), 6.82 (d, J = 2.9 Hz, 1H), 6.75 (dd, J = 8.6, 3.0 Hz, 1H), 3.82 (t, J = 10.1 Hz, 2H), 3.72 (s, 3H), 2.67 (t, J = 10.0 Hz, 2H), 2.39 (s, 3H), 2.21 (s, 3H); ^{13}C NMR (101 MHz, DMSO-d_6): δ 157.21, 149.54, 142.53, 139.65, 134.39, 133.98, 129.72, 126.28, 124.93, 116.33, 111.99, 108.84, 55.24, 54.51, 27.32, 20.97, 18.18; GCMS (EI) *m/z* calc. for $C_{19}H_{21}NO_3S$ [M^+] 343.1, found 343.1, 328.0, 278.1, 264.1, 204.0, 187.0, 172.0, 148.0, 134.0, 117.0, 91.0; HRMS (ESI-TOF) m/z: $(M+Na)^+$ calcd. for $C_{19}H_{21}NO_3SNa$, 366.1134; found, 366.1177; FT-IR ν_{max}(ATR) cm^{-1}: 2921, 2851, 1731, 1594, 1501, 1453, 1287, 1232, 1177, 1140, 1083, 1043, 832, 813, 803, 674.

1-(2-Fluorophenyl)-4-tosyl-2,3-dihydro-1*H*-pyrrole (27)

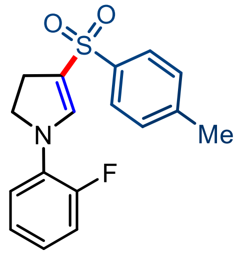

The title compound was synthesized according to the general procedure employing $Pd(OAc)_2$ (2.3 mg, 0.01 mmol), PPh_3 (10.5 mg, 0.04 mmol), K_2CO_3 (82.8 mg, 0.60 mmol), 1-(2-fluorophenyl)pyrrolidine (33.0 mg, 0.20 mmol), tosyl chloride (114.4 mg, 0.60 mmol), and 1,4-dioxane (0.1 M, 2 mL) for 48 h. The product was purified by column chromatography (aluminum oxide, gradient 15 to 17% EA/*n*-hexane). Yield = 48.8 mg (77%).

^{1}H NMR (400 MHz, DMSO-d_6): δ 7.73 (d, J = 8.2 Hz, 2H), 7.68 (s, 1H), 7.41 (d, J = 8.0 Hz, 2H), 7.31 – 7.19 (m, 1H), 7.18 – 7.08 (m, 2H), 7.07 – 6.96 (m, 1H), 4.07 (t, J = 9.9 Hz, 2H), 2.70 (t, J = 9.9 Hz, 2H), 2.39 (s, 3H); ^{13}C NMR (101 MHz, DMSO-d_6): δ 152.59 (d, J_{C-F} = 243.2 Hz), 145.03 (d, J_{C-F} = 10.6 Hz), 143.07, 138.76, 129.84, 129.41 (d, J_{C-F} = 8.9 Hz), 126.54, 125.20 (d, J_{C-F} = 3.3 Hz), 123.14 (d, J_{C-F} = 7.9 Hz), 119.15 (d, J_{C-F} = 2.4 Hz), 116.58 (d, J_{C-F} = 20.6 Hz),113.16, 52.01, 26.24, 21.00; ^{19}F NMR (377 MHz, DMSO-d_6): δ 125.74; GCMS (EI) *m/z* calc. for $C_{17}H_{16}FNO_2S$ [M^+] 317.1, found 317.0, 300.0, 252.1, 238.1, 178.0, 161.0, 134.0, 122.0, 109.0, 95.0, 77.0; HRMS (ESI-TOF) m/z: $(M+Na)^+$ calcd. for $C_{17}H_{16}FNO_2SNa$, 340.0778; found, 340.0801; FT-IR ν_{max}(ATR) cm^{-1}: 2924, 1595, 1570, 1518, 1505, 1404, 1339, 1300, 1287, 1185, 1143, 1117, 1096, 996, 920, 787, 705.

1-Phenyl-6-tosyl-2,3,4,5-tetrahydro-1*H*-azepine (28)

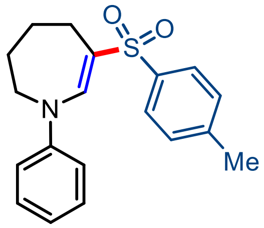

The title compound was synthesized according to the general procedure employing $Pd(OAc)_2$ (2.3 mg, 0.01 mmol), PPh_3 (10.5 mg, 0.04 mmol), K_2CO_3 (82.8 mg, 0.60 mmol), 1-phenylazepane (35.0 mg, 0.20 mmol), tosyl chloride (114.4 mg, 0.60 mmol), and 1,4-dioxane (0.1 M, 2 mL) for 48 h. The product was purified by column chromatography (aluminum oxide, gradient 18 to 20% EA/*n*-hexane). Yield = 53 mg (81%).

^{1}H NMR (400 MHz, DMSO-d_6): δ 7.72 (d, J = 8.0 Hz, 2H), 7.59 (s, 1H), 7.45 – 7.33 (m, 4H), 7.20 (d, J = 7.6 Hz, 2H), 7.13 (t, J = 7.3 Hz, 1H), 3.87 – 3.74 (m, 2H), 2.39 (s, 3H), 2.35 – 2.27 (m, 2H), 1.78 (p, J = 6.2 Hz, 2H), 1.64 (q, J = 6.1 Hz, 2H); ^{13}C NMR (101 MHz, DMSO-d_6): δ 146.92, 143.89, 142.79, 138.72, 129.74, 129.50, 126.90, 123.61, 119.31, 113.08, 50.82, 27.22, 25.51, 24.94, 20.99; GCMS (EI) *m/z* calc. for $C_{19}H_{21}NO_2S$ [M^+] 327.1, found 327.2, 273.0, 222.2, 177.1, 156.1, 143.1, 131.2, 119.1, 104.1, 77.1; HRMS (ESI-TOF) m/z: $(M+Na)^+$ calcd. for $C_{19}H_{21}NO_2SNa$, 350.1185; found, 350.1186; FT-IR ν_{max}(ATR) cm^{-1}: 2918, 2849, 1629, 1591, 1491, 1438, 1394, 1356, 1279, 1185, 1138, 1083, 1002, 904, 800, 706.

(*E*)-*N*-Ethyl-*N*-(2-tosylvinyl)aniline (29)

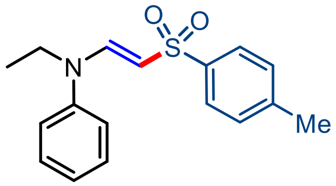

The title compound was synthesized according to the general procedure employing $Pd(OAc)_2$ (2.3 mg, 0.01 mmol), PPh_3 (10.5 mg, 0.04 mmol), K_2CO_3 (82.8 mg, 0.60 mmol), *N*,*N*-diethylaniline (29.8 mg, 0.20 mmol), tosyl chloride (114.4 mg, 0.60 mmol), and 1,4-dioxane (0.1 M, 2 mL) for 60 h. The product was purified by column chromatography (aluminum oxide, gradient 20 to 22% EA/*n*-hexane). Yield = 37.3 mg (62%).

^{1}H NMR (400 MHz, DMSO-d_6): δ 7.72 (d, J = 8.0 Hz, 2H), 7.57 (d, J = 12.9 Hz, 1H), 7.47 – 7.33 (m, 4H), 7.23 (dd, J = 14.7, 7.8 Hz, 3H), 5.68 (broad, 1H), 3.70 (q, J = 7.0 Hz, 2H), 2.37 (s, 3H), 1.06 (t, J = 7.0 Hz, 3H); ^{13}C NMR (101 MHz, DMSO-d_6): δ 145.29, 144.29, 142.21, 141.75, 129.72, 129.59, 126.02, 124.90, 120.79, 98.54, 44.60, 20.94, 11.53; GCMS (EI) *m/z* calc. for $C_{17}H_{19}NO_2S$ [M^+] 301.1, found 301.1, 208.1, 193.1, 146.1, 130.1, 118.1, 104.1, 91.1, 77.1, 65.1; HRMS (ESI-TOF) m/z: $(M+Na)^+$ calcd. for $C_{17}H_{19}NO_2SNa$, 324.1029; found, 324.1066; FT-IR ν_{max}(ATR) cm^{-1}: 2976, 1609, 1583, 1494, 1403, 1327, 1294, 1281, 1260, 1182, 1129, 1078, 1035, 1017, 865, 811, 721.

(*E*)-*N*,*N*-Diethyl-2-tosylethen-1-amine (30)

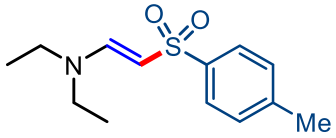

The title compound was synthesized according to the general procedure employing $Pd(OAc)_2$ (2.3 mg, 0.01 mmol), *rac*-BINAP (12.5 mg, 0.02 mmol), K_2CO_3 (82.8 mg, 0.60 mmol), triethylamine (20.2 mg, 0.20 mmol), tosyl chloride (114.4 mg, 0.60 mmol), and 1,4-dioxane (0.1 M, 2 mL) for 60 h. The product was purified by column chromatography (aluminum oxide, gradient 20 to 22% EA/*n*-hexane). Yield = 46 mg (91%).

^{1}H NMR (400 MHz, DMSO-d_6): δ 7.64 (d, J = 8.2 Hz, 2H), 7.32 (d, J = 8.1 Hz, 2H), 7.23 (d, J = 12.7 Hz, 1H), 5.07 (d, J = 12.7 Hz, 1H), 3.27 (broad, 2H), 3.09 (broad, 2H), 2.35 (s, 3H), 1.07 (broad, 3H), 0.99 (broad, 3H); ^{13}C NMR (101 MHz, DMSO-d_6): δ 148.96, 143.17, 141.44, 129.38, 125.51, 90.98, 48.97, 41.80, 20.89, 14.58, 10.94; GCMS (EI) *m/z* calc. for $C_{13}H_{19}NO_2S$ [M^+] 253.1, found 253.1, 224.1, 174.1, 160.1, 146.1, 133.1, 119.1, 98.1, 82.1, 56.1; HRMS (ESI-TOF) m/z: $(M+Na)^+$ calcd. for $C_{13}H_{19}NO_2SNa$, 276.1029; found, 276.1073; FT-IR ν_{max}(ATR) cm^{-1}: 2976, 2933, 1609, 1513, 1466, 1422, 1381, 1278, 1248, 1152, 1126, 1076, 1033, 1010, 876, 811, 684, 656.

(*E*)-*N*,*N*-bis(ethyl-d$_5$)-2-tosylethen-1-amine-1-d (30-D)

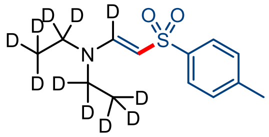

The title compound was synthesized according to the general procedure employing $Pd(OAc)_2$ (2.3 mg, 0.01 mmol), *rac*-BINAP (12.5 mg, 0.02 mmol), K_2CO_3 (82.8 mg, 0.60 mmol), d_{15}-triethylamine (23.2 mg, 0.20 mmol), tosyl chloride (114.4 mg, 0.60 mmol), and 1,4-dioxane (0.1 M, 2 mL) for 48 h. The product was purified by column chromatography (aluminum oxide, gradient 20 to 22% EA/*n*-hexane). Yield = 41.4 mg (78%).

^{1}H NMR (400 MHz, DMSO-d_6): δ 7.64 (d, J = 8.3 Hz, 2H), 7.32 (d, J = 7.8 Hz, 2H), 5.05 (s, 1H), 2.35 (s, 3H); ^{13}C NMR (101 MHz, DMSO-d_6): δ 149.07, 143.24, 141.48, 129.43, 125.54, 90.71, 48.57, 41.66, 20.92, 13.23, 8.68; GCMS (EI) *m/z* calc. for $C_{13}H_8D_{11}NO_2S$ [M^+] 264.1, found 264.2, 246.1, 200.2, 182.1, 182.1, 167.2, 150.1, 136.1, 123.1, 109.2, 89.1; HRMS (ESI-TOF) m/z: $(M+Na)^+$ calcd. for $C_{13}H_8D_{11}NO_2SNa$, 287.1725; found, 287.1719.

(*E*)-*N*,*N*-Dibutyl-2-tosylbut-1-en-1-amine (31)

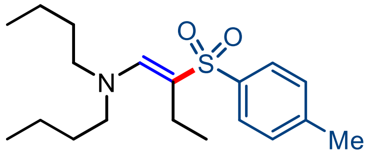

The title compound was synthesized according to the general procedure employing $Pd(OAc)_2$ (2.3 mg, 0.01 mmol), *rac*-BINAP (12.5 mg, 0.02 mmol), K_2CO_3 (82.8 mg, 0.60 mmol), tributylamine (37.1 mg, 0.20 mmol), tosyl chloride (114.4 mg, 0.60 mmol), and 1,4-dioxane (0.1 M, 2 mL) for 60 h. The product was purified by column chromatography (aluminum oxide, gradient 18 to 20% EA/*n*-hexane). Yield = 50 mg (74%).

^{1}H NMR (500 MHz, DMSO-d_6): δ 7.60 (d, J = 7.6 Hz, 2H), 7.33 (d, J = 7.7 Hz, 2H), 7.20 (s, 1H), 3.18 (t, J = 7.3 Hz, 4H), 2.35 (s, 3H), 2.11 (q, J = 7.0 Hz, 2H), 1.47 (p, J = 7.7 Hz, 4H), 1.25 (h, J = 7.4 Hz, 4H), 0.95 – 0.80 (m, 9H); ^{13}C NMR (101 MHz, DMSO-d_6): δ 145.20, 141.85, 140.82, 129.41, 126.49, 102.95, 51.76, 30.70, 20.88, 19.12, 18.16, 15.87, 13.64; GCMS (EI) *m/z* calc. for $C_{19}H_{31}NO_2S$ [M^+] 337.2, found 337.2, 322.2, 294.1, 217.2, 202.2, 182.2, 166.2, 140.2, 126.1, 105.1, 84.1; HRMS (ESI-TOF) m/z: $(M+Na)^+$ calcd. for $C_{19}H_{31}NO_2SNa$, 360.1968; found, 360.1997; FT-IR ν_{max}(ATR) cm^{-1}: 2925, 1614, 1491, 1338, 1318, 1263, 1122, 1080, 1050, 817, 820, 799, 682.

(*E*)-*N*,*N*-Dihexyl-2-tosylhex-1-en-1-amine (32)

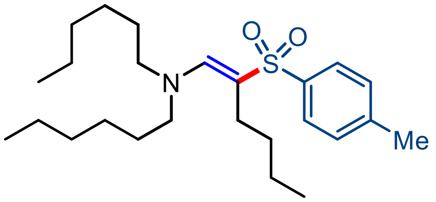

The title compound was synthesized according to the general procedure employing $Pd(OAc)_2$ (2.3 mg, 0.01 mmol), *rac*-BINAP (12.5 mg, 0.02 mmol), K_2CO_3 (82.8 mg, 0.60 mmol), trihexylamine (53.9 mg, 0.20 mmol), tosyl chloride (114.4 mg, 0.60 mmol), and 1,4-dioxane (0.1 M, 2 mL) for 60 h. The product was purified by column chromatography (aluminum oxide, gradient 18 to 20% EA/*n*-hexane). Yield = 57.3 mg (68%).

^{1}H NMR (400 MHz, DMSO-d_6): δ 7.58 (d, J = 8.0 Hz, 2H), 7.32 (d, J = 8.0 Hz, 2H), 7.19 (s, 1H), 3.16 (t, J = 7.4 Hz, 4H), 2.35 (s, 3H), 2.04 (dd, J = 9.8, 5.7 Hz, 2H), 1.47 (p, J = 7.3 Hz, 4H), 1.34 – 1.04 (m, 16H), 0.83 (q, J = 6.5 Hz, 6H), 0.76 (t, J = 6.9 Hz, 3H); ^{13}C NMR (101 MHz, DMSO-d_6): δ 145.32, 141.82, 140.81, 129.35, 126.43, 101.86, 52.03, 33.06, 30.91, 28.49, 25.49, 24.83, 22.05, 21.91, 20.88, 13.79, 13.56; GCMS (EI) *m/z* calc. for $C_{25}H_{43}NO_2S$ [M^+] 421.3, found 421.3, 378.2, 350.2, 294.1, 266.3, 230.2, 196.2, 182.2, 166.1, 152.1, 140.1, 126.1, 112.1, 91.1; HRMS (ESI-TOF) m/z: $(M+Na)^+$ calcd. for $C_{25}H_{43}NO_2SNa$, 444.2907; found, 444.2942; FT-IR ν_{max}(ATR) cm^{-1}: 2957, 2927, 2871, 1615, 1457, 1420, 1372, 1277, 1181, 1141, 1116, 1076, 924, 812, 707, 658.

(*E*)-*N*-Isopropyl-*N*-(2-tosylvinyl)propan-2-amine (33)

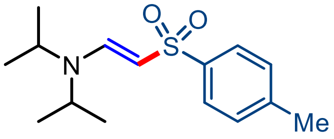

The title compound was synthesized according to the general procedure employing $Pd(OAc)_2$ (2.3 mg, 0.01 mmol), *rac*-BINAP (12.5 mg, 0.02 mmol), K_2CO_3 (82.8 mg, 0.60 mmol), *N*,*N*-diisopropylethylamine (25.8 mg, 0.20 mmol), tosyl chloride (114.4 mg, 0.60 mmol), and 1,4-dioxane (0.1 M, 2 mL) for 60 h. The product was purified by column chromatography (aluminum oxide, gradient 18 to 20% EA/*n*-hexane). Yield = 48.4 mg (86%).

^{1}H NMR (400 MHz, DMSO-d_6): δ 7.63 (d, J = 8.1 Hz, 2H), 7.32 (d, J = 8.1 Hz, 2H), 7.22 (d, J = 12.8 Hz, 1H), 5.22 (d, J = 12.8 Hz, 1H), 3.67 (broad, 2H), 2.35 (s, 3H), 1.11 (broad, 12H); ^{13}C NMR (101 MHz, DMSO-d_6): δ 144.65, 143.11, 141.44, 129.43, 125.48, 91.48, 48.61, 46.83, 23.13, 20.87, 18.99; GCMS (EI) *m/z* calc. for $C_{15}H_{23}NO_2S$ [M^+] 281.1, found 281.1, 266.1, 224.0, 174.1, 160.1, 139.0, 126.1, 110.1, 84.1, 68.1, 43.1; HRMS (ESI-TOF) m/z: $(M+Na)^+$ calcd. for $C_{15}H_{23}NO_2SNa$, 304.1342; found, 304.1373; FT-IR ν_{max}(ATR) cm^{-1}: 2968, 2925, 1600, 1495, 1459, 1436, 1315, 1274, 1259, 1129, 1099, 1078, 1016, 910, 886, 850, 816, 705, 683, 656.

(*E*)-*N*-Ethyl-*N*-methyl-2-tosylethen-1-amine (34)

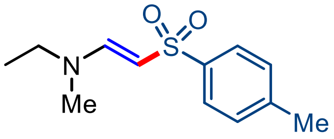

The title compound was synthesized according to the general procedure employing $Pd(OAc)_2$ (2.3 mg, 0.01 mmol), *rac*-BINAP (12.5 mg, 0.02 mmol), K_2CO_3 (82.8 mg, 0.60 mmol), *N*,*N*-diethylmethylamine (17.4 mg, 0.20 mmol), tosyl chloride (114.4 mg, 0.60 mmol), and 1,4-dioxane (0.1 M, 2 mL) for 60 h. The product was purified by column chromatography (aluminum oxide, gradient 18 to 20% EA/*n*-hexane). Yield = 31 mg (65%).

^{1}H NMR (400 MHz, DMSO-d$_6$): δ 7.64 (d, *J* = 8.1 Hz, 2H), 7.32 (d, *J* = 8.2 Hz, 3H), 5.00 (d, *J* = 12.0 Hz, 1H), 3.30 (d, *J* = 6.6 Hz, 2H), 3.03 (broad, 1H), 2.66 (s, 2H), 2.35 (s, 3H), 1.06 (broad, 3H); ^{13}C NMR (101 MHz, DMSO-*d*$_6$): δ 150.10, 143.09, 141.50, 129.39, 125.58, 91.74, 51.18, 34.56, 20.89, 13.94; GCMS (EI) *m/z* calc. for $C_{12}H_{17}NO_2S$ [M$^+$] 239.1, found 239.1, 224.0, 175.1, 160.1, 146.1, 119.1, 105.1, 84.1, 68.1, 55.1, 42.1; HRMS (ESI-TOF) m/z: (M+Na)$^+$ calcd. for $C_{12}H_{17}NO_2SNa$, 262.0872; found, 262.0899; FT-IR ν_{max}(ATR) cm^{-1}: 2973, 2923, 1611, 1494, 1407, 1380, 1306, 1292, 1264, 1129, 1077, 1017, 970, 889, 809, 789, 656.

1-Methyl-5-tosyl-1,2,3,4-tetrahydropyridine (35)

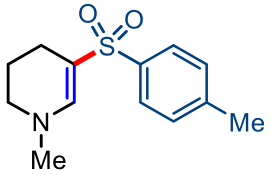

The title compound was synthesized according to the general procedure employing Pd(OAc)$_2$ (2.3 mg, 0.01 mmol), *rac*-BINAP (12.5 mg, 0.02 mmol), K_2CO_3 (82.8 mg, 0.60 mmol), 1-methylpiperidine (19.8 mg, 0.20 mmol), tosyl chloride (114.4 mg, 0.60 mmol), and 1,4-dioxane (0.1 M, 2 mL) for 60 h. The product was purified by column chromatography (aluminum oxide, gradient 18 to 20% EA/*n*-hexane). Yield = 33.6 mg (67%).

^{1}H NMR (400 MHz, DMSO-*d*$_6$): δ 7.59 (d, *J* = 8.2 Hz, 2H), 7.33 (d, *J* = 8.0 Hz, 2H), 7.24 (s, 1H), 2.98 (t, *J* = 5.7 Hz, 2H), 2.94 (s, 3H), 2.36 (s, 3H), 1.97 (t, *J* = 6.1 Hz, 2H), 1.66 (p, *J* = 6.1 Hz, 2H); ^{13}C NMR (101 MHz, DMSO-*d*$_6$): δ 144.76, 141.82, 140.32, 129.47, 126.15, 98.21, 46.53, 42.05, 20.91, 20.39, 19.07; GCMS (EI) *m/z* calc. for $C_{13}H_{17}NO_2S$ [M$^+$] 251.1, found 251.1, 234.1, 186.1, 160.0, 144.0, 112.0, 95.0, 81.0, 65.0, 42.0; HRMS (ESI-TOF) m/z: (M+Na)$^+$ calcd. for $C_{13}H_{17}NO_2SNa$, 274.0872; found, 274.0884; FT-IR ν_{max}(ATR) cm^{-1} : 2915, 2848, 1620, 1491, 1415, 1315, 1270, 1182, 1133, 1108, 1076, 1064, 1021, 984, 825, 808, 704.

1-Ethyl-5-tosyl-1,2,3,4-tetrahydropyridine (36)

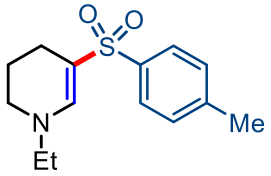

The title compound was synthesized according to the general procedure employing Pd(OAc)$_2$ (2.3 mg, 0.01 mmol), *rac*-BINAP (12.5 mg, 0.02 mmol), K_2CO_3 (82.8 mg, 0.60 mmol), 1-ethylpiperidine (22.6 mg, 0.20 mmol), tosyl chloride (114.4 mg, 0.60 mmol), and 1,4-dioxane (0.1 M, 2 mL) for 60 h. The product was purified by column chromatography (aluminum oxide, gradient 18 to 20% EA/*n*-hexane). Yield = 29.7 mg (56%).

^{1}H NMR (400 MHz, DMSO-*d*$_6$): δ 7.59 (d, *J* = 8.2 Hz, 2H), 7.33 (d, *J* = 8.1 Hz, 2H), 7.28 (s, 1H), 3.23 (q, *J* = 7.1 Hz, 2H), 3.06 – 2.95 (m, 2H), 2.35 (s, 3H), 1.98 (t, *J* = 6.1 Hz, 2H), 1.65 (p, *J* = 6.0 Hz, 2H), 1.07 (t, *J* = 7.1 Hz, 3H); ^{13}C NMR (101 MHz, DMSO-*d*$_6$): δ 143.75, 141.80, 140.34, 129.48, 126.14, 98.12, 49.45, 44.04, 20.91, 20.54, 19.45, 13.68; GCMS (EI) *m/z* calc. for $C_{14}H_{19}NO_2S$ [M$^+$] 265.1, found 265.1, 250.1, 200.1, 186.1, 174.1, 158.1, 139.0, 126.1, 109.1, 91.1; HRMS (ESI-TOF) m/z: (M+Na)$^+$ calcd. for $C_{14}H_{19}NO_2SNa$, 288.1029; found, 288.1030; FT-IR ν_{max}(ATR) cm^{-1}: 2921, 2851, 1613, 1493, 1438, 1378, 1312, 1274, 1263, 1133, 1090, 1076, 1060, 1011, 817, 810, 798, 683.

1-(1-Phenylethyl)-6-tosyl-2,3,4,5-tetrahydro-1*H*-azepine (37)

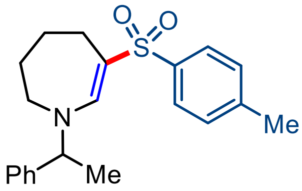

The title compound was synthesized according to the general procedure employing $Pd(OAc)_2$ (2.3 mg, 0.01 mmol), *rac*-BINAP (12.5 mg, 0.02 mmol), K_2CO_3 (82.8 mg, 0.60 mmol), 1-(1-phenylethyl)azepane (40.6 mg, 0.20 mmol), tosyl chloride (114.4 mg, 0.60 mmol), and 1,4-dioxane (0.1 M, 2 mL) for 60 h. The product was purified by column chromatography (aluminum oxide, gradient 20 to 22% EA/*n*-hexane). Yield = 60.4 mg (85%).

^{1}H NMR (400 MHz, DMSO-d_6): δ 7.59 (d, J = 8.2 Hz, 2H), 7.54 (s, 1H), 7.45 – 7.24 (m, 7H), 4.69 (q, J = 6.9 Hz, 1H), 3.16 – 3.00 (m, 2H), 2.36 (s, 3H), 2.24 – 2.11 (m, 2H), 1.63 – 1.40 (m, 7H); ^{13}C NMR (101 MHz, DMSO-d_6): δ 146.73, 142.04, 141.41, 139.93, 129.51, 128.54, 127.47, 126.67, 126.44, 104.45, 63.46, 48.56, 27.68, 26.07, 24.91, 20.93, 18.47; GCMS (EI) *m/z* calc. for $C_{21}H_{25}NO_2S$ [M^+] 355.2, found 355.2, 340.2, 251.1, 199.1, 184.1, 139.0, 105.1, 91.1, 79.1, 67.1; HRMS (ESI-TOF) m/z: $(M+Na)^+$ calcd. for $C_{21}H_{25}NO_2SNa$, 378.1498; found, 378.1499; FT-IR ν_{max}(ATR) cm^{-1}: 2933, 2856, 1613, 1493, 1448, 1398, 1373, 1297, 1271, 1178, 1140, 1091, 1077, 1038, 928, 909, 826.

1-(1-(Naphthalen-1-yl)ethyl)-6-tosyl-2,3,4,5-tetrahydro-1*H*-azepine (38)

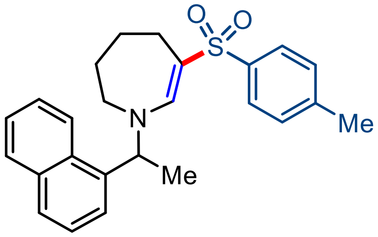

The title compound was synthesized according to the general procedure employing $Pd(OAc)_2$ (2.3 mg, 0.01 mmol), *rac*-BINAP (12.5 mg, 0.02 mmol), K_2CO_3 (82.8 mg, 0.60 mmol), 1-(1-(naphthalen-1-yl)ethyl)azepane (50.6 mg, 0.20 mmol), tosyl chloride (114.4 mg, 0.60 mmol), and 1,4-dioxane (0.1 M, 2 mL) for 60 h. The product was purified by column chromatography (aluminum oxide, gradient 20 to 23% EA/*n*-hexane). Yield = 64.9 mg (80%).

^{1}H NMR (400 MHz, DMSO-d_6): δ 8.12 – 8.05 (m, 1H), 8.04 – 7.96 (m, 1H), 7.93 (d, J = 7.7 Hz, 1H), 7.60-7.53 (m, 5H), 7.42 (d, J = 8.1 Hz, 2H), 7.28 (d, J = 8.1 Hz, 2H), 5.43 (q, J = 6.7 Hz, 1H), 3.35 – 3.28 (m, 1H), 3.13-3.07 (m, 1H), 2.35 (s, 3H), 2.10-2.06 (m, 2H), 1.67 (d, J = 6.7 Hz, 3H), 1.45-1.40 (m, 2H), 1.35-1.31 (m, 1H), 1.23-1.18 (m, 1H); ^{13}C NMR (101 MHz, DMSO-d_6): δ 146.51, 141.90, 139.97, 135.99, 133.58, 130.96, 129.38, 128.76, 128.60, 126.27, 126.23, 125.83, 125.28, 124.34, 123.63, 104.49, 59.62, 48.44, 27.59, 25.79, 24.72, 20.90, 17.97; GCMS (EI) *m/z* calc. for $C_{25}H_{27}NO_2S$ [M^+] 405.2, found 405.2, 390.1, 251.1, 155.1, 139.0, 129.1, 115.0, 95.1; HRMS (ESI-TOF) m/z: $(M+Na)^+$ calcd. for $C_{25}H_{27}NO_2SNa$, 428.1655; found, 428.1663; FT-IR ν_{max}(ATR) cm^{-1}: 2922, 2850, 1732, 1611, 1509, 1412, 1372, 1275, 1236, 1169, 1127, 1080, 1044, 1008, 939, 800, 778.

1-(1-Phenylethyl)-5-tosyl-1,2,3,4-tetrahydropyridine (39)

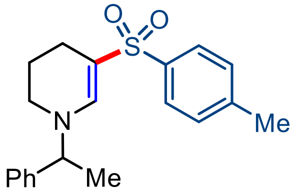

The title compound was synthesized according to the general procedure employing $Pd(OAc)_2$ (2.3 mg, 0.01 mmol), *rac*-BINAP (12.5 mg, 0.02 mmol), K_2CO_3 (82.8 mg, 0.60 mmol), 1-(1-phenylethyl)piperidine (37.9 mg, 0.20 mmol), tosyl chloride (114.4 mg, 0.60 mmol), and 1,4-dioxane (0.1 M, 2 mL) for 60 h. The products were purified by column chromatography (aluminum oxide, gradient 18 to 20% EA/*n*-hexane). Yield = 49.1 mg (**39**, 72%) and 7.5 mg (**39'**, 11%).

^{1}H NMR (400 MHz, DMSO-d_6): δ 7.62 (d, J = 8.1 Hz, 2H), 7.53 (s, 1H), 7.36 (t, J = 7.1 Hz, 4H), 7.32 – 7.24 (m, 3H), 4.68 (q, J = 6.9 Hz, 1H), 2.99-2.89 (m, 1H), 2.83-2.78 (m, 1H), 2.36 (s, 3H), 1.98 (t, J = 6.0 Hz, 2H), 1.67 – 1.40 (m, 5H); ^{13}C NMR (101 MHz, DMSO-d_6): δ 142.44, 141.93, 141.09, 140.15, 129.52, 128.55, 127.45, 126.51, 126.21, 99.36, 61.36, 42.36, 20.91, 20.46, 19.88, 18.17; GCMS (EI) *m/z* calc. for $C_{20}H_{23}NO_2S$ [M$^+$] 341.1, found 341.1, 326.1, 237.1, 172.1, 139.0, 105.1, 81.1, 65.0; HRMS (ESI-TOF) m/z: $(M+Na)^+$ calcd. for $C_{20}H_{23}NO_2SNa$, 364.1342; found, 364.1367; FT-IR ν_{max}(ATR) cm^{-1}: 2928, 2852, 1615, 1489, 1449, 1424, 1376, 1350, 1268, 1214, 1125, 1093, 1080, 1062, 985, 816, 798, 776.

(*E*)-1-(1-Phenyl-2-tosylvinyl)piperidine (39')

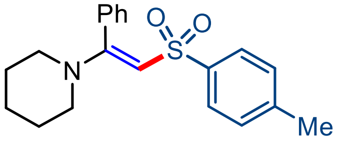

^{1}H NMR (400 MHz, DMSO-d_6): δ 7.40 (t, J = 7.7 Hz, 1H), 7.30 (t, J = 7.5 Hz, 2H), 7.26 – 7.16 (m, 4H), 6.94 (d, J = 7.1 Hz, 2H), 5.51 (s, 1H), 3.00 (s, 4H), 2.33 (s, 3H), 1.53 (q, J = 6.4, 5.9 Hz, 2H), 1.42 (s, 4H); ^{13}C NMR (101 MHz, DMSO-d_6): δ 159.71, 142.94, 141.49, 133.23, 128.99, 128.93, 128.89, 127.74, 126.00, 97.47, 48.15, 24.95, 23.64, 20.93; GCMS (EI) *m/z* calc. for $C_{20}H_{23}NO_2S$ [M$^+$] 341.1, found 341.1, 276.1, 241.0, 216.1, 186.1, 178.1, 156.1, 130.0, 103.0; HRMS (ESI-TOF) m/z: $(M+Na)^+$ calcd. for $C_{20}H_{23}NO_2SNa$, 364.1342; found, 364.1369; FT-IR ν_{max}(ATR) cm^{-1}: 2919, 2849, 1592, 1513, 1493, 1442, 1399, 1310, 1295, 1284, 1227, 1139, 1121, 1081, 1017, 951, 818, 797.

1-(1-Phenylethyl)-4-tosyl-2,3-dihydro-1*H*-pyrrole (40)

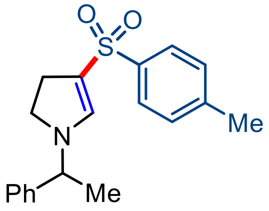

The title compound was synthesized according to the general procedure employing $Pd(OAc)_2$ (2.3 mg, 0.01 mmol), *rac*-BINAP (12.5 mg, 0.02 mmol), K_2CO_3 (82.8 mg, 0.60 mmol), 1-(1-phenylethyl)pyrrolidine (35.0 mg, 0.20 mmol), tosyl chloride (114.4 mg, 0.60 mmol), and 1,4-dioxane (0.1 M, 2 mL) for 60 h. The products were purified by column chromatography (aluminum oxide, gradient 18 to 20% EA/*n*-hexane). Yield = 9.2 mg (**40**, 14%) and 46.5 mg (**40',** 71%).

^{1}H NMR (400 MHz, DMSO-d_6): δ 7.61 (d, J = 8.1 Hz, 2H), 7.40 – 7.32 (m, 5H), 7.29 (dd, J = 7.4, 4.5 Hz, 3H), 4.47 (q, J = 7.0 Hz, 1H), 3.29 (t, J = 10.1 Hz, 2H), 2.45 (t, J = 10.3 Hz, 2H), 2.37 (s, 3H), 1.48 (d, J = 7.0 Hz, 3H); ^{13}C NMR (101 MHz, DMSO-d_6): δ 149.39, 142.20, 141.17, 139.99, 129.63, 128.61, 127.58, 126.68, 126.03, 104.80, 57.16, 49.47, 26.70, 20.95, 19.47; GCMS (EI)

m/z calc. for $C_{19}H_{21}NO_2S$ [M^+] 327.1, found 327.1, 312.1, 223.1, 158.1, 105.1, 91.1, 79.1, 67.0, 51.0; HRMS (ESI-TOF) m/z: $(M+Na)^+$ calcd. for $C_{19}H_{21}NO_2SNa$, 350.1185; found, 350.1188; FT-IR ν_{max}(ATR) cm^{-1}: 2918, 2849, 1569, 1493, 1452, 1377, 1283, 1181, 1134, 1085, 1033, 1017, 980, 917, 813, 701, 661.

(*E*)-1-(1-Phenyl-2-tosylvinyl)pyrrolidine (40')

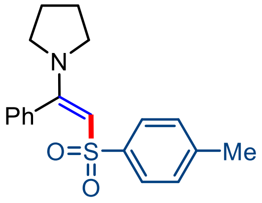

^1H NMR (400 MHz, DMSO-d_6): δ 7.38 (t, *J* = 7.4 Hz, 1H), 7.30 (t, *J* = 7.4 Hz, 2H), 7.20 (q, *J* = 8.2 Hz, 4H), 6.94 (d, *J* = 7.1 Hz, 2H), 5.12 (s, 1H), 3.18 (broad, 2H), 2.76 (broad, 2H), 2.32 (s, 3H), 1.89 (broad, 2H), 1.68 (broad, 2H); ^{13}C NMR (101 MHz, DMSO-d_6): δ 157.64, 143.50, 141.20, 133.81, 128.84, 128.62, 128.22, 127.68, 125.88, 94.16, 49.42, 47.90, 24.70, 20.89; GCMS (EI) *m/z* calc. for $C_{19}H_{21}NO_2S$ [M^+] 327.1, found 327.1, 262.1, 172.1, 143.1, 130.1, 116.0, 102.0, 89.0, 70.1; HRMS (ESI-TOF) m/z: $(M+Na)^+$ calcd. for $C_{19}H_{21}NO_2SNa$, 350.1185; found, 350.1192; FT-IR ν_{max}(ATR) cm^{-1}: 2941, 2866, 1536, 1496, 1457, 1441, 1348, 1314, 1281, 1228, 1131, 1081, 1018, 1001, 940, 913, 823.

(*E*)-*N*-Ethyl-*N*-(1-phenylethyl)-2-tosylethen-1-amine (41)

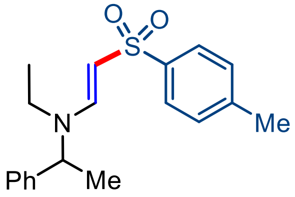

The title compound was synthesized according to the general procedure employing $Pd(OAc)_2$ (2.3 mg, 0.01 mmol), *rac*-BINAP (12.5 mg, 0.02 mmol), K_2CO_3 (82.8 mg, 0.60 mmol), *N*,*N*-diethyl-1-phenylethan-1-amine (35.4 mg, 0.20 mmol), tosyl chloride (114.4 mg, 0.60 mmol), and 1,4-dioxane (0.1 M, 2 mL) for 60 h. The product was purified by column chromatography (aluminum oxide, gradient 15 to 18% EA/*n*-hexane). Yield = 27.6 mg (**41**, 42%) and 20.4 mg (**41',** 31%).

^1H NMR (400 MHz, DMSO-d_6): δ 7.64 (d, *J* = 8.1 Hz, 2H), 7.45 (d, *J* = 12.5 Hz, 1H), 7.41 – 7.14 (m, 7H), 5.16 (d, *J* = 12.5 Hz, 1H), 4.79 (q, *J* = 6.8 Hz, 1H), 3.05 (broad, 2H), 2.36 (s, 3H), 1.53 (d, *J* = 6.8 Hz, 3H), 0.87 (s, 3H); ^{13}C NMR (101 MHz, DMSO-d_6): δ 147.36, 142.95, 141.56, 141.17, 129.43, 128.57, 127.60, 126.75, 125.53, 91.95, 61.75, 41.33, 20.89, 19.14, 11.45; GCMS (EI) *m/z* calc. for $C_{19}H_{23}NO_2S$ [M^+] 329.1, found 329.1, 236.1, 174.1, 158.1, 144.1, 132.1, 117.1, 104.1, 91.1, 72.1; HRMS (ESI-TOF) m/z: $(M+Na)^+$ calcd. for $C_{19}H_{23}NO_2SNa$, 352.1342; found, 352.1346; FT-IR ν_{max}(ATR) cm^{-1}: 2976, 1608, 1493, 1449, 1419, 1379, 1306, 1279, 1241, 1131, 1105, 1078, 1017, 912, 757, 733.

(*E*)-*N*,*N*-Diethyl-1-phenyl-2-tosylethen-1-amine (41')

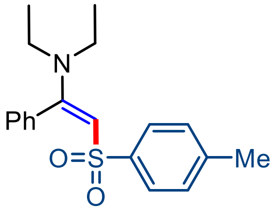

^1H NMR (400 MHz, DMSO-d_6): δ 7.38 (t, *J* = 7.4 Hz, 1H), 7.28 (t, *J* = 7.5 Hz, 2H), 7.19 (s, 4H), 6.88 (d, *J* = 7.1 Hz, 2H), 5.30 (s, 1H), 3.00 (broad, 4H), 2.33 (s, 3H), 0.97 (broad, 6H); ^{13}C NMR (101 MHz, DMSO-d_6): δ 158.72, 143.28, 141.25, 132.67, 128.84, 128.81, 128.72, 127.55, 125.90, 94.80, 43.50, 20.91, 12.34; GCMS (EI) *m/z* calc. for $C_{19}H_{23}NO_2S$ [M^+] 329.1, found 329.1, 300.1, 210.0, 174.1, 158.1, 144.1, 130.1, 119.1, 105.1,

91.1, 79.1; HRMS (ESI-TOF) m/z: $(M+Na)^+$ calcd. for $C_{19}H_{23}NO_2SNa$, 352.1342; found, 352.1349; FT-IR ν_{max}(ATR) cm^{-1}: 2973, 2932, 1533, 1494, 1443, 1427, 1379, 1279, 1254, 1199, 1128, 1076, 1028, 944, 812, 778.

1-Ethyl-1*H*-indole (42)

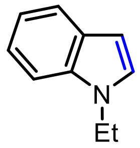

The title compound was synthesized according to the general procedure employing $Pd(OAc)_2$ (2.3 mg, 0.01 mmol), PPh_3 (10.5 mg, 0.04 mmol), K_2CO_3 (82.8 mg, 0.60 mmol), 1-ethylindoline (29.4 mg, 0.20 mmol), tosyl chloride (114.4 mg, 0.60 mmol), and 1,4-dioxane (0.1 M, 2 mL) for 24 h. The product was purified by column chromatography (silica gel, gradient 5 to 8% EA/*n*-hexane). Yield = 20.9 mg (72%).

^1H NMR (400 MHz, $CDCl_3$): δ 7.65 (d, *J* = 7.9 Hz, 1H), 7.37 (d, *J* = 8.2 Hz, 1H), 7.22 (t, *J* = 7.6 Hz, 1H), 7.14 – 7.07 (m, 2H), 6.51 (d, *J* = 3.0 Hz, 1H), 4.19 (q, *J* = 7.3 Hz, 2H), 1.48 (t, *J* = 7.3 Hz, 3H); ^{13}C NMR (101 MHz, $CDCl_3$): δ 135.81, 128.75, 127.11, 121.44, 121.09, 119.32, 109.38, 101.12, 41.08, 15.60; GCMS (EI) *m/z* calc. for $C_{10}H_{11}N$ [M^+] 145.1, found 145.1, 130.1, 116.0, 103.0, 89.0, 77.0, 63.0, 51.0, 39.0; FT-IR ν_{max}(ATR) cm^{-1}: 2919, 1505, 1489, 1457, 1343, 1313, 1224, 1168, 1154, 1083, 1062, 1012, 802, 759. Data are consistent with those reported in the literature.[12]

1-Ethyl-5-methyl-1*H*-indole (43)

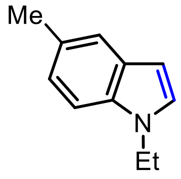

The title compound was synthesized according to the general procedure employing $Pd(OAc)_2$ (2.3 mg, 0.01 mmol), PPh_3 (10.5 mg, 0.04 mmol), K_2CO_3 (82.8 mg, 0.60 mmol), 1-ethyl-5-methylindoline (32.2 mg, 0.20 mmol), tosyl chloride (114.4 mg, 0.60 mmol), and 1,4-dioxane (0.1 M, 2 mL) for 24 h. The product was purified by column chromatography (silica gel, gradient 5 to 8% EA/*n*-hexane). Yield = 22.2 mg (70%).

^1H NMR (400 MHz, $CDCl_3$): δ 7.48 (s, 1H), 7.30 (d, *J* = 7.8 Hz, 1H), 7.13-7.06 (m, 2H), 6.47 (s, 1H), 4.19 (q, *J* = 7.3 Hz, 2H), 2.52 (s, 3H), 1.49 (t, *J* = 7.3 Hz, 3H); ^{13}C NMR (101 MHz, $CDCl_3$): δ 134.20, 129.02, 128.46, 127.15, 123.05, 120.70, 109.06, 100.50, 41.08, 21.51, 15.58; GCMS (EI) *m/z* calc. for $C_{11}H_{13}N$ [M^+] 159.1, found 159.1, 144.1, 130.1, 115.1, 103.0, 91.1, 77.0, 63.0; FT-IR ν_{max}(ATR) cm^{-1}: 3039, 1606, 1592, 1491, 1443, 1398, 1335, 1299, 1150, 1134, 1083, 1031, 1017, 913, 727. Data are consistent with those reported in the literature.[13]

1-Ethyl-5-methoxy-1*H*-indole (44)

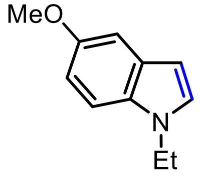

The title compound was synthesized according to the general procedure employing $Pd(OAc)_2$ (2.3 mg, 0.01 mmol), PPh_3 (10.5 mg, 0.04 mmol), K_2CO_3 (82.8 mg, 0.60 mmol), 1-ethyl-5-methoxyindoline (35.4 mg, 0.20 mmol), tosyl chloride (114.4 mg, 0.60 mmol), and 1,4-dioxane (0.1 M, 2 mL) for 24 h. The product was purified by column chromatography (silica gel, gradient 10 to 12% EA/*n*-hexane). Yield = 25.9 mg (74%).

^{1}H NMR (400 MHz, $CDCl_3$): δ 7.29 (d, *J* = 8.9 Hz, 1H), 7.15 (dd, *J* = 11.7, 2.5 Hz, 2H), 6.94 (dd, *J* = 8.8, 2.0 Hz, 1H), 6.47 (d, *J* = 2.9 Hz, 1H), 4.16 (q, *J* = 7.3 Hz, 2H), 3.90 (s, 3H), 1.49 (t, *J* = 7.3 Hz, 3H); ^{13}C NMR (101 MHz, $CDCl_3$): δ 154.00, 131.16, 129.02, 127.57, 111.81, 110.07, 102.65, 100.59, 55.96, 41.17, 15.58; GCMS (EI) *m/z* calc. for $C_{11}H_{13}NO$ [M^+] 175.1, found 175.1, 160.1, 146.0, 132.1, 117.1, 104.1, 89.0, 77.0, 63.0; FT-IR ν_{max}(ATR) cm^{-1}: 2987, 2954, 1505, 1491, 1446, 1435, 1406, 1377, 1236, 1224, 1192, 1149, 1027, 844, 795, 756. Data are consistent with those reported in the literature.[12]

1-Ethyl-1*H*-indole-5-carbonitrile (45)

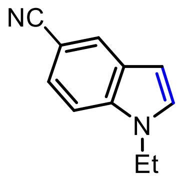

The title compound was synthesized according to the general procedure employing $Pd(OAc)_2$ (2.3 mg, 0.01 mmol), PPh_3 (10.5 mg, 0.04 mmol), K_2CO_3 (82.8 mg, 0.60 mmol), 1-ethylindoline-5-carbonitrile (34.4 mg, 0.20 mmol), tosyl chloride (114.4 mg, 0.60 mmol), and 1,4-dioxane (0.1 M, 2 mL) for 24 h. The product was purified by column chromatography (silica gel, gradient 10 to 14% EA/*n*-hexane). Yield = 20.7 mg (61%).

^{1}H NMR (400 MHz, $CDCl_3$): δ 7.94 (s, 1H), 7.39 (q, *J* = 8.6 Hz, 2H), 7.24 (d, *J* = 3.2 Hz, 1H), 6.56 (d, *J* = 3.2 Hz, 1H), 4.19 (q, *J* = 7.3 Hz, 2H), 1.47 (t, *J* = 7.3 Hz, 3H); ^{13}C NMR (101 MHz, $CDCl_3$): δ 137.22, 129.45, 128.31, 126.54, 124.25, 120.97, 110.14, 102.26, 102.17, 41.31, 15.42; GCMS (EI) *m/z* calc. for $C_{11}H_{10}N_2$ [M^+] 170.1, found 170.1, 155.1, 142.0, 128.0, 114.0, 101.0, 88.0, 75.0, 63.1; FT-IR ν_{max}(ATR) cm^{-1}: 3102, 2217, 1607, 1508, 1449, 1400, 1363, 1337, 1291, 1222, 1153, 1111, 1028, 938, 893. Data are consistent with those reported in the literature.[14]

1-Phenyl-3-tosylpiperidine (46)

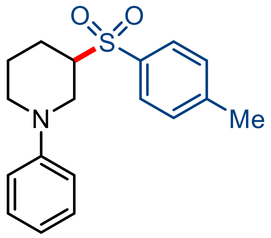

^{1}H NMR (400 MHz, $CDCl_3$): δ 7.78 (d, *J* = 7.8 Hz, 2H), 7.38 (d, *J* = 7.9 Hz, 2H), 7.27 – 7.19 (m, 2H), 6.87 (dd, *J* = 7.9, 3.8 Hz, 3H), 3.98 (d, *J* = 12.1 Hz, 1H), 3.63 (d, *J* = 12.4 Hz, 1H), 3.34 – 3.20 (m, 1H), 2.89 (t, *J* = 11.7 Hz, 1H), 2.67 (t, *J* = 10.9 Hz, 1H), 2.46 (s, 3H), 2.15 (d, *J* = 8.1 Hz, 1H), 1.84 (dd, *J* = 9.3, 2.9 Hz, 1H), 1.74 – 1.57 (m, 2H); ^{13}C NMR (101 MHz, $CDCl_3$): δ 150.53, 145.09, 134.33, 130.03, 129.41, 128.99, 120.34, 117.00, 60.66, 50.03, 49.19, 24.39, 23.94, 21.80; GCMS (EI) *m/z* calc. for $C_{18}H_{21}NO_2S$ [M^+] 315.1, found 315.2, 159.1, 144.1, 130.0, 118.0, 104.0, 91.0, 77.0, 65.0; HRMS (ESI-TOF) m/z: $(M+Na)^+$ calcd. for $C_{18}H_{21}NO_2SNa$, 338.1185; found, 338.1195; FT-IR ν_{max}(ATR) cm^{-1}: 2954, 1573, 1314, 1223, 1178, 1156, 1023, 989, 869, 802, 718, 689.

***N*-(4-Chloro-4-tosylbutyl)-*N*-phenylformamide (47)**

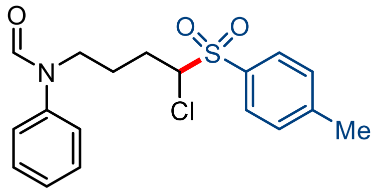

^{1}H NMR (400 MHz, $CDCl_3$): δ 8.36 (s, 1H), 7.81 (d, *J* = 8.1 Hz, 2H), 7.48 – 7.29 (m, 5H), 7.15 (d, *J* = 8.1 Hz, 2H), 4.76 (d, *J* = 10.5 Hz, 1H), 3.96 (dt, *J* = 14.2, 7.3 Hz, 1H), 3.85 – 3.62 (m, 1H), 2.47 (s, 3H), 2.39 – 2.32 (m, 1H), 1.93 – 1.81 (m, 2H), 1.79 – 1.68 (m, 1H); ^{13}C NMR (101 MHz, $CDCl_3$): δ 162.69, 145.91, 140.34,

132.19, 130.10, 130.00, 129.92, 127.37, 124.38, 74.03, 43.09, 27.67, 23.90, 21.89; GCMS (EI) *m/z* calc. for $C_{18}H_{20}ClNO_3S$ [M^+] 365.0, found 365.1, 336.0, 210.1, 148.1, 134.0, 121.0, 106.1, 91.1, 77.0; HRMS (ESI-TOF) m/z: $(M+Na)^+$ calcd. for $C_{18}H_{20}ClNO_3SNa$, 388.0745; found, 388.0757; FT-IR ν_{max}(ATR) cm^{-1}: 3390, 2924, 2250, 1665, 1446, 1377, 1285, 1236, 1149, 1027, 938, 844.

(2-Tosylethene-1,1-diyl)dibenzene

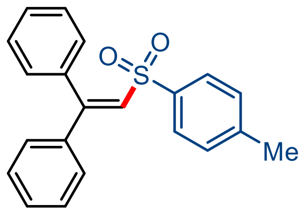

The title compound was synthesized according to the general procedure employing $Pd(OAc)_2$ (2.3 mg, 0.01 mmol), PPh_3 (10.5 mg, 0.04 mmol), K_2CO_3 (82.8 mg, 0.60 mmol), 1-phenylpyrrolidine (29.4 mg, 0.2 mmol, 1 equiv.), tosyl chloride (114.4 mg, 0.60 mmol), 1,1-diphenylethylene (108.0 mg, 0.6 mmol, 3 equiv.) and 1,4-dioxane (0.1 M, 2 mL) for 48 h. The product was purified by column chromatography (silica gel, gradient 12 to 15% EA/*n*-hexane). Yield = 40.7 mg (61%).

^1H NMR (400 MHz, $CDCl_3$): δ 7.49 (d, *J* = 8.2 Hz, 2H), 7.42 – 7.25 (m, 6H), 7.24 – 7.19 (m, 2H), 7.15 (d, *J* = 8.1 Hz, 2H), 7.11 (d, *J* = 7.2 Hz, 2H), 7.01 (s, 1H), 2.37 (s, 3H); ^{13}C NMR (101 MHz, $CDCl_3$): δ 154.70, 143.79, 139.16, 138.55, 135.55, 130.25, 129.75, 129.34, 128.92, 128.84, 128.57, 128.19, 127.80, 127.65, 21.57; GCMS (EI) *m/z* calc. for $C_{21}H_{18}O_2S$ [M^+] 334.1, found 334.1, 269.1, 254.1, 239.1, 195.1, 178.1, 167.1, 152.1, 139.0, 102.1, 91.1, 77.1; HRMS (ESI-TOF) m/z: $(M+Na)^+$ calcd. for $C_{21}H_{18}O_2SNa$, 357.0920; found, 357.0959; FT-IR ν_{max}(ATR) cm^{-1}: 2974, 2932, 1510, 1488, 1461, 1350, 1298, 1219, 1156, 1141, 1090, 945, 868, 787, 757, 711.

1-Phenyl-5-tosyl-1,2,3,4-tetrahydropyridine (4)

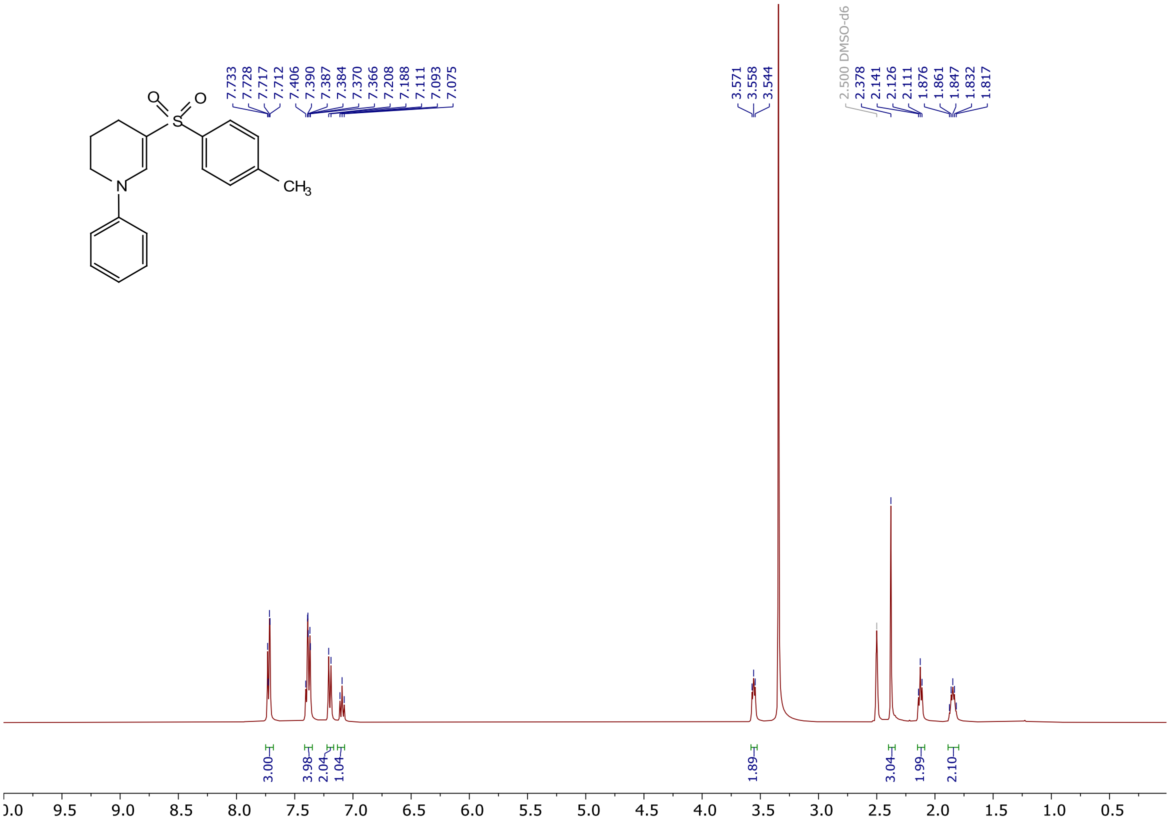

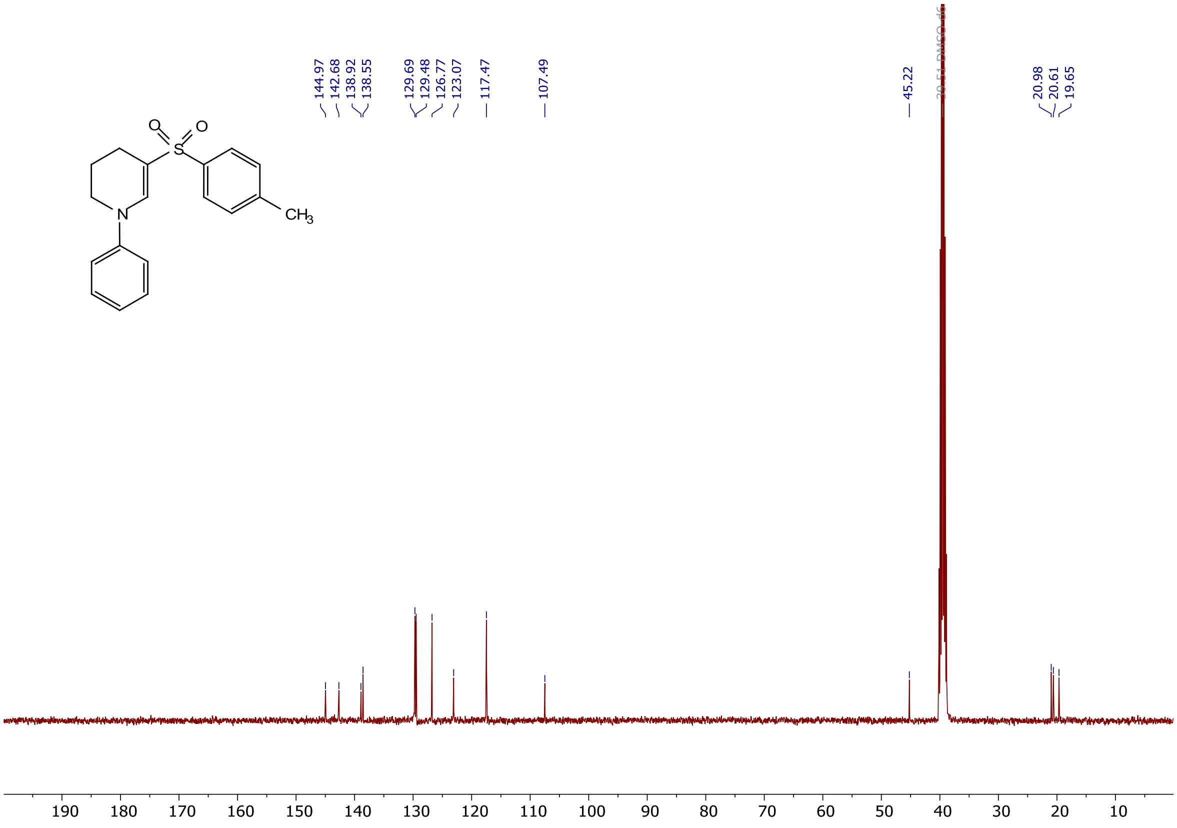

1-(*p*-Tolyl)-4-tosyl-2,3-dihydro-1*H*-pyrrole (5)

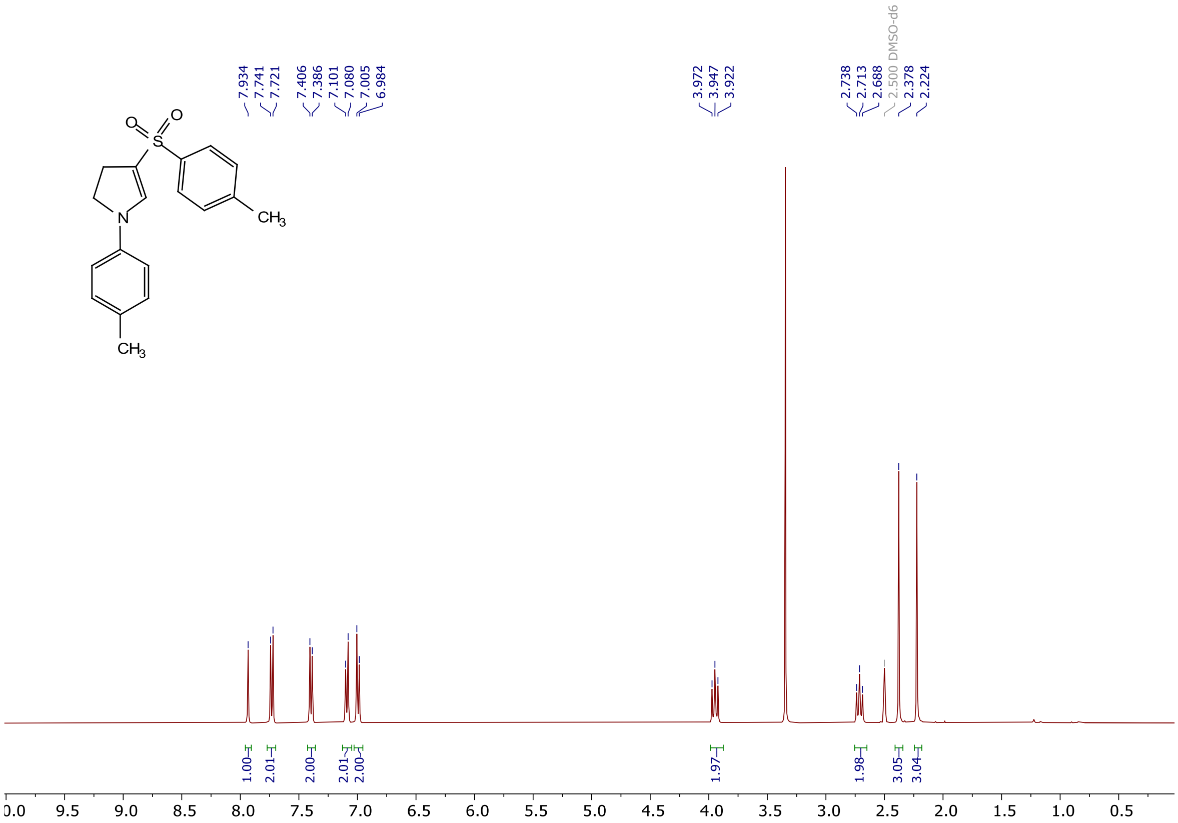

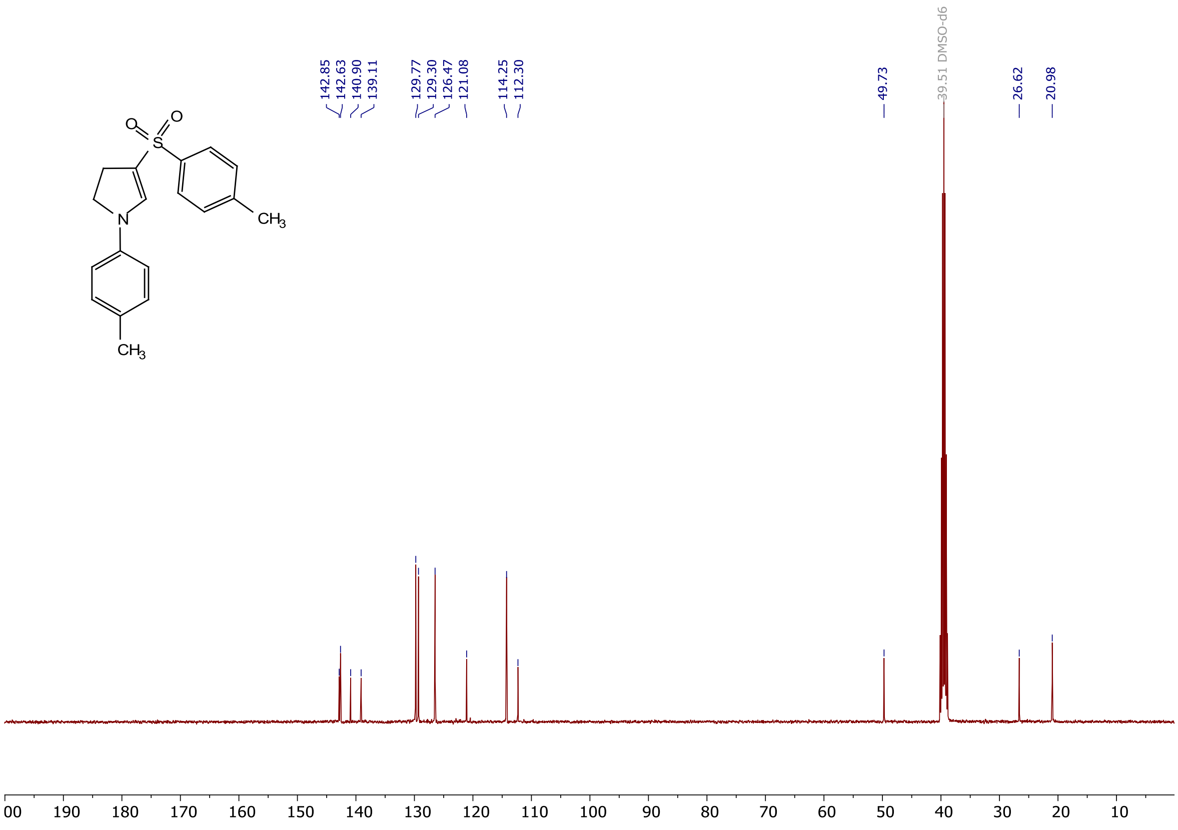

4-((4-(*tert*-Butyl)phenyl)sulfonyl)-1-(*p*-tolyl)-2,3-dihydro-1*H*-pyrrole (6)

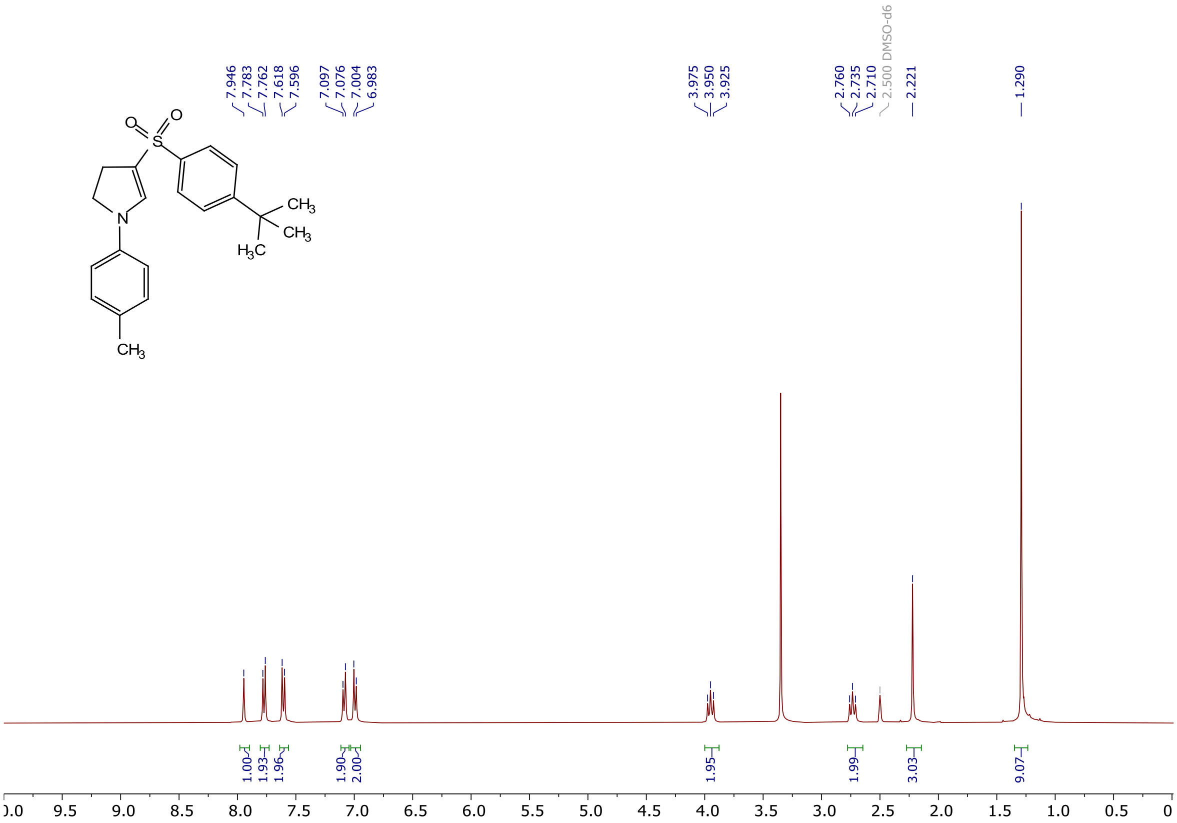

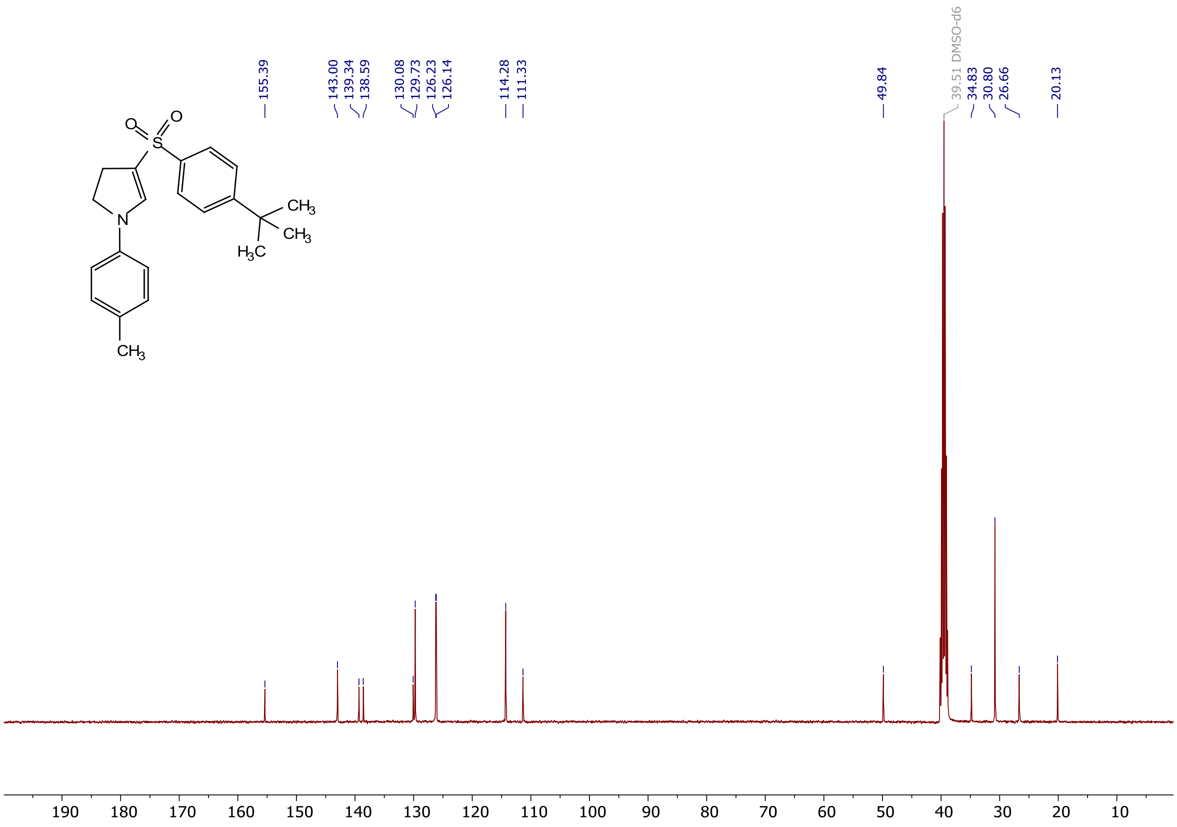

4-((4-Methoxyphenyl)sulfonyl)-1-(*p*-tolyl)-2,3-dihydro-1*H*-pyrrole (7)

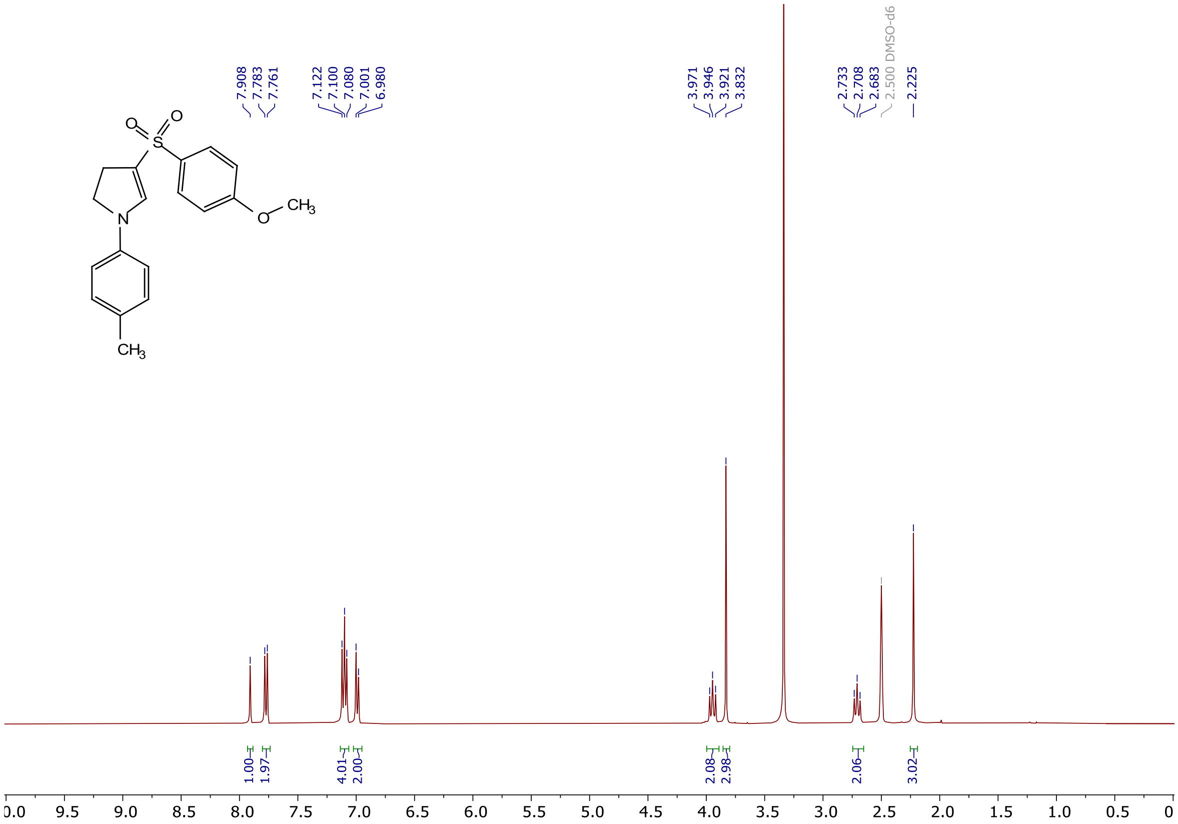

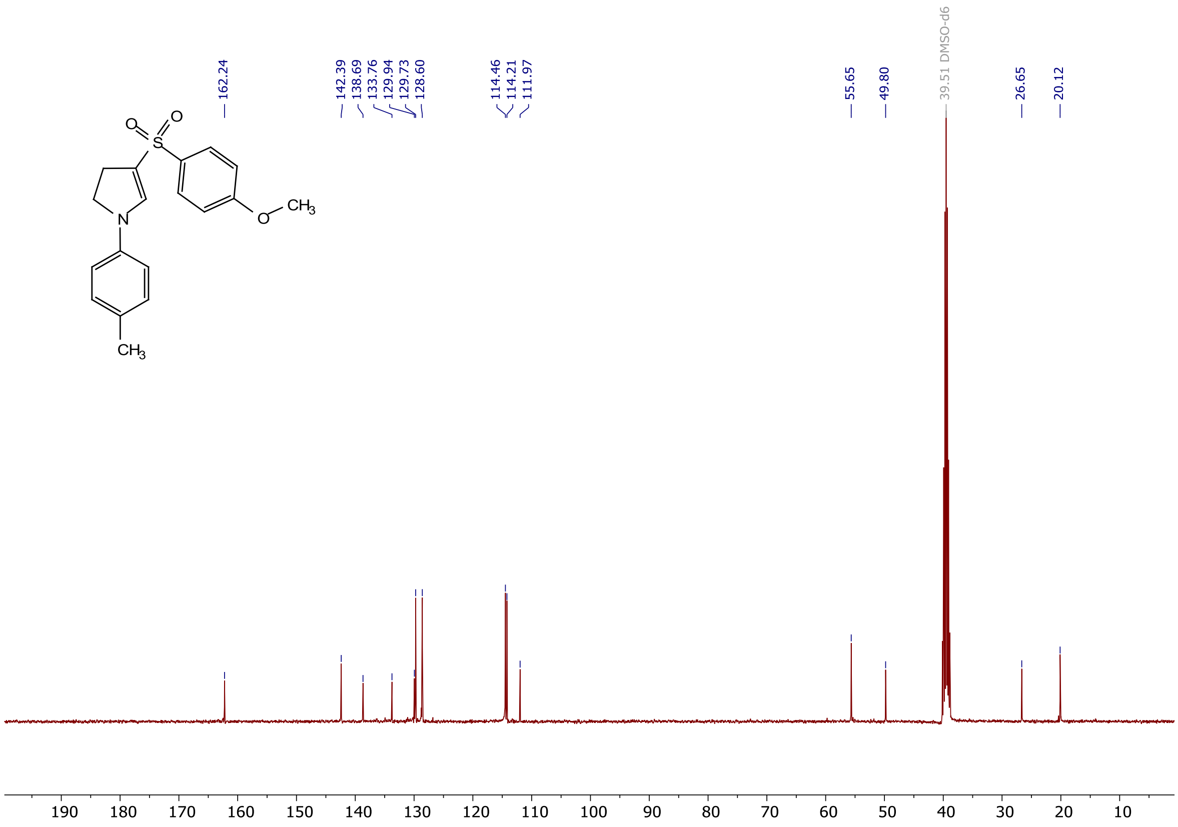

4-(Phenylsulfonyl)-1-(*p*-tolyl)-2,3-dihydro-1*H*-pyrrole (8)

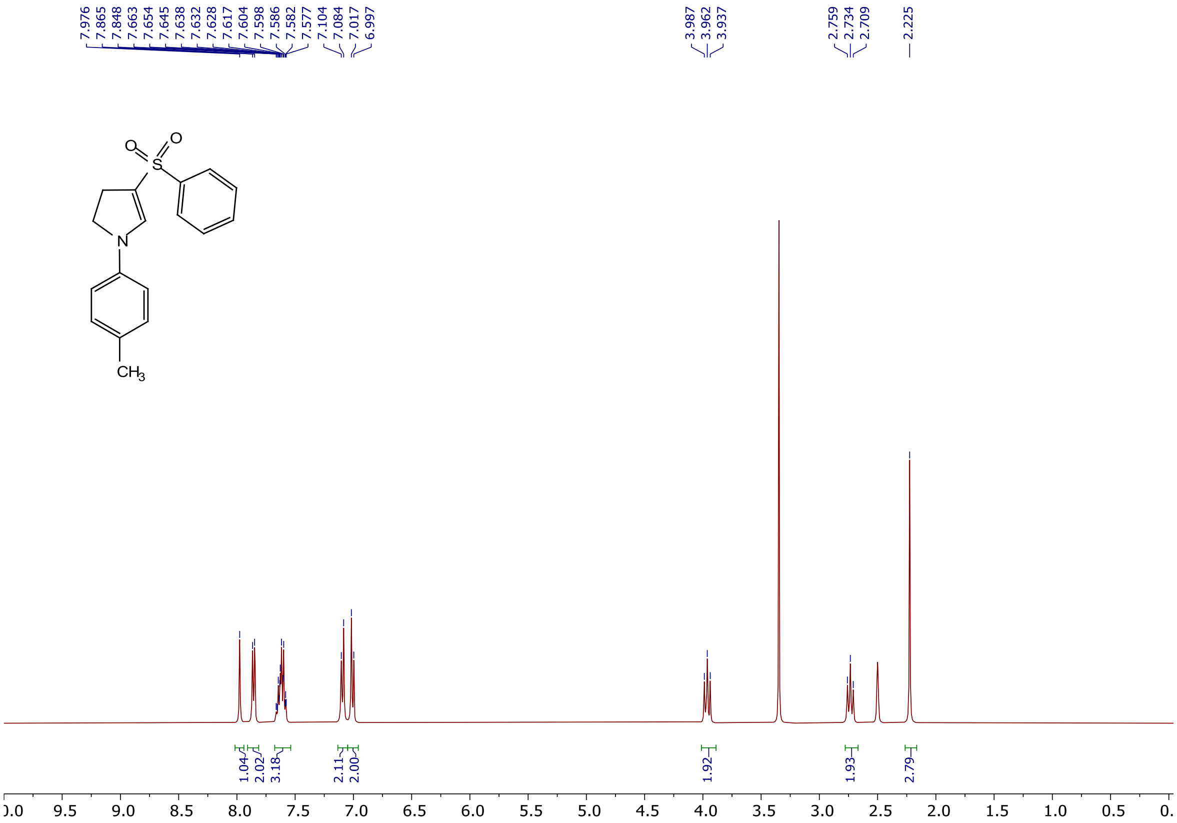

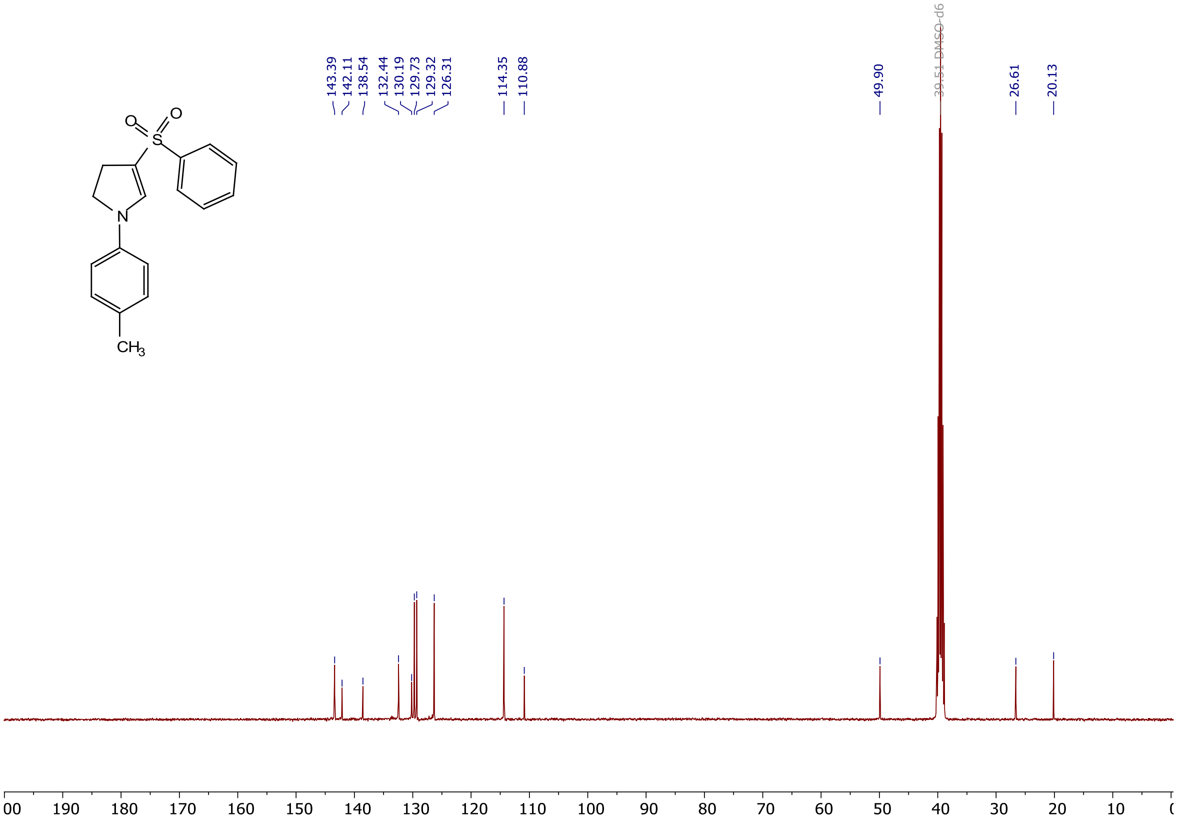

4-((4-Fluorophenyl)sulfonyl)-1-(*p*-tolyl)-2,3-dihydro-1*H*-pyrrole (9)

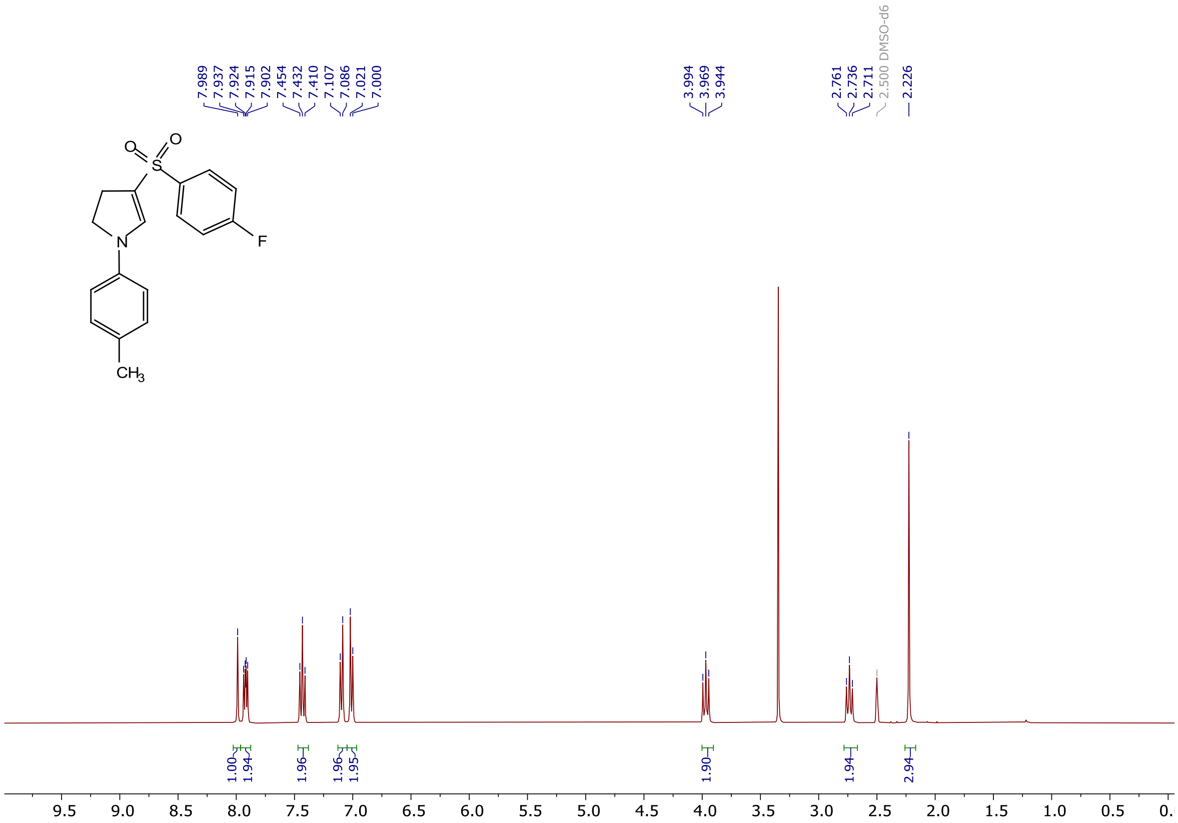

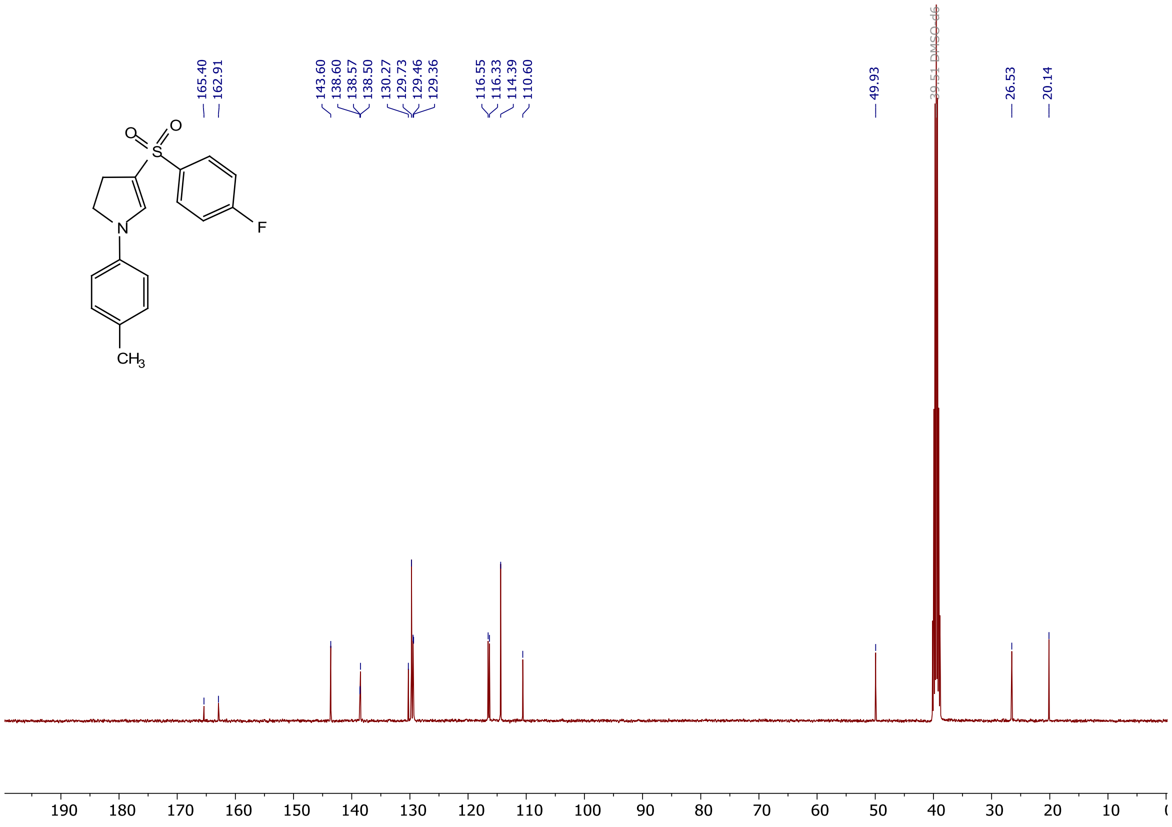

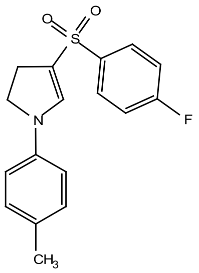
O
O
S
F
N
CH3

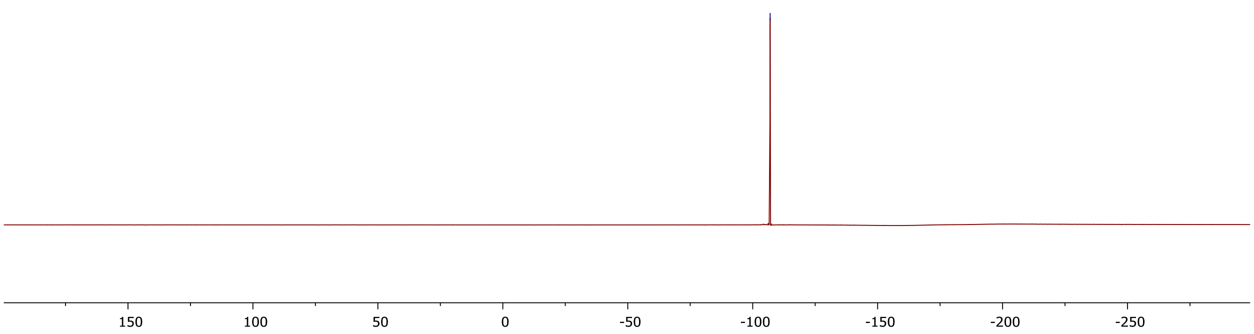
-106.98
150
100
50
0
-50
-100
-150
-200
-250

4-((4-Chlorophenyl)sulfonyl)-1-(*p*-tolyl)-2,3-dihydro-1*H*-pyrrole (10)

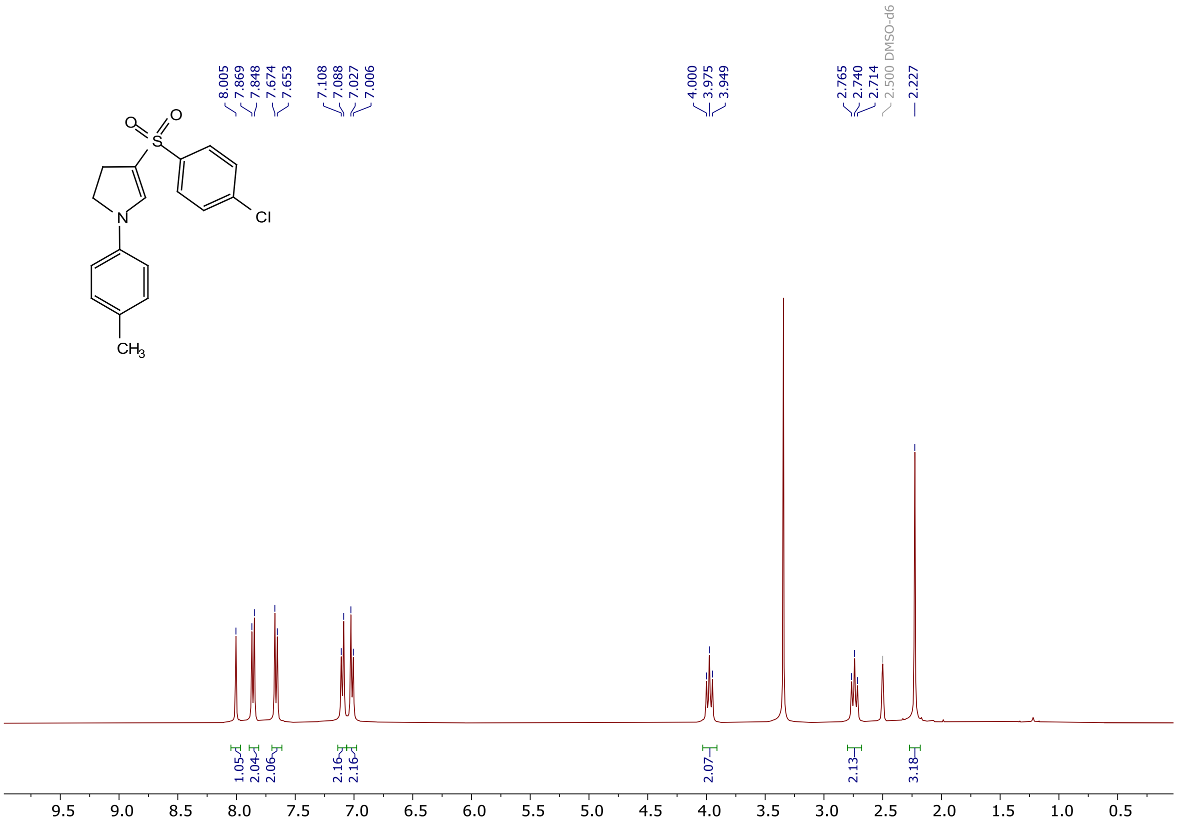

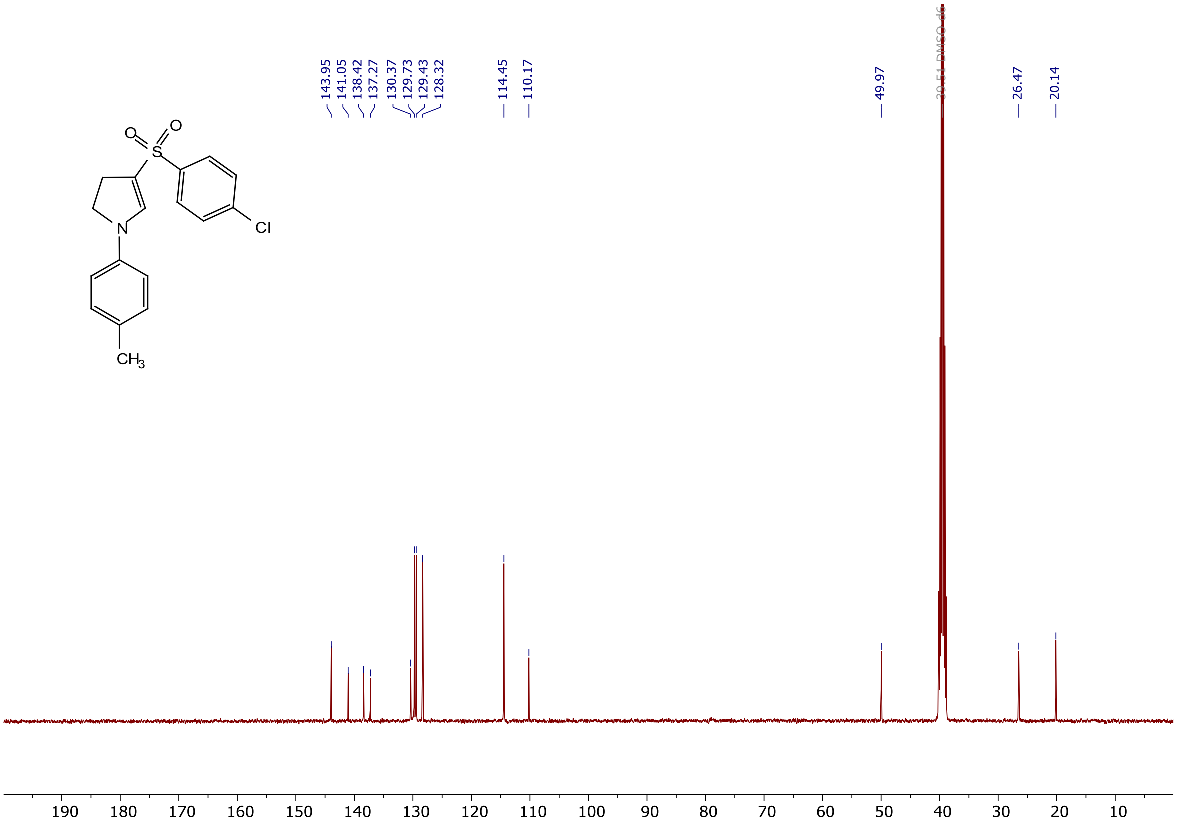

4-((4-Bromophenyl)sulfonyl)-1-(*p*-tolyl)-2,3-dihydro-1*H*-pyrrole (11)

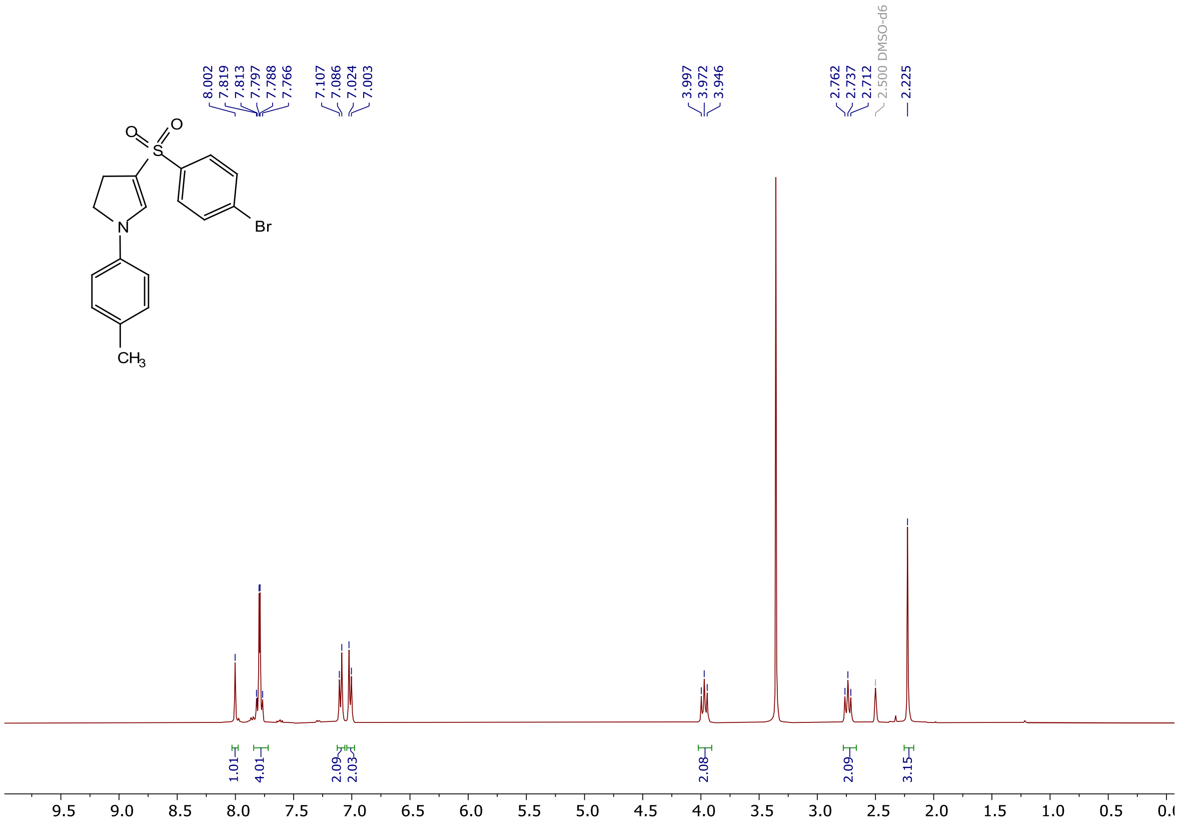

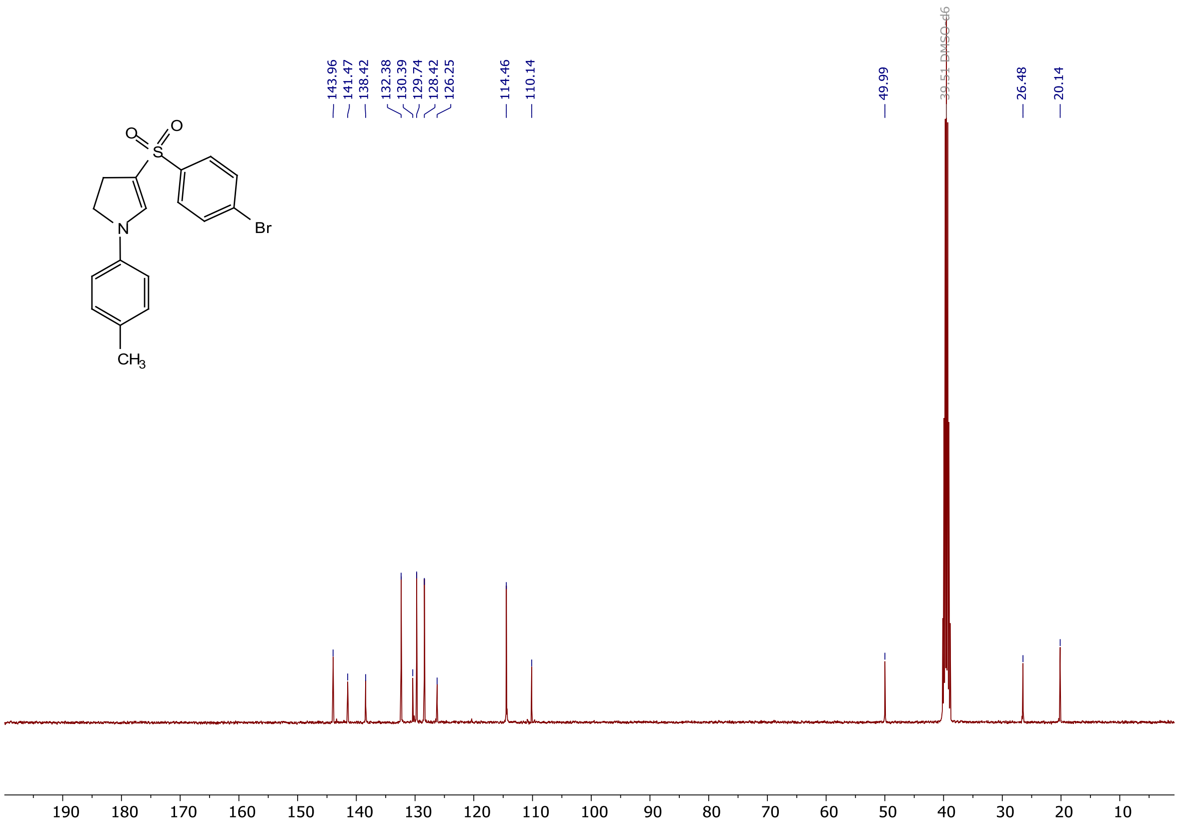

1-(*p*-Tolyl)-4-((4-(trifluoromethyl)phenyl)sulfonyl)-2,3-dihydro-1*H*-pyrrole (12)

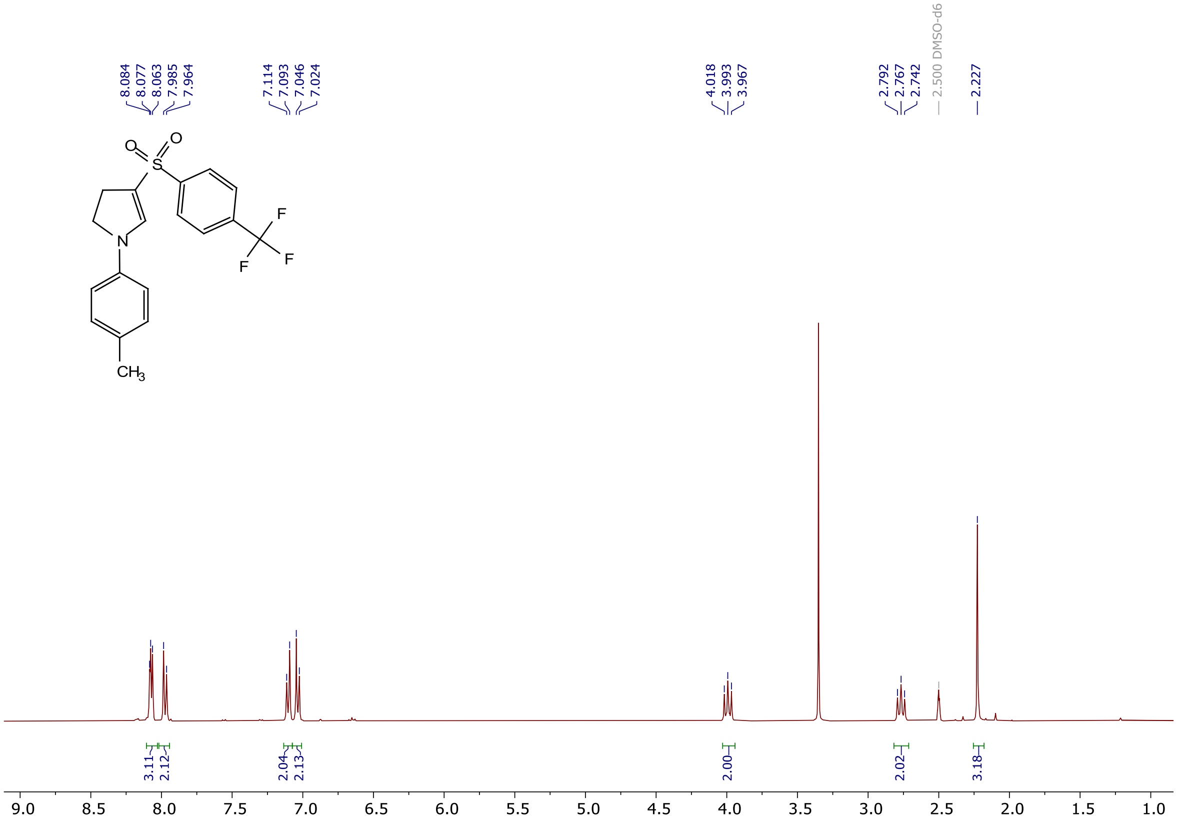

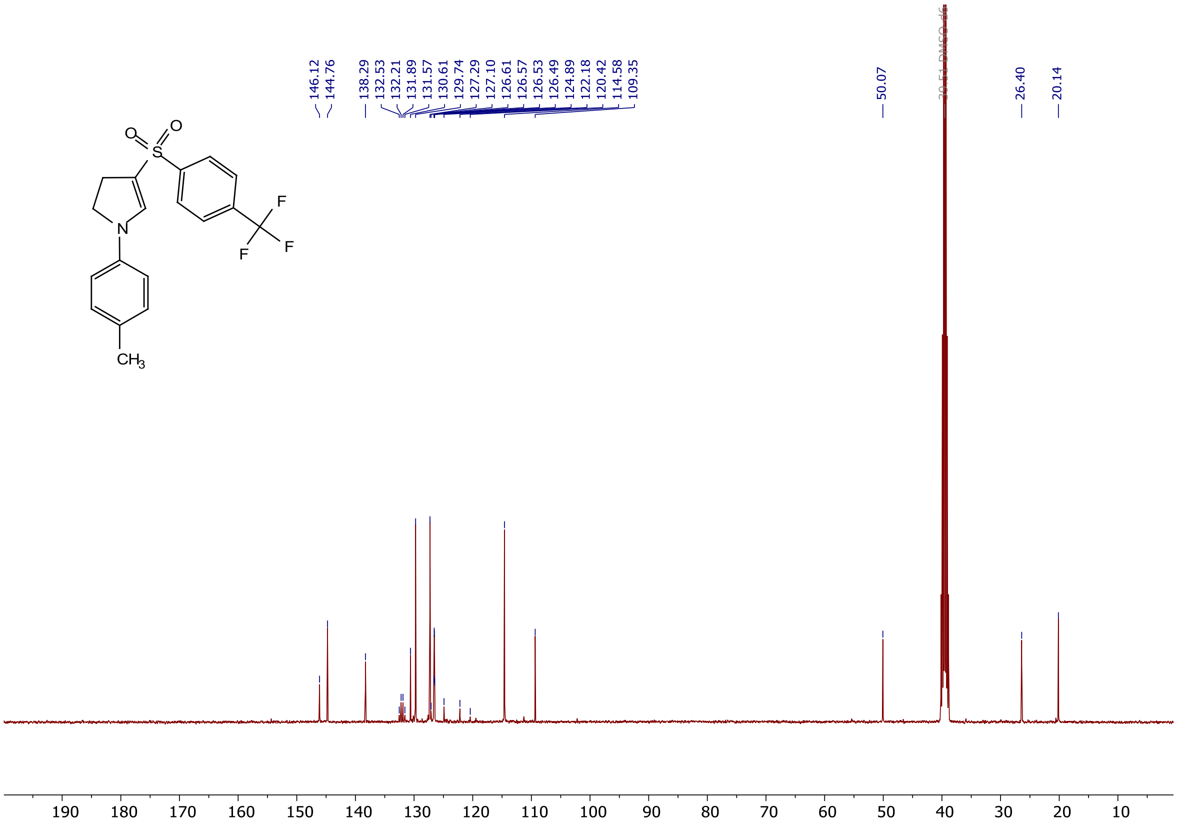

4-(Naphthalen-1-ylsulfonyl)-1-(*p*-tolyl)-2,3-dihydro-1*H*-pyrrole (13)

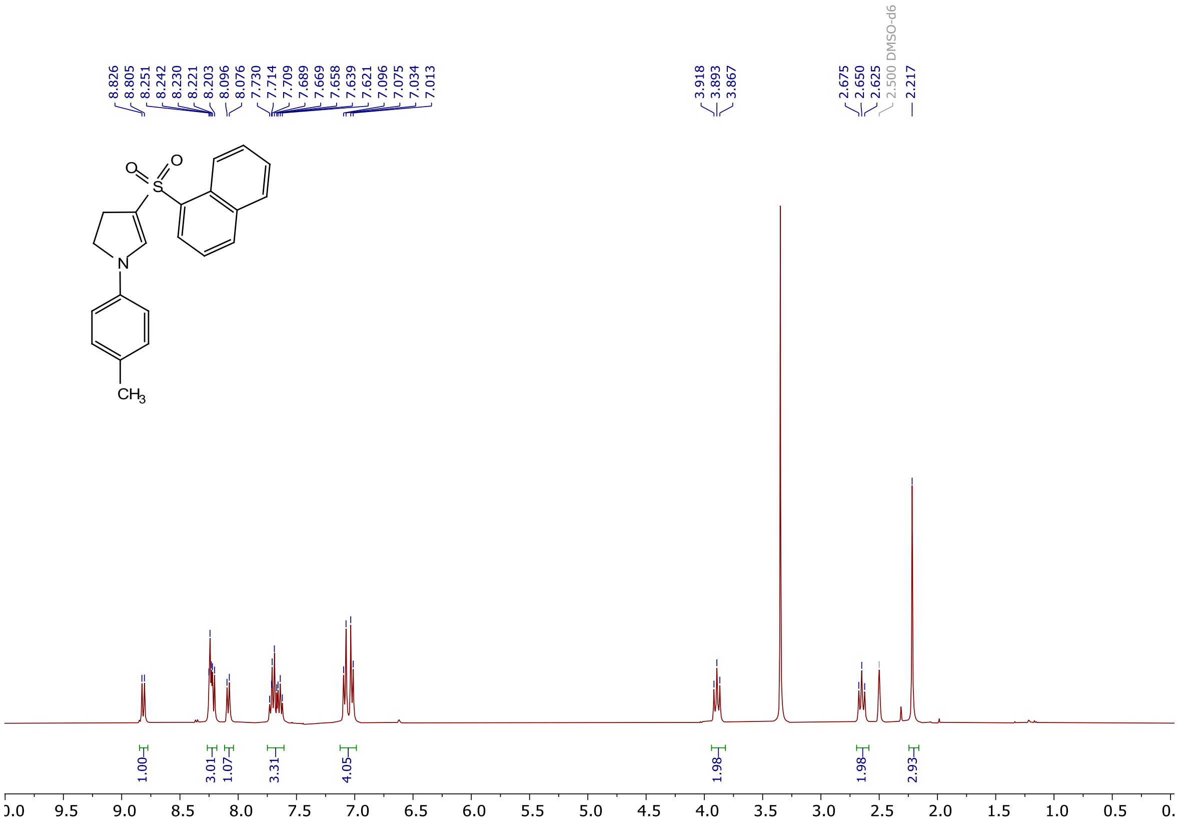

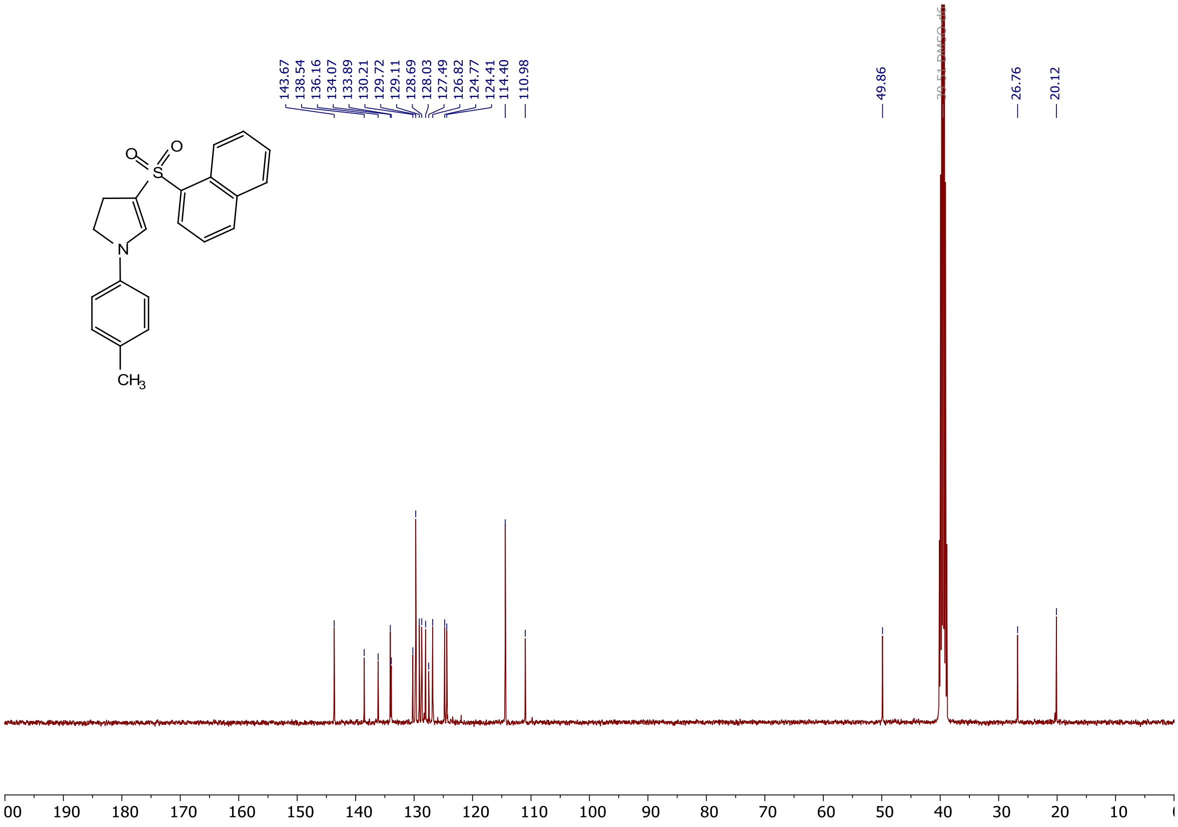

1-Phenyl-5-(*m*-tolylsulfonyl)-1,2,3,4-tetrahydropyridine (14)

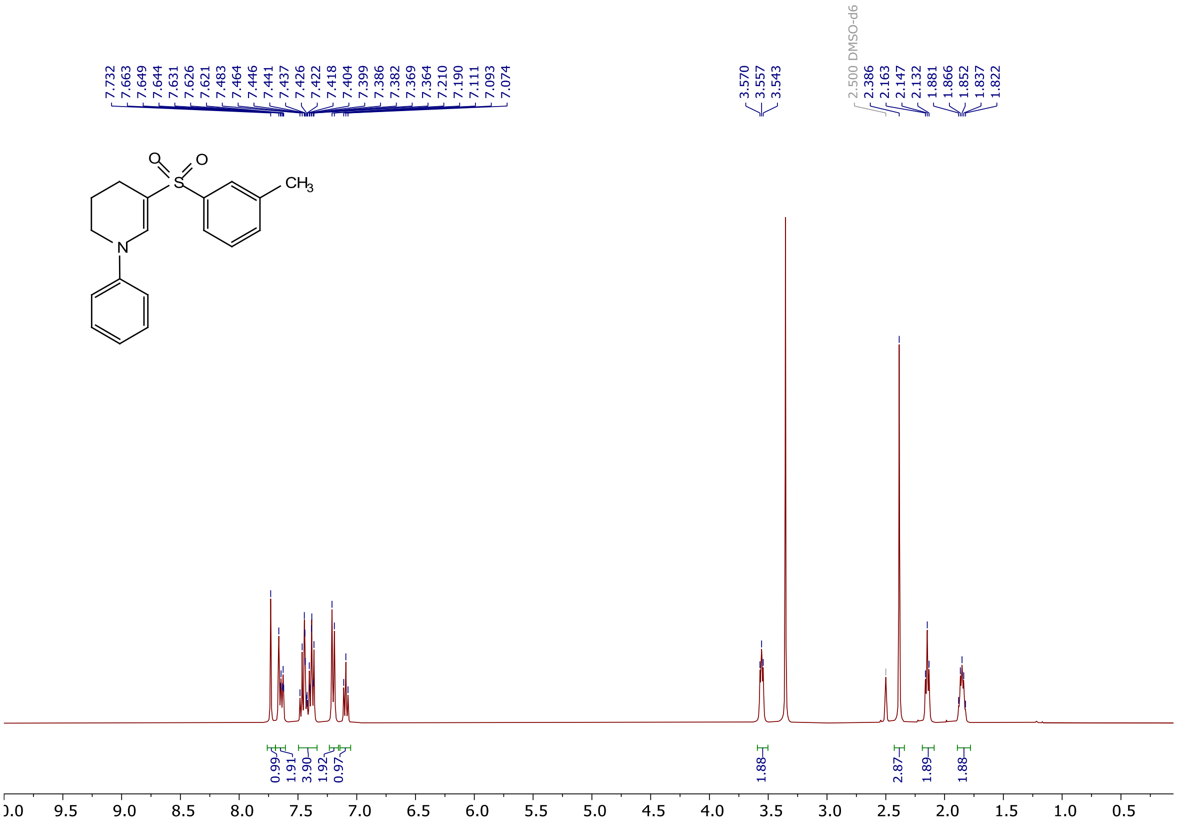

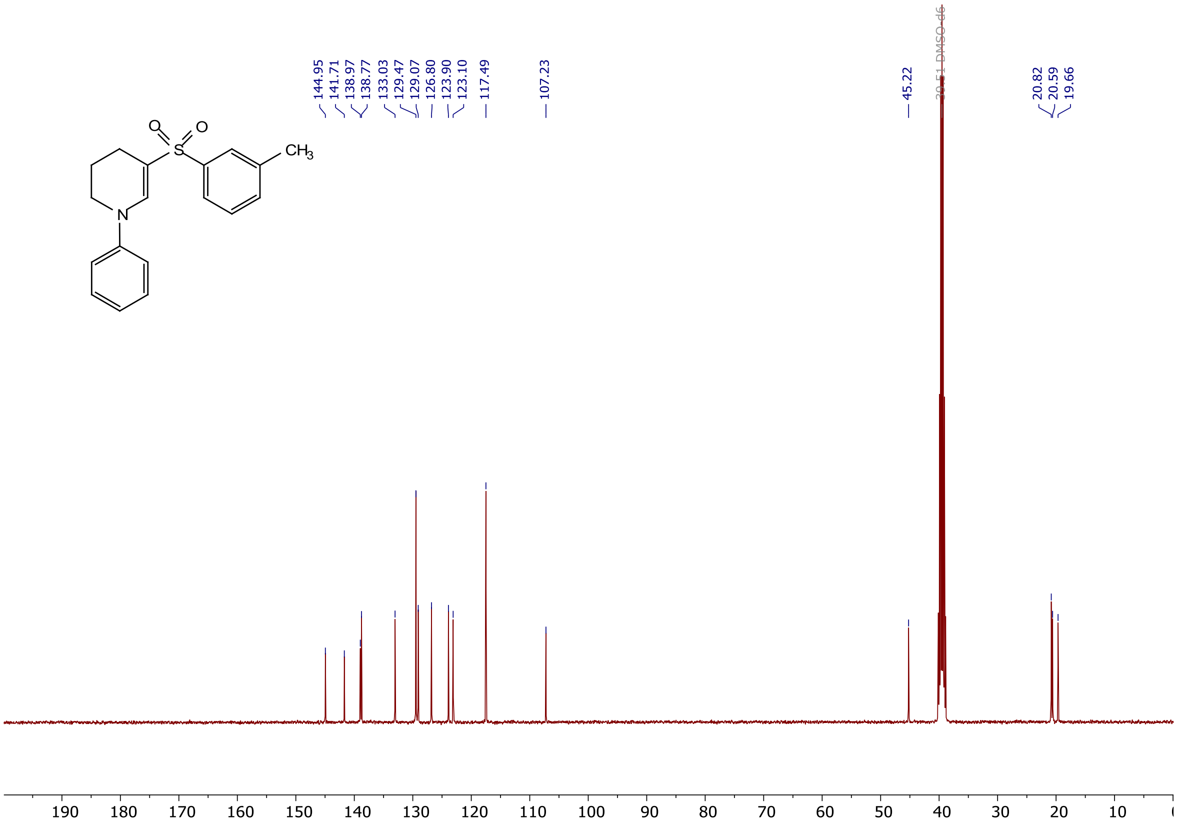

5-((3-Fluorophenyl)sulfonyl)-1-phenyl-1,2,3,4-tetrahydropyridine (15)

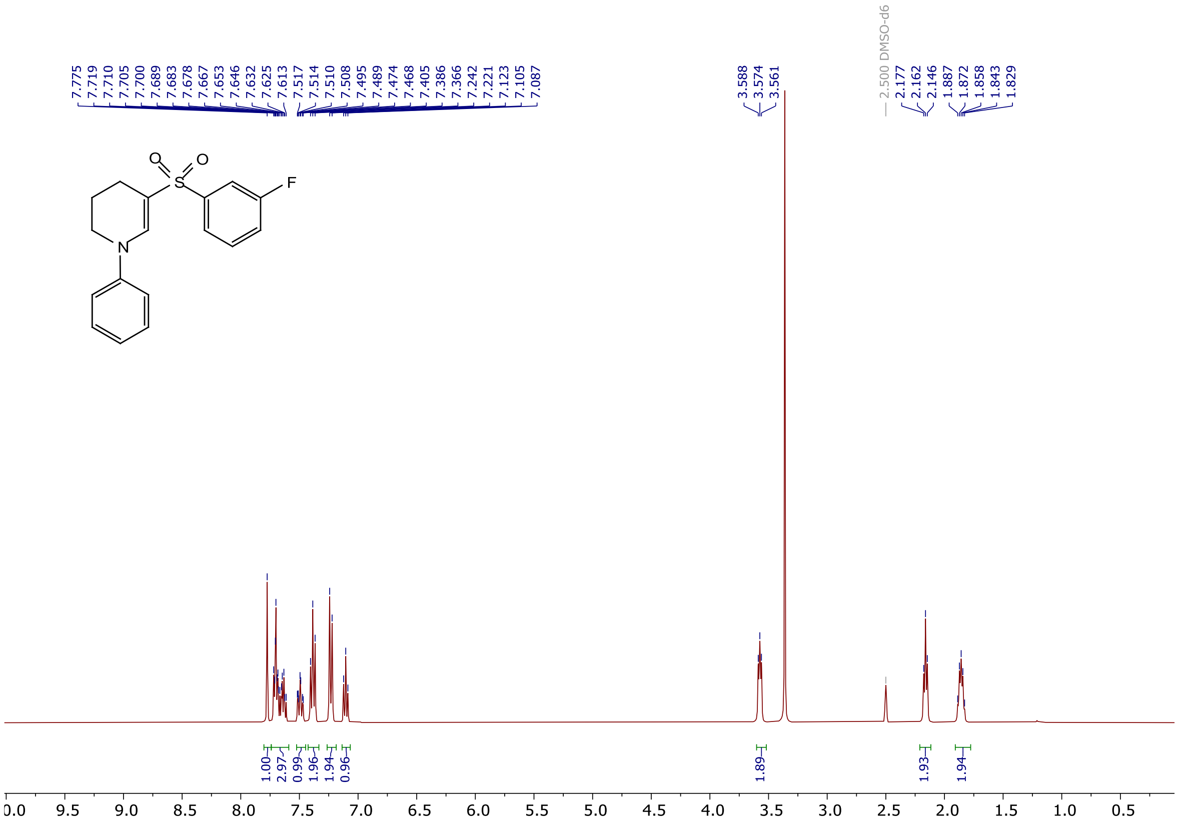

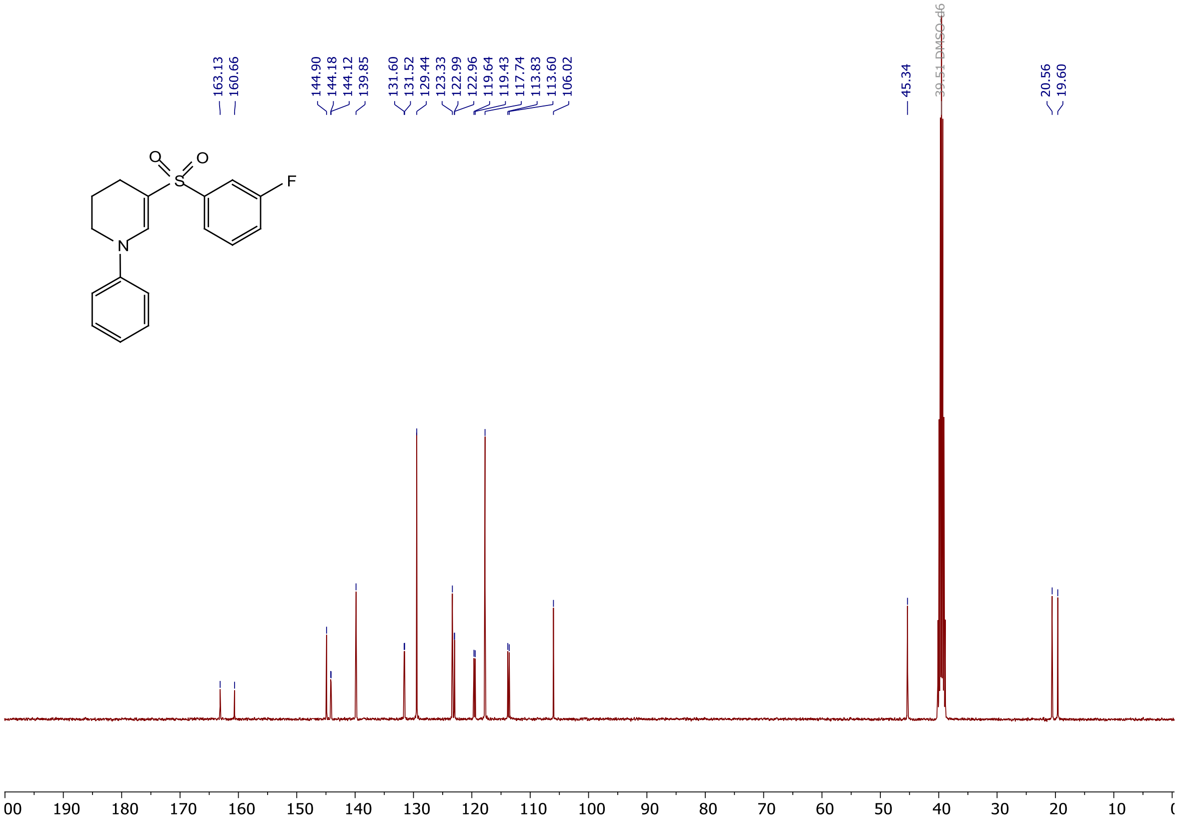

5-((2-Chlorophenyl)sulfonyl)-1-phenyl-1,2,3,4-tetrahydropyridine (16)

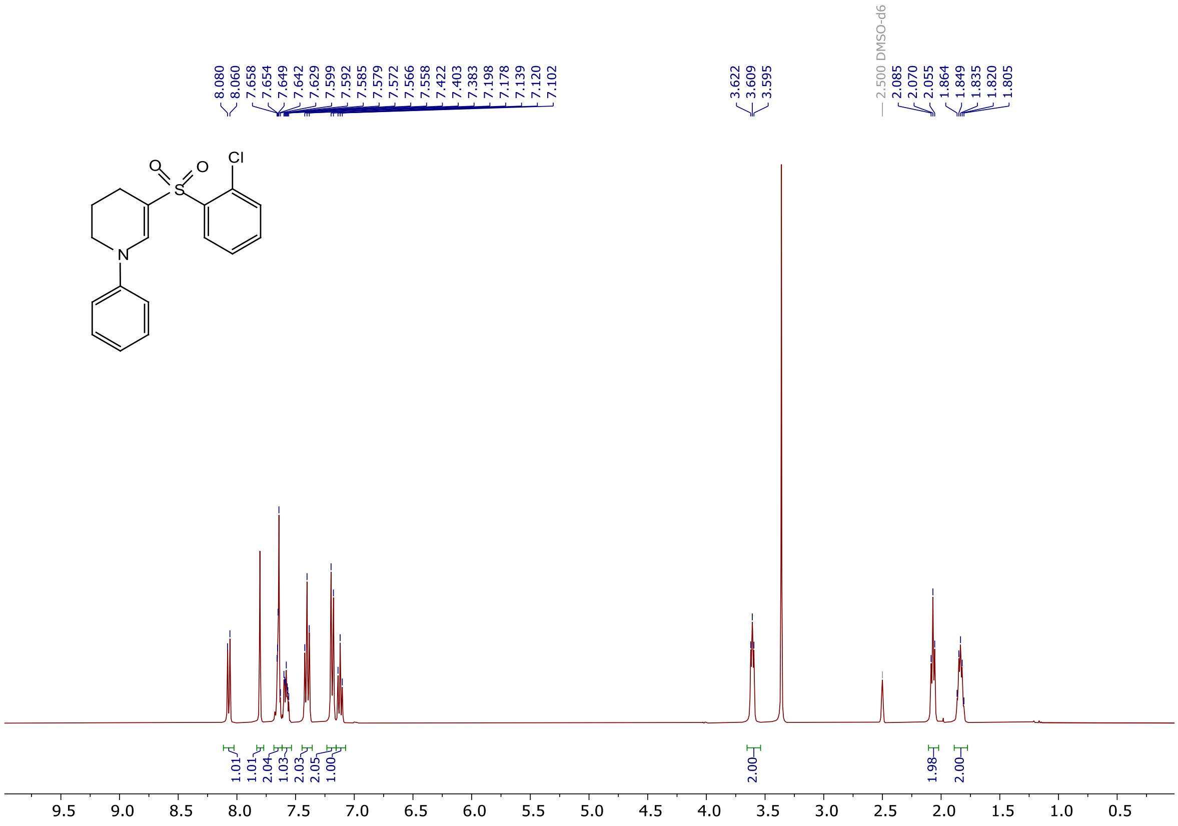

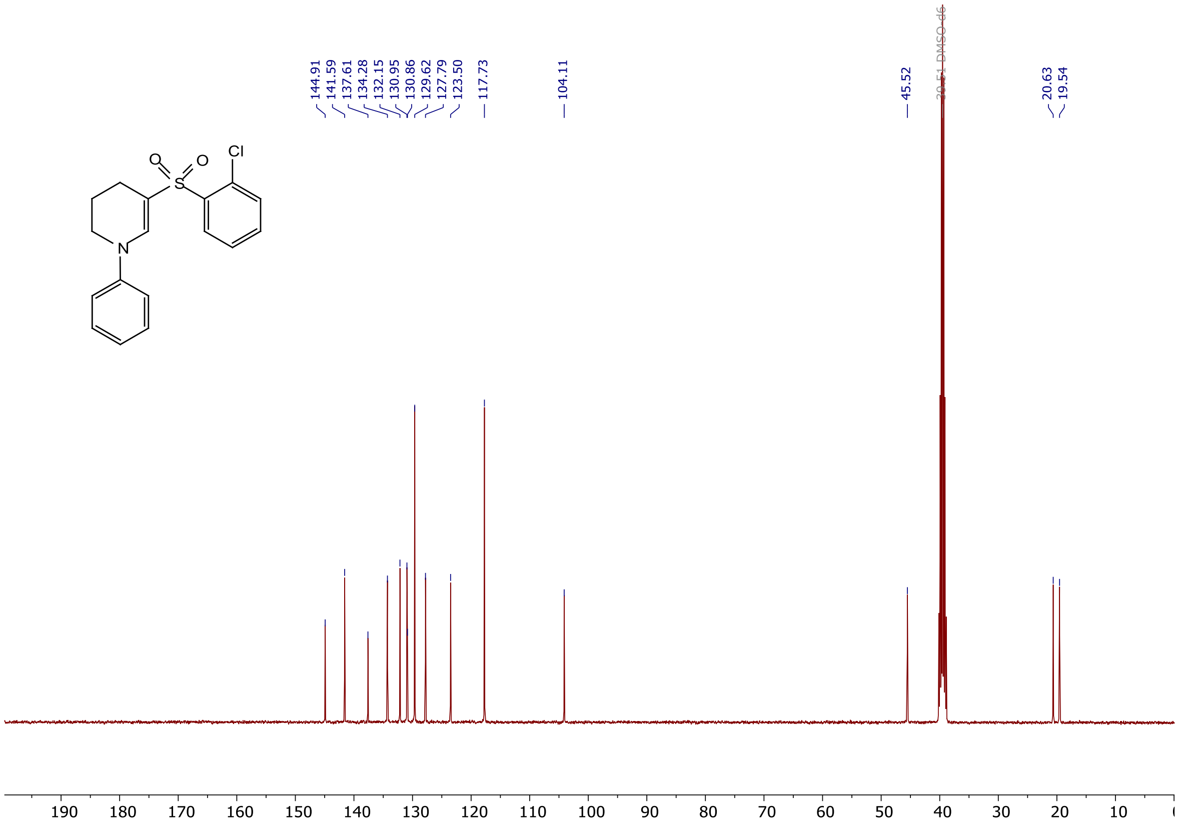

5-((3-Chloro-4-fluorophenyl)sulfonyl)-1-phenyl-1,2,3,4-tetrahydropyridine (17)

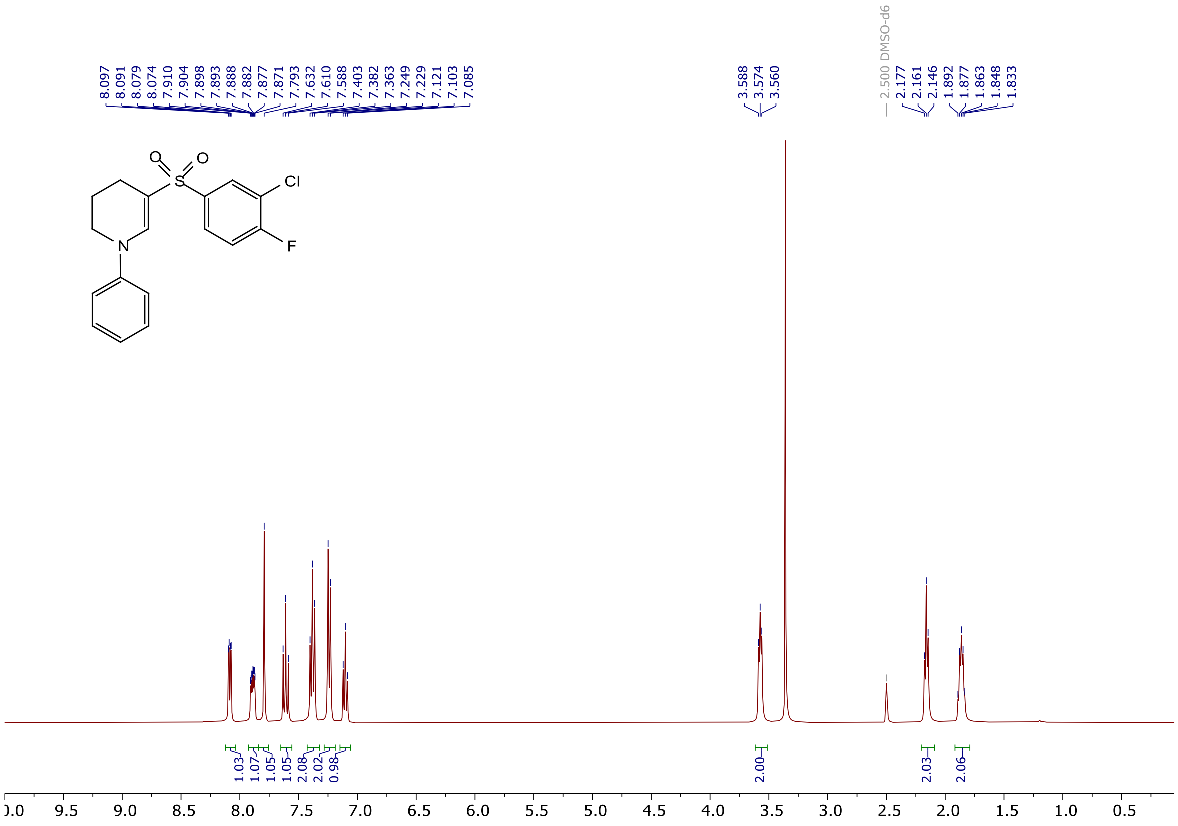

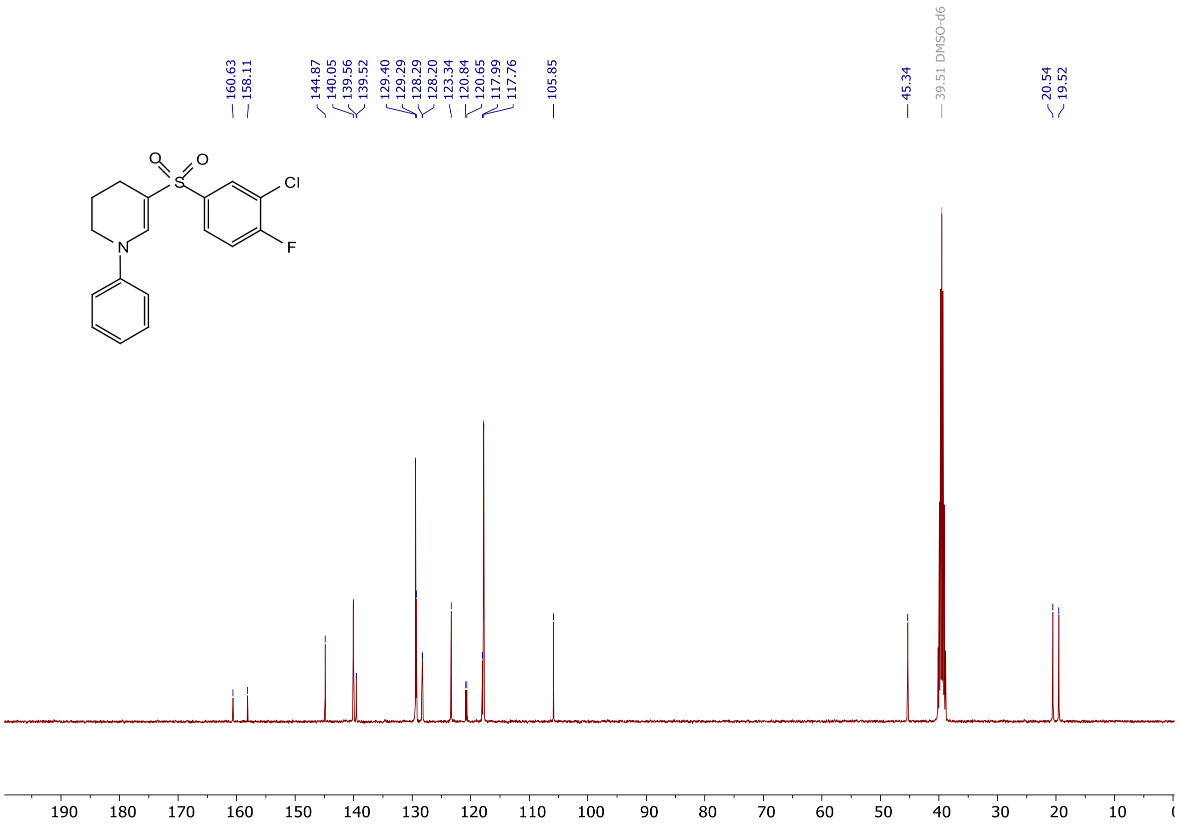

1-Mesityl-4-tosyl-2,3-dihydro-1*H*-pyrrole (18)

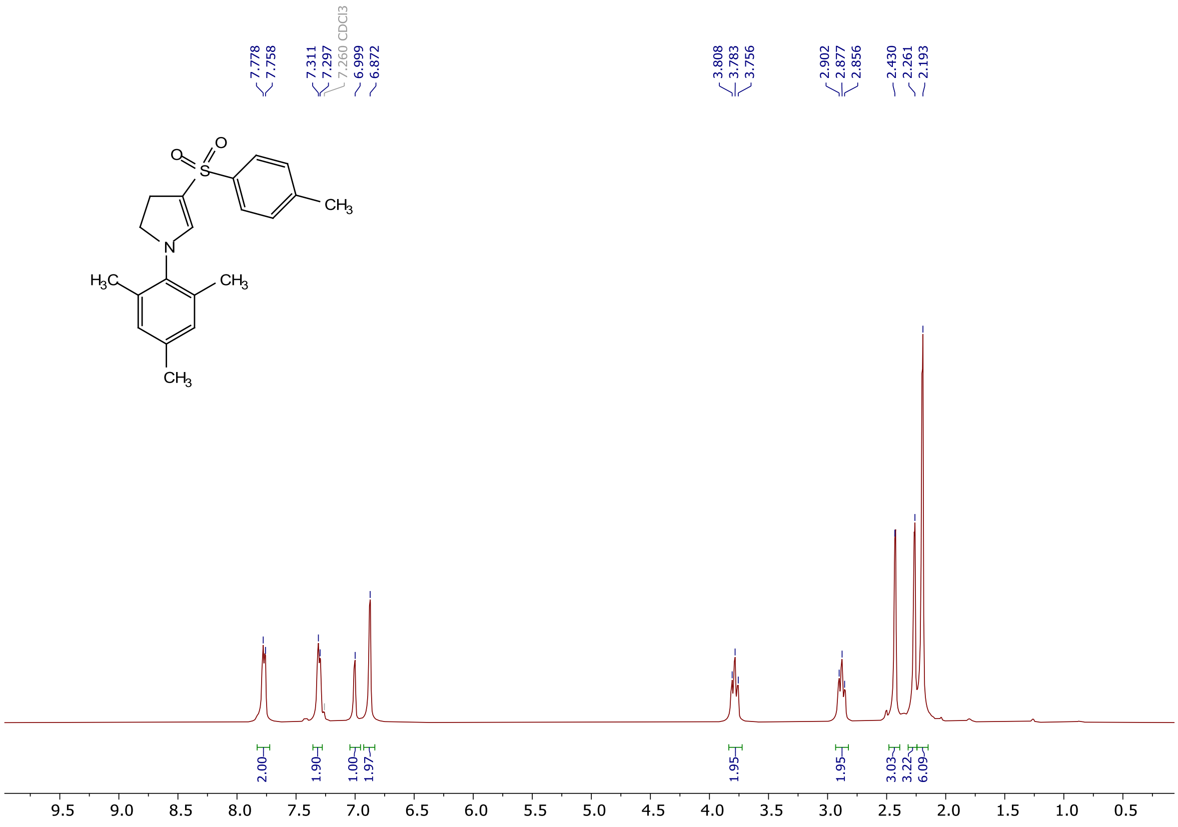

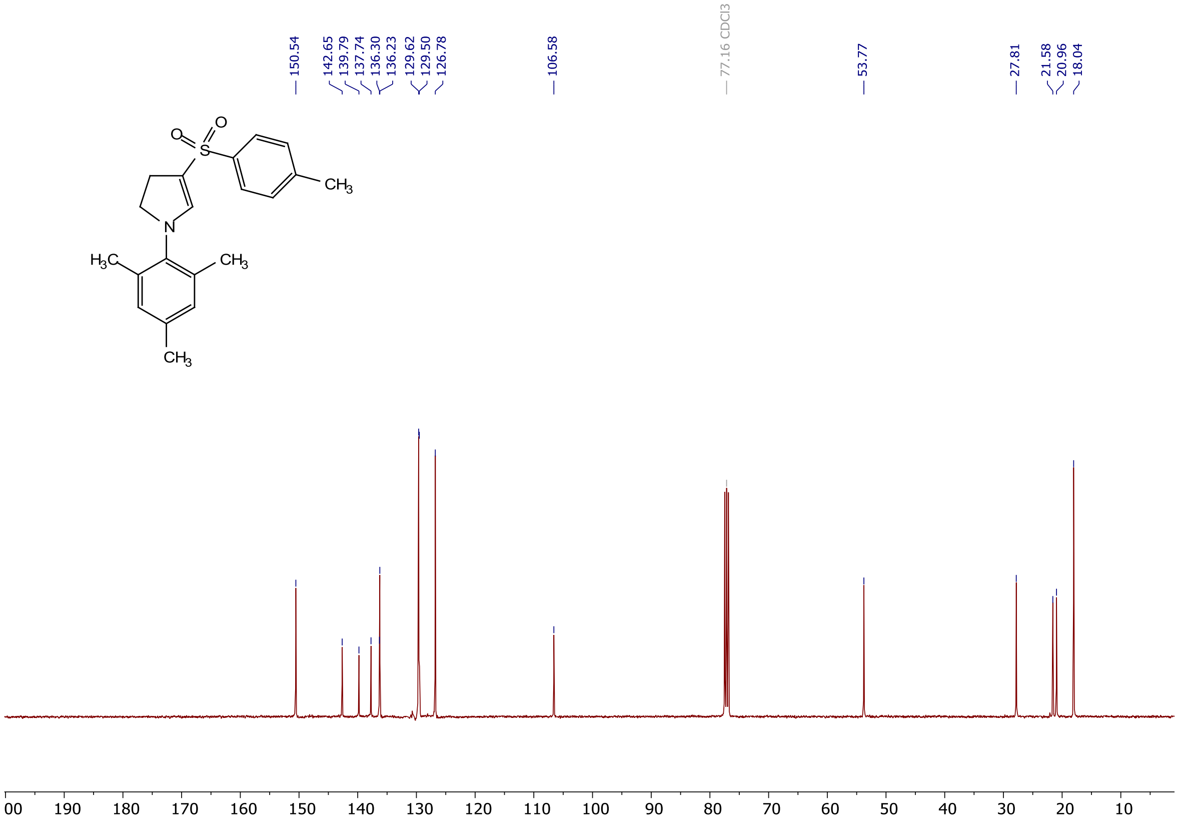

1-(2,6-Dimethylphenyl)-4-tosyl-2,3-dihydro-1*H*-pyrrole (19)

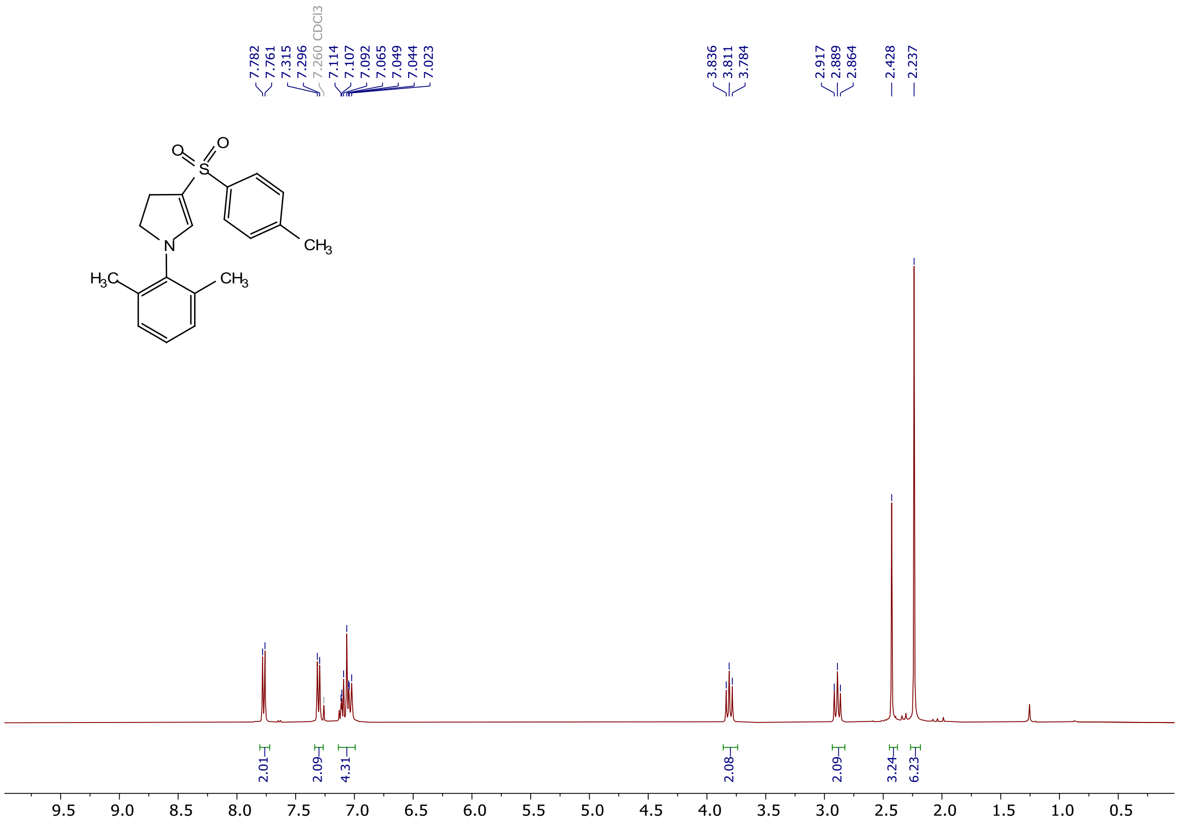

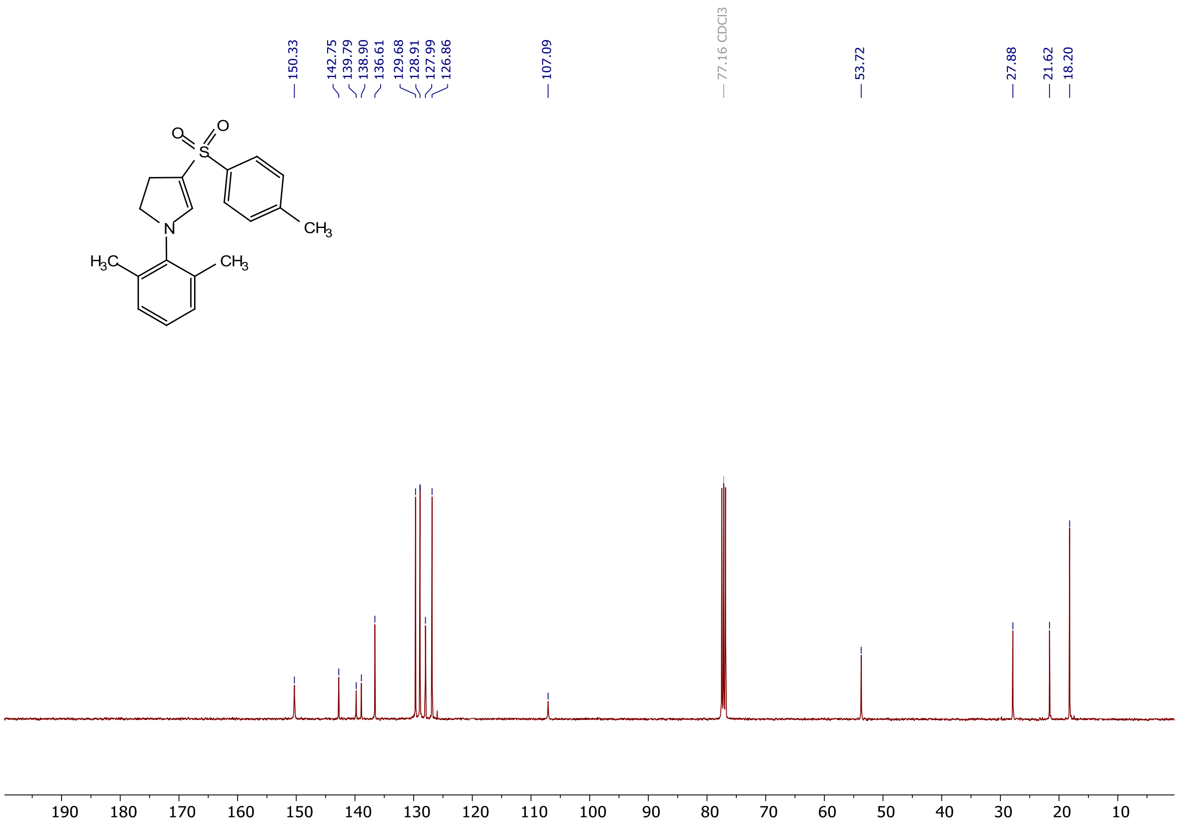

1-(4-(*tert*-Butyl)phenyl)-4-tosyl-2,3-dihydro-1*H*-pyrrole (20)

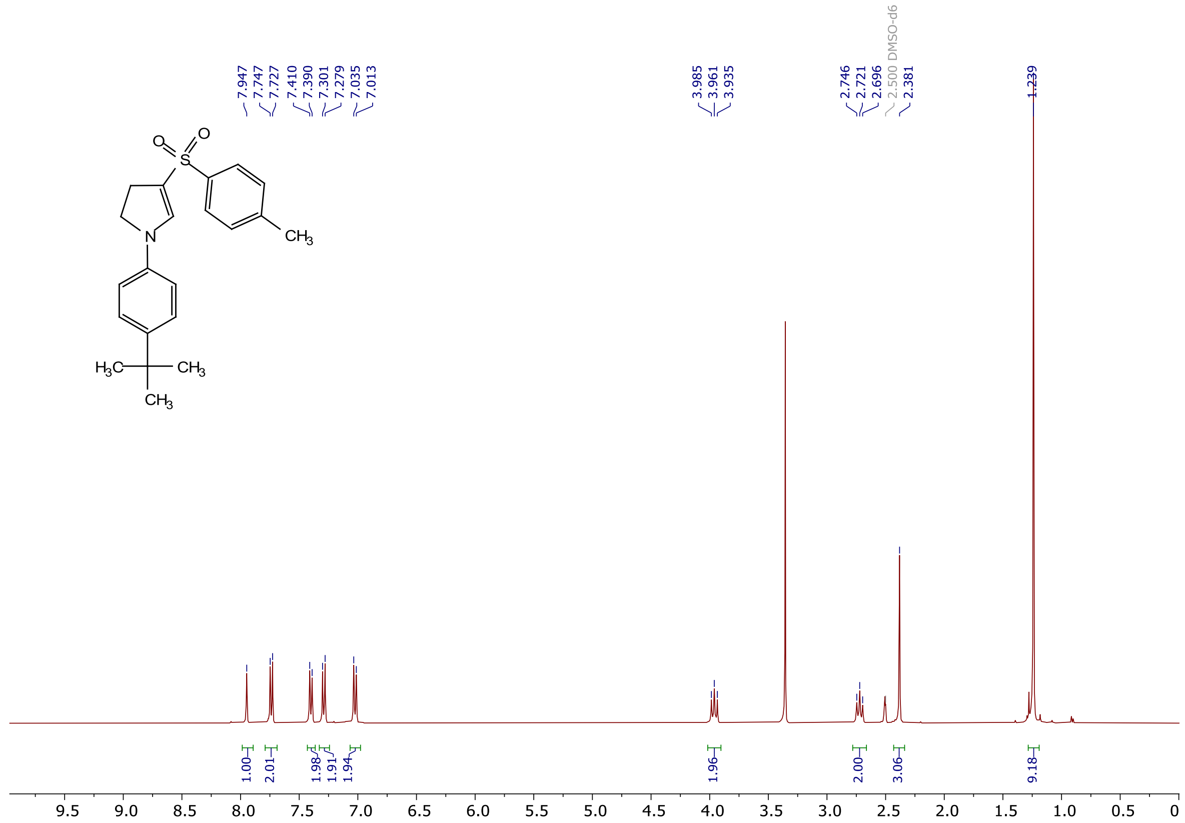

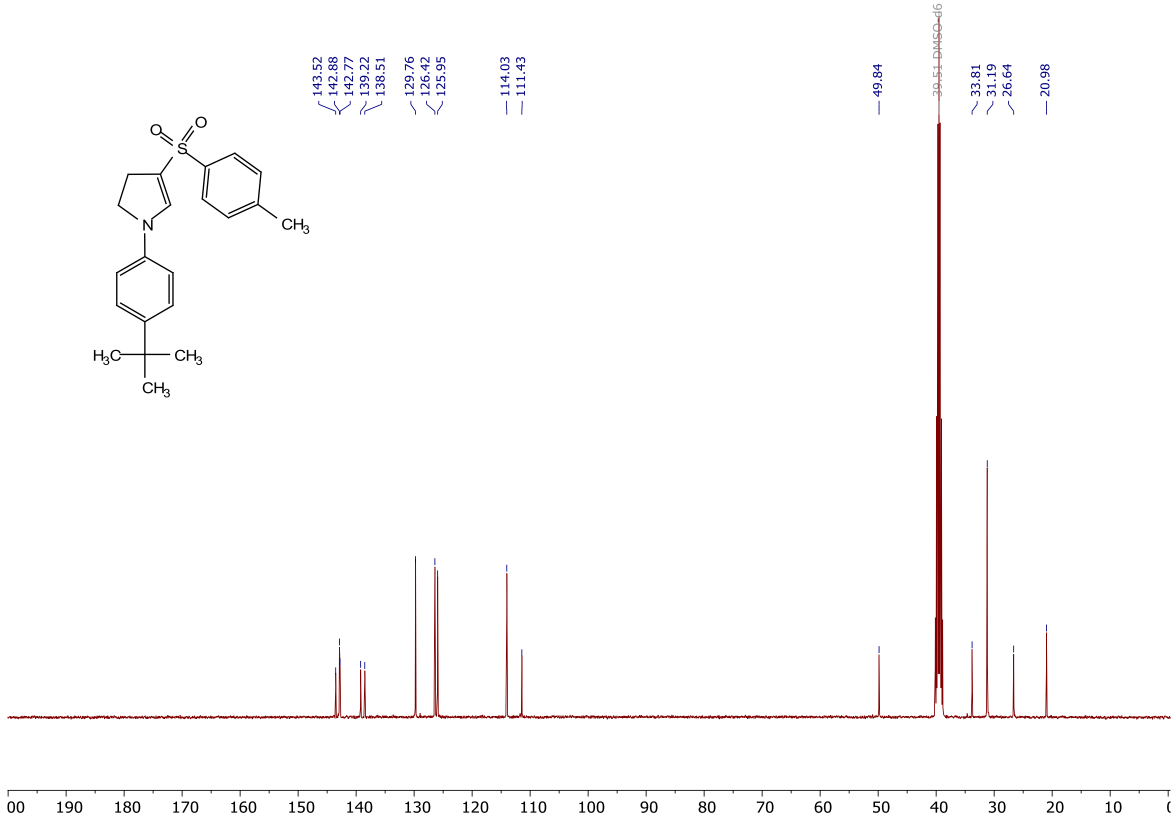

1-(4-Methoxyphenyl)-4-tosyl-2,3-dihydro-1*H*-pyrrole (21)

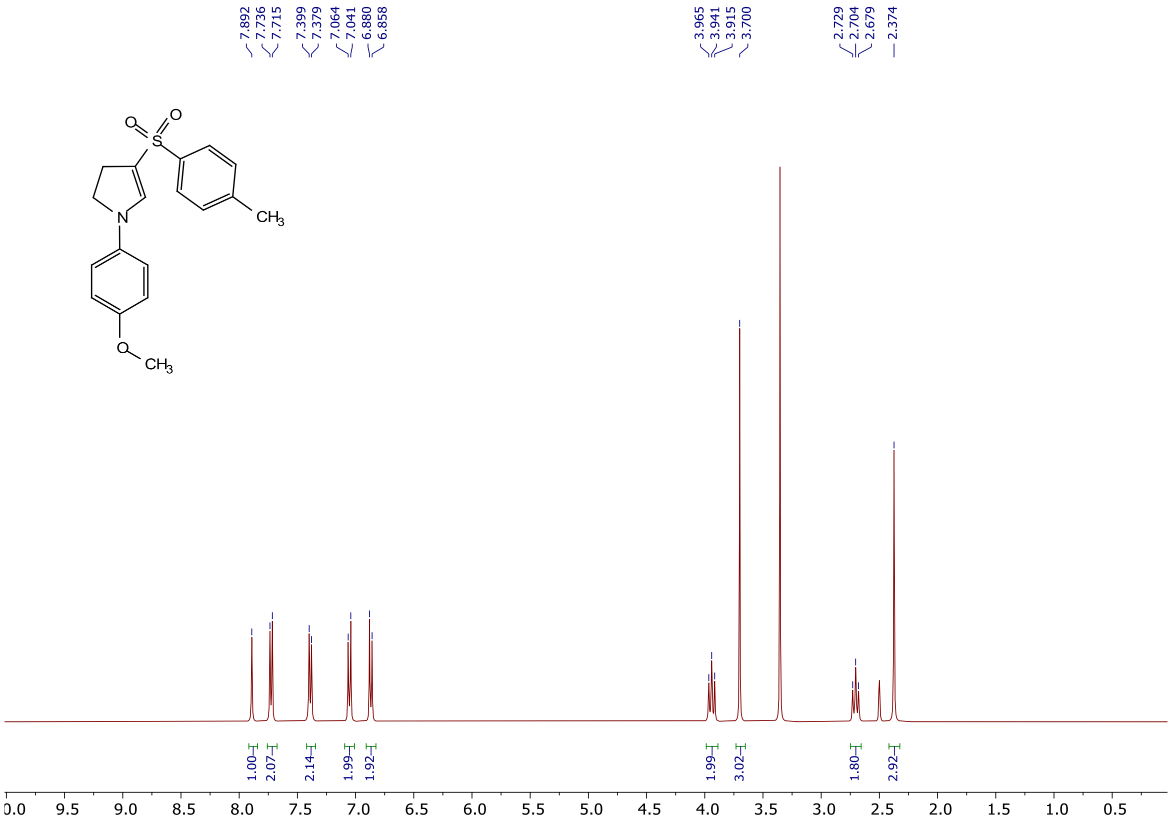

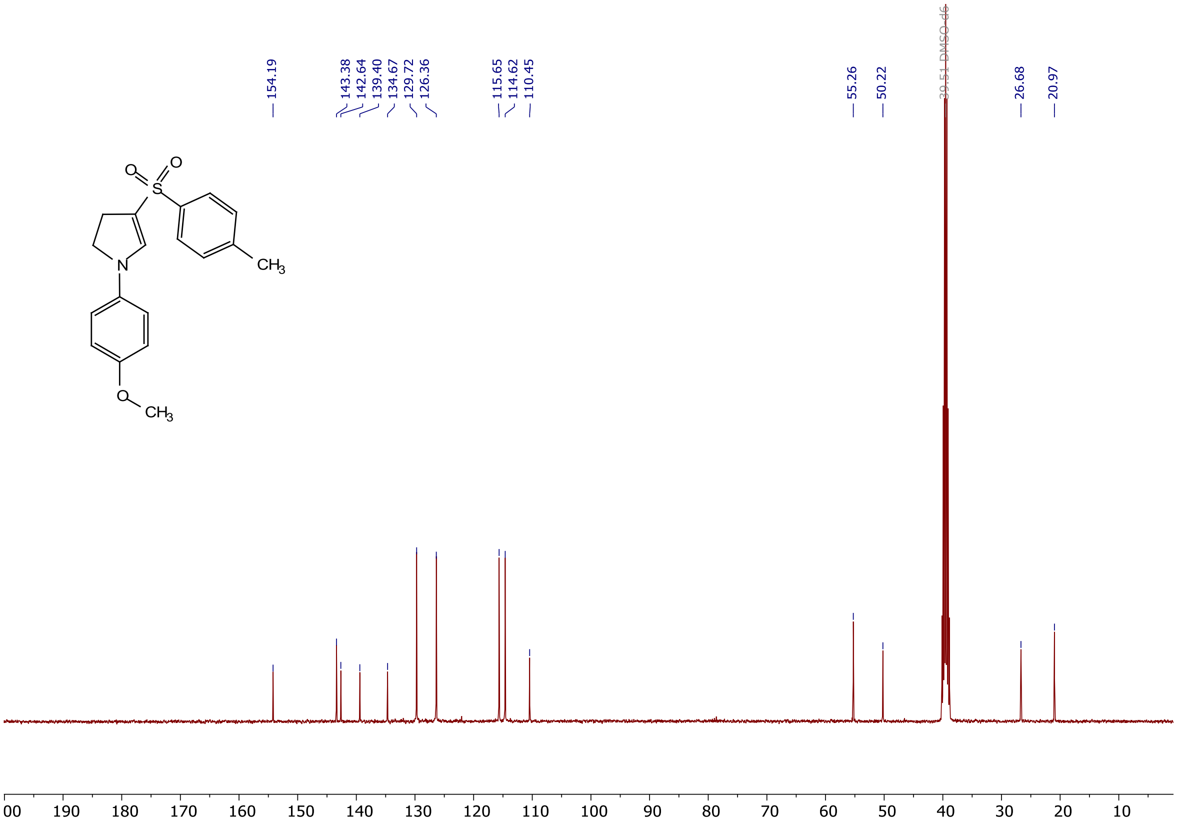

1-Phenyl-4-tosyl-2,3-dihydro-1*H*-pyrrole (22)

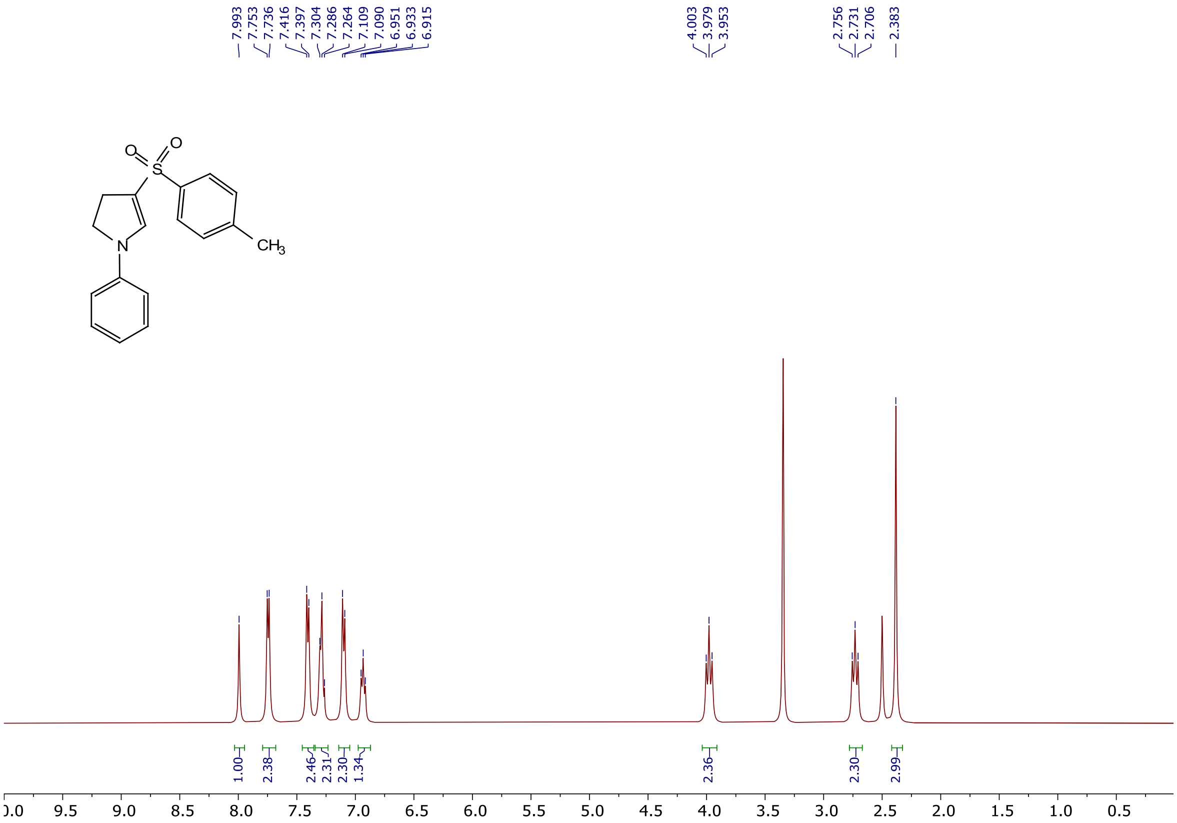

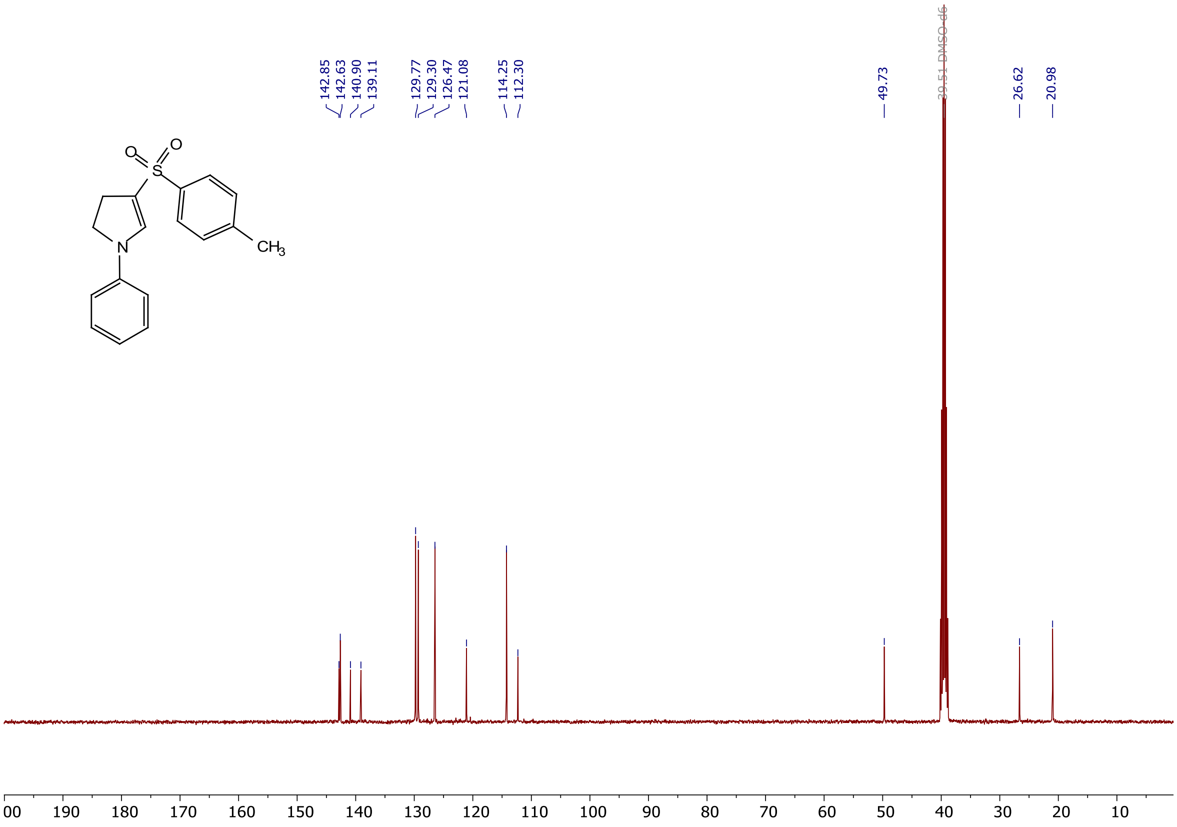

1-(4-Fluorophenyl)-4-tosyl-2,3-dihydro-1*H*-pyrrole (23)

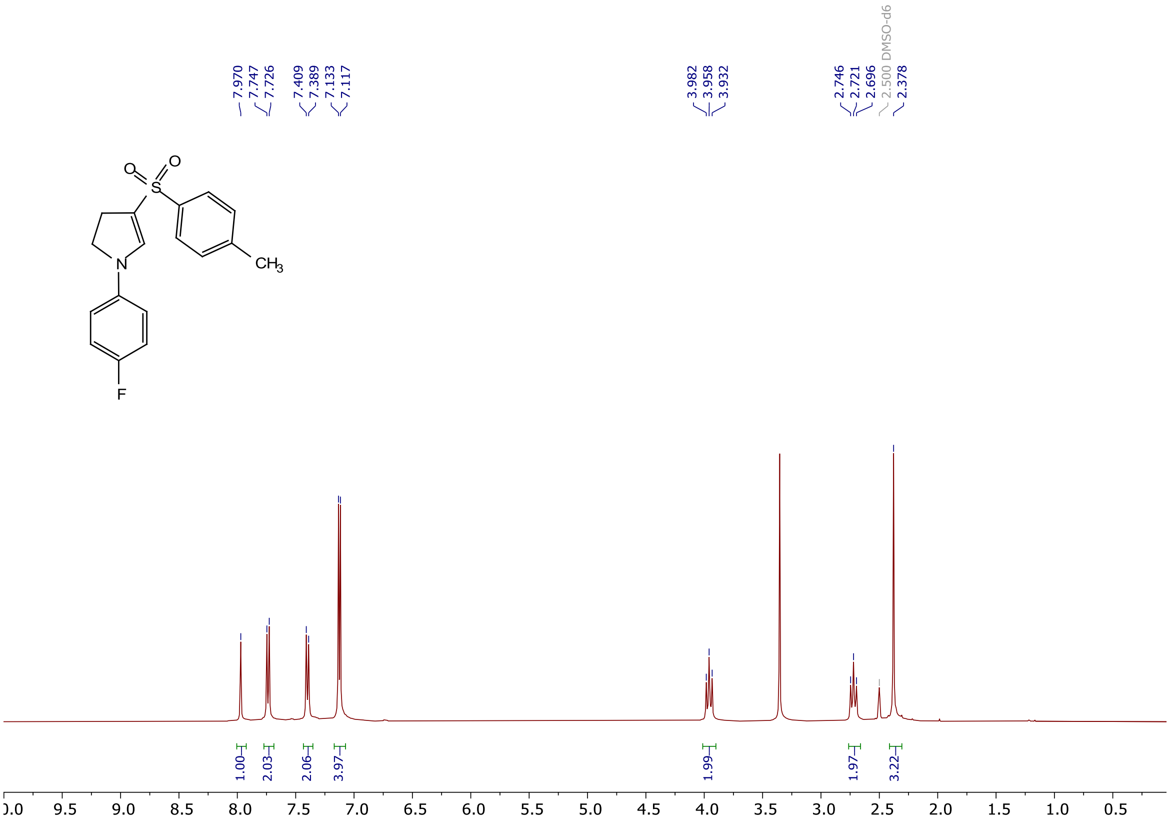

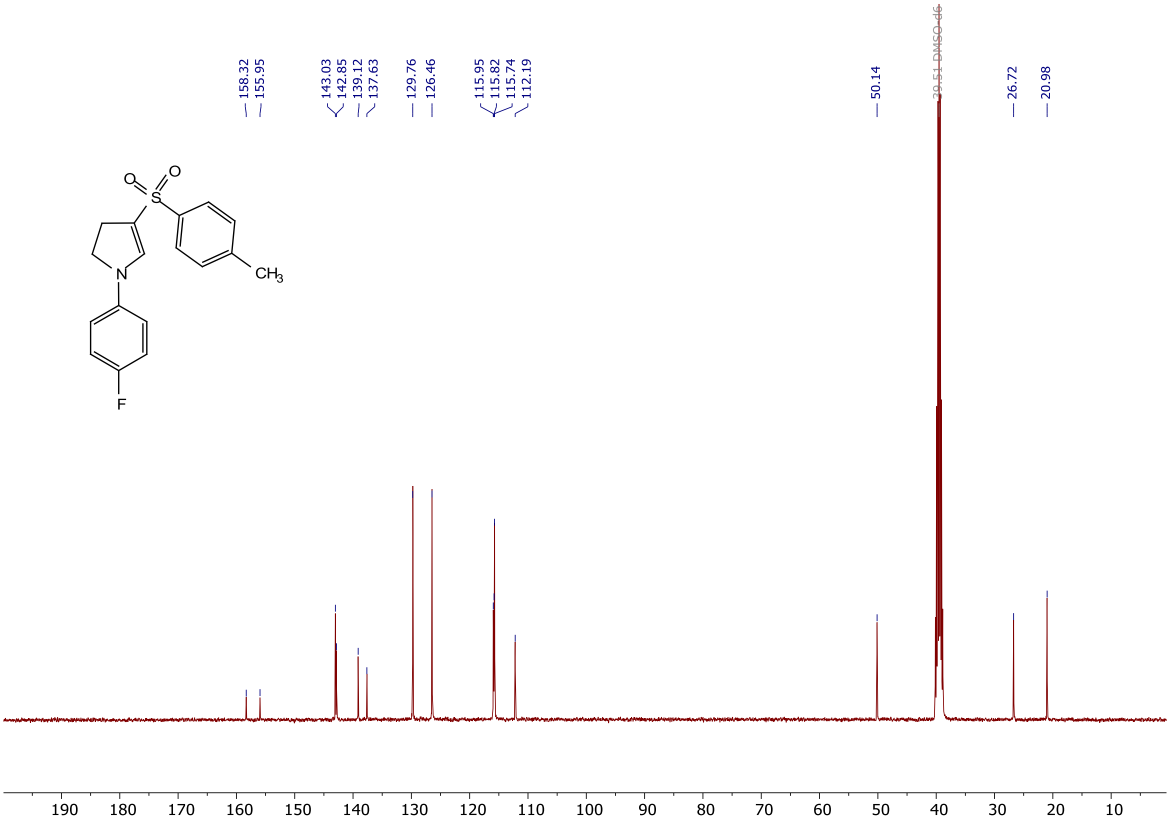

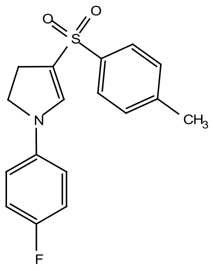

O
O
S
N
CH3
F

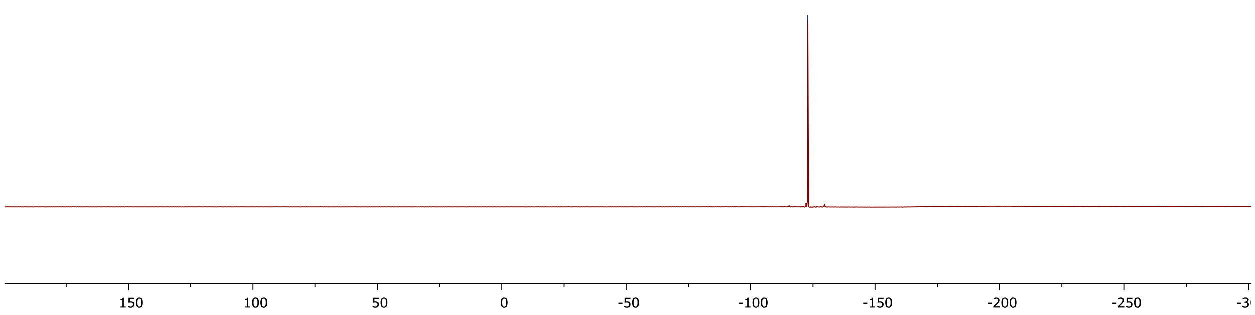

-122.94
150
100
50
0
-50
-100
-150
-200
-250

1-(4-Chlorophenyl)-4-tosyl-2,3-dihydro-1*H*-pyrrole (24)

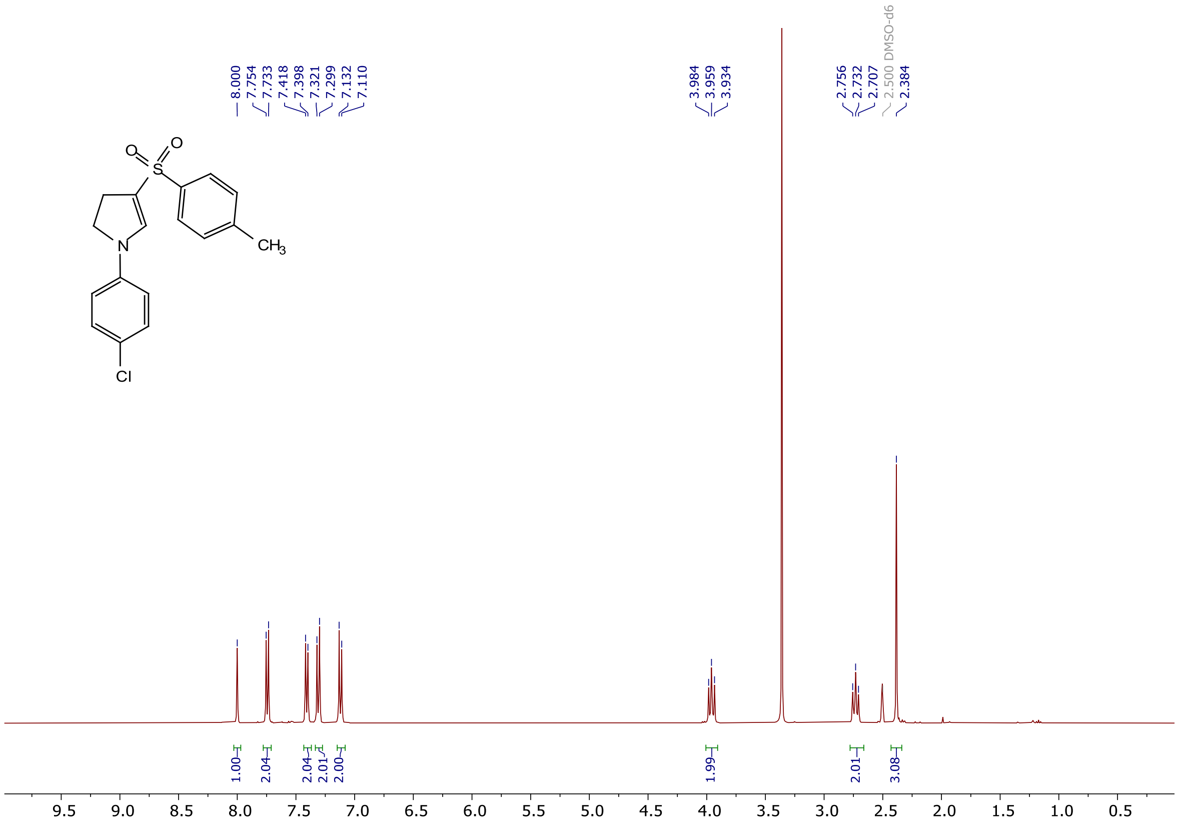

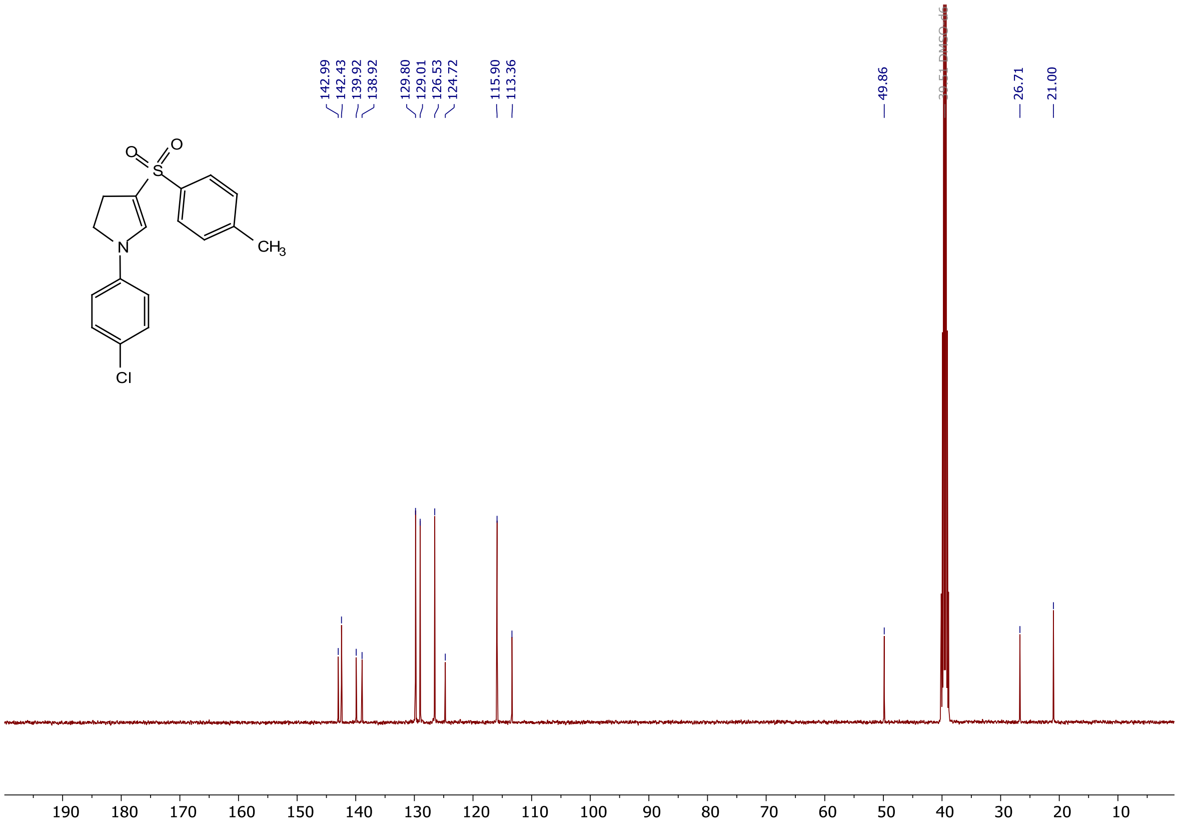

1-(2-Methoxyphenyl)-4-tosyl-2,3-dihydro-1*H*-pyrrole (25)

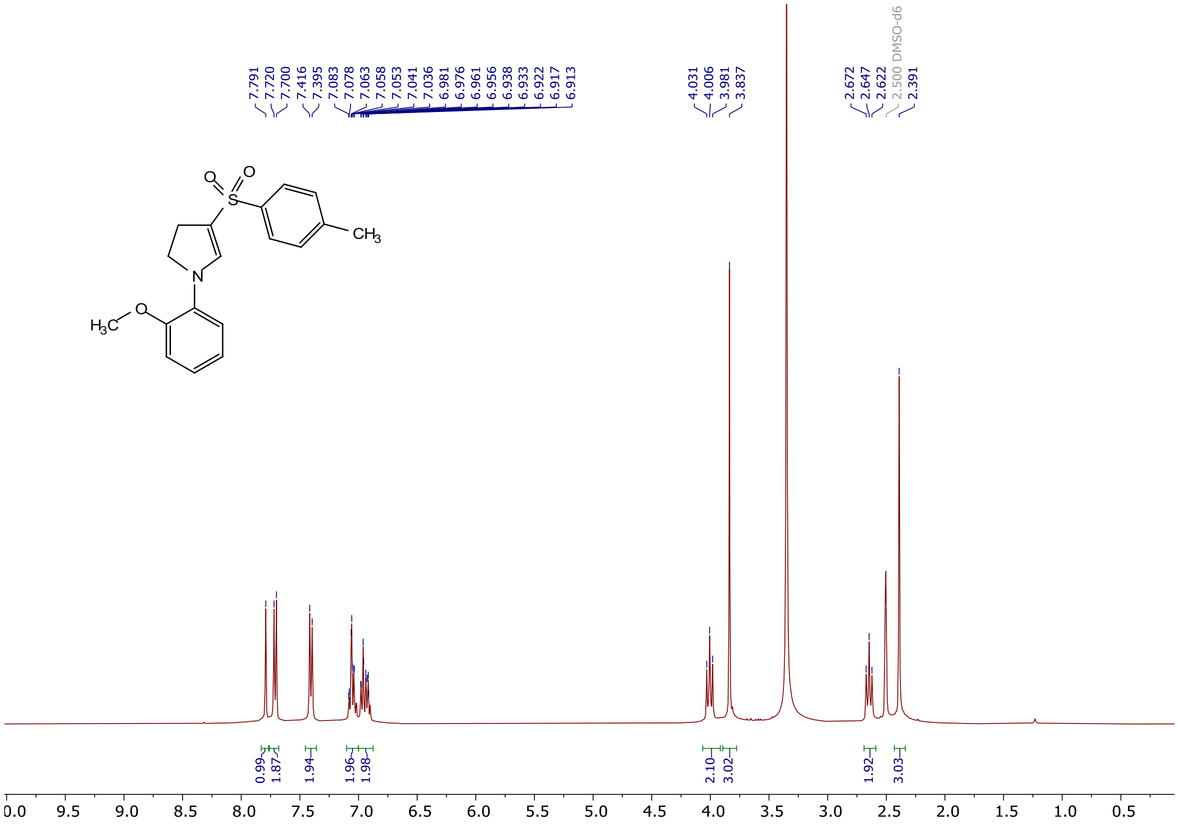

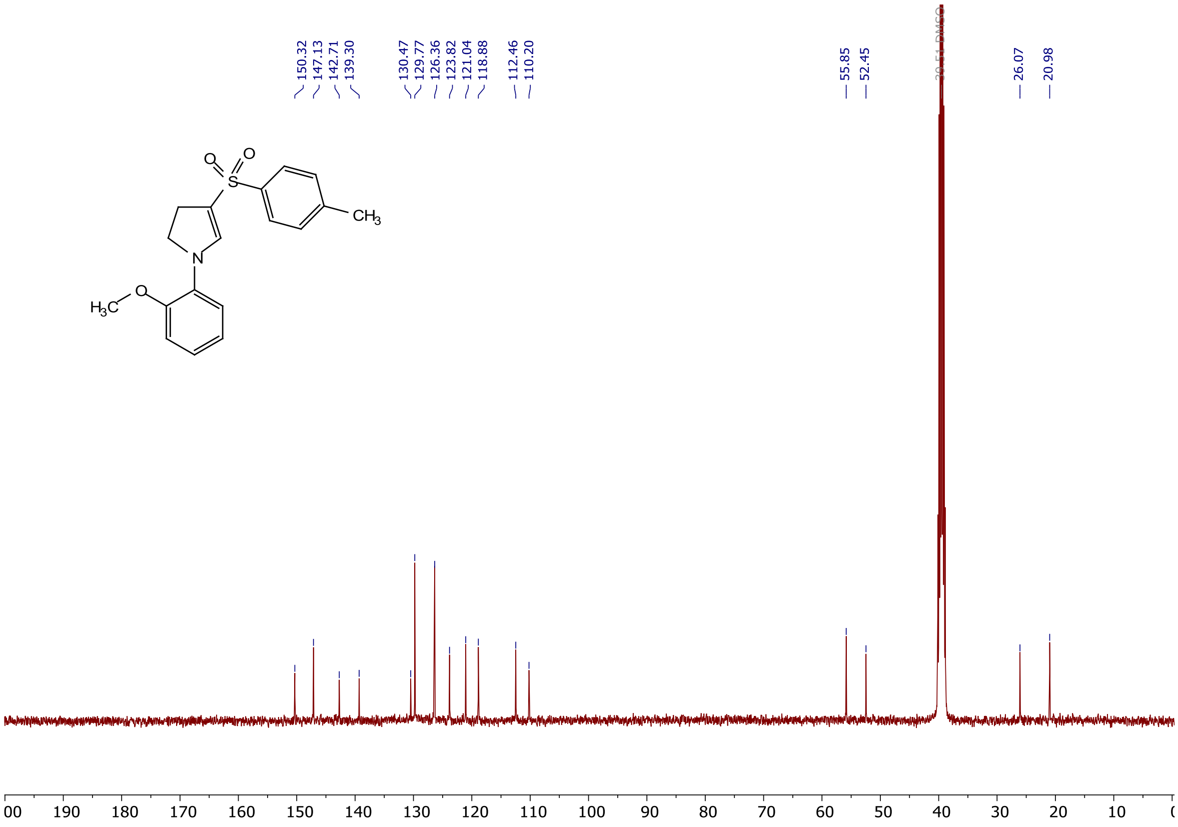

1-(4-Methoxy-2-methylphenyl)-4-tosyl-2,3-dihydro-1*H*-pyrrole (26)

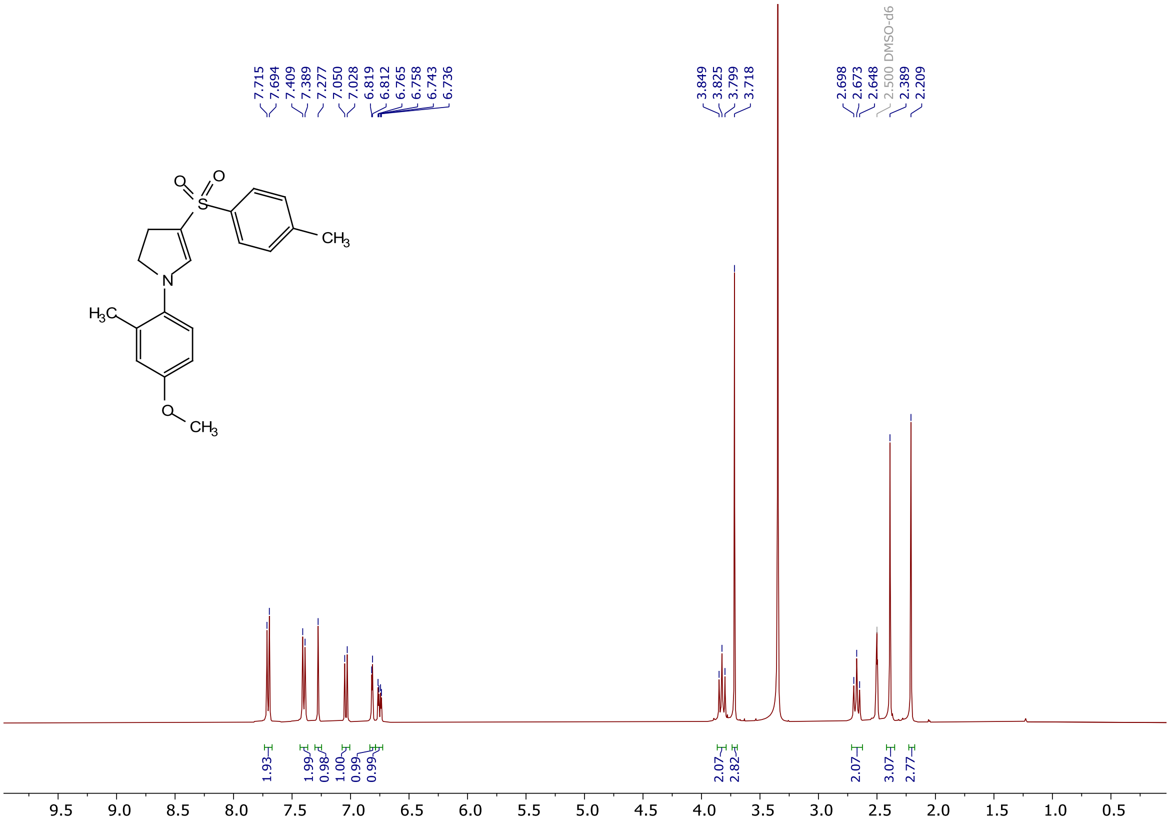

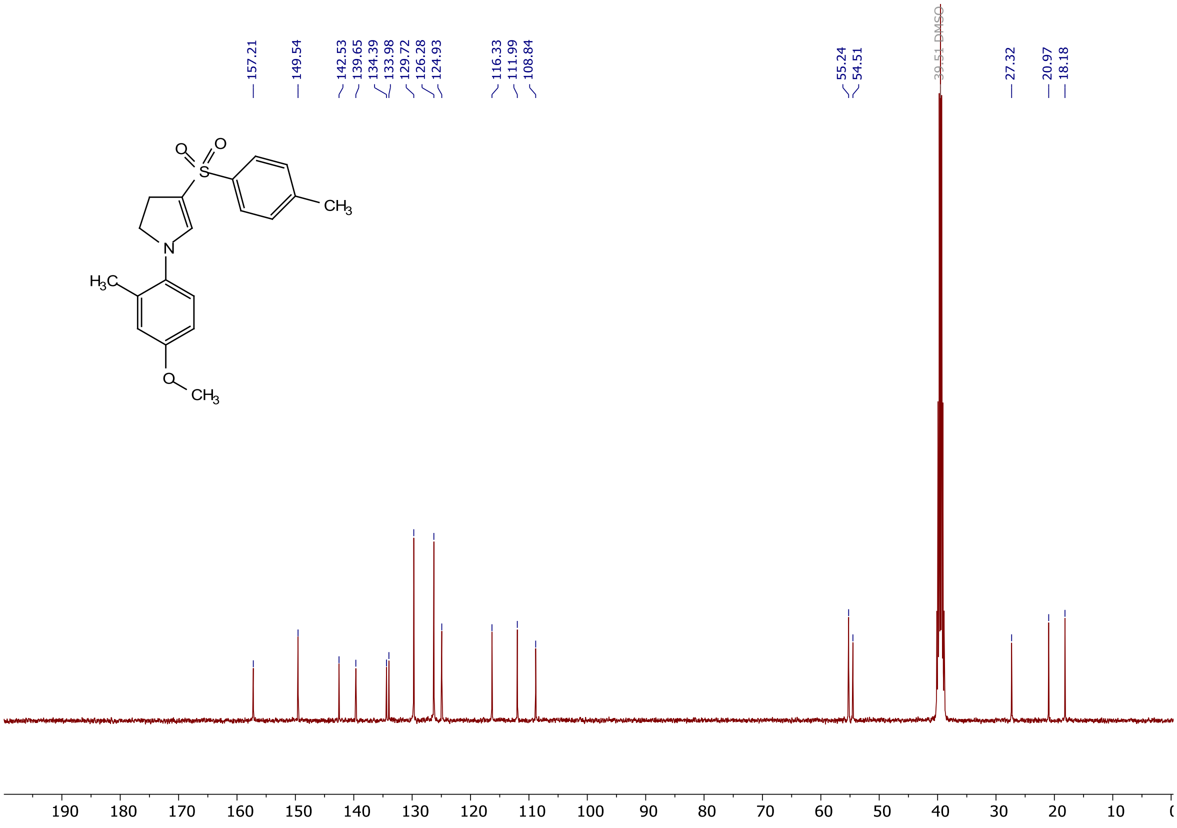

1-(2-Fluorophenyl)-4-tosyl-2,3-dihydro-1*H*-pyrrole (27)

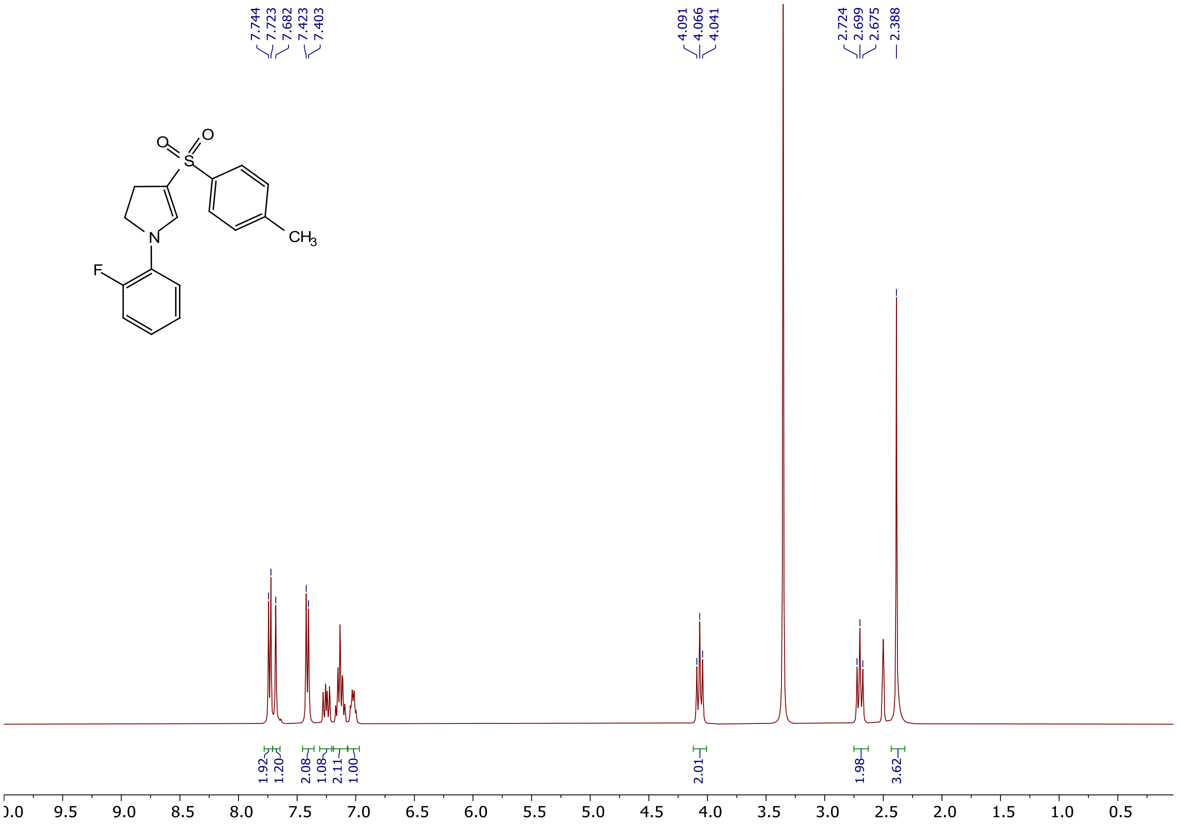

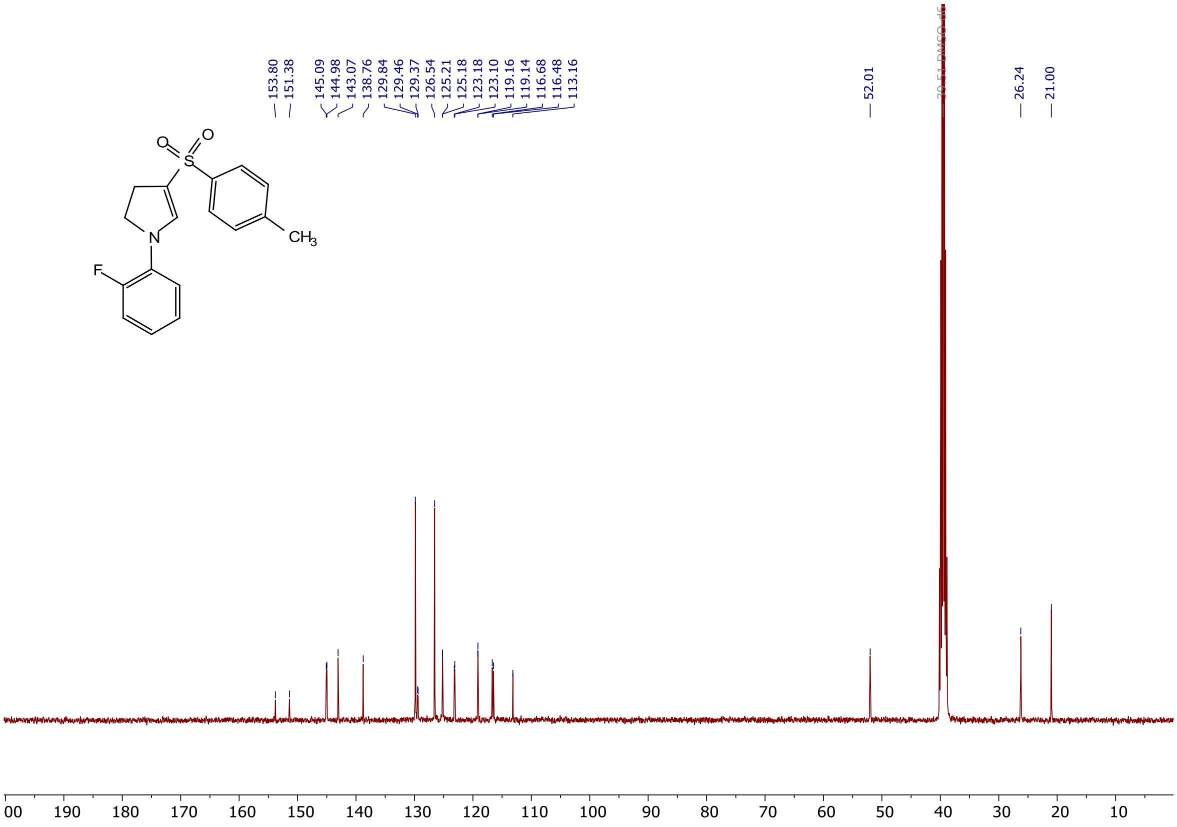

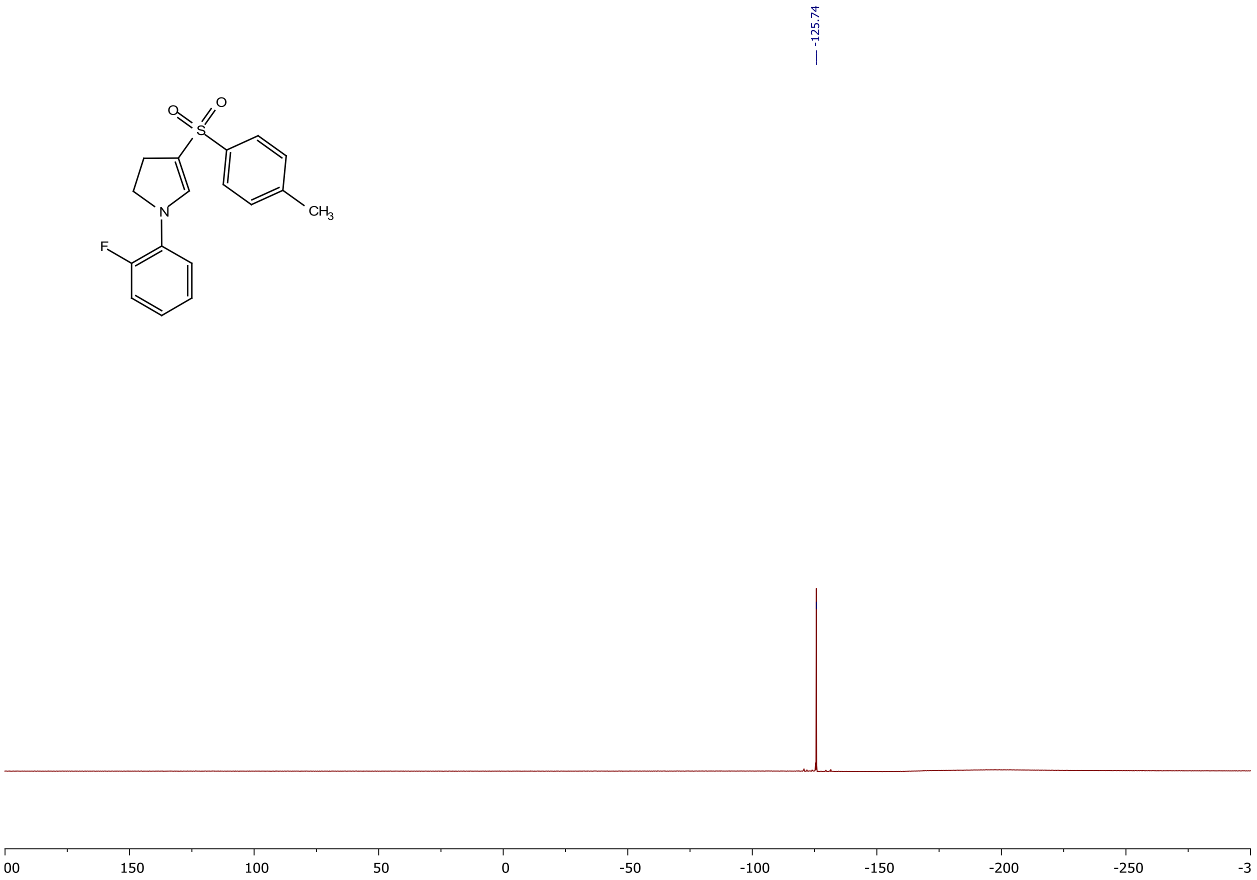
-125.74
O
O
S
N
F
CH3
00
150
100
50
0
-50
-100
-150
-200
-250
-3

1-Phenyl-6-tosyl-2,3,4,5-tetrahydro-1*H*-azepine (28)

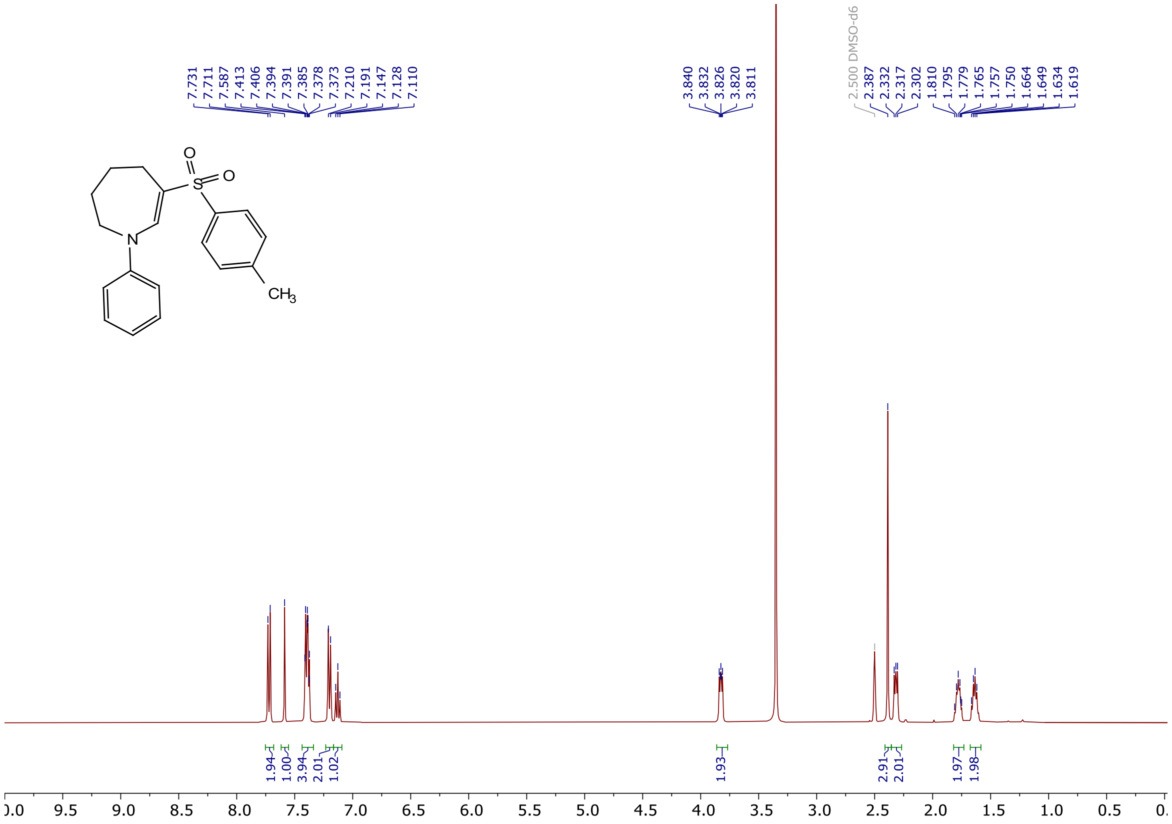

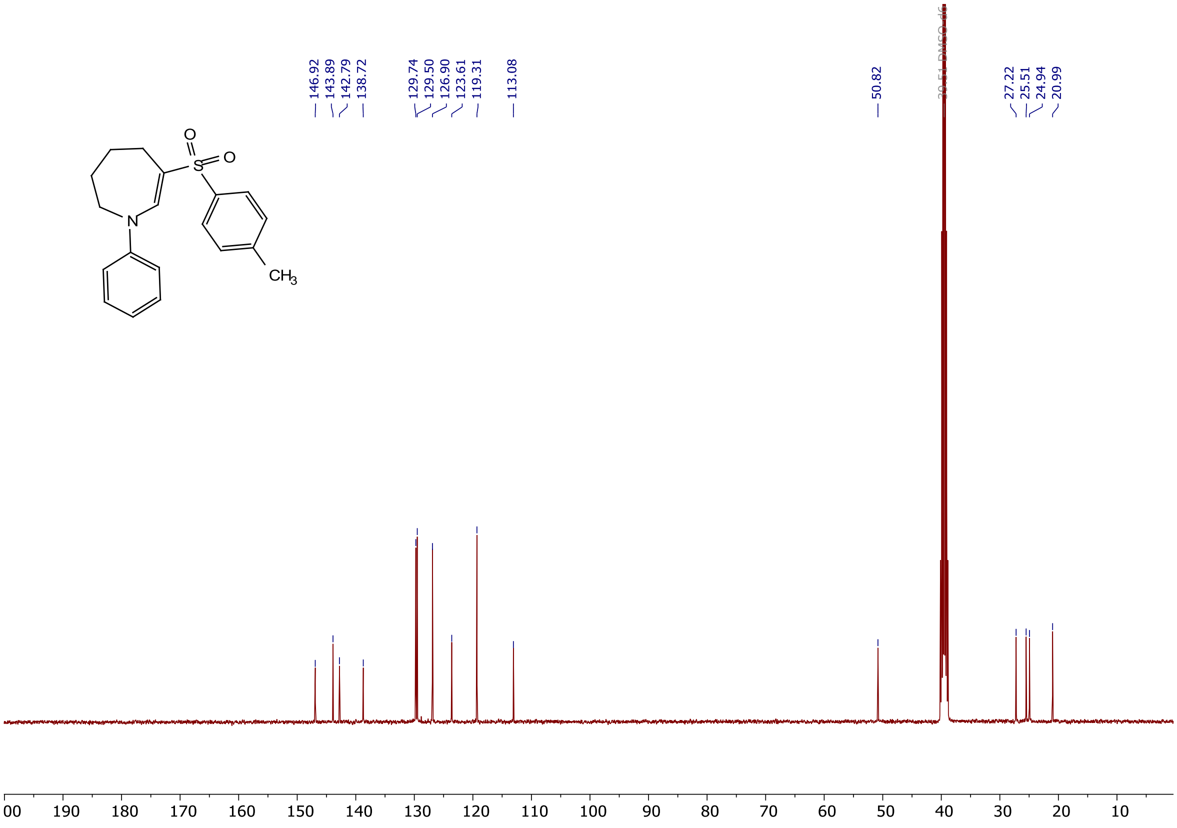

(*E*)-*N*-Ethyl-*N*-(2-tosylvinyl)aniline (29)

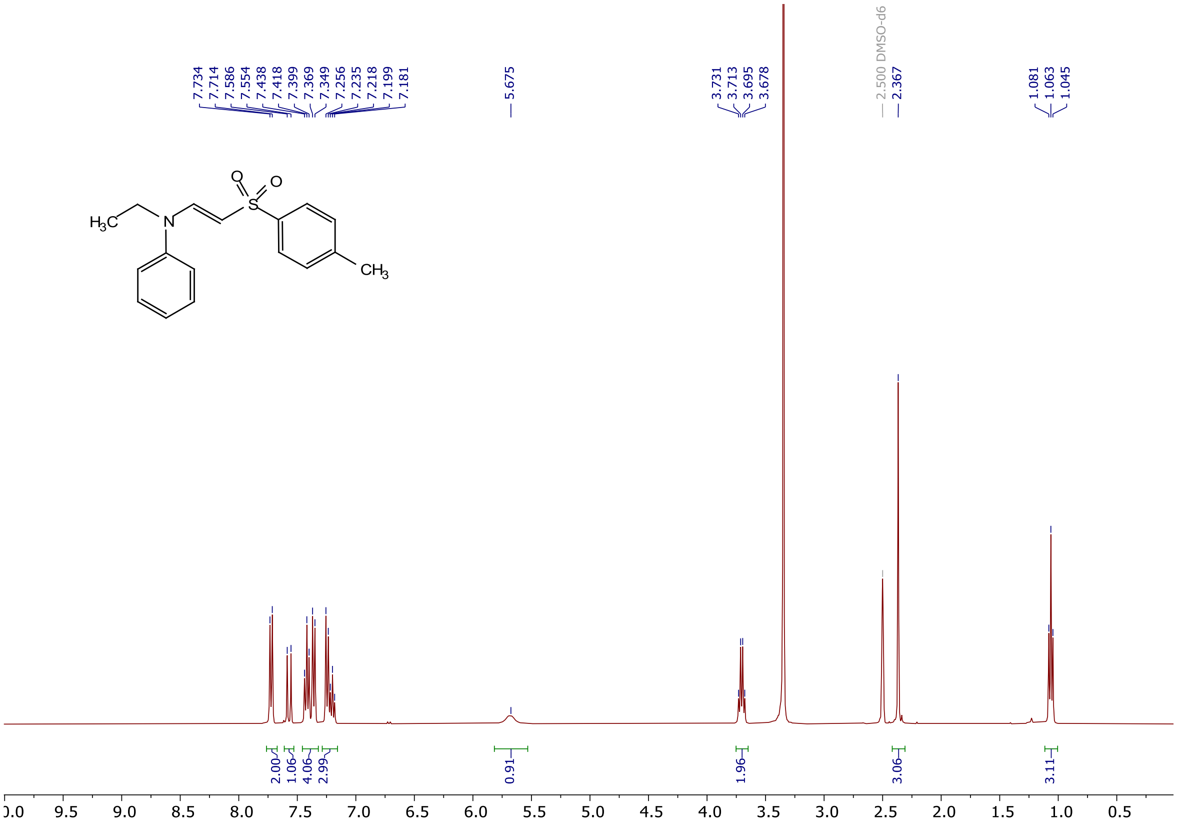

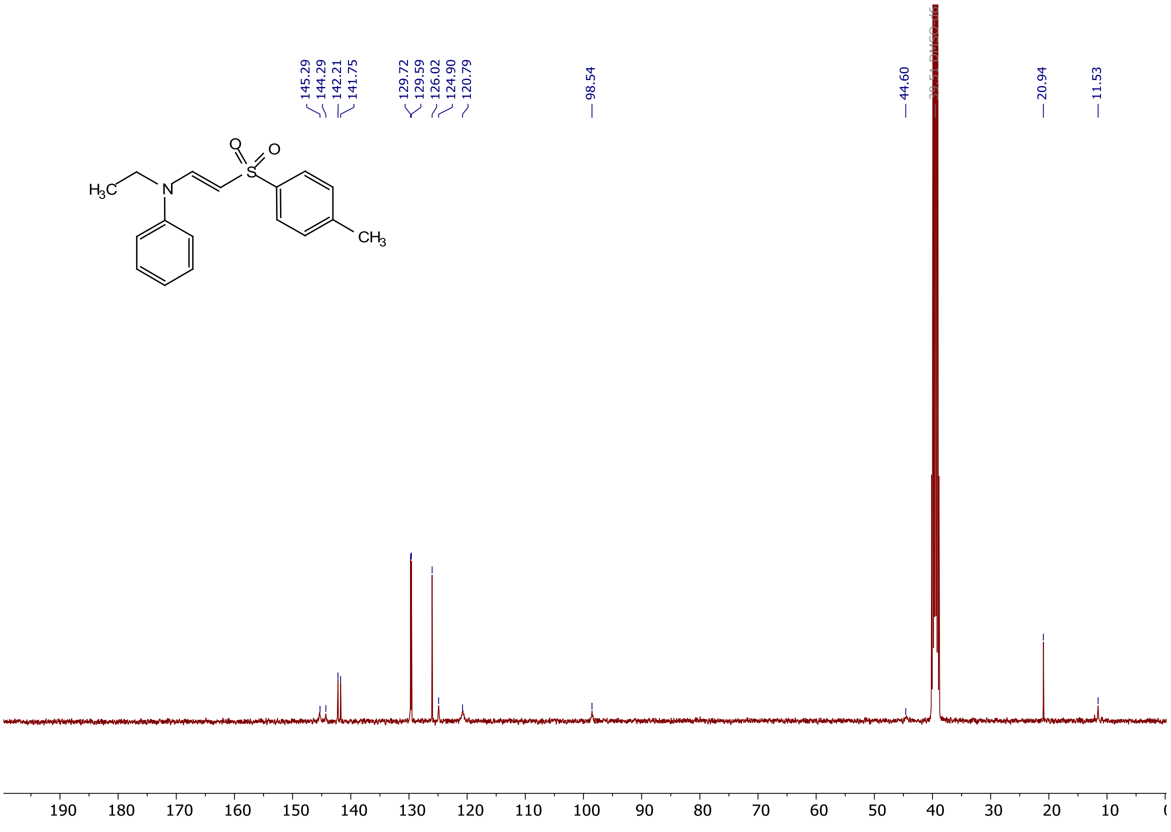

(*E*)-*N*,*N*-Diethyl-2-tosylethen-1-amine (30)

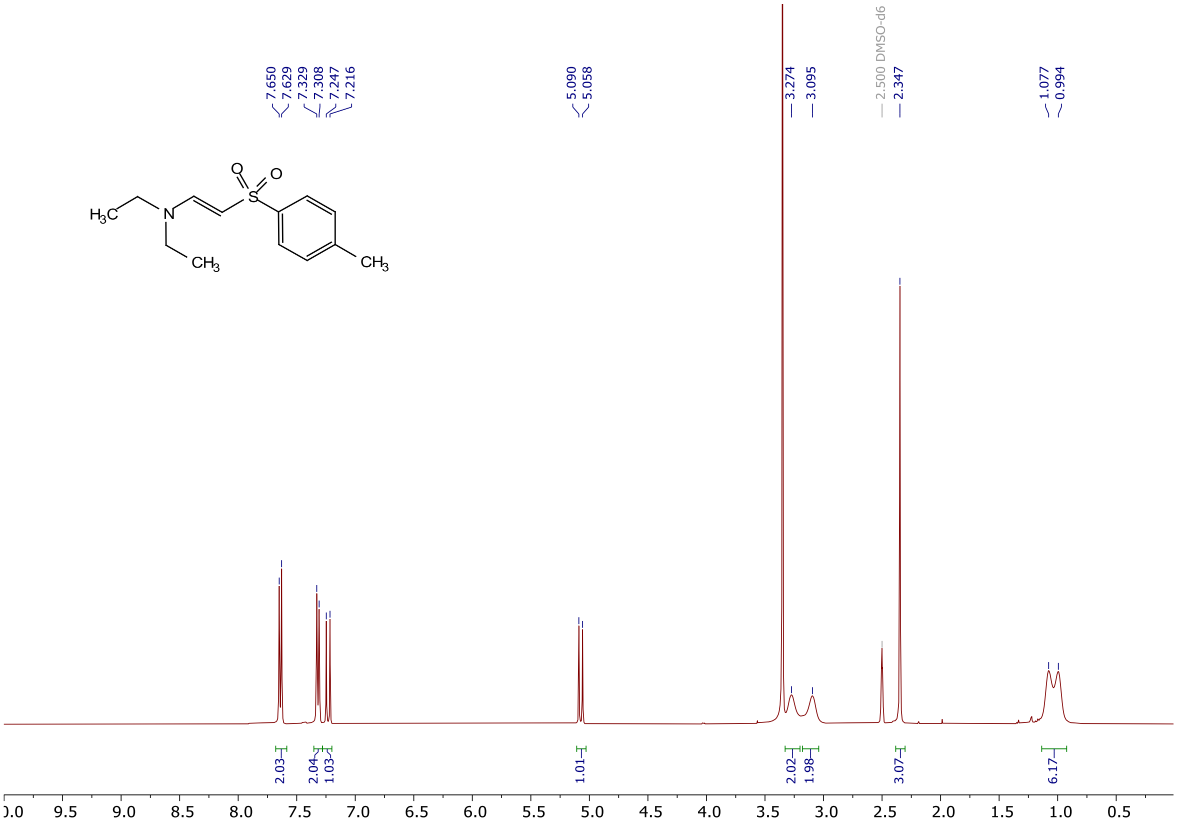

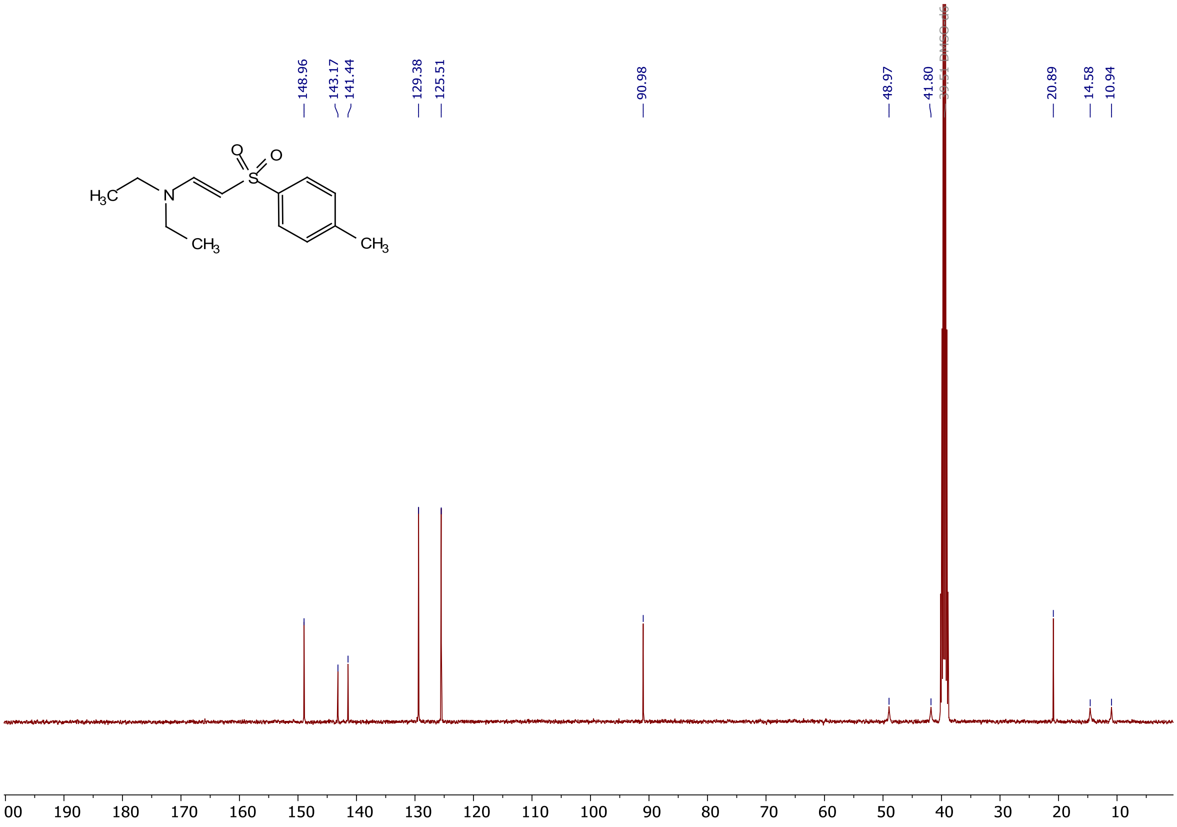

(*E*)-*N*,*N*-bis(ethyl-d5)-2-tosylethen-1-amine-1-d (30-D)

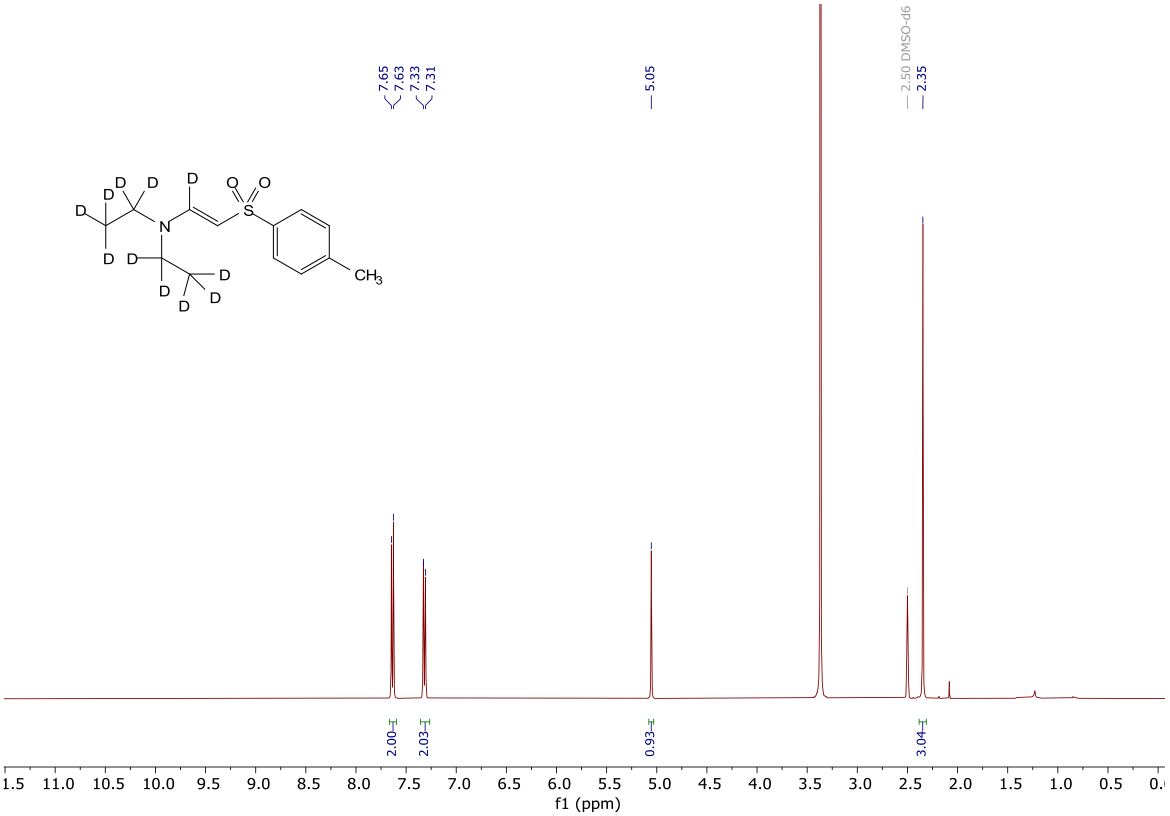

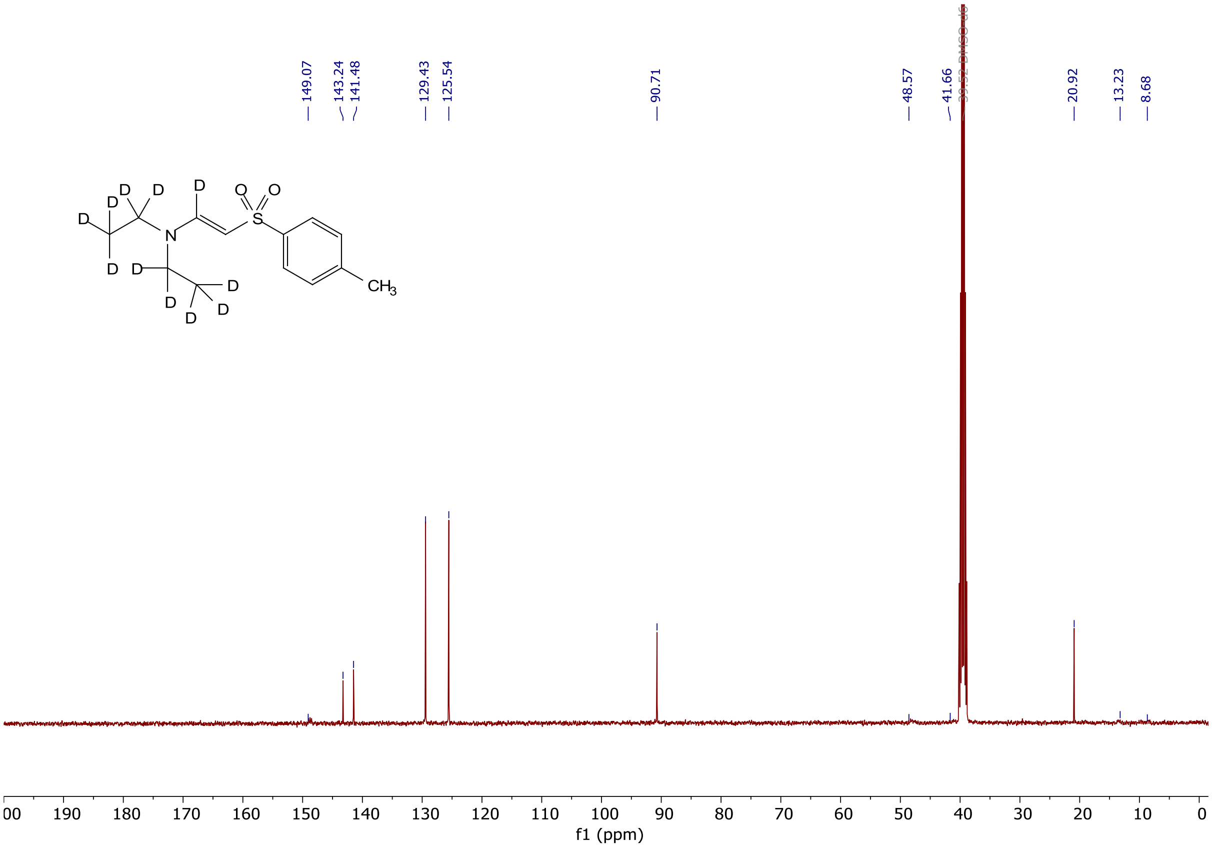

(*E*)-*N*,*N*-Dibutyl-2-tosylbut-1-en-1-amine (31)

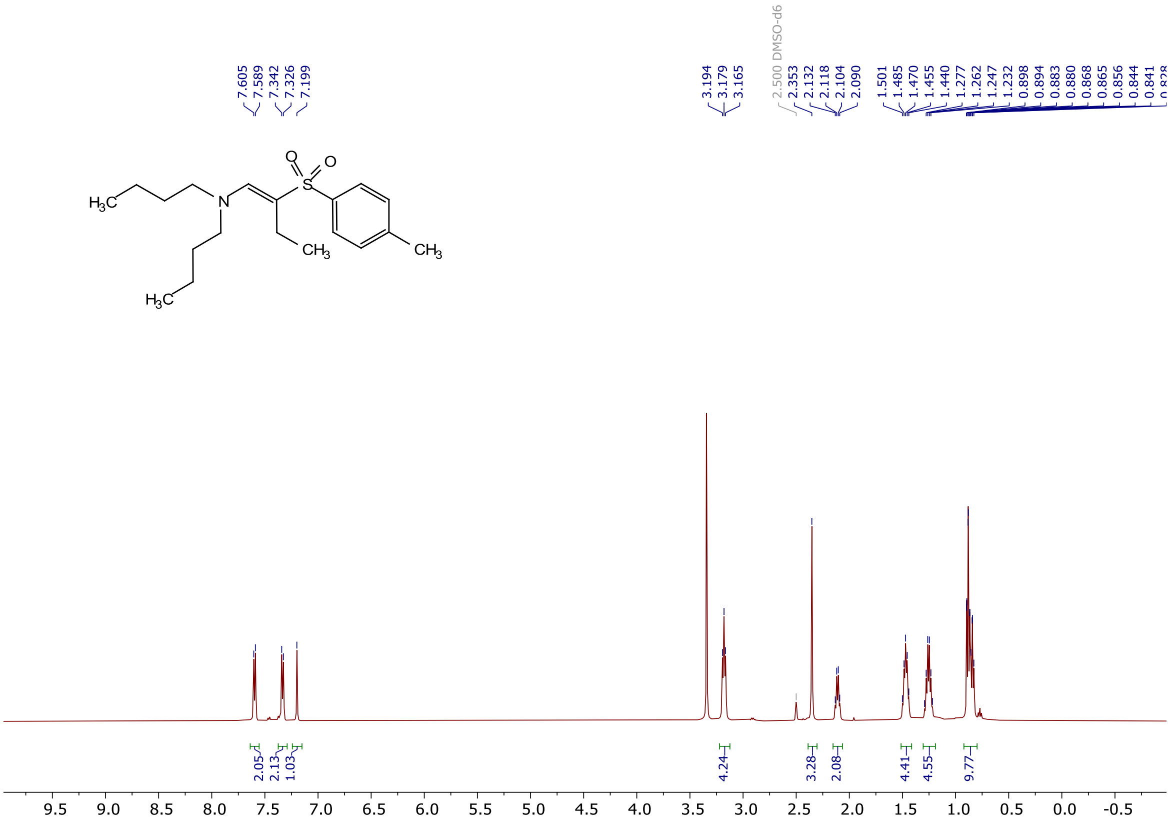

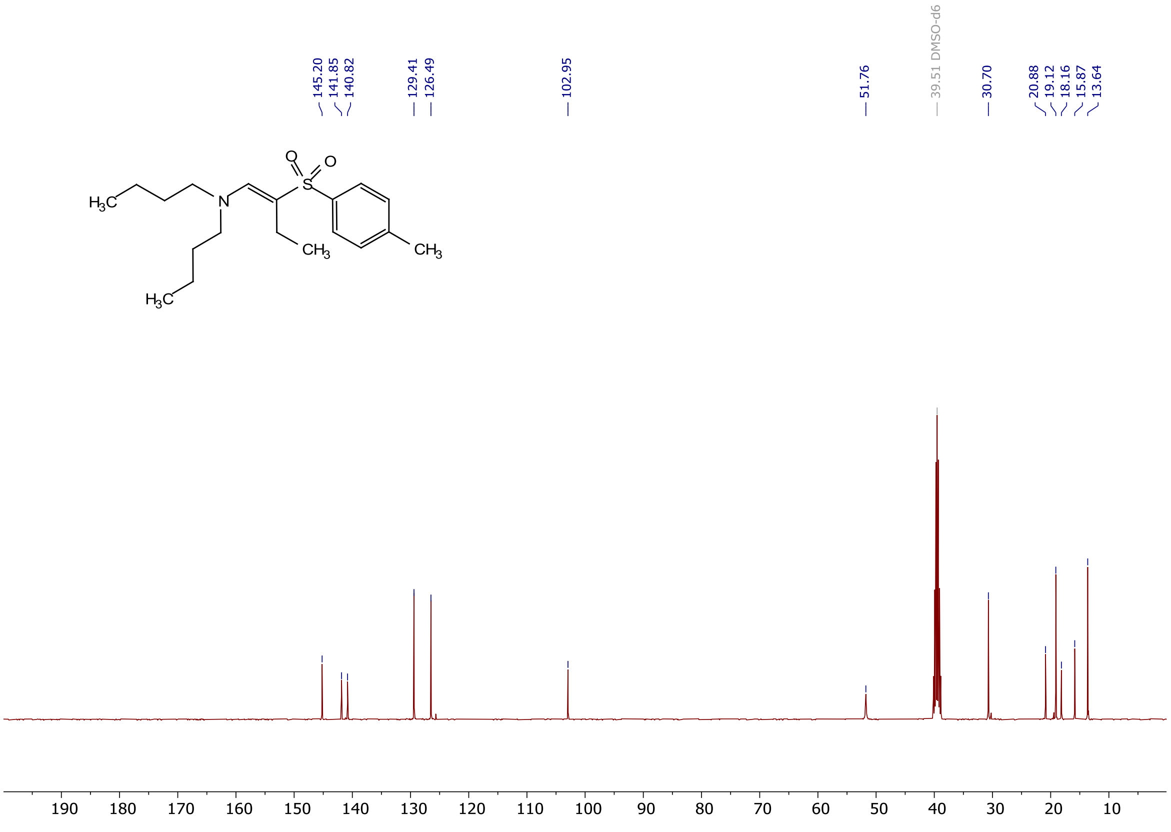

(*E*)-*N*,*N*-Dihexyl-2-tosylhex-1-en-1-amine (32)

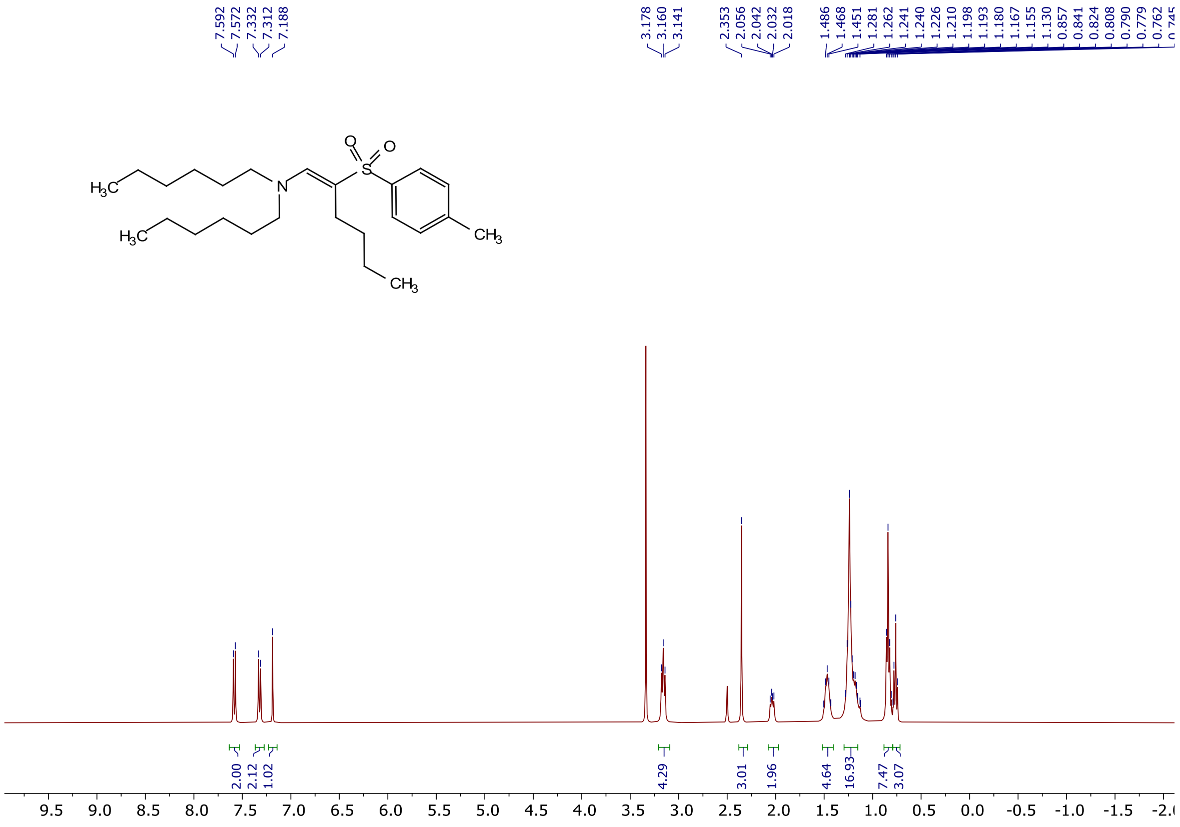

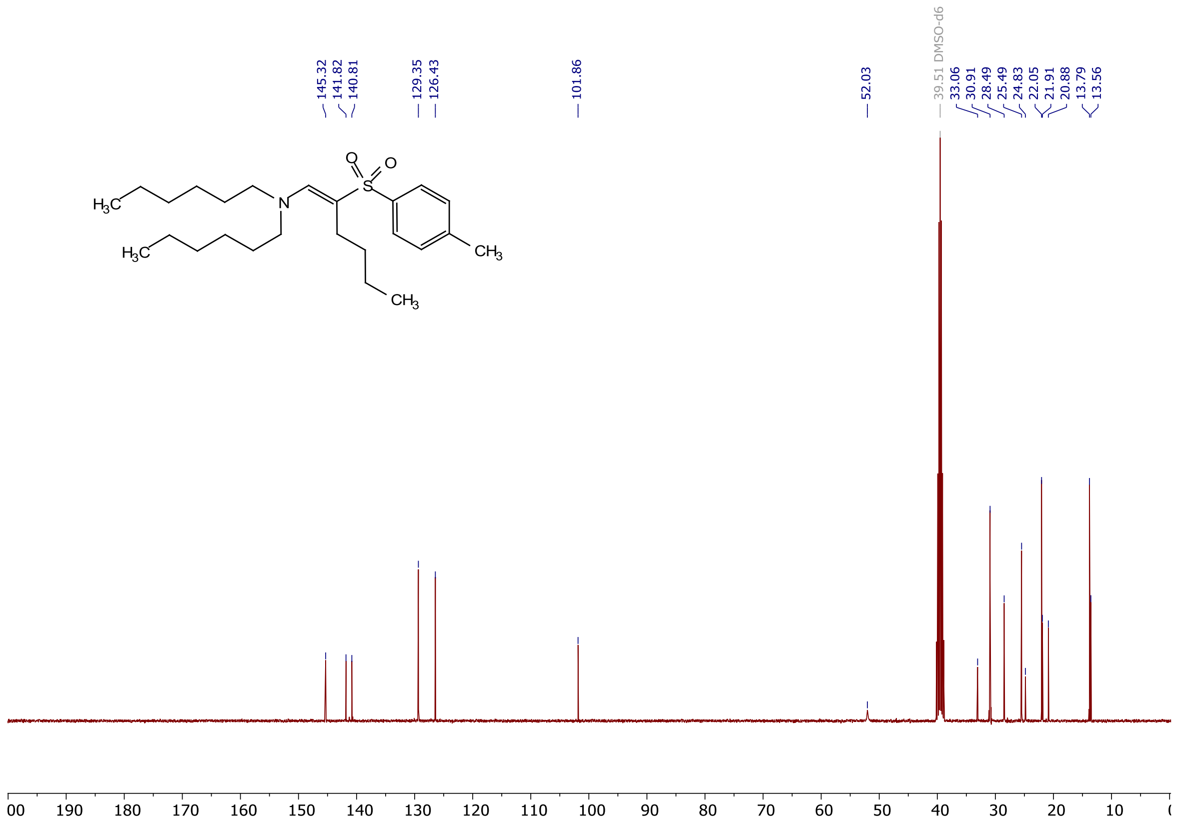

(*E*)-*N*-Isopropyl-*N*-(2-tosylvinyl)propan-2-amine (33)

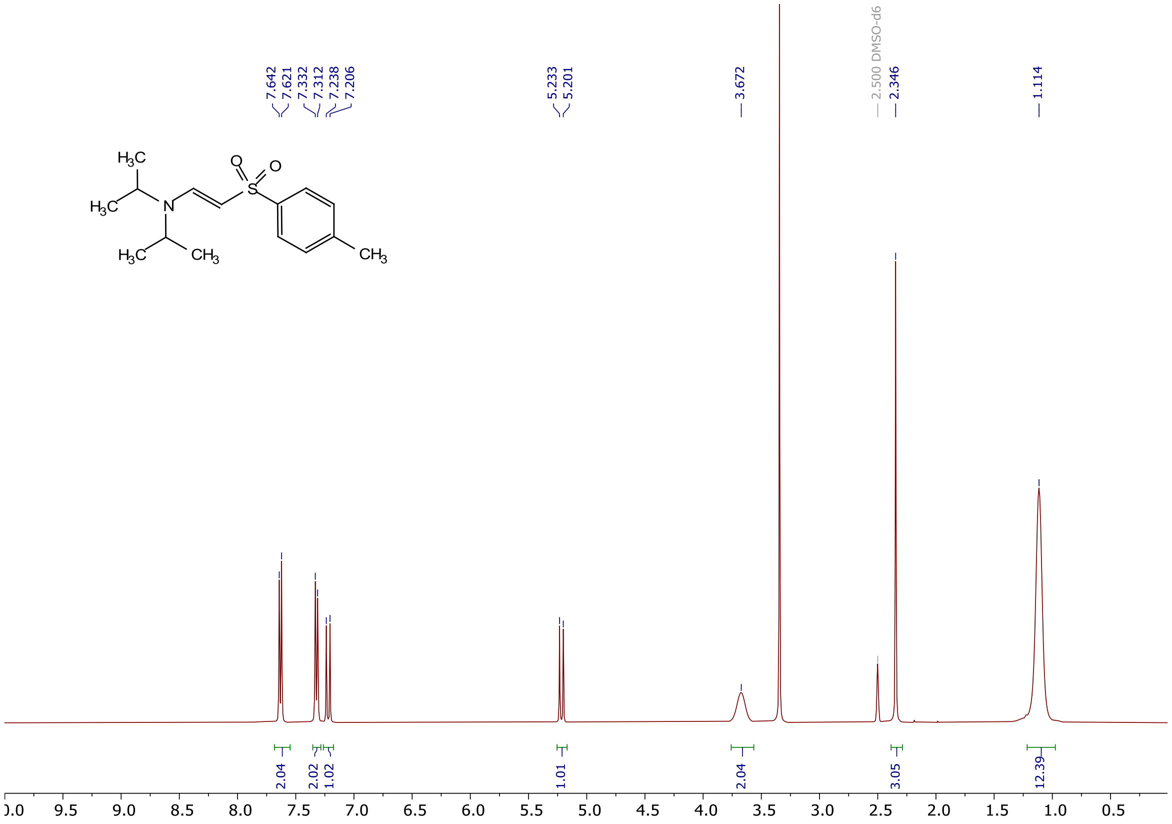

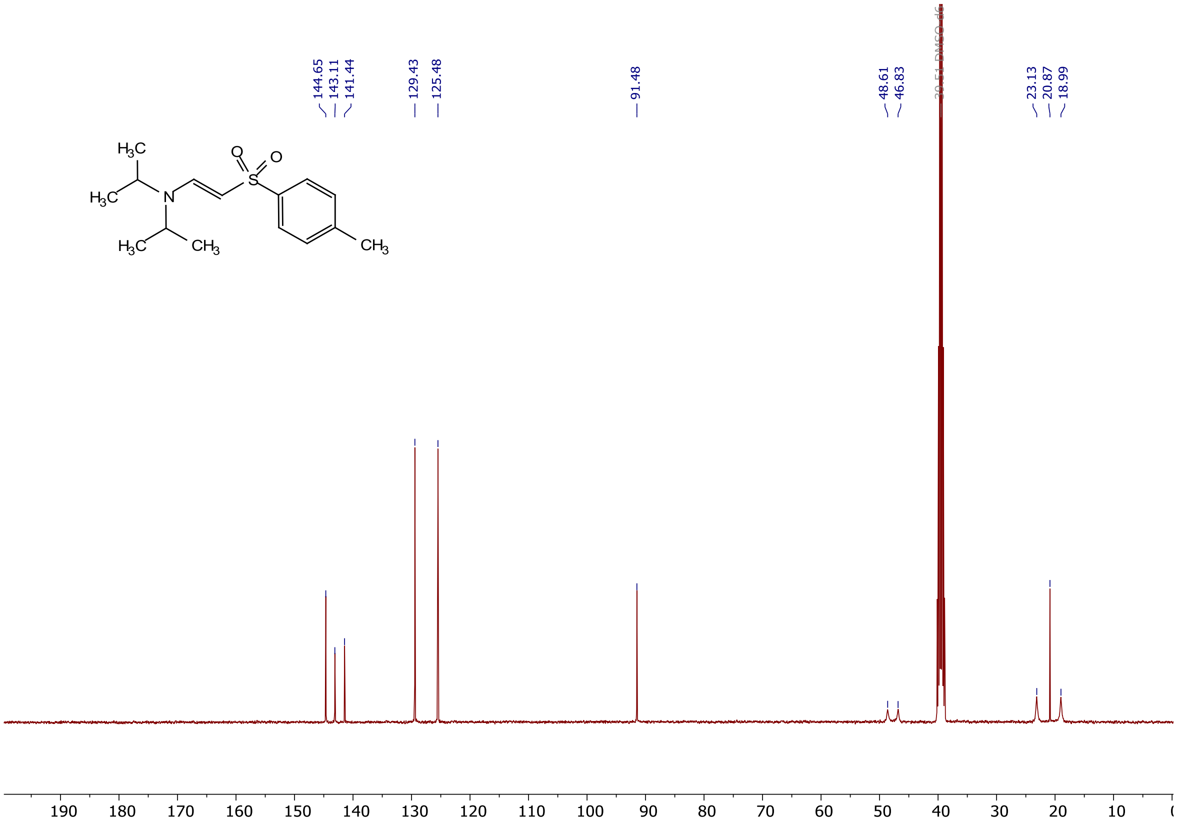

(*E*)-*N*-Ethyl-*N*-methyl-2-tosylethen-1-amine (34)

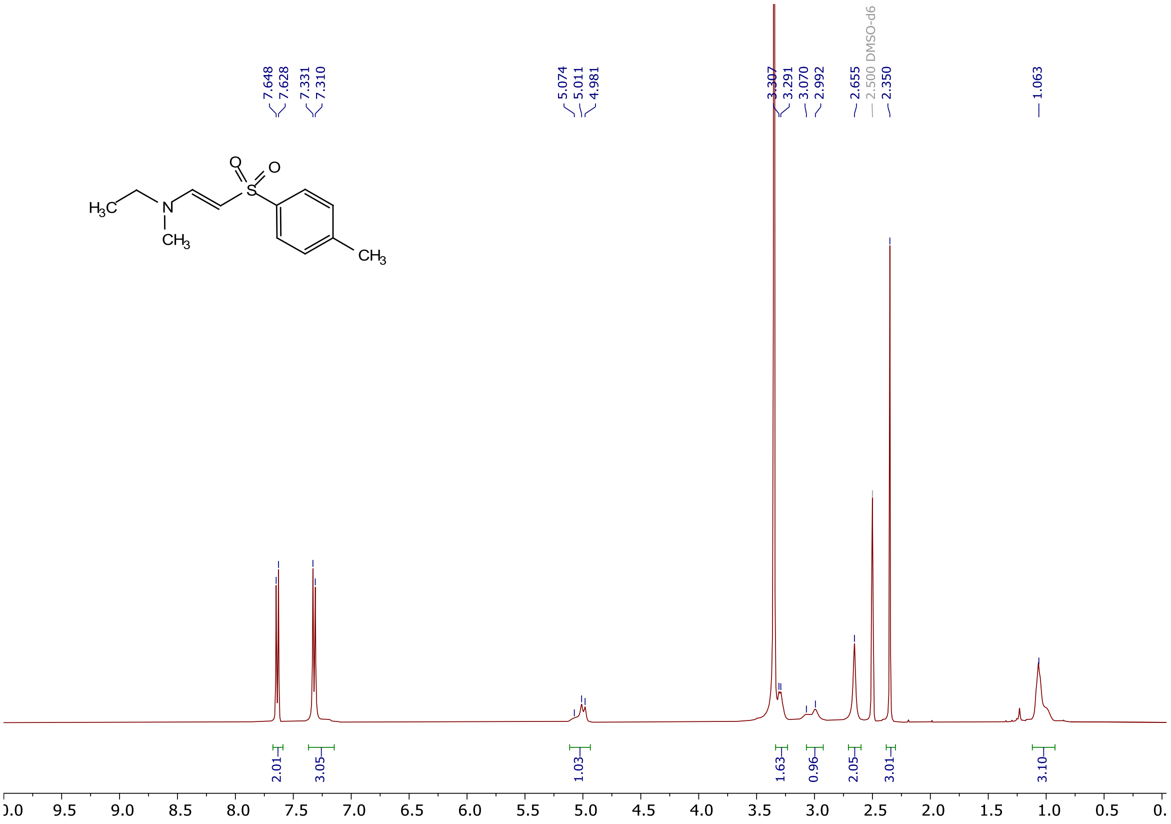

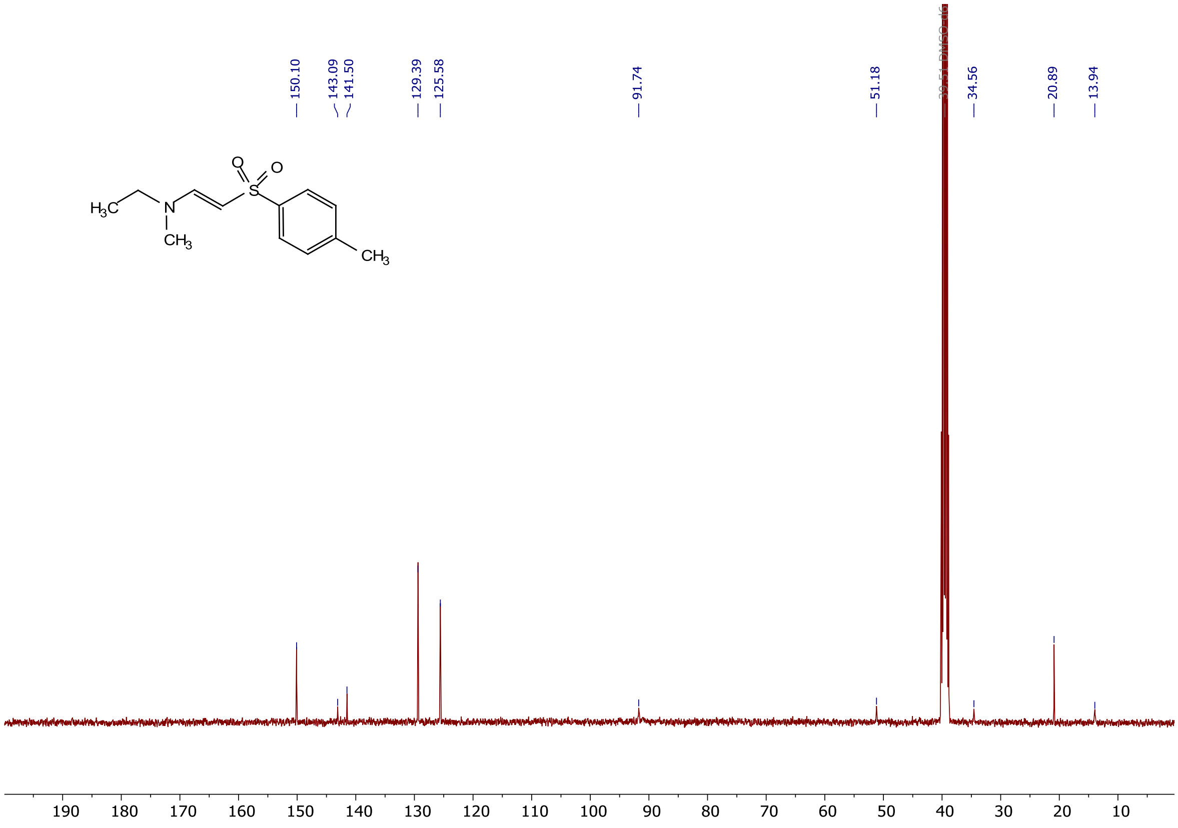

1-Methyl-5-tosyl-1,2,3,4-tetrahydropyridine (35)

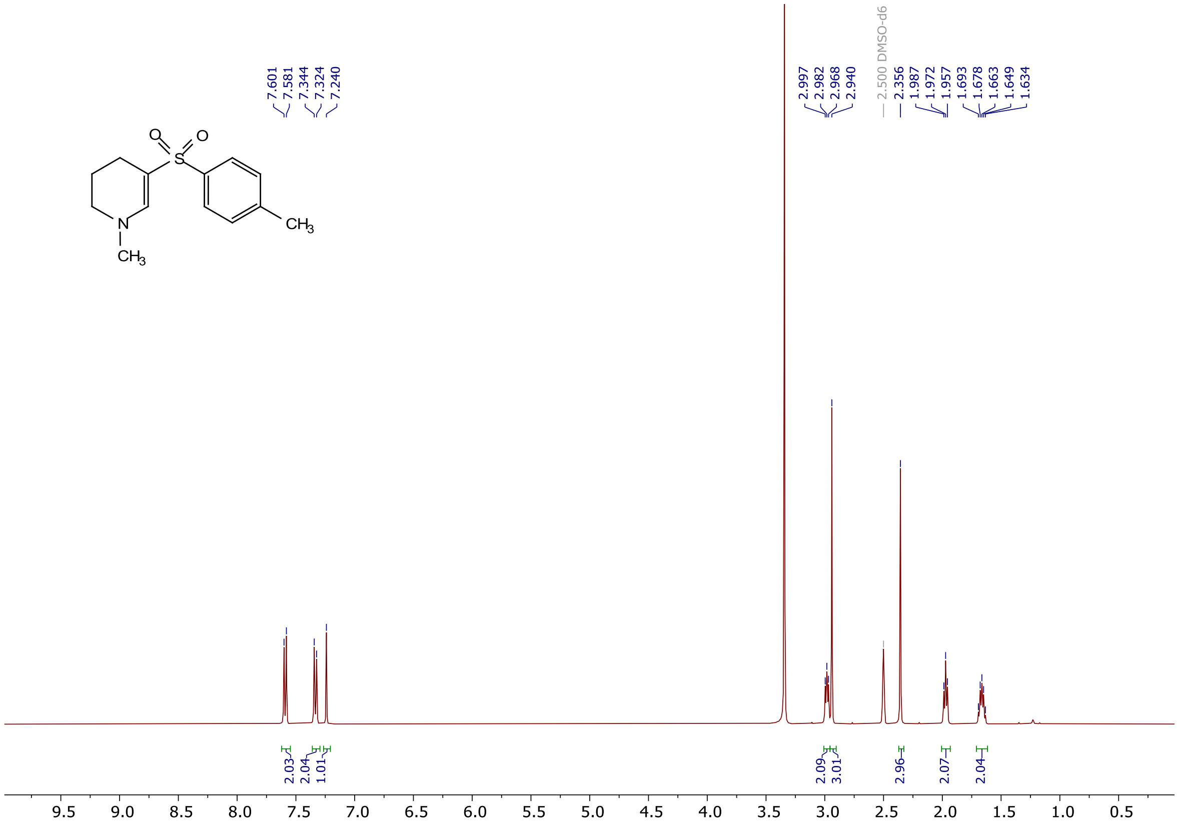

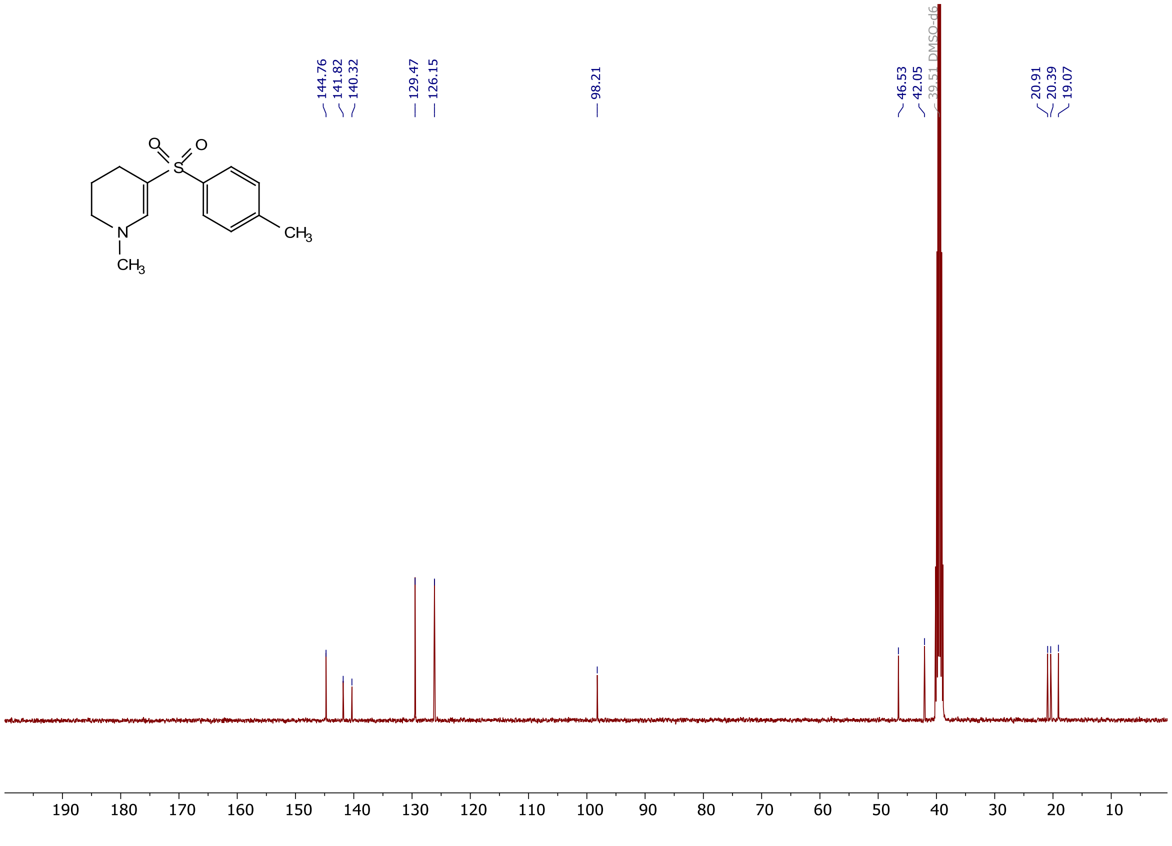

1-Ethyl-5-tosyl-1,2,3,4-tetrahydropyridine (36)

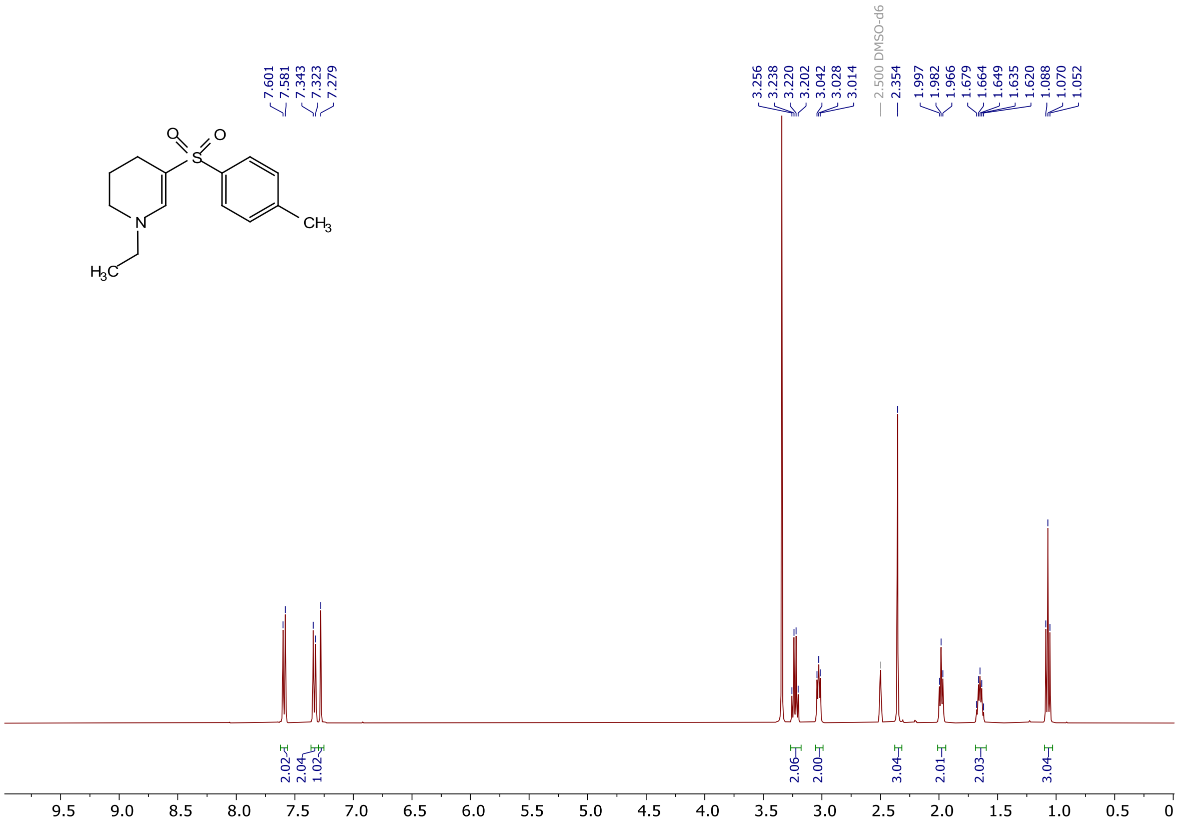

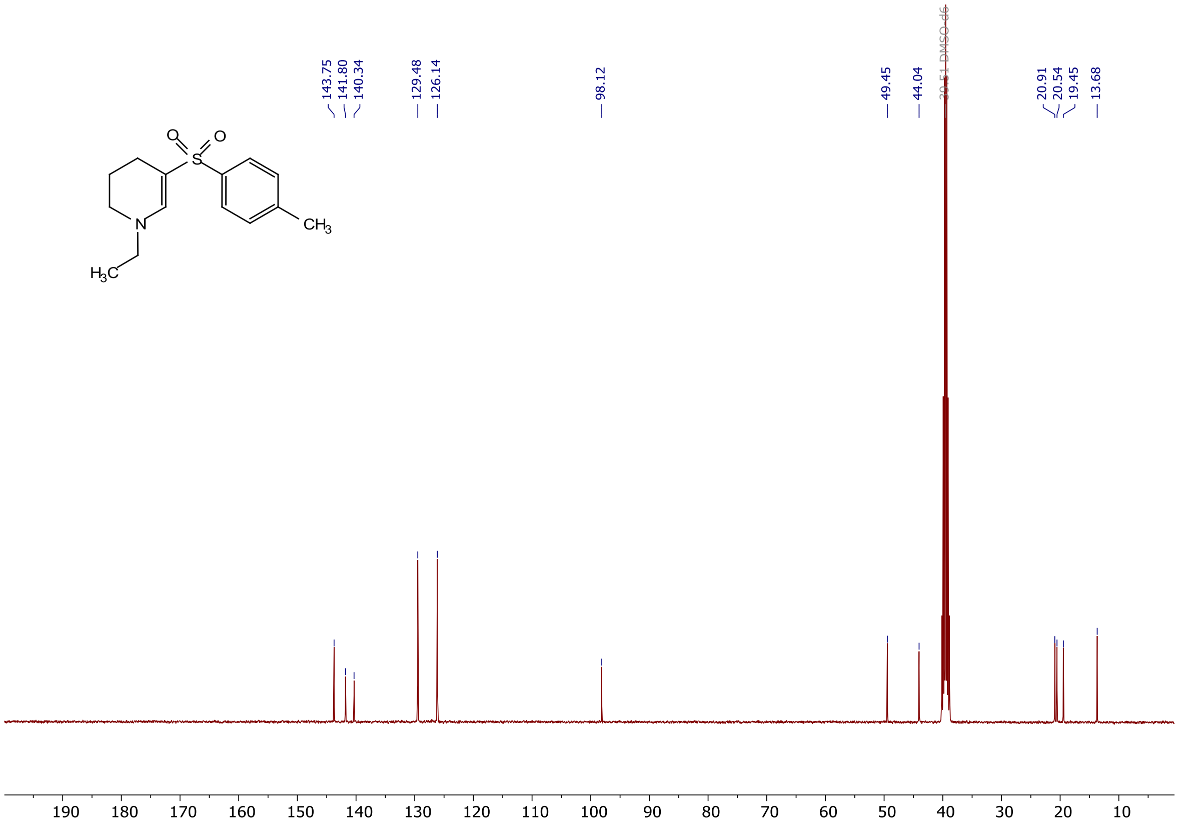

1-(1-Phenylethyl)-6-tosyl-2,3,4,5-tetrahydro-1*H*-azepine (37)

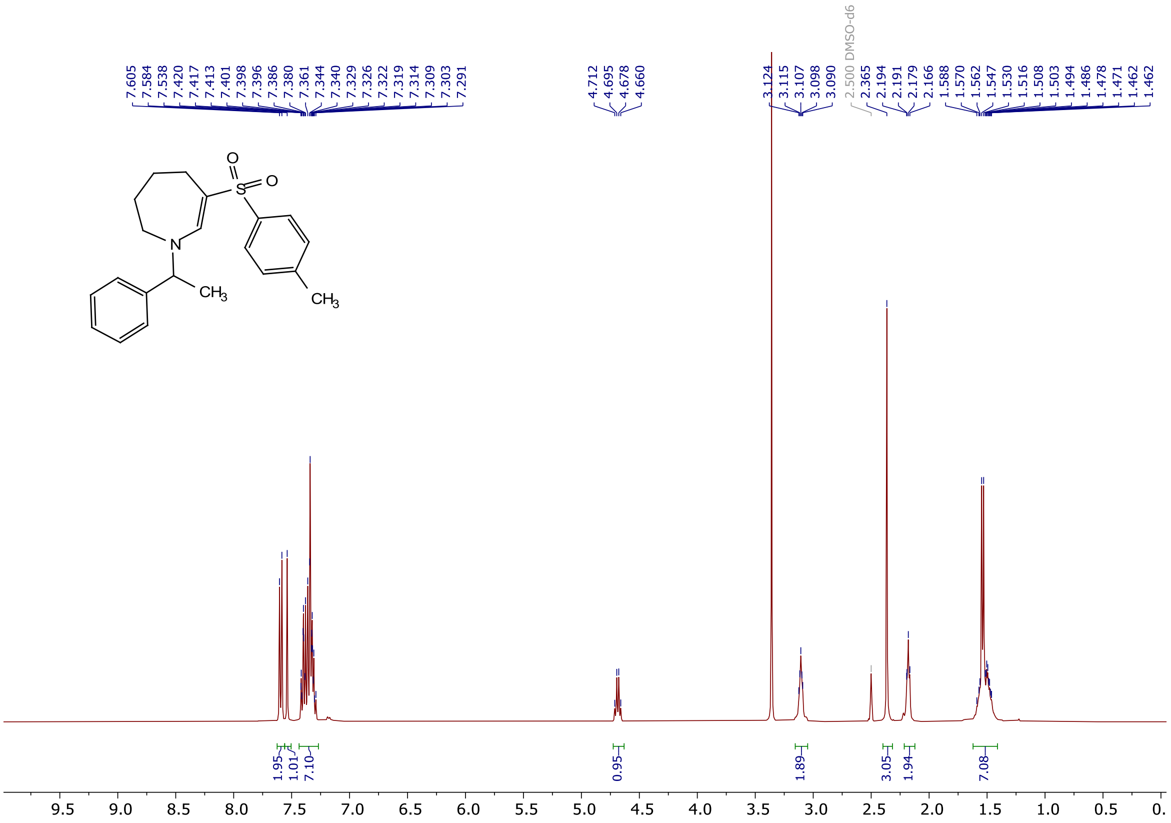

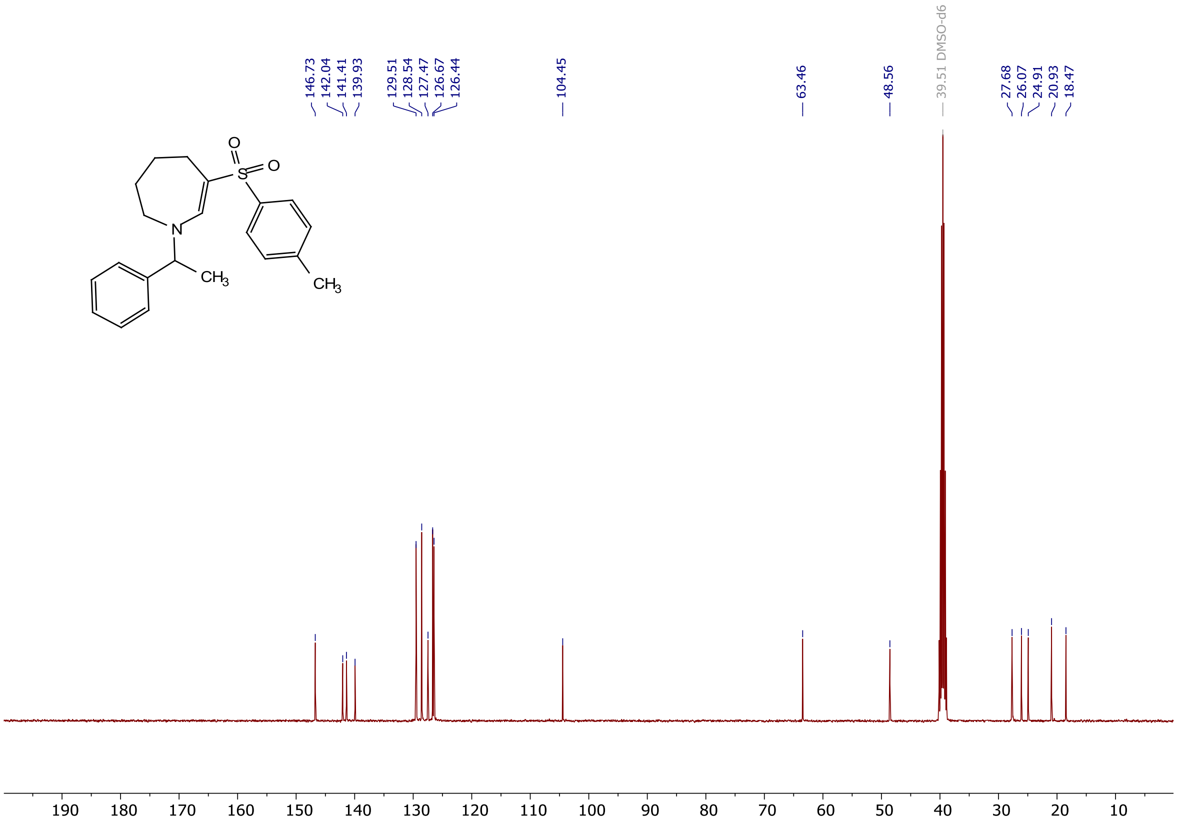

1-(1-(Naphthalen-1-yl)ethyl)-6-tosyl-2,3,4,5-tetrahydro-1*H*-azepine (38)

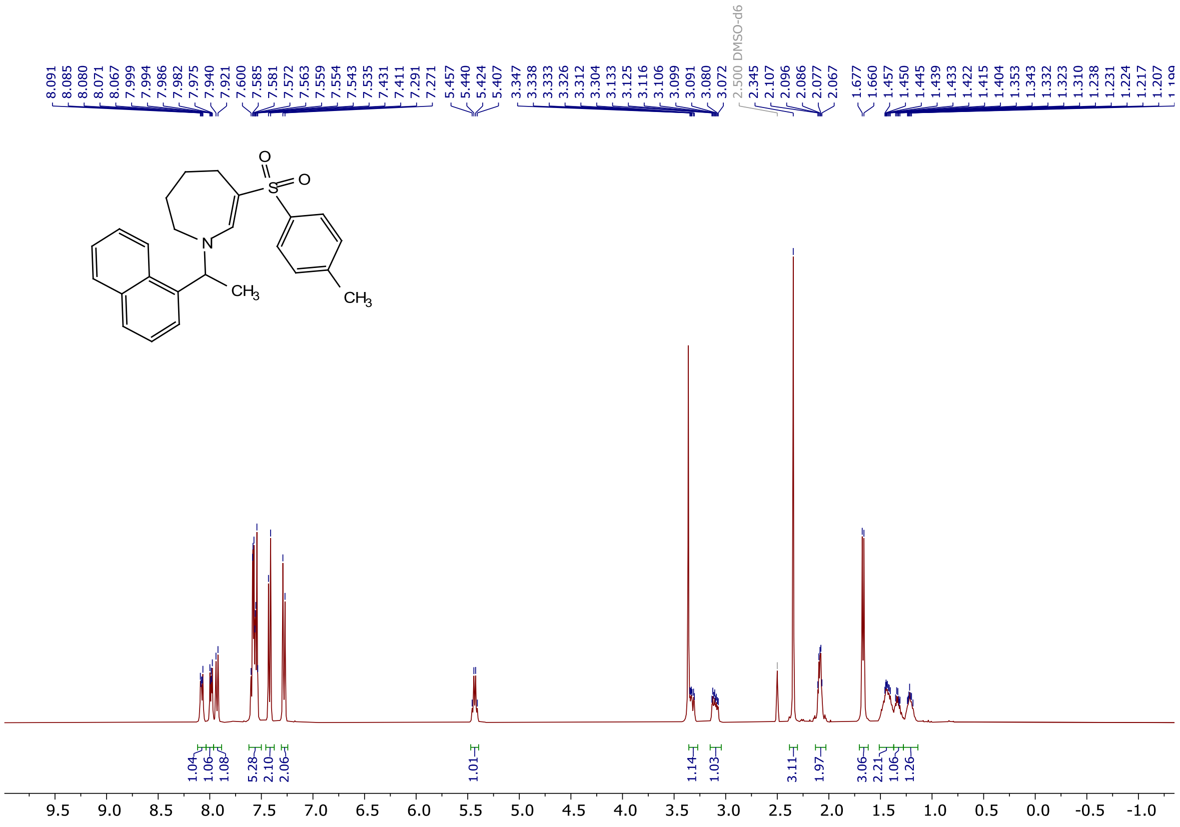

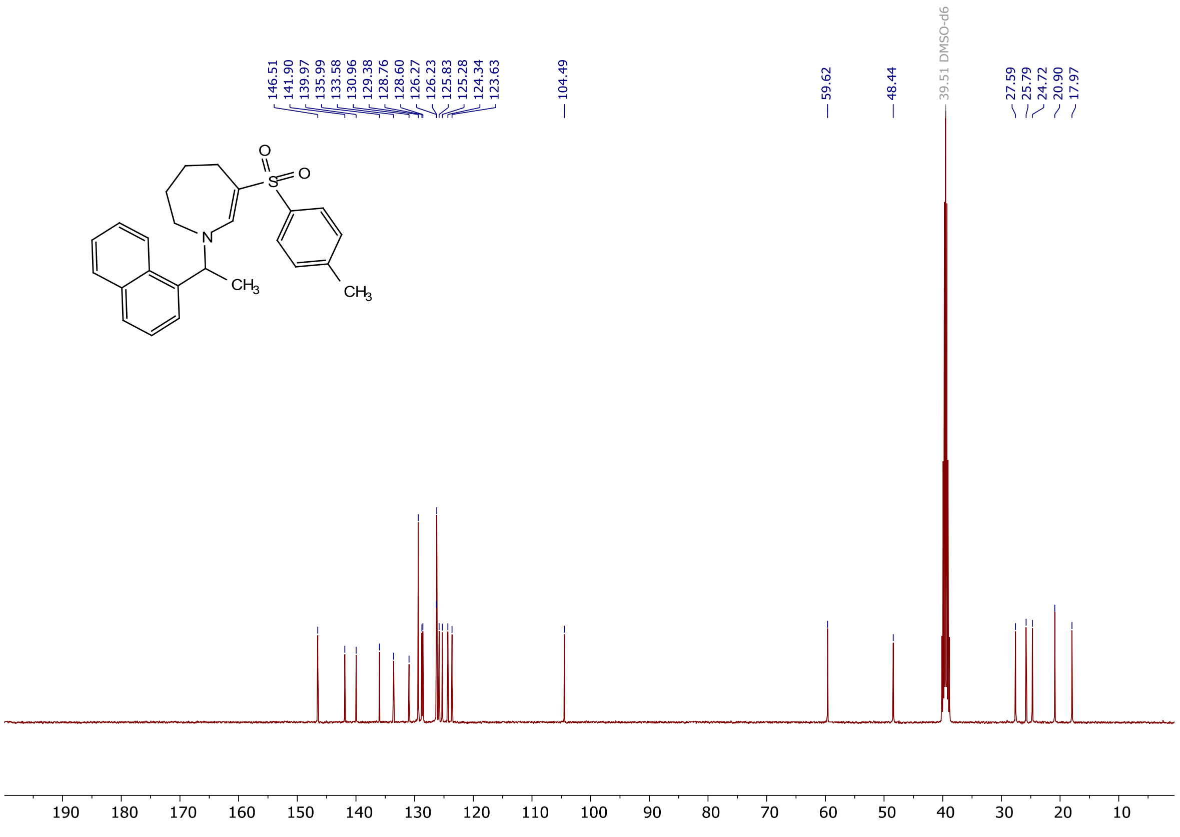

1-(1-Phenylethyl)-5-tosyl-1,2,3,4-tetrahydropyridine (39)

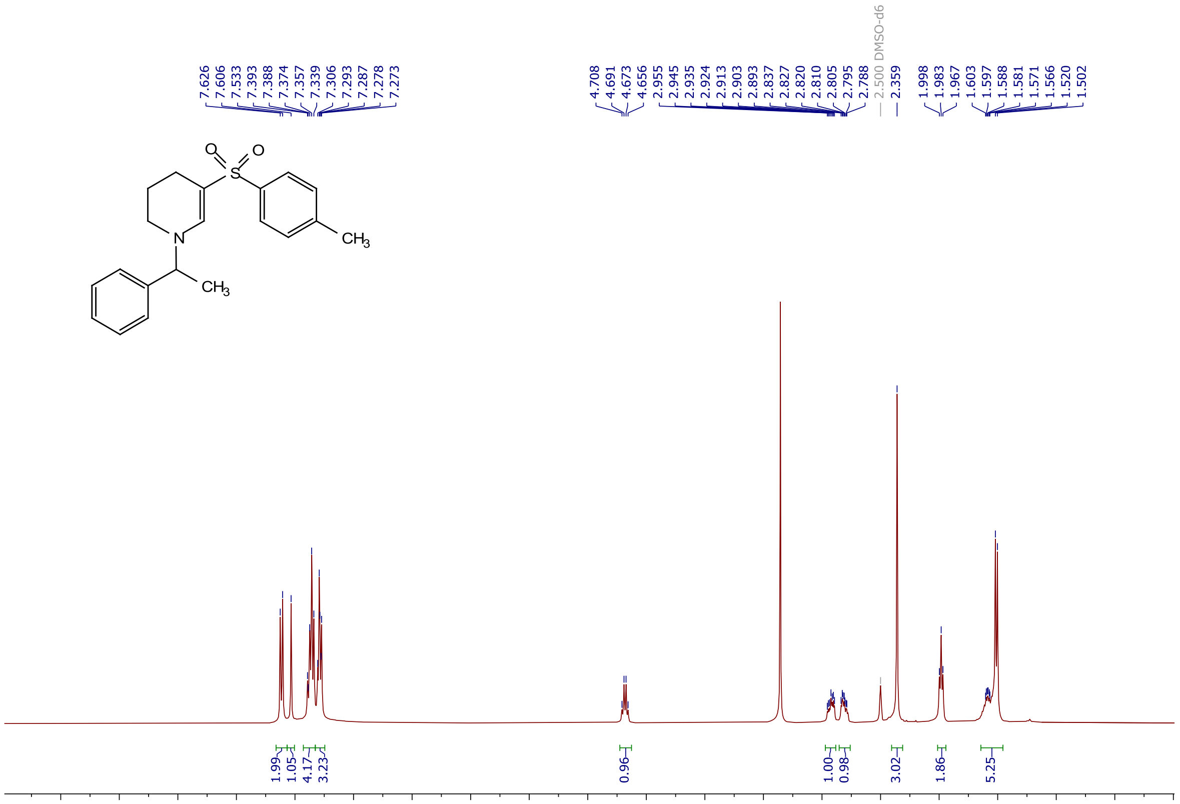

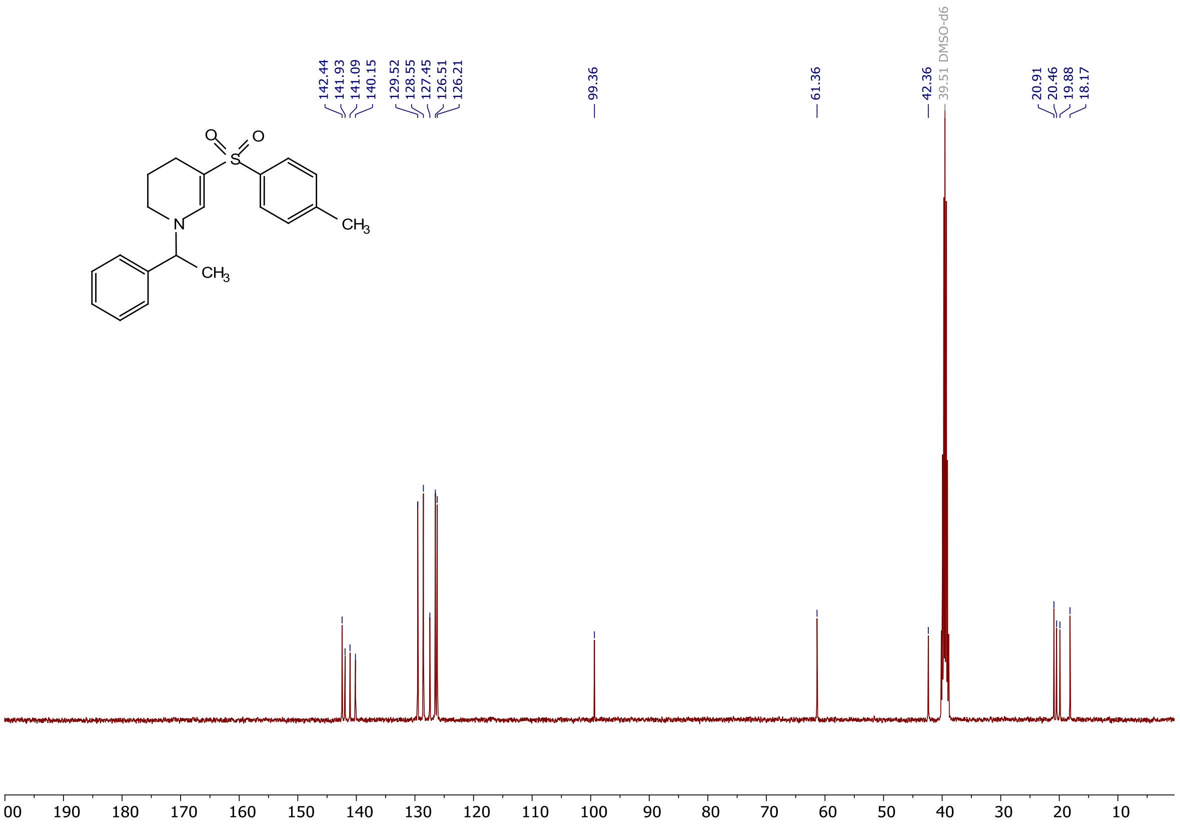

(*E*)-1-(1-Phenyl-2-tosylvinyl)piperidine (39')

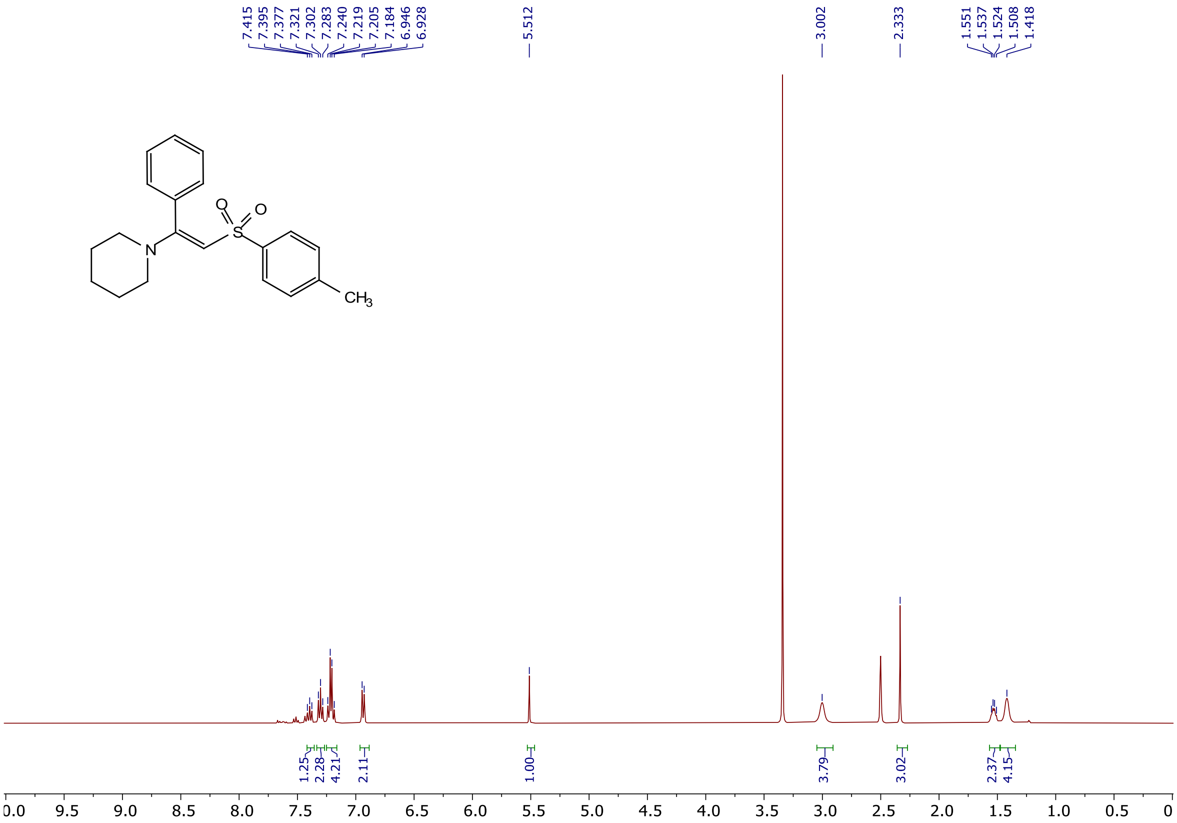

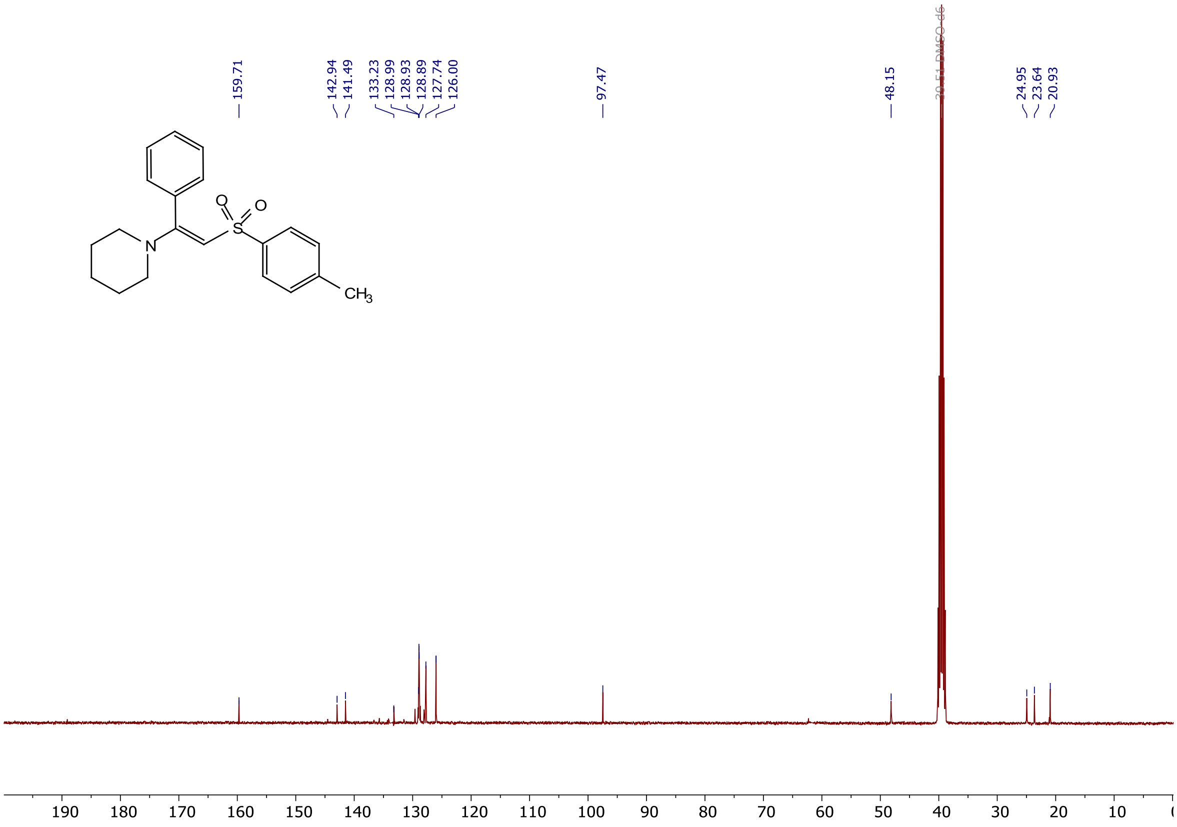

1-(1-Phenylethyl)-4-tosyl-2,3-dihydro-1*H*-pyrrole (40)

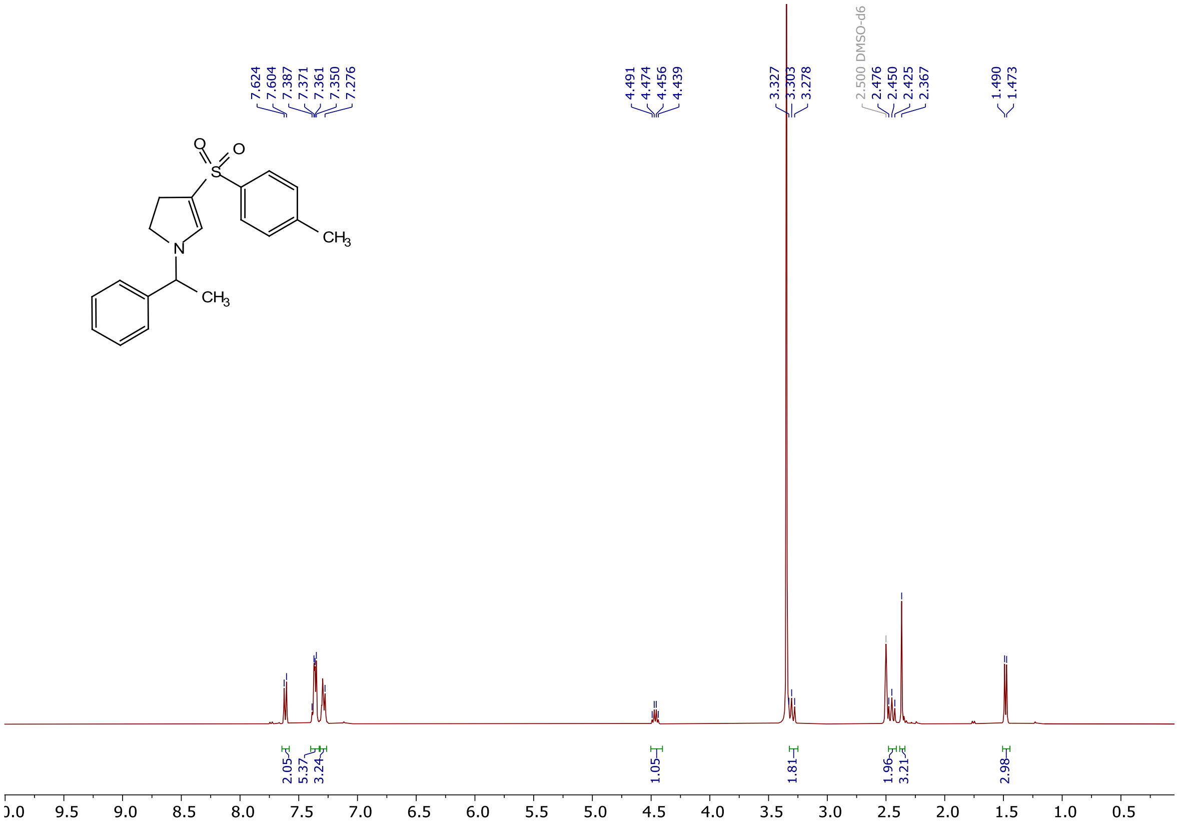

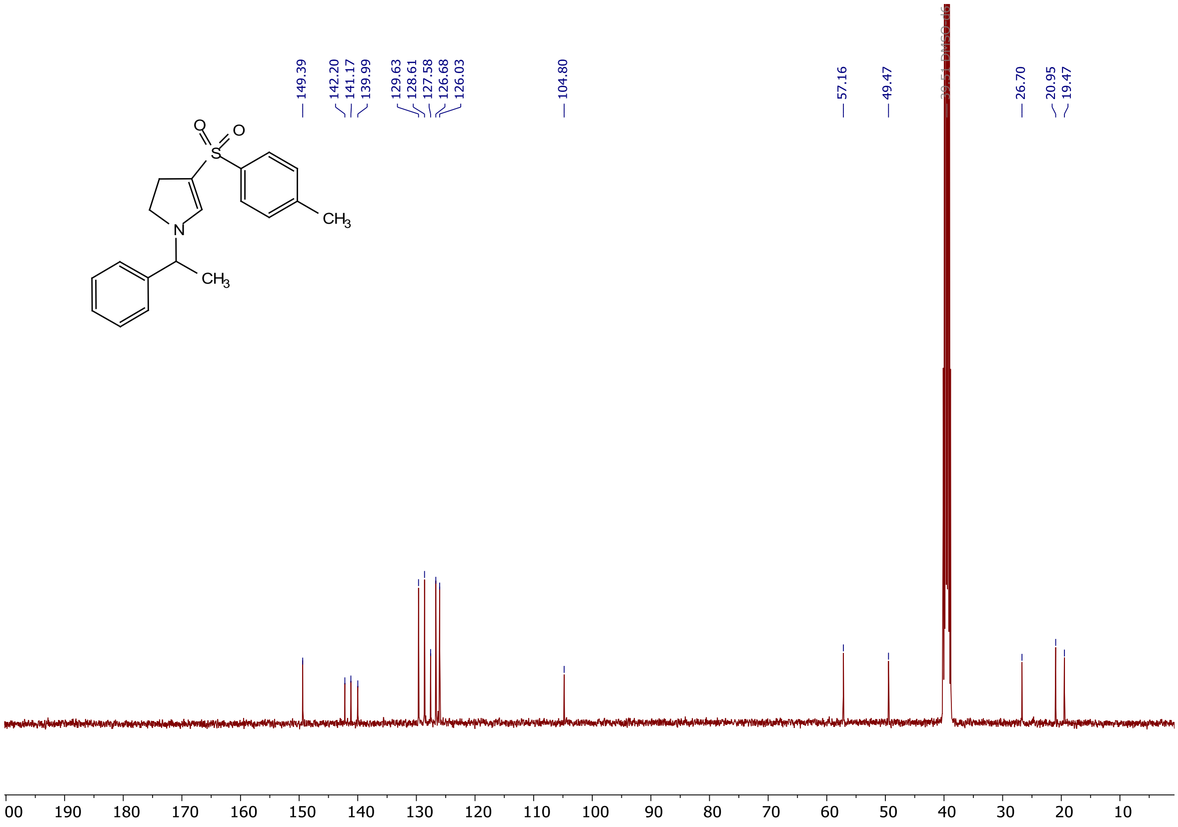

(*E*)-1-(1-Phenyl-2-tosylvinyl)pyrrolidine (40')

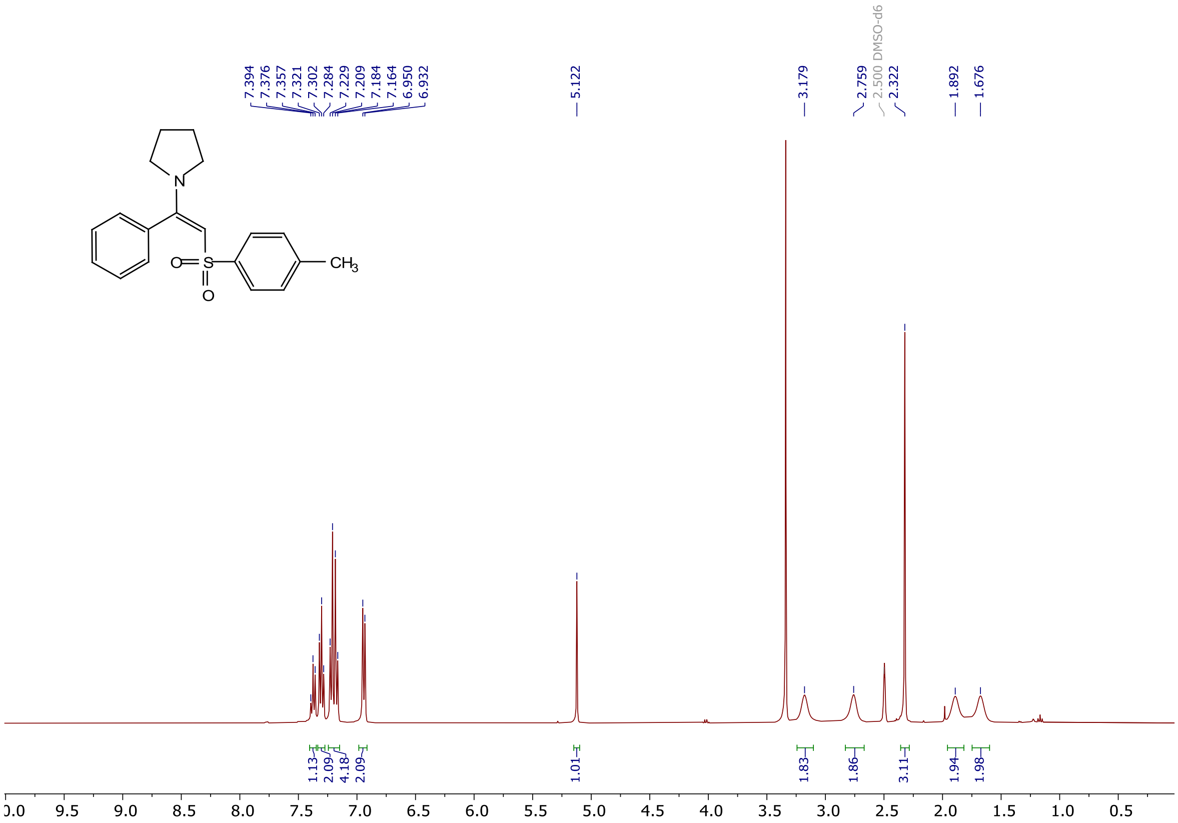

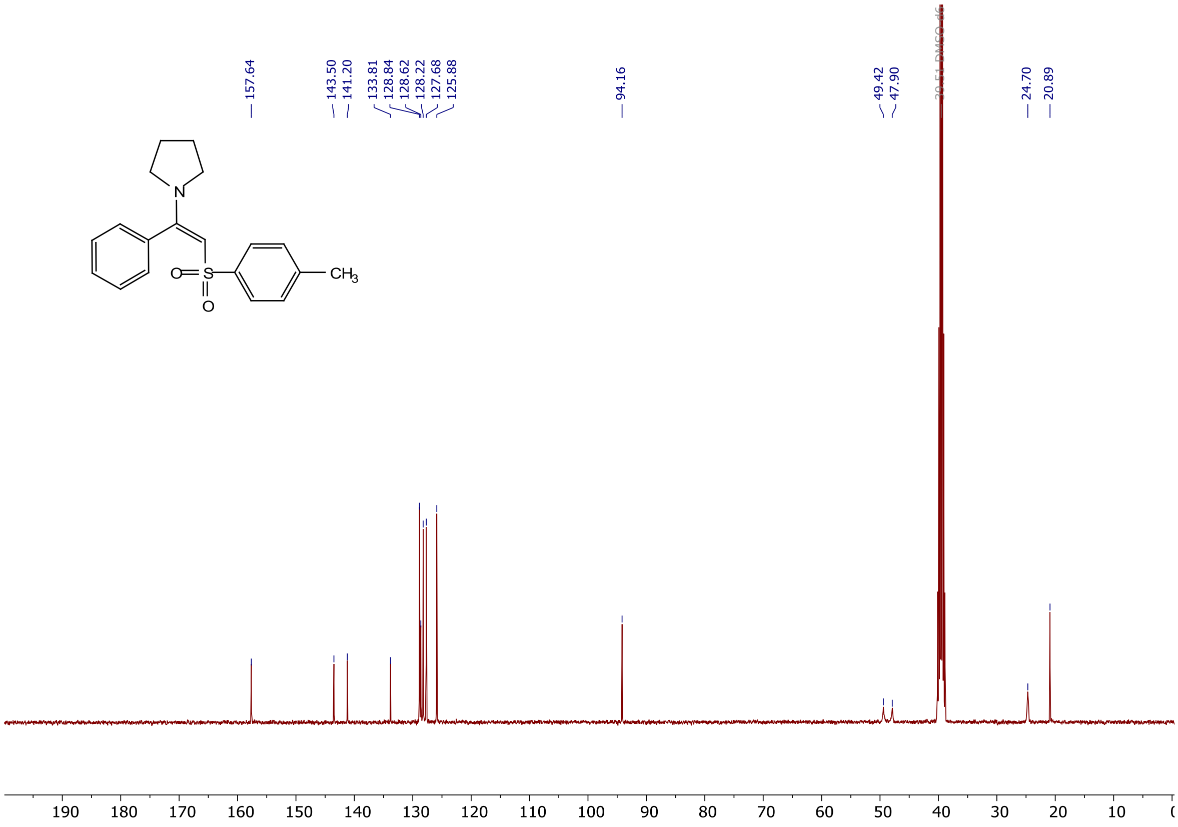

(*E*)-*N*-Ethyl-*N*-(1-phenylethyl)-2-tosylethen-1-amine (41)

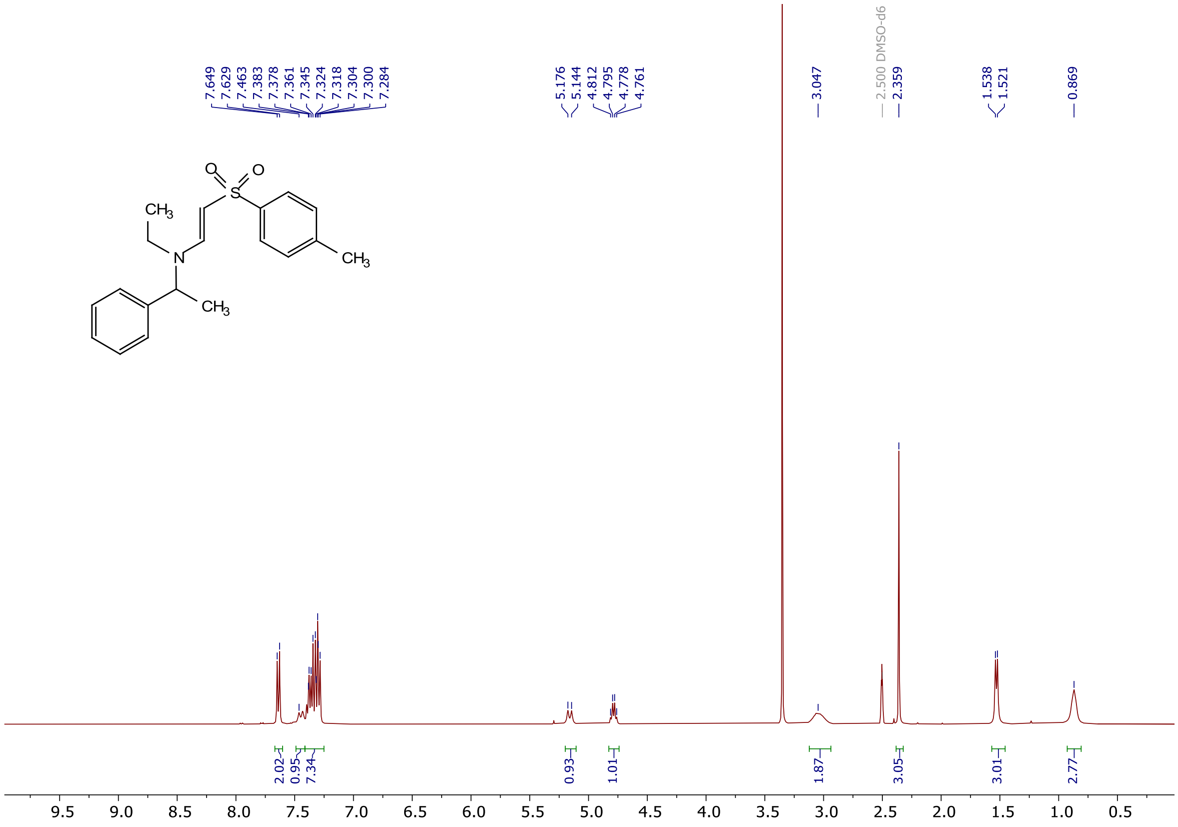

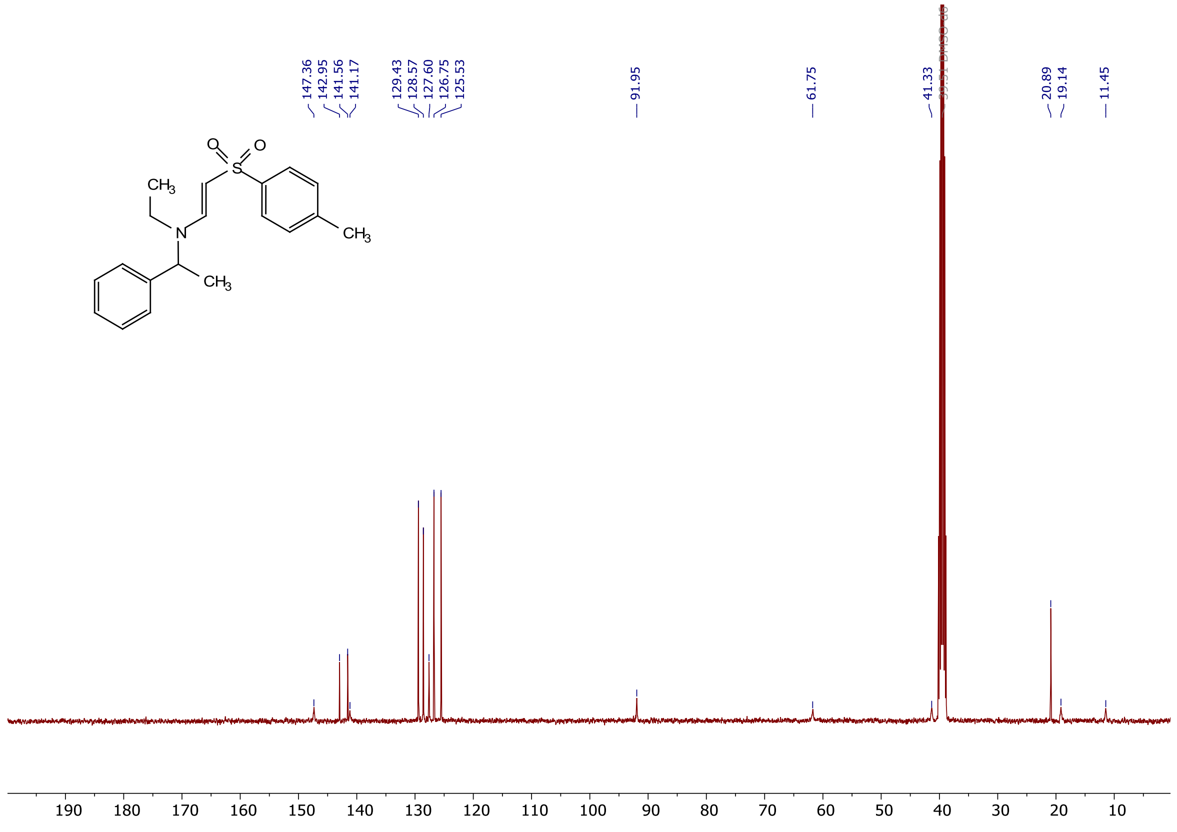

(*E*)-*N*,*N*-Diethyl-1-phenyl-2-tosylethen-1-amine (41')

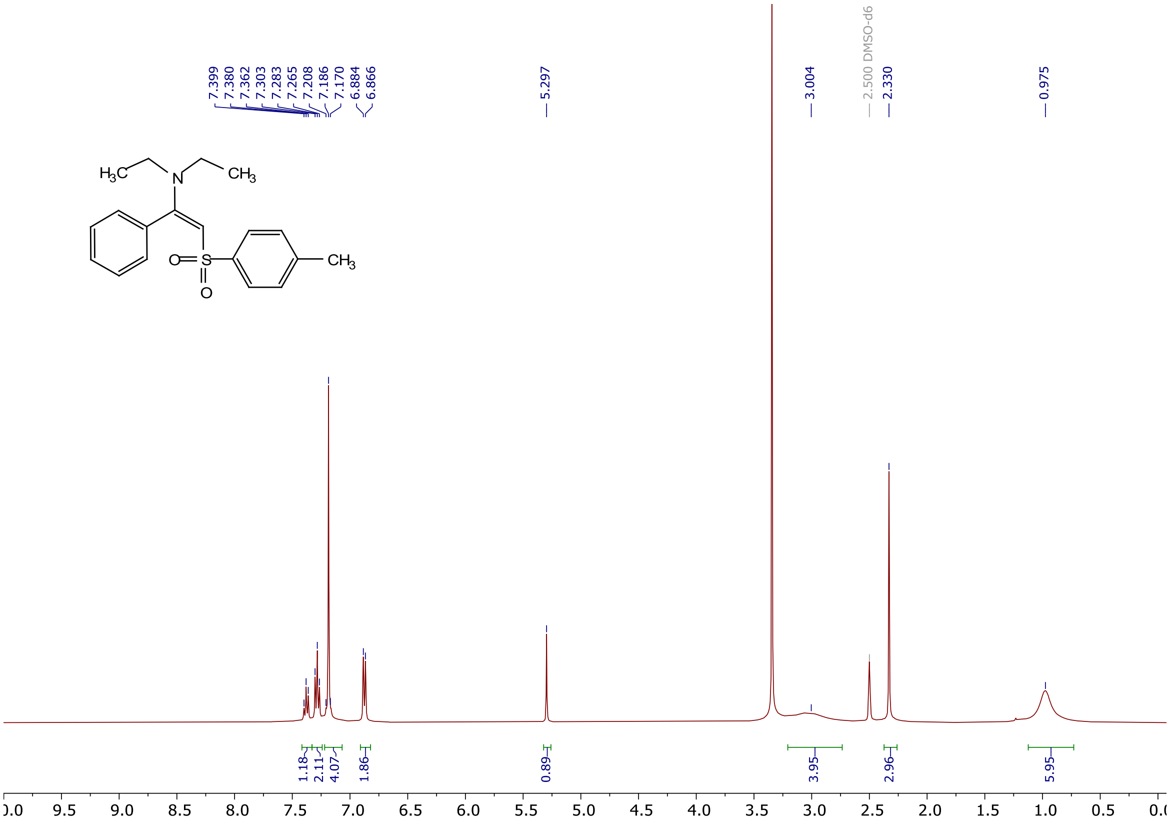

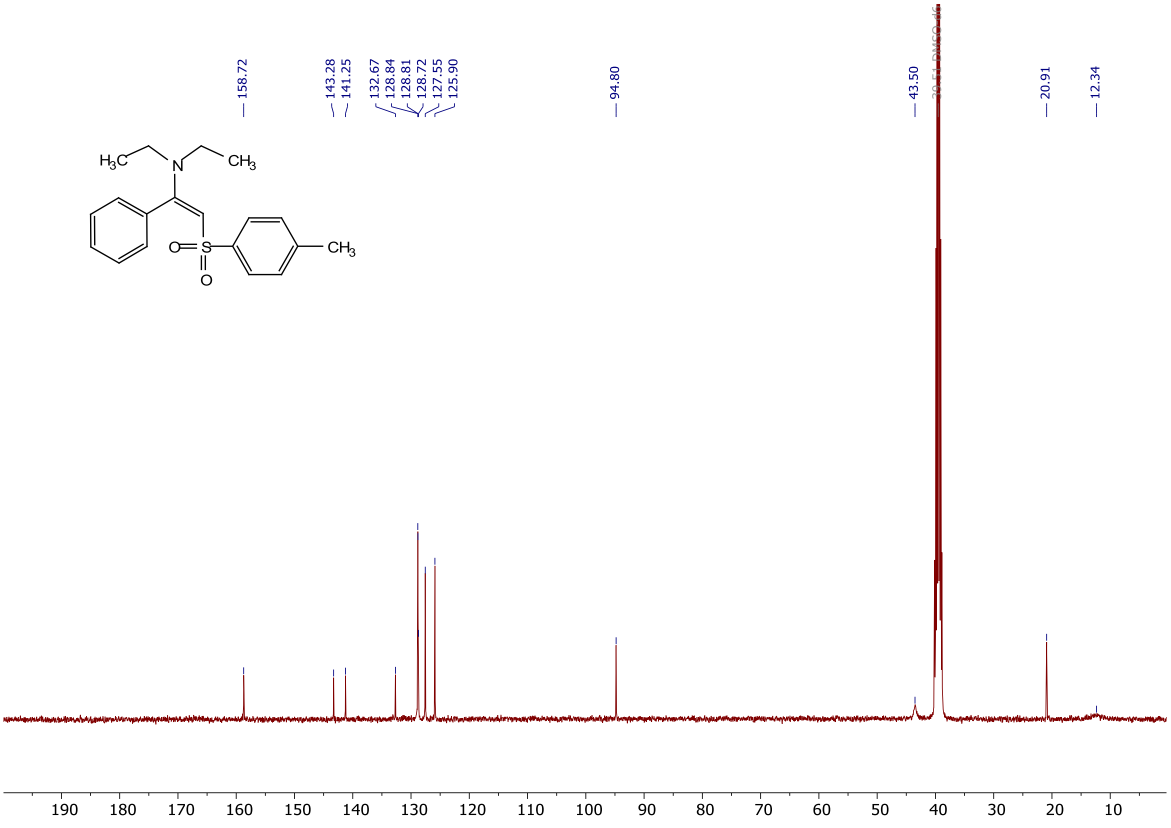

1-Ethyl-1*H*-indole (42)

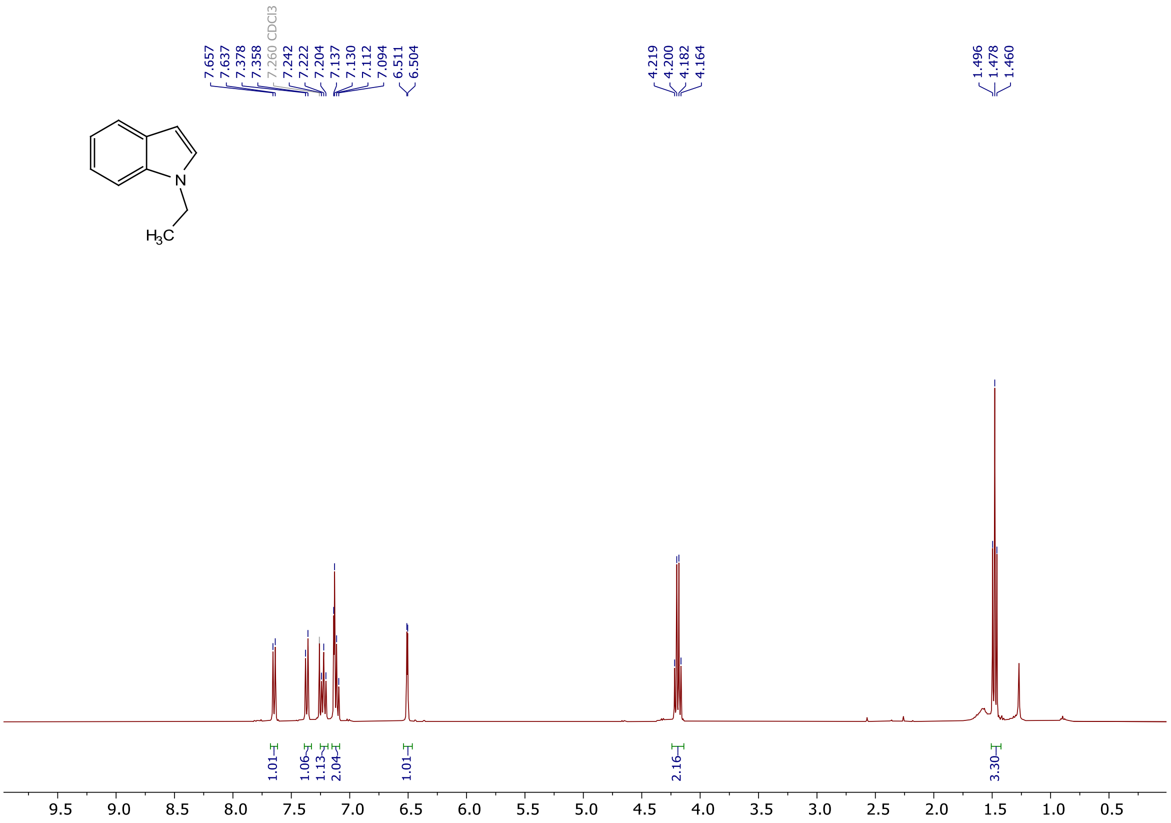

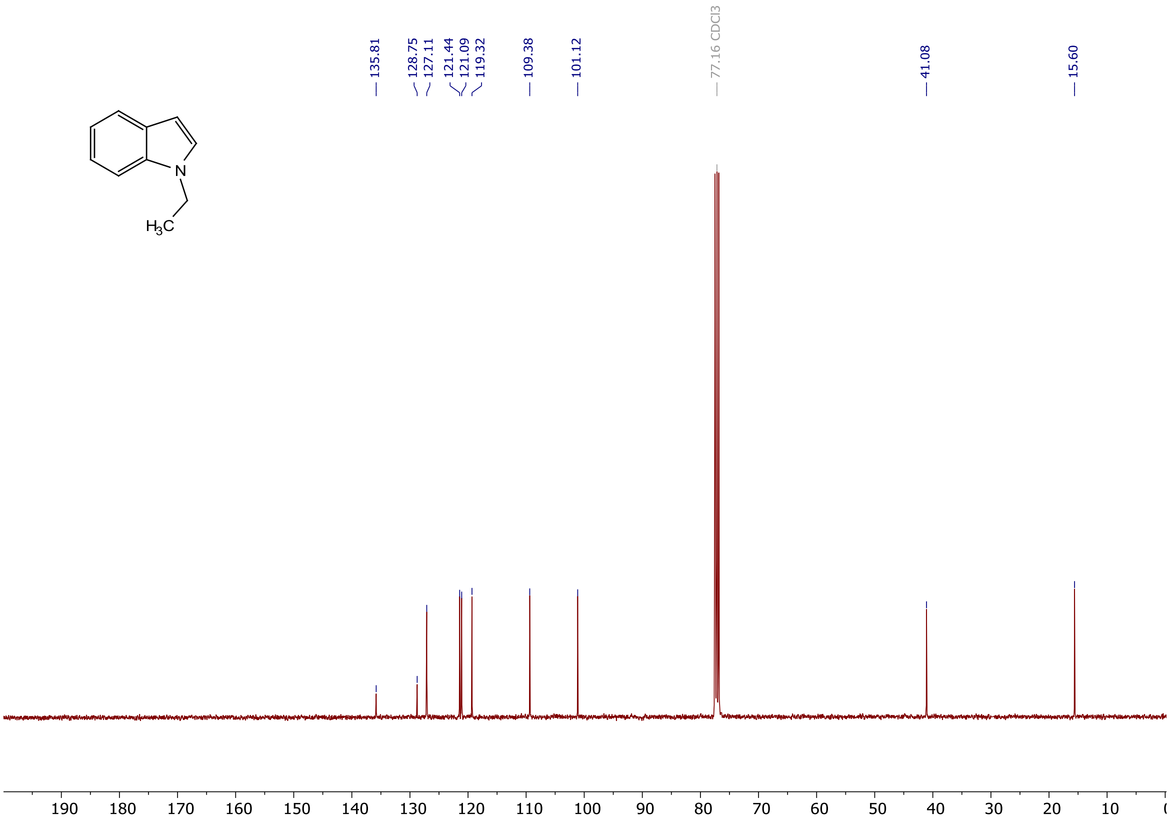

1-Ethyl-5-methyl-1*H*-indole (43)

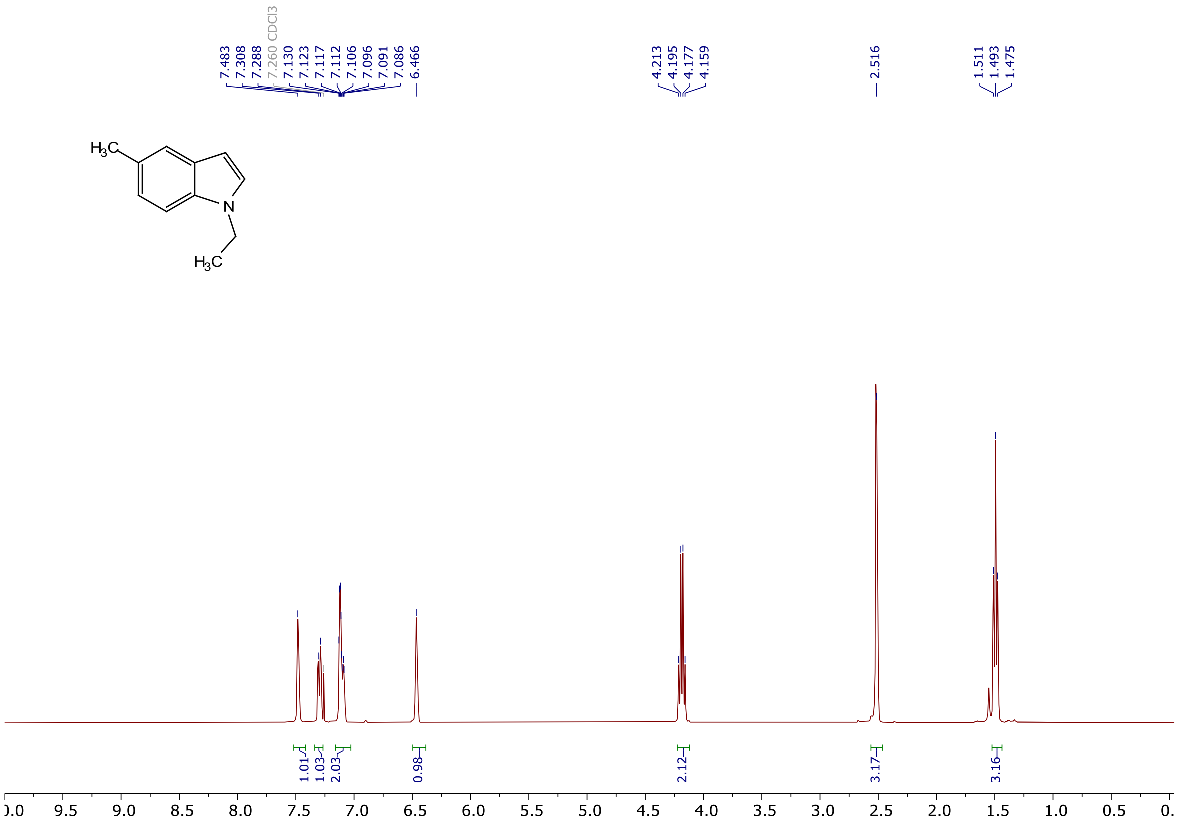

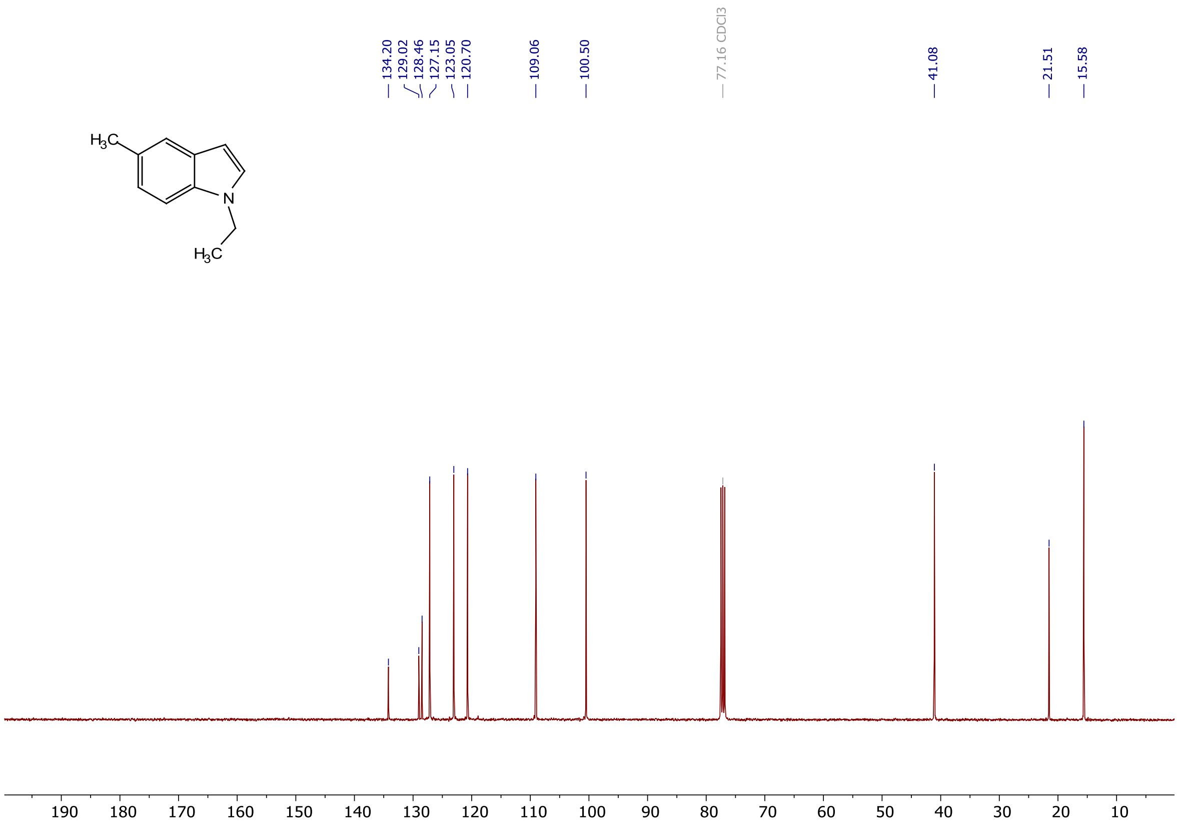

1-Ethyl-5-methoxy-1*H*-indole (44)

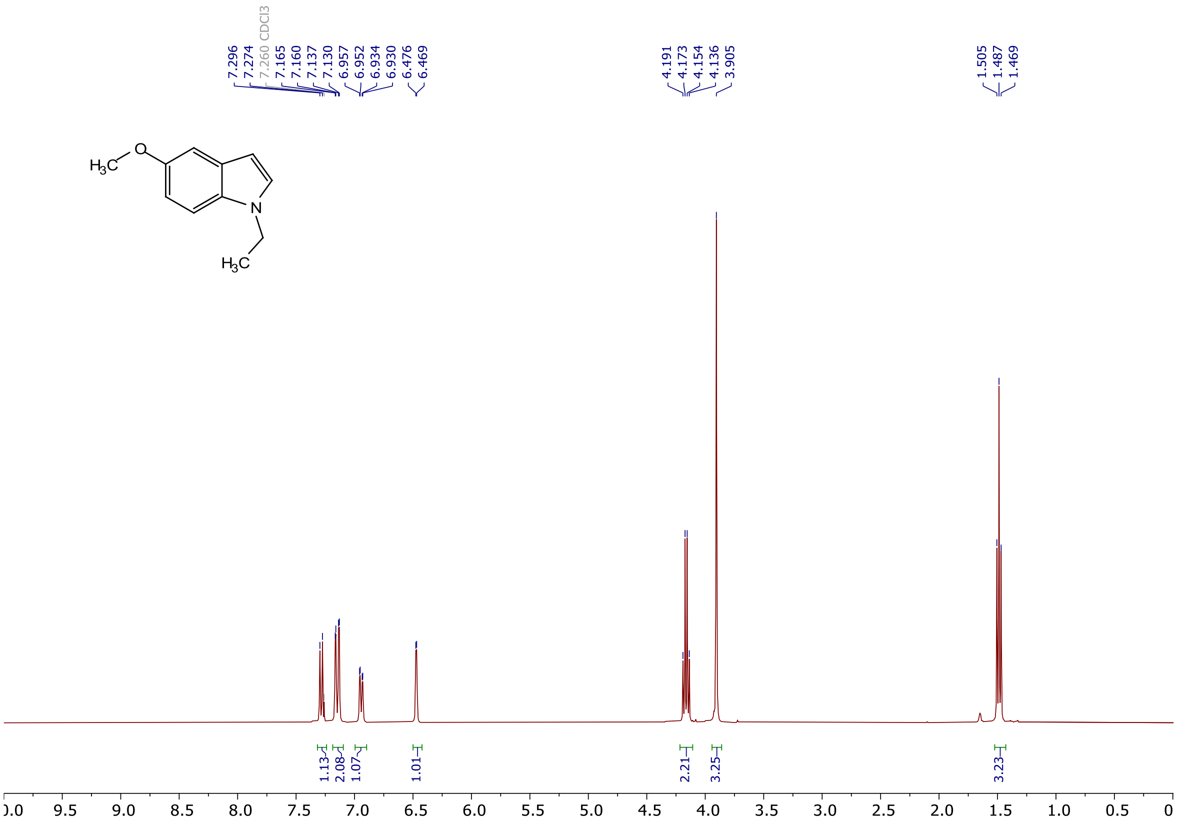

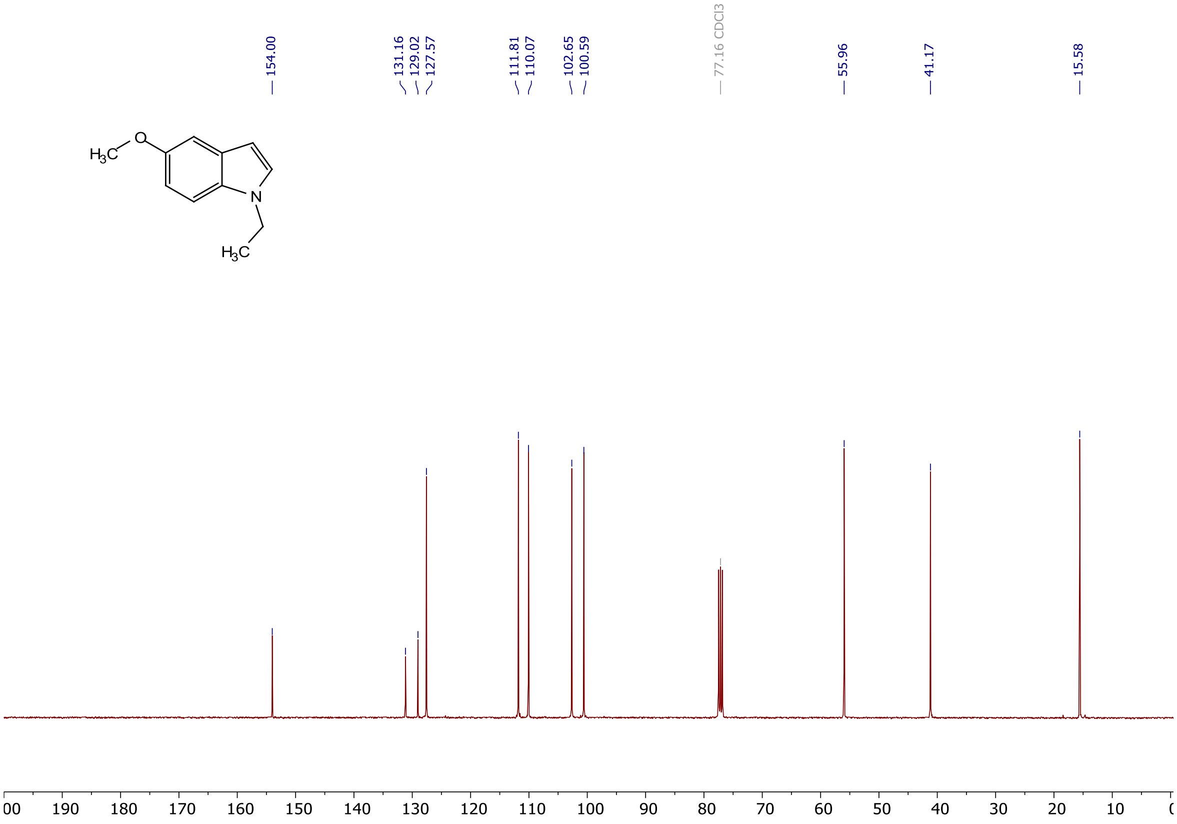

1-Ethyl-1*H*-indole-5-carbonitrile (45)

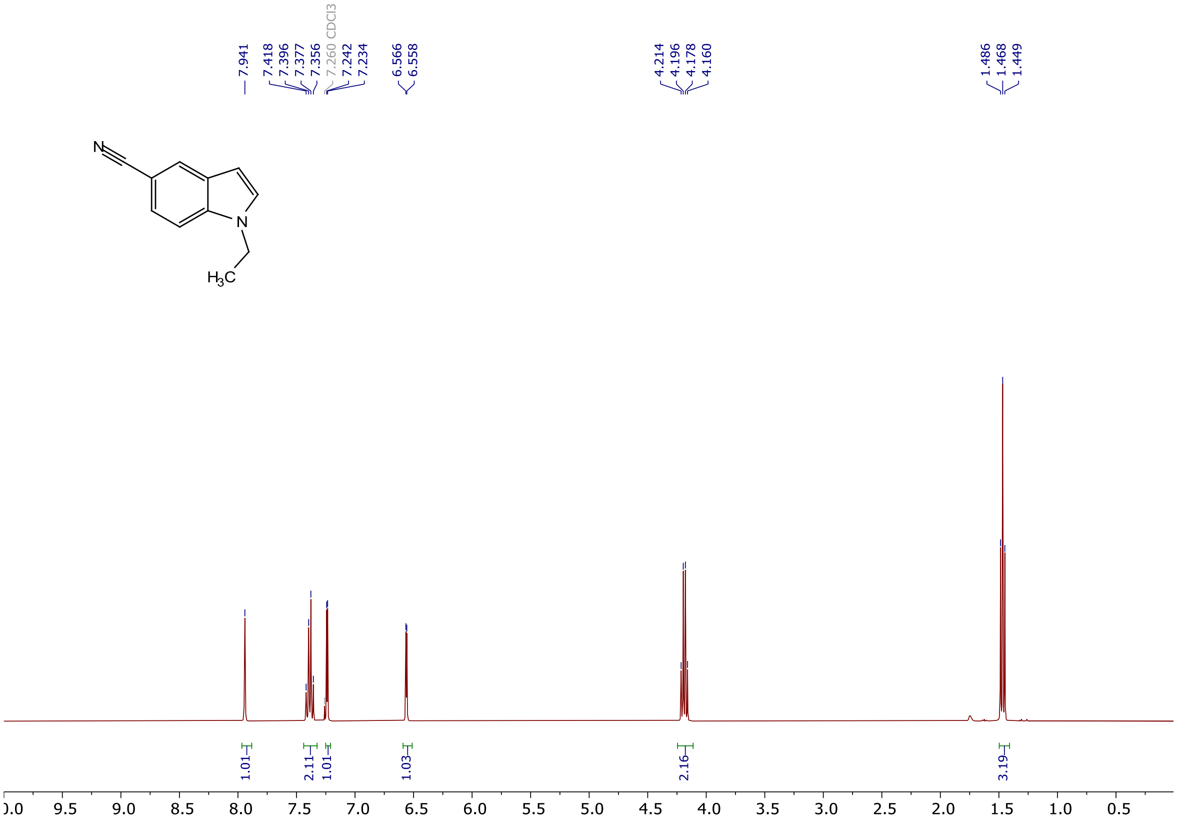

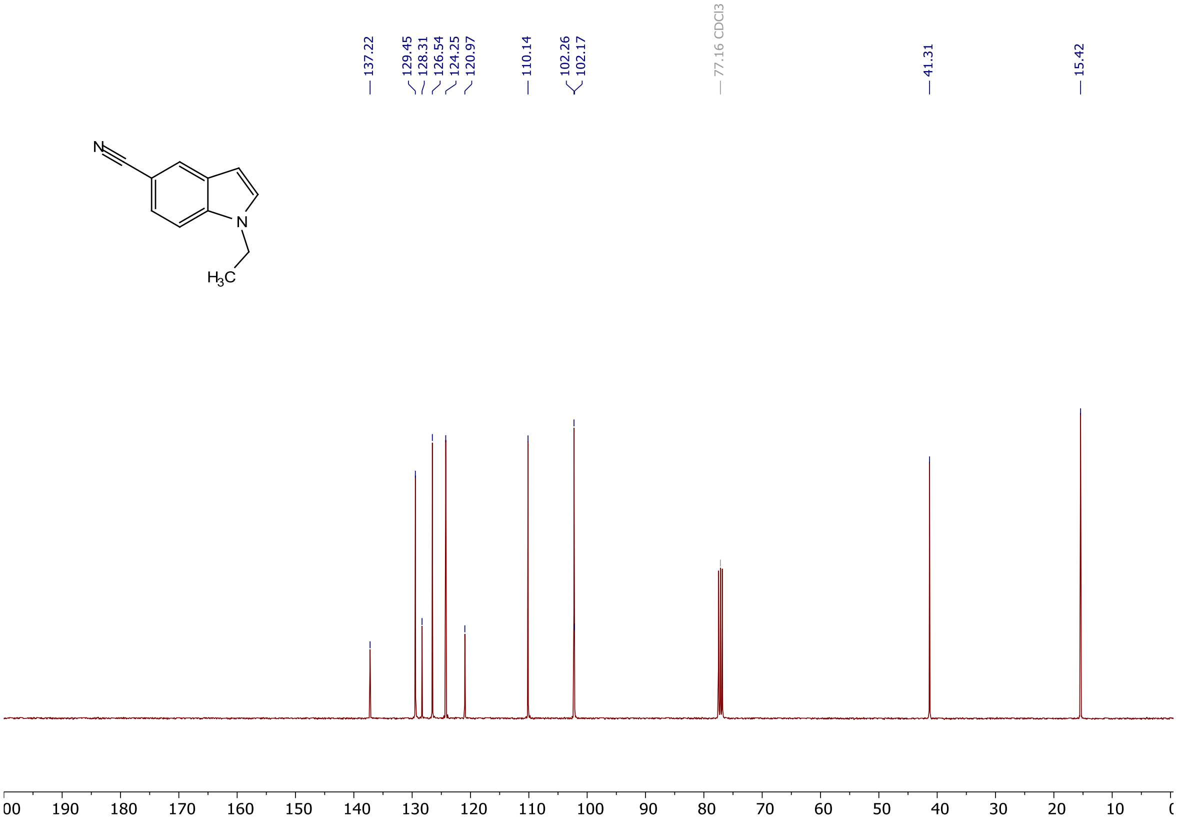

1-Phenyl-3-tosylpiperidine (46)

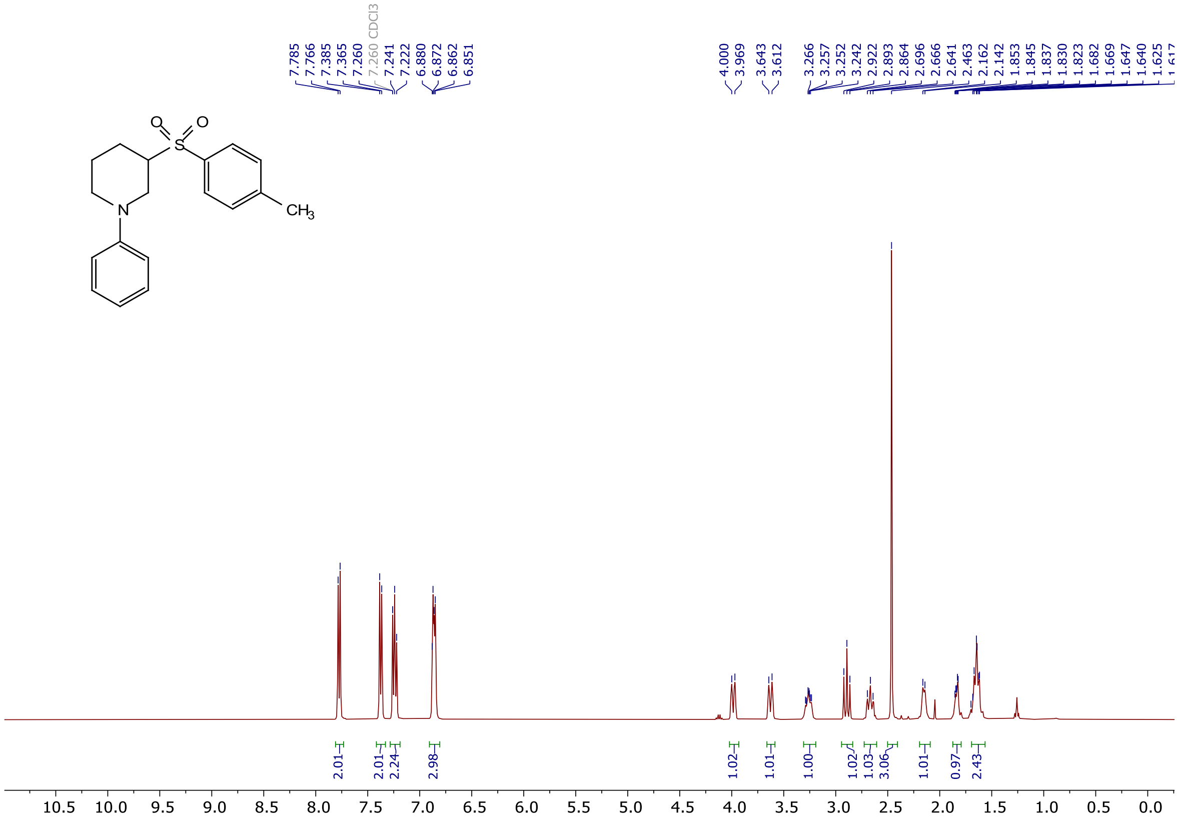

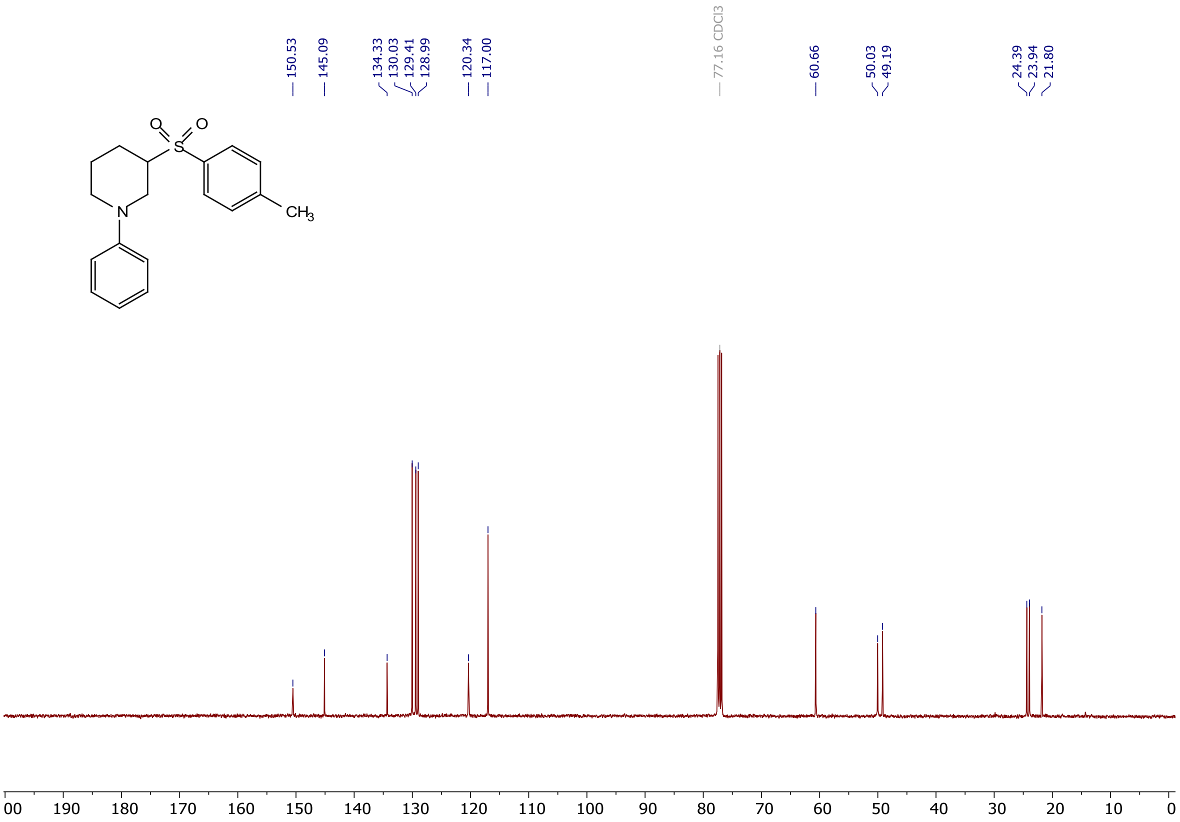

N-(4-Chloro-4-tosylbutyl)-*N*-phenylformamide (47)

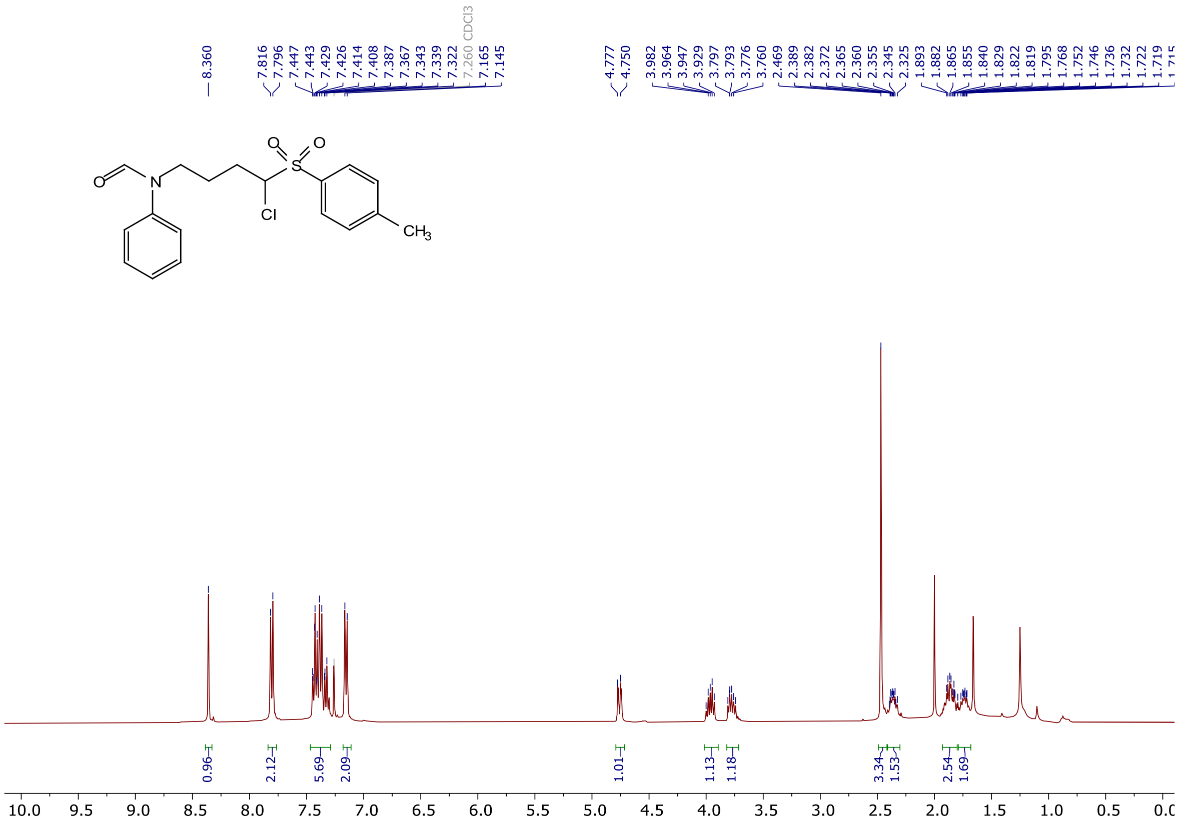

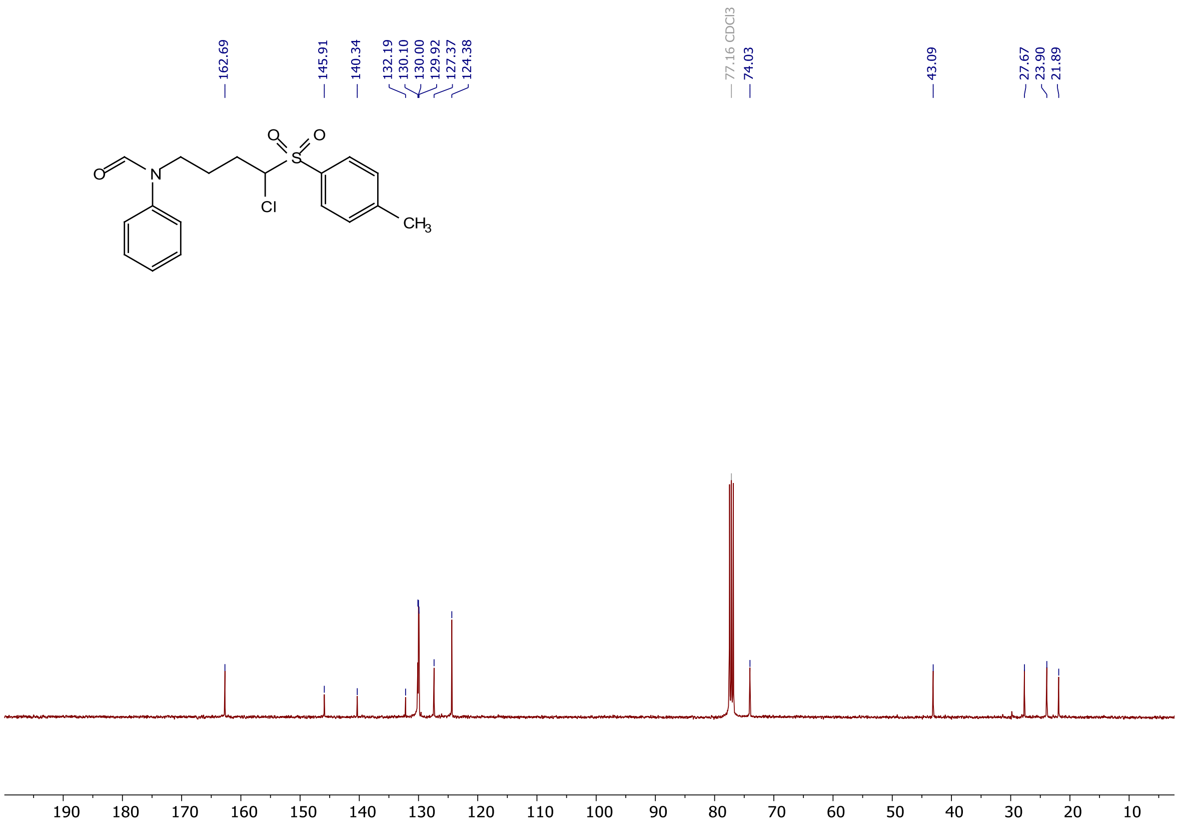

(2-Tosylethene-1,1-diyl)dibenzene

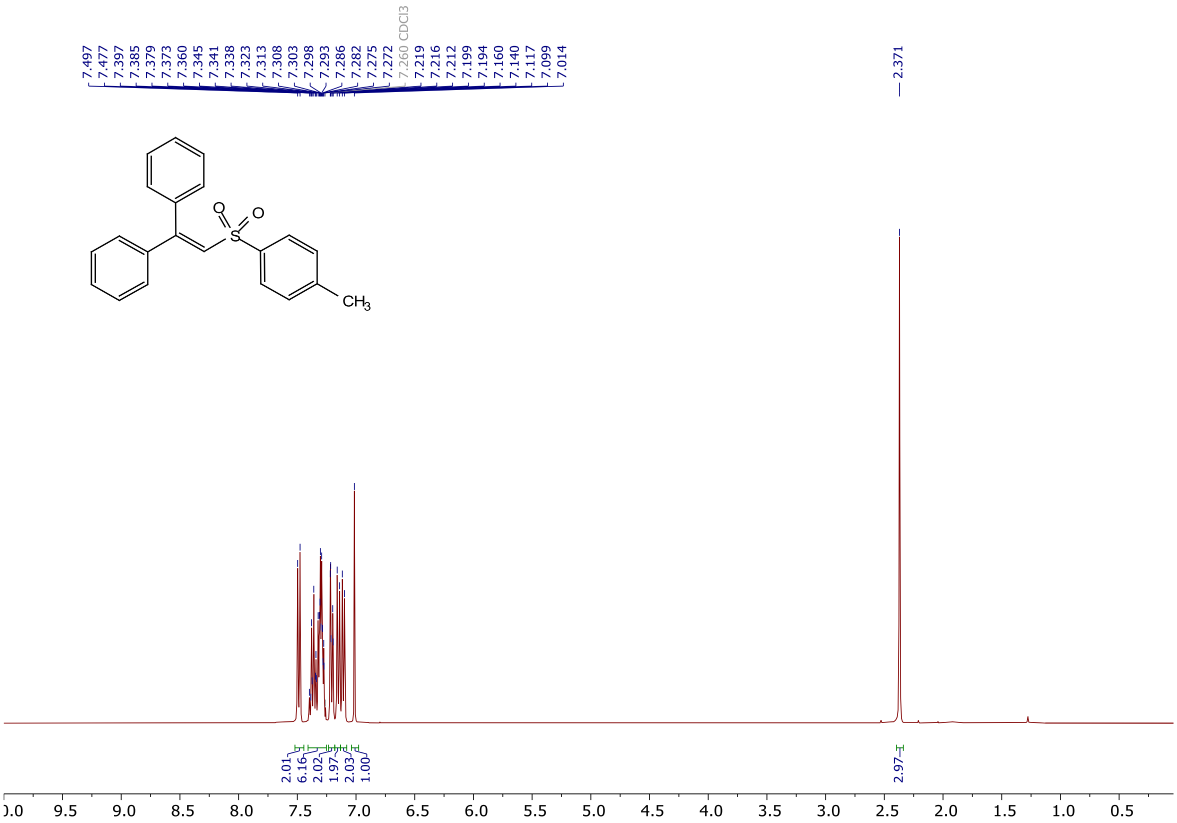

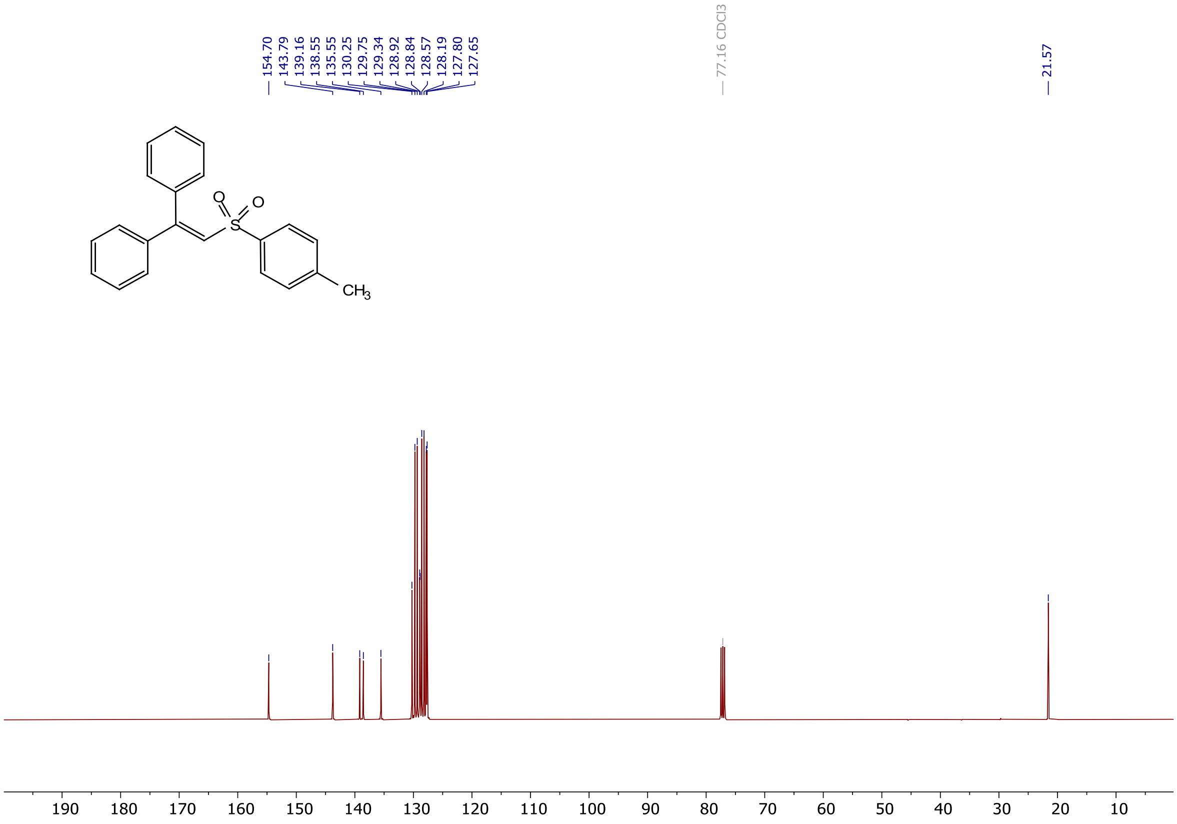

Supplementary References

1. Gaussian 09, Revision D.01, M. J. Frisch, G. W. Trucks, H. B. Schlegel, G. E. Scuseria, M. A. Robb, J. R. Cheeseman, G. Scalmani, V. Barone, B. Mennucci, G. A. Petersson, H. Nakatsuji, M. Caricato, X. Li, H. P. Hratchian, A. F. Izmaylov, J. Bloino, G. Zheng, J. L. Sonnenberg, M. Hada, M. Ehara, K. Toyota, R. Fukuda, J. Hasegawa, M. Ishida, T. Nakajima, Y. Honda, O. Kitao, H. Nakai, T. Vreven, J. A. Montgomery, Jr., J. E. Peralta, F. Ogliaro, M. Bearpark, J. J. Heyd, E. Brothers, K. N. Kudin, V. N. Staroverov, T. Keith, R. Kobayashi, J. Normand, K. Raghavachari, A. Rendell, J. C. Burant, S. S. Iyengar, J. Tomasi, M. Cossi, N. Rega, J. M. Millam, M. Klene, J. E. Knox, J. B. Cross, V. Bakken, C. Adamo, J. Jaramillo, R. Gomperts, R. E. Stratmann, O. Yazyev, A. J. Austin, R. Cammi, C. Pomelli, J. W. Ochterski, R. L. Martin, K. Morokuma, V. G. Zakrzewski, G. A. Voth, P. Salvador, J. J. Dannenberg, S. Dapprich, A. D. Daniels, O. Farkas, J. B. Foresman, J. V. Ortiz, J. Cioslowski, and D. J. Fox, Gaussian, Inc., Wallingford CT, (2013).
2. C. Adamo, V. Barone, *J. Chem. Phys.*, **110**, 6158-6169 (1999).
3. A. Schäfer, H. Horn, R. Ahlrichs, *J. Chem. Phys*. **97**, 2571-2577 (1992).
4. a) U. Haeusermann, M. Dolg, H. Stoll, H. Preuss, *Mol. Phys.* **78**, 1211-1224 (1993). b) W. Kuechle, M. Dolg, H. Stoll, H. Preuss, *J. Chem. Phys*. **100**, 7535-7542 (1994). c) T. Leininger, A. Nicklass, H. Stoll, M. Dolg, P. Schwerdtfeger, *J. Chem. Phys*. **105**, 1052-1059 (1996).
5. Y. Zhao, D. G. Truhlar, *Theor. Chem. Acc*. **120**, 215-241 (2008).
6. A. Schaefer, C. Huber, R. Ahlrichs, *J. Chem. Phys*. **100**, 5829-5835 (1994).
7. A. V. Marenich, C. J. Cramer, D. G. Truhlar, *J. Phys. Chem. B* **113**, 6378-6396 (2009).
8. a) C. P. Kelly, C. J. Cramer, D. G. Truhlar, *J. Chem. Theory Comput.* **1**, 1133–1152 (2005). b) C. P. Kelly, C. J. Cramer, D. G. Truhlar, *J. Phys. Chem. B* **110**, 16066–16081 (2006).
9. C. Y. Legault, CYLView, 1.0b; Université de Sherbrooke: Canada, (2009); http://www.cylview.org.
10. R. Kancherla, K. Muralirajan, B. Maity, C. Zhu, P. E. Krach, L. Cavallo, M. Rueping, *Angew. Chem. Int. Ed*. **58**, 3412-3416 (2019).
11. S. Tin, T. Fanjul, M. L. Clarke, *Beilstein J. Org. Chem.* **11**, 622–627 (2015).
12. J. Templ, E. Gjata, F. Getzner, M. Schnurch, *Org. Lett.* **24**, 7315–7319 (2022).
13. T. Yasushi, H. K. Tae, W. Yoshihisa, *J. Org. Chem.* **52**, 1673–1680 (1987).
14. D. Zhao, Y. Wang, M-X. Zhu, Q. Shen, L. Zhang, Y. Du, J-X. Li, *RSC Adv*. **3**, 10272–10276 (2013).
