# Supplementary material for: Mechanistic insights into excited-state palladium catalysis for C–S bond formations and dehydrogenative sulfonylation of amines
Source: Nat Commun. 2023 Oct 19;14:6622. doi: 10.1038/s41467-023-42392-2 (PMC10587301; doi:10.1038/s41467-023-42392-2)
Supplement: Supplementary file 2 — Peer Review File [file 41467_2023_42392_MOESM2_ESM.pdf]

nature portfolio

Peer Review File

Mechanistic Insights into Excited-state Palladium Catalysis for C–S bond Formations and Dehydrogenative Sulfonylation of Amines

Open Access This file is licensed under a Creative Commons Attribution 4.0 International License, which permits use, sharing, adaptation, distribution and reproduction in any medium or format, as long as you give appropriate credit to the original author(s) and the source, provide a link to the Creative Commons license, and indicate if changes were made. In the cases where the authors are anonymous, such as is the case for the reports of anonymous peer reviewers, author attribution should be to 'Anonymous Referee' followed by a clear attribution to the source work. The images or other third party material in this file are included in the article's Creative Commons license, unless indicated otherwise in a credit line to the material. If material is not included in the article's Creative Commons license and your intended use is not permitted by statutory regulation or exceeds the permitted use, you will need to obtain permission directly from the copyright holder. To view a copy of this license, visit http://creativecommons.org/licenses/by/4.0/.

Reviewers' Comments:

Reviewer #1:
Remarks to the Author:
This work by Rueping and coworkers describes a Pd-photocatalysed desaturative β-sulfonylation of tertiary amines, and reasonable mechanistic insights are presented by using detailed computational mechanistic studies in combination with time-resolved optical spectroscopy. Although the authors claim that such a process has not been reported to date, similar transformations of desaturative β-functionalization have been published by using copper catalysis (J. Org. Chem. 2018, 83, 6524), palladium catalysis (Chem. Commun. 2017, 53, 4002) and even greener electrochemical catalysis (Angew. Chem. Int. Ed. 2022, 61, e2021151). In particular, the same transformation for the construction of vinyl sulfone derivatives from tertiary amines have been reported by Fan (J. Org. Chem. 2020, 85, 15600) and Xia (Eur. J. Org. Chem. 2021, 701), respectively. Additionally, this manuscript falls short in showing the synthetic applicability of the products. When I consider the high standard of novelty required for Nature Communication, I think this work may be not suitable for publication.
Other Comments:
In the conclusion, the claim "this one-pot intermolecular HAT, oxidative dehydrogenation, and C−S cross-coupling method represent the first demonstration of the versatility of tertiary amines in cross-coupling reactions" is not true. A revision is needed.

Reviewer #2:
Remarks to the Author:
In this manuscript, Laquai, Cavallo and Rueping decribed an interesting strategy to achieve intermolecular HAT, oxidative dehydrogenation and C-S formation catalyzed by single Pd photocatalyst via the generation of two distinct metal radical hybrid species. Transient absorption spectra, detailed DFT calculations and other in-depth experiments were clearly performed to support the proposed mechanism of excited-state Pd-catalyzed dehydrogenative C-S cross-coupling reaction. This manuscript provides a good paradigm for integrating mechanistic studies into the developement of organic synthesis methodology.
I recommend publication in the Nature Communications after consideration of the minor revisions outlined below.
1) In my opinion, a consice table for optimization should be included before mechanism discussion. Similarly, schemes including some selected important mechanistic experiments from the section 5 in the SI should be considered to put into article.
2) In the sections discussing spectroscopy experiments and DFT calculations, too many Figures in the SI were cited, I think that moving the related discussion on unfavored or less favored pathways to SI would get a more concise text.

Reviewer #3:
Remarks to the Author:
In this manuscript, Profs. Laquai, Cavollo, and Rueping et. al developed a cascade intermolecular HAT, oxidative dehydrogenation, and cross-coupling reactions employing a single photocatalyst (Pd) via the generation of two metal radical hybrid species. Photo-induced Pd-catalyzed cross-coupling reactions via the single electron oxidative addition of a C−X bond (X = I, Br, and COO-NR1R2) to Pd(0) to generate Pd(I)-radical hybrid species have attracted broad attention. I believe in the overall mechanistic proposal and appreciate the detailed mechanistic studies performed, although there are many precedented reports.

The major issue is the mis-selling of the work. The key selling point I get from the title and

introduction is that “Two distinct excited-state Palladium radical hybrid species”. From the mechanistic cycle proposed in Fig.2, Int. IV (RSO2 – Pd-Cl*) Int. IV (R– Pd-Cl*) are the “excited-state” species the authors mean. Apparently, this is not correct. The photoexcited Pd(0) species undergo oxidative quenching with TsCl generating radical species (RSO2 – Pd-Cl, R– Pd-Cl), but not in so-called excited-state species labeled with “*”. No mechanistic experiments performed give evidence to excited state Pd(I). I also see a contradiction among the authors’ previous publications. In Org. Lett. 2021, 23, 6905−6910, Pd(I)-radical species is labeled as excited (*), while in Chem. Sci., 2022,13, 8583-8589 is not. In addition, it is difficult to say whether there is a significant improvement over the authors’ previous Chem. Sci. work.
From the aspect of the transformation, as cited by the authors as refs. 47-48 (Org. Biomol. Chem. 12, 9337-9340 (2014). Green Chem. 24, 1995-1999 (2022)), there are quite a lot of methods for synthesizing the product.

Some other descriptions need rephrasing which in this reviewer’s mind are not rigorous.
1. Abstract: “To date, most photocatalytic C-H cross-coupling reactions require two different transition metal complexes in order to complete the reaction cycle.” I believe the authors mean two different catalysis. One for HAT, and another for coupling. Apparently, many reports use a single organo-HAT catalyst, or a combination of organophotoredox catalysts with transition metals.
2. I would not use theThe current title is not common for a research paper. Especially, after careful evaluations of the MS, I am still not clear how the authors break what existing concept. Additionally, “excited-state Palladium radical Hybrid species” is not correct as I stated vide supra.
3. “To date, practical and sustainable methods for the transition-metal-catalyzed oxidative dehydrogenation of alkanes to alkenes are scarce” While this may true for transition-metals, this reviewer sees a lot of sustainable and practical methods for dehydrogenation, such as electrochemical methods (Angew. Chem.Int. Ed.2022,61, e202115), metal-free methods (as cited by the authors), also transition-metal-catalysis (Acc. Chem. Res. 2021, 54, 1118-1130.).
4. “Interestingly, we also observed that seven-membered N-phenyl amine and open-chain (N,N-diethyl)phenyl amine) could also be used to prepare the target compounds, vinyl-sulfonylated products (28–29), suggesting that ring strain is important for an efficient oxidative dehydrogenation process. However, this effect was not observed with aliphatic amines. For example, tri-ethyl, -butyl, and -hexyl amines undergo efficient dehydrogenative alkene functionalization to afford the more stable terminal and internal vinyl sulfones (30–31) in excellent yields.” Reads weird.

Some additional experiments might improve the scientific quality of the manuscript:
1. Deuterium experiment. It is the most straightforward way to classify a HAT path or a SET/ET path for the radical Int V generation.
2. Monitor the intermediacy of enamine 3. The authors stated “Two different orthogonal reactivities”. The two ground state Pd intermediates Tol-Pd(I)-Cl and Ts-Pd(I)-Cl are associated with each other. Is it possible that the accumulation of intermediate 3 leads to the trap by Tol-Pd(I)-Cl. Therefore, classifying “orthogonal” seems necessary.

Reviewer #4:
Remarks to the Author:
Rueping, Cavallo, Laquai and coworkers reported an excited-state Pd-catalyzed dehydrogenative sulfonylation reaction. By combining experimental and computational studies, a single photocatalyst (Pd) was shown to facilitate intermolecular HAT, oxidative dehydrogenation, and cross-coupling reactions. The experimental and computational results are well consistent, showcasing a clear description for this reaction. I recommend the publication in Nature Communications after addressing the following comments:

1) In the computed mechanism, several different types of radicals (phenyl radical, sulfonyl radical C and alkyl radical E) are proposed. What’s the difference in HAT reactivity for these radicals.

2) Also, is it a barrierless process for the radical addition to Pd(I), such as E→F and J→K?

3) The authors claim that Pd(II) species formed in the beta-H elimination step could be reduced to Pd(0) in the presence of K_2CO_3. It may be a complicated process, but it is an interesting issue. I am wondering if there are computational or experimental evidences to support this.

4) In the energy profiles shown in Fig 4 and Fig 5, there are several steps involving ligand dissociation. For PPh_3, this is reasonable for the subsequent beta-H elimination. However, since bidentate phosphine ligands (e.g., BINAP) are also effective for this reaction, it is questionable for partial ligand dissociation to promote beta-H elimination.

5) In Table S1, several Pd precursors were considered. While both Pd acetate and Pd(0) are effective, other Pd(II) precursors, such as $PdCl_2$, PdI_2 and $PdBr_2$, are less reactive. Is this because these Pd(II) species are difficult to be reduced?

Response to the reviewers

Reviewer 4:

Rueping, Cavallo, Laquai and coworkers reported an excited-state Pd-catalyzed dehydrogenative sulfonylation reaction. By combining experimental and computational studies, a single photocatalyst (Pd) was shown to facilitate intermolecular HAT, oxidative dehydrogenation, and cross-coupling reactions. The experimental and computational results are well consistent, showcasing a clear description for this reaction. I recommend the publication in Nature Communications after addressing the following comments:

We thank the reviewer for recommending to publish our manuscript in Nature Communication..

***Reviewer's Comment 1*:** In the computed mechanism, several different types of radicals (phenyl radical, sulfonyl radical C and alkyl radical E) are proposed. What's the difference in HAT reactivity for these radicals.

***Response*:** The authors thank the reviewer for constructive comments/suggestions. The following section with the Figure is added in the SI.

We have calculated HAT steps to activate α-amino-C_{sp3}–H bond of amine using different radical species (**D**, **C,** and **J**) involved in the proposed catalytic pathway. Calculations indicate that the HAT step involving the phenyl radical, **D**, is most preferred by 10.1 and 15.0 kcal/mol over the other two radicals, **C** and **J**, respectively (Scheme S3). Therefore, radical **D** is most active for the HAT step to activate the α-amino-C_{sp3}–H bond.

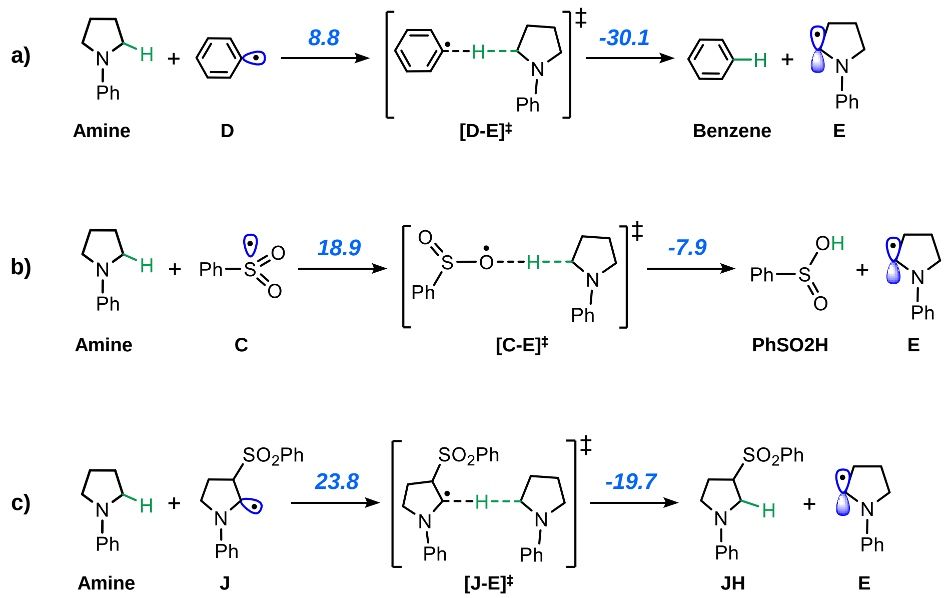

Scheme. S3. Energetics (in kcal/mol) of HAT steps by different radical species.

***Reviewer's Comment 2*:** Also, is it a barrierless process for the radical addition to Pd(I), such as E→F and J→K?

Response: After several attempts, we were unable to find transition states for E→F and J→K. These steps are the coupling of two doublet species, and thus barrierless.

Reviewer's Comment 3: The authors claim that Pd(II) species formed in the beta-H elimination step could be reduced to Pd(0) in the presence of K_2CO_3. It may be a complicated process, but it is an interesting issue. I am wondering if there are computational or experimental evidences to support this.

Response**:** Yes, we completely agree that this step is not a single-step process. We have carefully studied the literature but are unable to find similar chemical transformations. However, we have discussed this in a stepwise fashion. The following sentence along with Scheme S4 is added in SI.

Calculations suggest that the trans isomer of $(Ph_3P)_2Pd(II)HCl$ is more stable by 5.2 kcal/mol over cis isomer (**I**$_c$). The liberation of HCl from the cis isomer is thermodynamically feasible and is further accelerated in the presence of the strong base K_2CO_3 (Scheme S4).

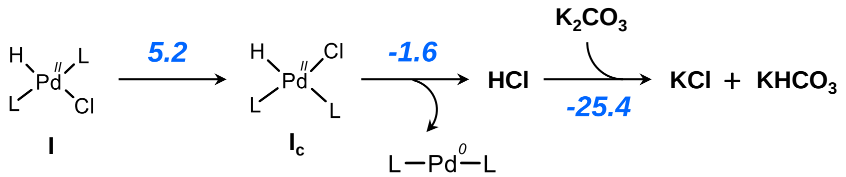

Scheme S4. Reduction of $(Ph_3P)_2Pd(II)HCl$ to $(Ph_3P)_2Pd(II)$ in presence of base K_2CO_3.

Reviewer's Comment 4: In the energy profiles shown in Fig 4 and Fig 5, there are several steps involving ligand dissociation. For PPh_3, this is reasonable for the subsequent beta-H elimination. However, since bidentate phosphine ligands (e.g., BINAP) are also effective for this reaction, it is questionable for partial ligand dissociation to promote beta-H elimination.

Response**:** We thank the reviewer for this important point. We have added the following text and Fig. S5 in SI.

We have considered both the pathways of β-H elimination from intermediate **F** and the PPh_3 dissociated from, **G** (Fig. S8). Calculated results indicate that the ligand-dissociated pathway is the preferred one and has a free energy barrier of 21.8 kcal/mol. However, the alternative pathway, having two PPh_3 ligands, requires a barrier of 23.9 kcal/mol and is also viable at the current reaction conditions. Therefore, in the presence of BINAP ligand, the β-H elimination step is expected to be feasible. To support this statement, we have performed further calculations incorporating the BINAP ligand. Consistently, the calculated free energy barrier is 23.5 kcal/mol, which can be achieved at this reaction condition, and thus the step should be feasible (Scheme. S5).

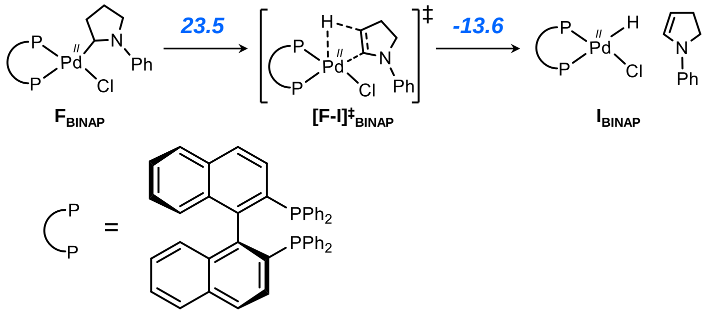

Scheme S5. Energetics (in kcal/mol) of β-H elimination step in presence of BINAP ligand

Reviewer's Comment 5: In Table S1, several Pd precursors were considered. While both Pd acetate and Pd(0) are effective, other Pd(II) precursors, such as $PdCl_2$, PdI_2 and $PdBr_2$, are less reactive. Is this because these Pd(II) species are difficult to be reduced?

Response: Compared to $Pd(OAc)_2$, the reduction of other Pd(II) salts by phosphine ligands is slightly more difficult. Therefore the yields with [$Pd(OAc)_2$ + PPh_3] are superior compared to other Pd(0) precursors.

Reviewer 3:

In this manuscript, Profs. Laquai, Cavollo, and Rueping et. al developed a cascade intermolecular HAT, oxidative dehydrogenation, and cross-coupling reactions employing a single photocatalyst (Pd) via the generation of two metal radical hybrid species. Photo-induced Pd-catalyzed cross-coupling reactions via the single electron oxidative addition of a C−X bond (X = I, Br, and COO-NR1R2) to Pd(0) to generate Pd(I)-radical hybrid species have attracted broad attention. I believe in the overall mechanistic proposal and appreciate the detailed mechanistic studies performed, although there are many precedented reports.

We thank the reviewer for reviewing our manuscript and valuable comments.

Reviewer's Comment 1: The major issue is the mis-selling of the work. The key selling point I get from the title and introduction is that "Two distinct excited-state Palladium radical hybrid species". From the mechanistic cycle proposed in Fig.2, Int. IV (RSO2 – Pd-Cl*) Int. IV (R– Pd-Cl*) are the "excited-state" species the authors mean. Apparently, this is not correct. The photoexcited Pd(0) species undergo oxidative quenching with TsCl generating radical species (RSO2 – Pd-Cl, R– Pd-Cl), but not in so-called excited-state species labeled with "*". No mechanistic experiments performed give evidence to excited state Pd(I). I also see a contradiction among the authors' previous publications. In Org. Lett. 2021, 23, 6905−6910, Pd(I)-radical species is labeled as excited (*), while in Chem. Sci., 2022,13, 8583-8589 is not. In addition, it is difficult to say whether there is a significant improvement over the authors' previous Chem. Sci. work.

Response: We are grateful for the constructive suggestions. We do agree that the distinct triplet species, namely RSO_2 – Pd-Cl and R– Pd-Cl, are not excited-state intermediates in the excited state catalysis. We have revised this and removed the asterisks. Additionally, we have diligently reviewed our revised manuscript to ensure clarity and eliminate any potential confusion for readers.

Now we changed the title from "Two distinct excited-state Palladium radical hybrid species in one compo" to "Mechanistic Insights into Excited-state Palladium Catalysis in C–S bond Formation and Dehydrogenative Sulfonylation of Amines".

In comparison to the previous reports, there is a significant novelty in the present work. We focus on the mechanistic aspects of excited-state Pd-catalysis using the combination of DFT studies and advanced spectroscopic methods. In addition, C–S bond formations via a HAT process in excited-state Pd-catalysis is to our knowledge also not known.

Reviewer's Comment 2: From the aspect of the transformation, as cited by the authors as refs. 47-48 (Org. Biomol. Chem. 12, 9337-9340 (2014). Green Chem. 24, 1995-1999 (2022)), there are quite a lot of methods for synthesizing the product.

Response: The mentioned manuscripts have been cited in the manuscript text and the references have been added accordingly.

However, the key point in our manuscript are the detailed mechanism studies of the excited-state Pd-catalysis exemplified in the C-S bond formation. Both have not been reported to date.

We present the observation of the one-electron oxidative addition of aryl sulfonyl chloride to Pd(0) at room temperature. Conventional two-electron oxidative addition of palladium to sulfonyl chloride necessitates elevated temperatures (up to 140 °C). However, our approach enables facile execution at room temperature under visible light, an advancement in palladium chemistry. The combination of experimental and computational mechanistic studies, along with comprehensive spectroscopic measurements, employed in our current work will certainly be of interest to a broader readership as it covers new reaction development, mechanistic aspects as well as DFT studies and advanced spectroscopy to gain valuable insights into the photophysics and mechanism of excited-state Pd catalysis which is an important and topical area.

Reviewer's Comment 3: Abstract: "To date, most photocatalytic C-H cross-coupling reactions require two different transition metal complexes in order to complete the reaction cycle." I believe the authors mean two different catalysis. One for HAT, and another for coupling. Apparently, many reports use a single organo-HAT catalyst, or a combination of organophotoredox catalysts with transition metals.

Response: Yes, indeed. In C−H cross-coupling reactions, a photocatalyst is typically employed in conjunction with either an external organo-HAT reagent or a metal complex (such as a nickel bromide complex which could release bromine radical) to facilitate the HAT process. Photochemical cross-coupling reactions have been established based on this principle. However, to the best of our knowledge, substrates that can serve as both HAT agents and cross-coupling reagents is very rare. Within this context, our current protocol demonstrates the dual functionality of aryl sulfonyl chlorides as an HAT agent and C–S cross-coupling reagent, even at room temperature.

Reviewer's Comment 4: I would not use the current title is not common for a research paper. Especially, after careful evaluations of the MS, I am still not clear how the authors break what existing concept. Additionally, "excited-state Palladium radical Hybrid species" is not correct as I stated vide supra.

Response: Indeed, we have revised the title of the manuscript. Thank you for raising this point!

Reviewer's Comment 5: "To date, practical and sustainable methods for the transition-metal-catalyzed oxidative dehydrogenation of alkanes to alkenes are scarce" While this may true for transition-metals, this reviewer sees a lot of sustainable and practical methods for dehydrogenation, such as electrochemical methods (Angew. Chem.Int. Ed.2022,61, e202115), metal-free methods (as cited by the authors), also transition-metal-catalysis (Acc. Chem. Res. 2021, 54, 1118-1130.).

Response: We have revised the sentence and incorporated the references.

Reviewer's Comment 6: "Interestingly, we also observed that seven-membered *N*-phenyl amine and open-chain (N,N-diethyl)phenyl amine) could also be used to prepare the target compounds, vinyl-sulfonylated products (28–29), suggesting that ring strain is important for an efficient oxidative dehydrogenation process. However, this effect was not observed with aliphatic amines. For example, tri-ethyl, -butyl, and -hexyl amines undergo efficient dehydrogenative alkene functionalization to afford the more stable terminal and internal vinyl sulfones (30–31) in excellent yields." Reads weird.

Response: We apologize for the confusion. As per the reviewer's suggestion, we revised the above sentence to "Interestingly, we also observed that seven-membered *N*-phenyl amine and open-chain (*N*,*N*-diethyl)phenyl amine) could also lead to the vinyl-sulfonylated products (**28–29)** in 81% and 62% yields, respectively. The reactivity difference suggests that electron-rich amines exhibit high efficiency in the oxidative dehydrogenation process. Likewise, we also noticed that simple electron-rich aliphatic amines, such as triethyl, butyl, and hexyl amines, undergo efficient dehydrogenative alkene functionalization reactions, resulting in the formation of more stable vinyl sulfones (**30–31**) with excellent yields (page 6, paragraph 1)."

Reviewer's Comment 7: Deuterium experiment. It is the most straightforward way to classify a HAT path or a SET/ET path for the radical Int V generation.

Response: In response to the reviewer's recommendations, we conducted deuterium experiments to classify a HAT or a SET/ET path for the radical intermediate **V** generation during the reaction (Fig. 7c). Our findings revealed product distribution ratios of 3.27 (trial-1) and 3.46 (trial-2). The observed high P_H/P_D ratio indicates that the C–H bond-breaking process is predominantly achieved through a hydrogen atom transfer (HAT) pathway. On the other hand, the oxidation of the amine to generate intermediate **V** (as shown in Fig. 2) via Pd(I)Cl intermediate through SET is not expected to occur kinetically under standard reaction conditions. Additionally, we have also included the final sulfonylated product of d-15 triethyl amine in the revised SI (Compound **30-D**). We have checked the feasibility of single-electron oxidation of N-phenyl pyrrolidine by DFT analysis. The calculated energy values indicate that this electron transfer is unfavored due to very high endergonicity. We added Fig. S5 in the revised SI. Detailed discussions regarding these findings have been included in the revised manuscript on page 9, first paragraph.

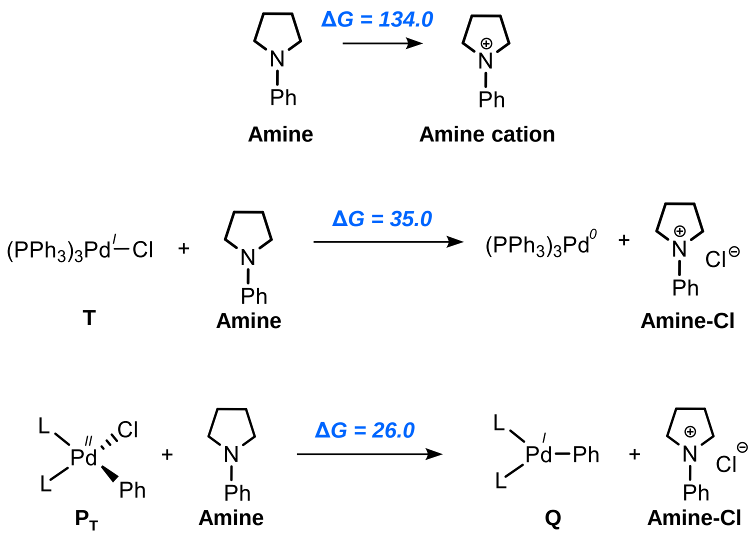

Fig. S5. Energetics (in kcal/mol) of single-electron oxidation of N-phenyl pyrrolidine by Pd(I) and Pd(II) complexes. Calculated energy values indicate that both the Pd(I) and Pd(II) complexes are unable to oxidize the N-phenyl pyrrolidine due to of high endergonicity.

Reviewer's Comment 8: Monitor the intermediacy of enamine 3. The authors stated "Two different orthogonal reactivities". The two ground state Pd intermediates Tol-Pd(I)-Cl and Ts-Pd(I)-Cl are associated with each other. Is it possible that the accumulation of intermediate 3 leads to the trap by Tol-Pd(I)-Cl. Therefore, classifying "orthogonal" seems necessary.

Response: As suggested by the reviewer, we conducted an arylation reaction using *N*,*N*-diethyl-1-phenylethen-1-amine and 1-iodo-4-methylbenzene under the specified reaction conditions (Scheme S10). However, no significant results were observed, indicating that the Tol-Pd(I)-Cl intermediate is limited to functioning solely as a hydrogen atom transfer (HAT) process rather than facilitating an arylation reaction. In order to enhance clarity, we have revised the manuscript by removing the term "orthogonal" in conclusion part.

Reviewer 2:
In this manuscript, Laquai, Cavallo and Rueping decribed an interesting strategy to achieve intermolecular HAT, oxidative dehydrogenation and C-S formation catalyzed by single Pd photocatalyst via the generation of two distinct metal radical hybrid species. Transient absorption spectra, detailed DFT calculations and other in-depth experiments were clearly performed to support the proposed mechanism of excited-state Pd-catalyzed dehydrogenative C-S cross-coupling reaction. This manuscript provides a good paradigm for integrating mechanistic studies into the developement of organic synthesis methodology. I recommend publication in the Nature Communications after consideration of the minor revisions outlined below.

We thank the reviewer for recommending publishing in Nature Communication.

Reviewer's Comment 1**:** In my opinion, a concise table for optimization should be included before mechanism discussion. Similarly, schemes including some selected important mechanistic experiments from the section 5 in the SI should be considered to put into the article.

Response: As per the suggestion of the reviewer, we included both the optimization table (Table 1) and some important mechanistic experiments with discussions (Figure 7) in the revised manuscript (page 9, paragraph 1).

Reviewer's Comment 2**:** In the sections discussing spectroscopy experiments and DFT calculations, too many Figures in the SI were cited, I think that moving the related discussion on unfavored or less favored pathways to SI would get a more concise text.

Response: We thank the reviewer for this comment. The proposed C-S bond formation by excited state Pd catalysis is new and detailed mechanistic investigations were required which resulted in multiple figures (feasible and non-feasible pathways). We agree with the reviewer and moved the corresponding text and figures of the corresponding to non-feasible pathways to the SI.

Reviewer 1:
Reviewer's Comment 1**:** This work by Rueping and coworkers describes a Pd-photocatalysed desaturative β-sulfonylation of tertiary amines, and reasonable mechanistic insights are presented by using detailed computational mechanistic studies in combination with time-resolved optical spectroscopy. Although the authors claim that such a process has not been reported to date, similar transformations of desaturative β-functionalization have been published by using copper catalysis (J. Org. Chem. 2018, 83, 6524), palladium catalysis (Chem. Commun. 2017, 53, 4002) and even greener electrochemical catalysis (Angew. Chem. Int. Ed. 2022, 61, e2021151). In particular, the same transformation for the construction of vinyl sulfone derivatives from tertiary amines have been reported by Fan (J. Org. Chem. 2020, 85, 15600) and Xia (Eur. J. Org. Chem. 2021, 701), respectively. Additionally, this manuscript falls short in showing the synthetic applicability of the products. When I consider the high standard of novelty required for Nature Communication, I think this work may be not suitable for publication.

Response: We thank the reviewer for the comments. It seems to be a misunderstanding. We have not claimed that the products cannot be made otherwise. It is also not our aim to compare methods including oxidative metal catalysis or electrochemical processes with our reported mechanism study on excited state Pd catalysis. (However, if we would compare our work including all aspects (safety, CapEx/OpEx, applicability/scope) as well as scientific knowledge gain, our new methodology is certainly of general interest).

We here present the observation of the one-electron oxidative addition of aryl sulfonyl chloride to Pd(0) at room temperature. Conventional two-electron oxidative addition of palladium to sulfonyl chloride necessitates elevated temperatures (up to 140 °C). Our approach enables facile execution at room temperature under visible light and shows the different intermediates in the catalytic cycle which adds to the understanding and an advancement of palladium chemistry. The combination of experimental and computational mechanistic studies, along with comprehensive spectroscopic measurements, employed in our current work will certainly be of interest to a broader readership as it covers new reaction development, mechanistic aspects as well as DFT studies and advanced spectroscopy to gain valuable insights into the photophysics and mechanism of excited-state Pd catalysis, which is an important and topical area. We trust, our work will have an impact and it will be taken as a reference for further understanding excited-state catalysis.

Reviewers' Comments:

Reviewer #1:
Remarks to the Author:
The authors have addressed my correction as well as that of the other reviewers, but I continue to believe that the novelty is limited as there are quite a lot of methods for synthesizing the products. However, as novelty is highly subjective and mine is the minority opinion, I am ready to concede to the majority opinion of the other reviewers. In consideration of the combination of DFT studies and advanced spectroscopic methods, there is an obvious novelty in the understanding of excited-state Pd-catalysis. In principle, I agree to recommend publication in Nature Communications as is.

Reviewer #2:
Remarks to the Author:
The authors have properly responded to the points raised in my previous review. I recommend publication in Nature Communications.

Reviewer #3:
Remarks to the Author:
The authors have addressed the comments properly. I would recommend the publication of this paper at this stage. Congratulations!

Reviewer #4:
Remarks to the Author:
All my concerns have been addressed.
